# The pan-cancer pathological regulatory landscape

**DOI:** 10.1038/srep39709

**Published:** 2016-12-21

**Authors:** Matias M. Falco, Marta Bleda, José Carbonell-Caballero, Joaquín Dopazo

**Affiliations:** 1 Computational Genomics Department, Centro de Investigación Príncipe Felipe (CIPF), Valencia, 46012, Spain; 2 Department of Medicine, University of Cambridge, School of Clinical Medicine, Addenbrooke’s Hospital, Hills Road, CB2 0QQ, Cambridge, United Kingdom; 3 Functional Genomics Node (INB-ELIXIR-es), C/Eduardo Primo Yufera 3, Valencia, 46012, Spain; 4 Bioinformatics in Rare Diseases (BiER), Centro de Investigación Biomédica en Red de Enfermedades Raras (CIBERER), C/Eduardo Primo Yufera 3, Valencia, 46012, Spain

## Abstract

Dysregulation of the normal gene expression program is the cause of a broad range of diseases, including cancer. Detecting the specific perturbed regulators that have an effect on the generation and the development of the disease is crucial for understanding the disease mechanism and for taking decisions on efficient preventive and curative therapies. Moreover, detecting such perturbations at the patient level is even more important from the perspective of personalized medicine. We applied the Transcription Factor Target Enrichment Analysis, a method that detects the activity of transcription factors based on the quantification of the collective transcriptional activation of their targets, to a large collection of 5607 cancer samples covering eleven cancer types. We produced for the first time a comprehensive catalogue of altered transcription factor activities in cancer, a considerable number of them significantly associated to patient’s survival. Moreover, we described several interesting TFs whose activity do not change substantially in the cancer with respect to the normal tissue but ultimately play an important role in patient prognostic determination, which suggest they might be promising therapeutic targets. An additional advantage of this method is that it allows obtaining personalized TF activity estimations for individual patients.

Transcription factors (TFs) play a crucial role in the dynamic regulation of the gene expression program^1^. The knowledge cumulated in the last years on diverse cellular gene expression programs has drastically increased our understanding of the effects of dysregulation of gene expression in disease. In fact, a broad range of diseases and syndromes, including cancer^2^, are caused by mutations that affect TFs either directly or indirectly, by affecting cofactors, regulatory sequences, chromatin regulators, and noncoding RNAs that interact with these regions^3^. Specifically, dysregulations or changes in the activation status of distinct TFs are known to be linked to a number of cancers^4^,^5^,^6^. Actually, many oncogenes and tumour suppressor genes, including the well-known P53 gene^7^, are in fact^8^ TFs. Moreover, many cancer treatments are essentially transcriptional interventions^9^. Thus, hormonal therapies in breast and prostate cancers to block tumour progression are classical examples. More sophisticated interventions are the inhibition of global epigenomic regulators like *BRD4*^10^. Consequently, understanding the determinants of the transcriptional changes leading to disease states in patients is a prerequisite to restore the normal functions of a cell or a tissue.

Alterations in the transcriptional regulatory network due to perturbed TF activity cause the dysregulation of gene expression observed during cancer progression. Different reverse engineering methods^11^,^12^,^13^,^14^,^15^,^16^,^17^ have been proposed to infer the specific TF activity that accounts for the observed differential expression across conditions. Reverse engineering methods use the transcription level of a TF to estimate its activity by calculating different types of correlation to its corresponding target genes. However, using TF expression levels as proxies of their activities can be misleading by several reasons. Firstly, the mRNA expression levels of many TFs are often relatively low compared to other genes, which increase the uncertainty of the corresponding measurements. Secondly, the regulation of TFs at the protein level has shown to be more relevant than changes at the mRNA level, as demonstrated for example in hypoxia-inducible factors^18^ and p53^19^. Moreover, the binding of a TF to the corresponding TFBS does not necessarily imply a transcriptional activity because post-transcriptional modifications and some extra co-factors may be required to promote gene expression^20^,^21^.

As a consequence of this, TF expression levels cannot be considered good descriptors of their activity. Contrarily, the expression levels of the TF’s targets, in which all the above mentioned effects are integrated, seem to be a more reasonable readout of TF activity. Despite the simplicity of this idea and its enormous potential, only a few algorithmic proposals have been made that exploit TF’s target expression levels to infer the corresponding TF activities, such as BASE^22^, RENATO^23^, REACTIN^24^, RABIT^25^ or others^26^. These methods have been applied to the study of survival in breast cancer^27^ or to obtain signatures of tumour stage in kidney renal clear cell carcinoma^28^.

Here we use a simple but efficient method to systematically detect TFs with altered activity by studying the activity of their corresponding target genes across a total of 5607 samples covering eleven cancer types. This study allowed us to produce the first comprehensive catalogue of TFs with activity altered across a broad spectrum of cancer types. Since the method used can also return personalized values of TF activity for each patient, we could also identify a number of TFs whose altered activity was significantly associated to patient’s survival, demonstrating their relevance in cancer progression and their potential as therapeutic targets.

## Results and Discussion

### Changes in TF activity across the different cancers

**Figure 1:**
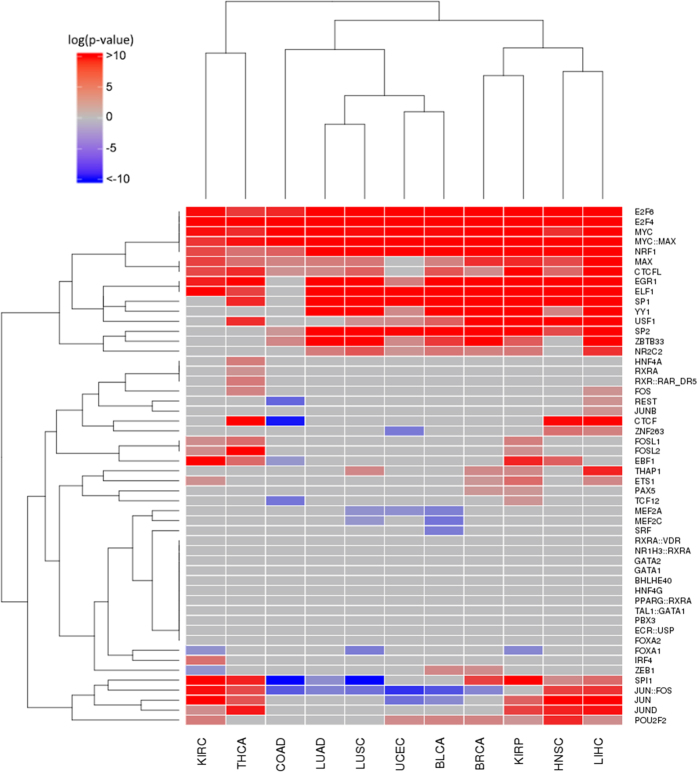
Change of TF activity in the different cancers studied. Cells in red indicate a significant increased activity of the TF in the cancer with respect to the corresponding normal tissue, according to the TFTEA, cells in blue indicate a significant decreased activity and cells in grey indicate that no significant change in activity was detected. Columns correspond to cancers and rows to TFs.

Raw RNA-seq counts for all the eleven cancers studied (Table 1) were normalized as described in Methods and tumour samples were compared to their normal tissue counterparts to obtain lists of genes differentially expressed. TF Target Enrichment Analysis (TFTEA) was applied to these lists ranked by value of the statistic. Figure 1 show changes in the activity of the different TFs when cancers are compared to their corresponding normal tissues. The predominant observed behaviour is the increase in TF activity. Actually, a set of TFs (*E2F6, E2F4, MYC, MYC:MAX* and *NRF1*) are always significantly more active in cancers than in normal tissues, and others (*EGR1, ELF1, SP1, YY1, USF1, SP2, ZBTB33, MAX, CTCFL* and *NR2C2*) are significantly active in almost all the cancers with a few exceptions, which suggest for them an important role in cancer development and progression. Actually, all of them appear in the COSMIC database^29^ and some of them are well-known oncogenes such as *MYC*^30^,^31^,^32^, *MAX* and *MYC:MAX*^33^, or proteins of the *E2F* family^34^, whose over-expression induces uncontrolled cell proliferation because they are TFs located upstream in pathways that control cell cycle^35^, being also considered prognostic factors^36^. The *YY1* TF is a multifunctional protein that regulates various processes of development and differentiation and have a clear involvement in tumorigenesis, having been proposed as potential prognostic marker of diverse cancers^37^. *SP1* and *SP2* regulate many of the genes involved in the Warburg effect^38^, a well-known cancer hallmark^39^. Actually, high levels of *SP1* protein are considered a negative prognostic factor for several cancers^40^,^41^.

There are also a few TFs that show simultaneously significant, though opposite, behaviours across the studied cancers. This is the case, for example, of *JUN:FOS*, which induces anchorage-independent growth^42^ and *SPI1*, a known oncogene that increases the speed of replication^43^, which are deactivated in colon (COAD), uterine (UCEC), bladder (BLCA), lung (LUAD and LUSC) and prostate adenocarcinoma (PRAD) cancers, while are activated in the rest of cancers, suggesting the existence of different growing strategies in these two groups of cancers.

On the other hand, a few TFs systematically display a significant decrease in their activities. For example, two TFs with a largely unexplored role in human tumorigenesis, *MEF2A* and *MEF2C,* significantly reduce their activity in uterine (UCED), bladder (BLCA) and lung (LUSC) cancers. Supporting this observation, a significant down-regulation of *MEF2A* and *MEF2C* TFs was recently described in glioblastoma multiforme^44^. Actually, studies suggested that *MEF2C* is as target of miR-223^45^, an miRNA known to promote the invasion of breast cancer cells^46^.

Finally, other TFs display activations or deactivations shared by a few cancers and some of them present cancer-specific activities (See Fig. 1). Thus, *FOS* is activated in LIHC and THCA, or *FOSL1* and *FOSL2* are activated in KIRP, KIRC and THCA. Genes of the *FOS* family have been implicated as regulators of cell proliferation, differentiation, and transformation and are involved in many tumorigenic processes. Also *REST* gene, a transcriptional repressor that represses neuronal genes in non-neuronal tissues, is significantly activated in LIHC but significantly deactivated in COAD, maybe due to its dual role as a tumour suppressor and oncogene^47^.

Regarding TFs specific of cancers, *JUNB*, with a known role in liver regeneration^48^, but previously associated to different lymphomas such as Hodgkin^49^ or cutaneous T-cell^50^, seems to be also relevant in LIHC tumorigenesis. Thyroid carcinoma (THCA) presents a quite atypical pattern of TF activation. While it lacks some ubiquitous TFs, such as *YY1, SP2, ZBTB33* or *NR2C2*, it presents significant activations in *HNF4A, RXRA* and *RXR::RAR_DR5*. Although *HFN4A* has traditionally been linked to diabetes, it has recently been suggested that this TF could be the link between ulcerative colitis and colorectal cancer^51^ and it has even be proposed as a biomarker of this cancer^52^ (colorectal cancer is not among the cancers included in this study). *RXR* and *RAR* are retinoid receptors that regulate cell growth and survival^53^, which have been proposed as cancer therapeutic targets^54^.

Cancers can be grouped in three main clusters according to their TF activity patterns. One of them is composed of uterine (UCEC), bladder (BLCA), lung (LUAD and LUSC) and prostate adenocarcinoma (PRAD) cancers. Another, more dispersed cluster is composed of breast (BRCA), kidney papillary cell (KIRP) head and neck squamous cell (HNSC) and liver (LIHC) cancers. Although showing a regulatory behaviour quite different among them, kidney clear cell (KIRC) and head and neck thyroid (THCA) carcinomas cluster together. Colon adenocarcinoma (COAD) maps closer to the first cluster but seems to be an outlier in terms of TF activity pattern.

Some cancers, however, display atypical activity patterns of activity for several TFs. For example, COAD shows a specific significant activity decrease of *REST, CTCF* (a known chromatin insulator protein that may play a central role in mediating long-range chromatin interactions, whose deregulation has an increasingly important role in the epigenetic imbalance in cancer^55^), *EBF1* (identified as a tumour suppressor^56^) and *TCF12*. These regulatory differences might account, at least partially, for the different clinical behaviours of the distinct cancers analysed.

With respect to tissue of origin, both lung cancers, LUAD and LUSC, present quite similar TF activity profiles. Contrarily, kidney cancers KIRC and KIRP display remarkably different TF activity profiles. Interestingly, *FOSL1* and *FOSL2* TFs are specifically active almost uniquely in both cancers, while *FOXA1* is significantly inactive. In particular, *FOXA1,* a TF involved in the differentiation of the pancreas and liver, is known to be expressed in breast cancer^57^ and others. Its remarkable down-activation in the two cancers originated in kidney could be part of the tumorigenesis in this organ.

It is worth noticing that, as previously mentioned, the expression level of TFs in the tissues according to The Protein Human Atlas database^58^ is uncorrelated with the corresponding activity detected from the expression of the corresponding targets (Supplementary Figure 1). This reinforces the usefulness of this approach, given that the direct observation of TF expression would have not rendered detectable changes in their behaviours.

Supplementary Table 1 contains the complete list of p-values obtained for all the TFs in all the cancers studied.

### Changes in TF activity across cancer stages

**Figure 2:**
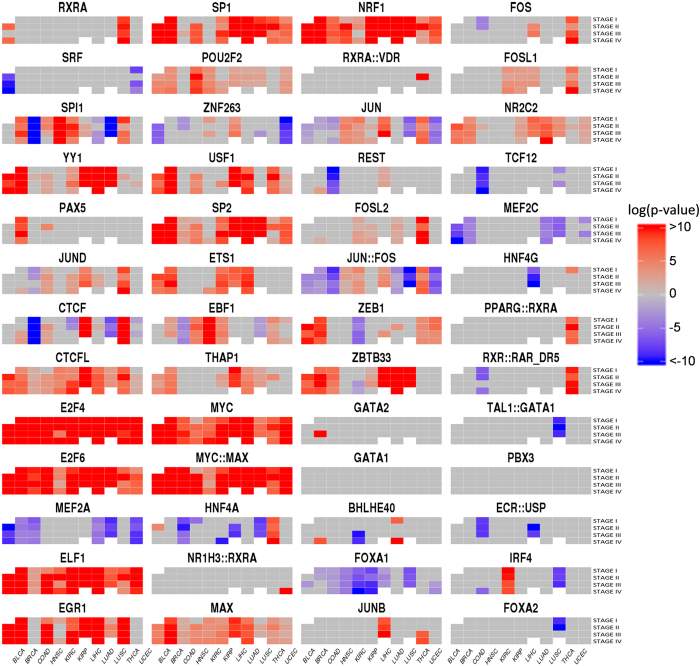
Change of activity in all TF included in this study across cancer stages in the different cancers studied. Each panel corresponds to a single TF, with stages in rows and cancers in columns. The colour scale in the figure ranges from red, indicating a significant increased activity of the TF in the stage of the cancer with respect to the corresponding normal tissue, according to the TFTEA, to blue, indicating a significantly decreased activity. The colour scale represents −log10 (adjusted p-value). Cells in grey indicate that no significant change in activity was detected. Cells in white correspond to stages in cancers with very few individuals (see Table 1) in which the analysis could not be carried out.

The availability of clinical information, such as cancer stage allowed the stratification of cancer samples into their different stages. In any cancer type, the samples in any stage were compared to the corresponding normal samples. Figure 2 summarizes the changes in the activity status of TFs with respect to the normal situation in all the stages of the cancers analysed. Although the profiles of TF activity observed in this analysis are overall similar to the results produced by the comparison of cancer versus normal gene expressions, this analysis renders a more detailed picture of the changes in TF activity across stages in the different cancers. In fact, all the TFs present some activity change in some stages, even if this activity was not detected in the general cancer-control comparison. Thus, for example, *RXRA*, that was significantly active only in THCA in the cancer – control comparison, here presents a complex activation pattern across stages in BLCA as well. Other TFs, for which no significant change in the activity was previously found comparing cancer versus normal tissues, present however significant stage-specific activations, such as *PPARG::RXRA* activated in THCA, *TAL::GATA1*, down-activated in LUSC, *ECR::USP*, down-activated in several stages of LIHC and COAD.

Supplementary Table 1 contains the complete list of p-values obtained for all the comparisons of TF activities across stages in all the cancers studied.

### TF activity and survival

**Figure 3:**
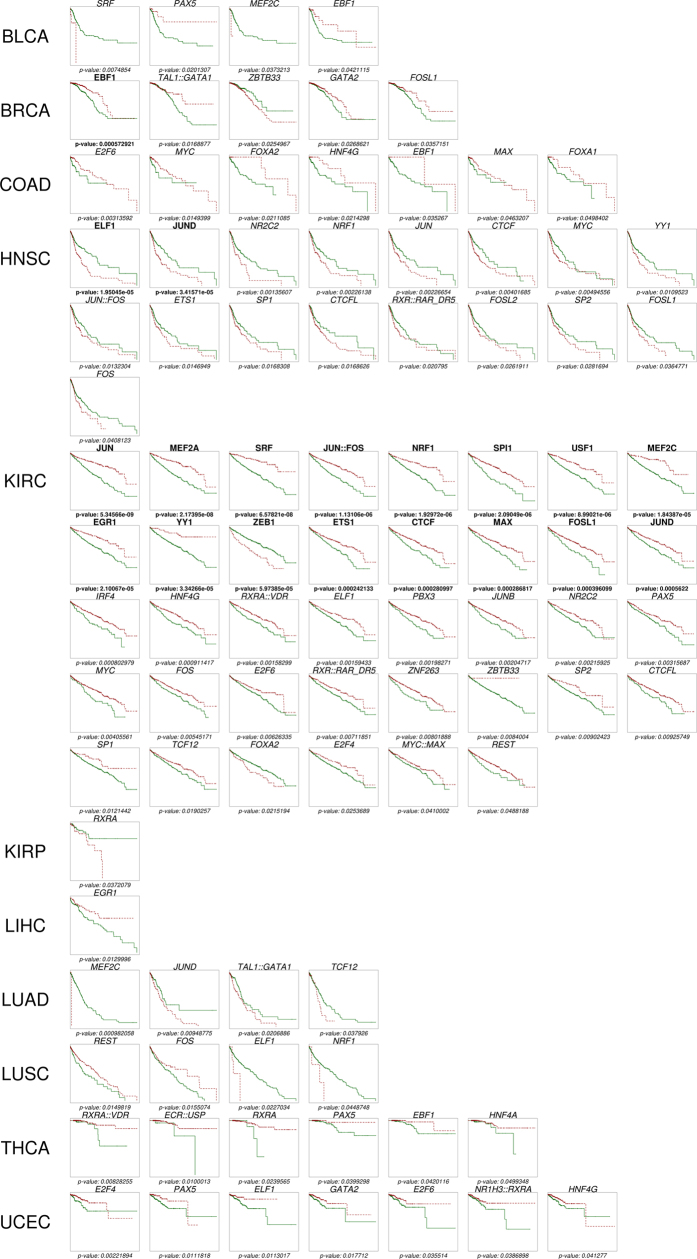
K-M plots representing TF activities significantly associated to patient survival in all the cancers analysed. TFs in bold present a significant association with adjusted p-values < 0.05 and TFs in italics have nominal p-values < 0.05.

**Figure 4:**
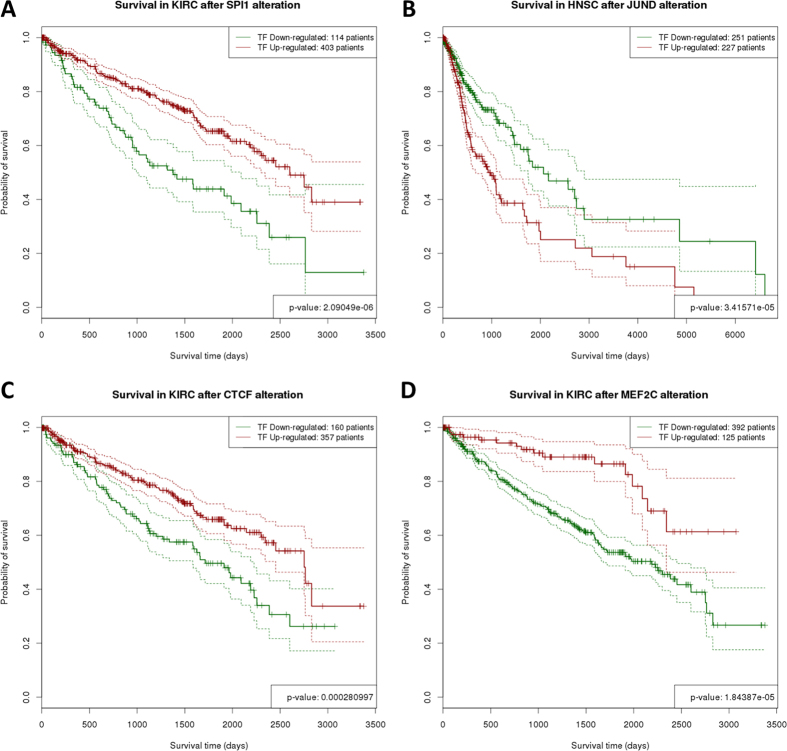
K-M plots representing TF activities significantly associated to patient survival. Survival curves are represented as solid lines and their corresponding confidence intervals as dotted lines. (**A**) High activity (green curve) of *SPI1* in KIRC is significantly associated to patient survival (FDR-adjusted p-value = 2.09 × 10^−6^); (**B**) High activity of *JUND* in HNSC is significantly associated to bad prognostic (FDR-adjusted p-value = 3.42 × 10^−5^); (**C**) Low activity of *CTCF* in KIRC is significantly associated to bad prognostic (FDR-adjusted p-value = 2.81 × 10^−4^); (**D**) Low activity of *MEF2C* in KIRC is significantly associated to bad prognostic (FDR-adjusted p-value = 1.84 × 10^−5^).

The availability of survival data for the cancers analysed (Table 1) allows testing hypotheses on the contribution of distinct TF activities in the cancers to the disease outcome by validating their association with patient’s survival. Since TFTEA can be applied in a personalized way to individual samples (see Methods) it is possible to know what TFs are active in any particular sample. Therefore, it is straightforward to test the relationship between TF activity and patient’s survival using Kaplan-Meier (K-M) curves^59^. Figure 3 summarises the K-M plots representing TF activities significantly associated to patient survival (See detailed plots in Supplementary Figure 2 and Supplementary Table 2). As expected, more significant results were found in the cancers with more detailed data on survival, which are KIRC, BRCA and HNSC (See Table 1). A total of 19 TFs presented a strong significant (adjusted p-values < 0.05) association between its activity and patient survival in BRCA, HNSC and KIRC. The number of TFs in the figure increases to 92 if we consider significant nominal p-values, and cover all the cancers (Fig. 3, Table 2 and Supplementary Table 3). Some of the TFs highly associated to survival have been detected in the study of TF activity across cancers. Examples in KIRC are: *JUN:FOS*, known to be correlated to KIRK survival^28^ and probably related to metastatic proliferation^42^, *SPI1*, whose activation has been linked to survival in gastric cancer^41^ in agreement with our observation (Fig. 4A), *JUND*, whose upregulation is significantly related to bad prognostic (Fig. 4B) and it has been described that can collaborate with NF-κB to increase antiapoptotic gene expression^60^ and also *NRF1, EGR1, ETS1, ZEB1, MAX* and *FOSL1*. Previous studies of TF activity in KIRK reveal a number of them significantly correlated to survival^28^. Among the TFs that overlap with this study, *FOS, JUN::FOS, REST* and *TCF12* are found to be significantly related to survival, while *GATA1* did not reached the significance threshold. In HNSC, *JUND* and *ELF1* are differentially activated between the cancer and the normal tissue and also significantly associates to survival. In agreement with our results (Fig. 3, Table 2 and Supplementary Table 3), it has already been described that TAL1 was significantly correlated with breast cancer survival^27^.

Interestingly, there are TFs whose activity does not change significantly between the cancer and the normal tissue (see Fig. 1), but play, however, an unquestionable role in survival. These are the cases of *EBF1* in BRCA, which is a tumour suppressor^56^ and its lower activity is associated to higher mortality or *CTCF* in KIRC^61^ (see Fig. 4C). The case of *MEF2A* and *MEF2C* is similar: lower activity is significantly associated to worst prognostic (Fig. 4D), which is supported by the fact that its inhibition by miR-223 promotes the invasion of breast cancer cells^45^,^46^. These observations suggest that TFs whose activity is not especially relevant in the cancer tissue are however important in the determination of the prognostic of the patients and might be interesting therapeutic targets.

A complete list of p-values obtained for all the relationships of TF activities with survival in all the cancers studied can be found in Supplementary Table 3.

### Combined contribution of TF activity to survival and the impact of tumour purity

Despite the obvious impact of individual TF activities in patient survival, it is clear that such a complex phenotype cannot be the effect of unique TF activities but rather will require of the interplay of several TFs. In order to capture at least part of the complexity of this interplay of TF activities that will ultimately affect patient survival we used a multivariate procedure. Conceptually, increasing levels of TF activity, as reported by TFTEA, accounts for higher expressions of increasingly larger number of targets of the TF. This continuous variable is modelled for multiple TFs with respect to the event of death in the patients by applying Cox multiple regression models and using a stepwise algorithm (see details in Methods).

Recently, the importance that the non-cancerous components of the tumour (that include immune cells, fibroblasts, endothelial cells and normal epithelial cells) may have in cancer biology has been described^62^. Actually, it has been shown in some circumstances, the presence of these cells may alter the results of genomic analyses, including survival^62^. In order to check potential alterations in the TF activities inferred from the datasets studied here, we have compared the mean tumour purities with the outcome of the application of the method to see if there was any relationship between the mean purity of the cancer and the potential sensibility of the method in detecting TF activations (measured as the number of significant TF activity changes detected). Supplementary Figure 3 clearly shows that there is no observable trend between both variables, which strongly suggests that the application of the method to the analysed datasets was not significantly affected by the mean cancer purity. However, the fact that TF activity estimations are not affected by tumour purity does not discard a possible confounding effect of the non-cancerous component of the cancer in this measurement. To study this potential confounding effect, the value of tumour purity was introduced in the Cox model as another variable.

**Figure 5:**
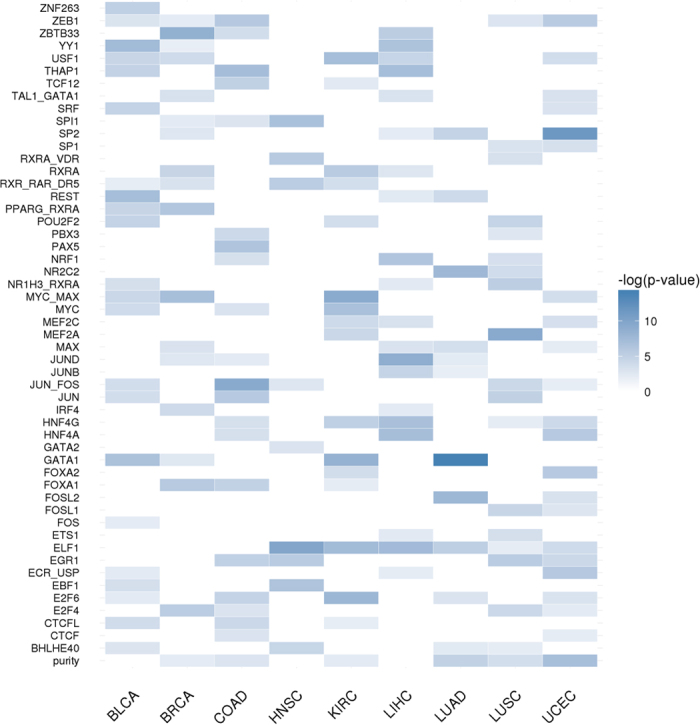
Combinations of TFs significantly associated to patient survival in the different cancers when a Cox model is applied. Cancers are represented in columns and TFs in rows. For each cancer, several TFs and sometimes tumour purity were included in a cox model. The colour intensity is related to the significance of this association (p-value).

The results obtained, listed in Table 2 and summarized in Fig. 5, clearly demonstrate a significant connection between multiple TF activity and patient survival for all the cancers analysed. The influence of TF activity in bad prognostic of the tumour seems to be a complex process in which different TF act cooperatively to activate (or deactivate) a large number of cell programmes that initiate and/or progress distinct cancer hallmarks^39^ in the tumour cells. The results depict a relevant contribution of tumour purity to patient survival in three out of the eleven cancers analysed (both lung cancers LUAD and LUSC, and the endometrial carcinoma, UCEC). Although non-significant, tumour purity is still selected by the Cox model in another three cancers (BRCA, COAD and KIRK), where probably plays a more marginal role.

### Potential limitations of the method

It must be taken into account that the information on TFBSs might contain a non-negligible number of false positives along with the true TFBSs in the real TF targets. This reduces the power of detection of the method given that, if a TF is activating their real targets, and a number of genes with random activity are considered to be part of the gene set of the TF, the complete gene set will show an activity lower than the actual activity. In addition, only a relatively low number of TFs are well characterized in terms of target genes they activate.

Another potential problem that can reduce the sensitivity of the analysis is the fact that many TFs need of a combination of factors to properly carry out transcription.

Finally, only KIRC (and to a lesser extent HNSC) had enough data on deceased patients to carry out robust survival analysis. Supplementary Figure 4 clearly depicts this trend. The survival is best detected in KIRC and HNSC because a higher number of deceased patients is present in the dataset analysed (which also explains the high values of these cancers, unrelated with cancer purity, observed in Supplementary Figure 3).

In spite of these problems that reduce the potential of discovery of the proposed methodology in the current datasets, we have discovered a reasonable amount of significant associations of TF activity with cancer progression and with survival. Despite limited, the results obtained, which can be considered the “tip of the iceberg”, and are quite encouraging.

## Conclusions

The availability of survival and other relevant clinical data makes the analysis of pan-cancer *Big Data* repositories (ICGC and others) especially compelling, given that new unexpected associations of genomic data to relevant clinical outcomes can be found. Despite the relevance of regulation in cancer this seems to be the first pan-cancer analysis carried out to date. We have applied the TFTEA, a simple but robust methodology, to detect significant changes in the TF activity status when two groups of individuals are compared. In addition, the methodology also provides TF activity values per individual. This interesting property allows detecting TF-mediated deregulations specific for individuals, thus opening the door to possible personalized therapeutic interventions.

Regardless of the expectable reduction in the detection power that the current definitions of TF target gene sets could produce in methods that rely on this knowledge, the TFTEA still discovered a considerable number of significant associations between TF activity and the acquisition of cancer, the progression of cancer across stages or the survival of patients. Actually, many of the altered activities in TFs found were described in the literature either directly as causal alterations or, at least, linked to cancer, providing an extra support to the validity of the proposed methodology. Moreover, statistical modelling allowed detecting an important role of tumour purity in survival. This suggests that, in some cases, the TF activity related to survival detected by the test could be due in part to other non-cancerous components of the tumour (probably immune cells, but also fibroblasts, endothelial cells and normal epithelial cells).

Actually, the findings of this work constitute most probably an underestimation of the total number of TFs linked to bad prognostic, due to the lack of enough survival data among the samples that precluded obtaining significant results for more TFs. This suggests that more detailed results would be obtained by the application of TFTEA to patient cohorts with richer clinical annotations.

## Methods

### Cancer samples used

Eleven cancer types amounting to 5607 samples (Table 1) were selected on the basis of the simultaneous availability of paired samples (transcriptome analysis from both tumour sample and adjacent healthy tissue) and clinical data (tumour stage and survival). Raw read count data files were downloaded from the ICGC data portal^63^ and clinical data were downloaded from the TCGA data portal^64^ using sample IDs to cross-reference patient’s data.

### Gene expression data processing

The trimmed mean of M-values normalization (TMM)^65^ was the method of choice and was applied using the *edgeR* package^66^, using the default parameters. The differential expression analysis between cases and controls was carried out using the *limma* package^67^,^68^. Firstly, the *voom* function^69^ is applied to weight and transform TMM normalized values to make them suitable for lineal model analysis. Then, the *lmFit* function is used to adjust a lineal model and an empirical Bayes method is used to estimate differential expression values.

The Human Protein Atlas^58^,^70^ was used as a reference for the gene expression levels of TFs in normal tissues.

### Transcription factors used in the study

We have used a total of 52 TF available in ENSEMBL (GRCh38.p3), which are: *RXRA, SRF, SPI1, YY1, PAX5, JUND, CTCF, CTCFL, E2F4, E2F6, MEF2A, ELF1, EGR1, SP1, POU2F2, ZNF263, USF1, SP2, ETS1, EBF1, THAP1, MYC, MYC::MAX, HNF4A, NR1H3::RXRA, MAX, NRF1, RXRA::VDR, JUN, REST, FOSL2, JUN::FOS, ZEB1, ZBTB33, GATA2, GATA1, BHLHE40, FOXA1, JUNB, FOS, FOSL1, NR2C2, TCF12, MEF2C, HNF4G, PPARG::RXRA, RXR::RAR_DR5, TAL1::GATA1, PBX3, ECR::USP, IRF4* and *FOXA2.*

Any of these TFs activates a set of genes. Here we consider that a gene can potentially be activated by a TF if it includes possible binding sites for it, located between 5000 bp upstream from the most external transcription origin and the first exon. TFBSs have been mapped by Ensembl^71^,^72^ along the genome. Briefly, for any TF which has both a ChIP-seq data and a JASPAR^73^ publicly available position weight matrix (PWM), Ensembl annotates the position of putative TFBSs within the ChIP-seq peaks (details can be found in specific Ensembl web pages^74^). This information is accessible in a more efficient way in different publicly available resources, such as CellBase^75^, whose web services^76^ were used here. Supplementary Table 4 shows the list of target genes for each TF.

### Estimation of significant transcription factor activities in a cancer datasets

Since direct inference of TF activity from its own gene expression level is problematic, in this work we indirectly infer its activity from the collective activity of their gene targets. The method used here is an analysis of Gene Set Enrichment (GSE) that we call TF Target Enrichment Analysis (TFTEA). In this approach, each TF has an associated gene sets composed by the all their target genes (those containing a TFBS for the TF located between 5000 bp upstream from the transcription origin and the first exon of the gene).

Like other GSE methods, the TFTEA algorithm detects asymmetrical distributions of targets of TFs in the top (or the bottom) of a list of ranked genes. When two conditions are compared and the genes are ranked by differential expression (or fold change or any other related parameter), the detection of a significant accumulation of targets of a given TF in the upper (or lower) part of the ranked list indicates that such TF has significantly increased (or decreased) its activity in one of the conditions with respect to the other one. Here, differential expression in calculated by means of a *limma* test^67^, and the results of the statistic are used to define the ranked list of genes. A logistic regression is the most efficient methodology used for the detection of gene sets with a significant systematic over- or under-expressed^77^,^78^. Specifically, the association of a gene set composed by the targets of a specific TF to high or low values of the ranked list of genes is tested by means of the value of the slope of the logistic regression. The null hypothesis, slope = 0, is tested against the alternative slope ≠ 0 based on the maximum likelihood parameter estimates and the Wald test. For testing slope = 0, the Wald statistic can be shown to follow a chi-square distribution with one degree of freedom and the p-value is calculated assuming this null distribution^77^,^78^.

**Figure 6:**
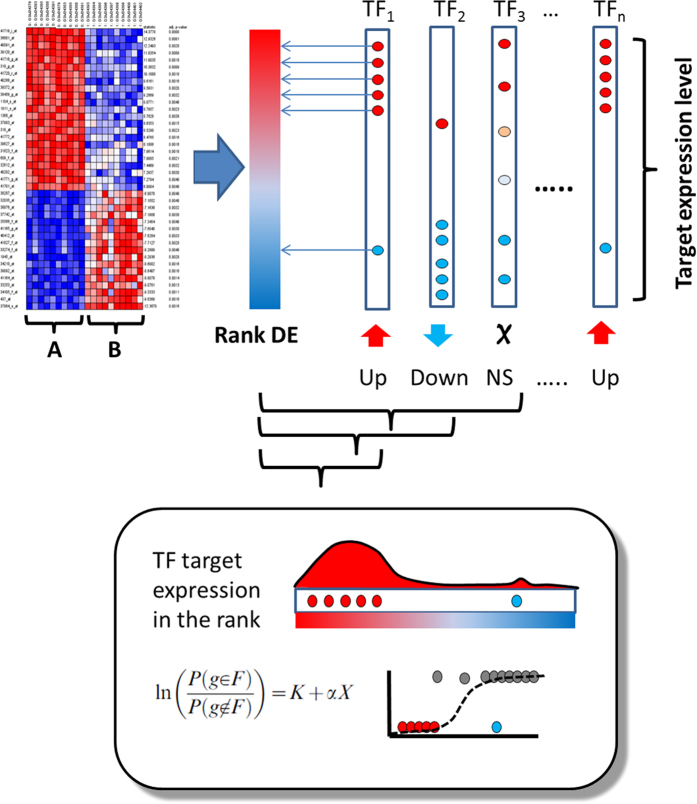
Schema of the TFTEA method to obtain TFs differentially activated between two conditions compared. The method uses gene expression values and compares two conditions (**A** and **B**) by means of any test to obtain a rank of differentially expressed genes (Rank DE) based on the statistic. Then, for each TF, a logistic regression^78^ is applied to discover associations of the TF targets to high or low values of the rank (lower panel). Thus, targets of TF_1_ show a clear association to high values of the statistic, meaning that have significantly higher expression in condition (**A**) than in condition ( ), which demonstrated the differential activity of TF_1_. TF_2_ is the opposite case, in which the TF is significantly less active in (**B**) than in (**A**). TF_3_ have their targets active or inactive in both conditions, meaning that these activities are not a collective property and consequently are not due to TF_3_, but maybe to other regulators.

Since many TFs were tested with the logistic regression across the eleven cancers, multiple testing effects need to be corrected- Here we have used the popular FDR method^79^ for this purpose. Figure 6 schematizes the application of the method.

### Estimation of personalized transcription factor activities per individual

**Figure 7:**
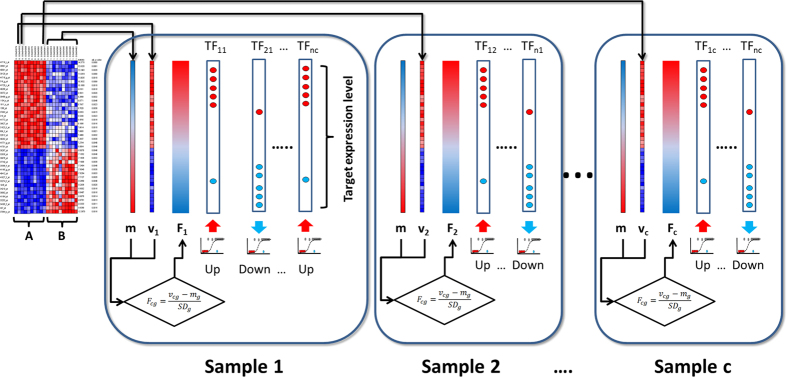
Schema of the TFTEA method to obtain personalized values of survival. The method uses gene expression values and compares two conditions (**A** and **B**). However, in this case all the samples in the (**B**) condition are used to produce an average expression value for any of the genes in this condition (m vector). Then, each sample in the (**A**) condition (v_c_) can be compared to the average expression in the (**B**) condition and a rank of fold change (F_cg_) is generated for each sample. Then, this ranking is used in the same way that the rank of differential expression was used in Fig. 6 to find differentially activated TFs in samples from the condition (**A**) with respect to the average (**B**) condition. Is samples are paired the F_cg_ value can be derived from the direct comparison between them.

We also used TFTEA to relate individual survival events to TF activity. Since this method requires of a ranked list of genes, each normalized patient sample needs to be compared to a reference. This reference value was obtained as the average normalized expression value across all the normal samples (see Fig. 7). For each gene (g) of any cancer sample (c), its expression value (*v*_*cg*_) is compared with the corresponding average expression value for this gene in all the healthy samples (*m*_*g*_) and the resulting value is divided by the standard deviation of the gene expression value in the healthy samples. This comparison provides for each cancer sample a value per gene that can be interpreted as a fold change (*F*_*cg*_) with respect to the average healthy expression:


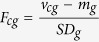


*F*_*cg*_ values can thus be used to rank genes in a unique individual according to its relative expression with respect to the average expression values of their counterparts in a normal tissue.

Once a list of genes ranked by decreasing *F*_*cg*_ values is obtained for each patient, the TFTEA can be applied in a personalized manner to detect those TFs significantly activated (or deactivated) in each particular individual.

If samples are paired, the *F*_*cg*_ rank can be generated by direct comparison or each pair.

### Correlation between transcription factor activity and patient survival

Kaplan-Meier (K-M) curves^59^ are used to relate TF activity to survival in the different cancers. The value of the statistic of each TF in each individual was used as a proxy of its activity. Only FT-cancer pairs with 10 events (deaths) or more were taken into account and the multiple testing adjustments were made taking into account only the pairs analysed. Calculations were carried out using the function *survdiff* from the *survival* R package^80^.

Cox regression analysis^81^ is used to relate combined TF activity to survival in the different cancers. Since tumour purity has been involved in survival^62^, we used individual tumour purity values as an extra variable in the cox regression. Calculations were carried out using the function *coxph* from the *survival* R package^80^. A stepwise algorithm, implemented in the *step* function from the R package *stats*^82^,^83^, is used to add or remove TFs or the tumour purity value according to the significance of their contributions to explain survival in the multiple regression model. In this way a final list of variables (TFs and cancer purity) whose combination is significantly related to survival is obtained. The *step* package uses Akaike Information Criterion (AIC) to select the best model by iteratively adding and removing variables.

### Tumour purity estimation

There are different approaches to estimate tumour purity values, such as ESTIMATE, based on gene expression profiles of known immune stromal genes^84^; ABSOLUTE, based on somatic copy-number data^85^; LUMP (leukocytes unmethylation for purity), based on averages of non-methylated immune-specific CpG sites^62^.

Consensus measurement of purity estimations (CPE) is the median purity level after normalizing levels from all methods to give them equal means and s.d.’s (75.3 ± 18.9%).

Here, the per individual purity values provided in Supplementary Data 1 in the Aran’s paper^62^ are used to study the contribution of tumour purity to survival in the Cox regression.

### Code availability

The code is open and available at: https://github.com/babelomics/TFTEA.

## Additional Information

**How to cite this article**: Falco, M. M. *et al*. The pan-cancer pathological regulatory landscape. *Sci. Rep.* **6**, 39709; doi: 10.1038/srep39709 (2016).

**Publisher's note:** Springer Nature remains neutral with regard to jurisdictional claims in published maps and institutional affiliations.

## Supplementary Material

Supplementary Table 1

Supplementary Table 3

Supplementary Table 4

Supplementary Figures and Table 2

## Figures and Tables

**Table 1:** Cancer samples available for any cancer type selected.

Cancer type	Tumour	Normal	Stage I	Stage II	Stage III	Stage IV	Alive	Deceased
*Bladder Urothelial Cancer* **[BLCA]**	294	17	1	95	99	98	221	73
*Breast Cancer* **[BRCA]**	1039	113	177	591	237	17	937	98
*Colon Adenocarcinoma* **[COAD]**	428	41	73	168	120	58	374	53
*Head and Neck Squamous Cell Carcinoma* **[HNSC]**	480	42	26	74	72	245	320	158
*Kidney Renal Clear Cell Carcinoma* **[KIRC]**	517	72	256	56	125	81	358	159
*Kidney Renal Papillary Cell Carcinoma* **[KIRP]**	222	32	138	16	43	13	199	23
*Liver Hepatocellular carcinoma* **[LIHC]**	294	48	132	66	71	5	222	72
*Lung Adenocarcinoma* **[LUAD]**	473	55	255	116	81	24	355	118
*Lung Squamous Cell Carcinoma* **[LUSC]**	426	45	217	128	75	6	290	136
*Head and Neck Thyroid Carcinoma* **[THCA]**	500	58	282	54	110	52	481	14
*Uterine Corpus Endometrial Carcinoma* **[UCEC]**	508	23	318	49	114	27	464	43

**Table 2:** TFs significantly associated to survival.

Cancer type	Variables (TFs and PURITY) selected by the Cox model	Total	TFs with individual effect on survival (K-M)	Total
**BLCA**	**SRF, YY1, CTCFL, POU2F2, ZNF263, USF1, EBF1, THAP1, MYC, MYC::MAX, NR1H3::RXRA, JUN, REST, JUN::FOS, GATA1, PPARG::RXRA,** *E2F6, ZEB1, BHLHE40, FOS, RXR::RAR_DR5, ECR::USP*	22	*EBF1, MEF2C, PAX5, SRF*	4
**BRCA**	**RXRA, E2F4, USF1, MYC::MAX, MAX, ZBTB33, FOXA1, PPARG::RXRA, RXR::RAR_DR5, TAL1::GATA1, IRF4**, *SPI1, YY1, JUND, SP2, ZEB1, GATA1, PURITY*	18	*EBF1, FOSL1, GATA2, TAL1::GATA1, ZBTB33*	5
**COAD**	**PAX5, CTCFL, E2F6, EGR1, THAP1, MYC, HNF4A, NRF1, JUN, JUN::FOS, ZEB1, ZBTB33, FOXA1, TCF12, HNF4G, PBX3**, *SPI1, JUND, CTCF, E2F4, PURITY*	21	*MYC, E2F6, EBF1, FOXA1, FOXA2, HNF4G, MAX*	7
**HNSC**	**SPI1, ELF1, EGR1, EBF1, RXRA::VDR, BHLHE40, RXR::RAR_DR5**, *JUN::FOS, GATA2*	9	**ELF1, JUND,** *FOS, JUN, MYC, CTCF, CTCFL, ETS1, FOSL1, FOSL2, JUN::FOS, NRF1, RXR::RAR_DR5, SP1, SP2, NR2C2, YY1*	17
**KIRC**	**RXRA, E2F6, MEF2A, ELF1, POU2F2, USF1, MYC, MYC::MAX, GATA1, MEF2C, HNF4G, RXR::RAR_DR5, FOXA2**, *CTCFL, FOXA1, TCF12, PURITY*	17	**EGR1, JUN::FOS, JUN, MEF2A, NRF1, SPI1, SRF, USF1, YY1, ZEB1**, *FOS, MYC, CTCF, CTCFL, E2F4, E2F6, ELF1, ETS1, FOSL1, FOXA2, HNF4G, IRF4, JUNB, JUND, MAX, MYC::MAX, REST, PAX5, PBX3, RXR::RAR_DR5, RXRA::VDR, SP1, SP2, TCF12, NR2C2, ZBTB33, ZNF263*	38
**KIRP**	—	0	*RXRA*	1
**LIHC**	**YY1, JUND, ELF1, USF1, THAP1, HNF4A, MAX, NRF1, ZBTB33, JUNB, MEF2C, HNF4G, TAL1::GATA1**, *RXRA, SP2, ETS1, NR1H3::RXRA, REST, ECR::USP, IRF4*	20	*EGR1*	1
**LUAD**	**PURITY, ELF1, SP2, MAX, REST, FOSL2, GATA1, NR2C2**, *JUND, E2F6, BHLHE40, JUNB*	12	*JUND, MEF2C, TAL1::GATA1, TCF12*	4
**LUSC**	**PURITY, E2F4, MEF2A, EGR1, SP1, POU2F2, ETS1, NR1H3::RXRA, NRF1, RXRA::VDR, JUN, JUN::FOS, FOSL1, NR2C2**, *ELF1, ZEB1, BHLHE40, HNF4G, PBX3*	19	*FOS, ELF1, NRF1, REST*	4
**THCA**	—	0	*EBF1, ECR::USP, HNF4A, PAX5, RXRA, RXRA::VDR*	6
**UCEC**	**PURITY, SRF, E2F6, ELF1, EGR1, SP1, USF1, SP2, MYC::MAX, HNF4A, FOSL2, ZEB1, MEF2C, HNF4G, TAL1::GATA1, ECR::USP, FOXA2**, *CTCF, E2F4, MAX, JUN::FOS, FOSL1*	22	*E2F4, E2F6, ELF1, GATA2, HNF4G, PAX5*	6

The first column denoted the cancer type analysed. The second column contains the variables included in the Cox multiple regression model, which can be TFs and tumour purity. The third column contains the total number of TFs included in the Cox model. The fourth column shows TFs that show a significant association to survival by themselves. The fifth column contains the number of TFs significant in the K-M analysis. TFs in bold are significant with an adjusted p-value < 0.05. TFs in grey and in italic are significant with a nominal p-value < 0.05.
