# Supplementary material for: The pan-cancer pathological regulatory landscape
Source: Sci Rep. 2016 Dec 21;6:39709. doi: 10.1038/srep39709 (PMC5175166; doi:10.1038/srep39709)

The pan-cancer pathological regulatory landscape

Matias M. Falco, Marta Bleda, José Carbonell-Caballero, Joaquín Dopazo

Supplementary Material

Supplementary Table 2.

List of p-values of the Kaplan-Meyer test that relates TF with survival, along with hazard ratios and the number of patients with high and low TF activity.

Cancer	Transcription Factor	Kaplan-Meyer p-value	Hazard ratio (confidence interval)	Patients high TF activity	Patients low TF activity
BLCA	EBF1	0.0421115	0.475 (0.228-0.99)	61	233
BLCA	MEF2C	0.0373213	6.508 (0.853-49.628)	9	285
BLCA	PAX5	0.0201307	0.277 (0.087-0.883)	35	259
BLCA	SRF	0.0074854	4.375 (1.34-14.281)	13	281
BRCA	EBF1	0.000572921	0.467 (0.299-0.728)	443	592
BRCA	FOSL1	0.0357151	0.582 (0.349-0.971)	270	765
BRCA	GATA2	0.0268621	0.639 (0.428-0.953)	540	495
BRCA	TAL1_GATA1	0.0168877	0.578 (0.367-0.911)	383	652
BRCA	ZBTB33	0.0254967	1.619 (1.056-2.481)	533	502
COAD	MYC	0.0149399	0.491 (0.275-0.88)	193	233
COAD	E2F6	0.00313592	0.415 (0.228-0.756)	196	230
COAD	EBF1	0.035267	0.154 (0.021-1.129)	24	402
COAD	FOXA1	0.0498402	0.467 (0.215-1.016)	65	361
COAD	FOXA2	0.0211085	0.216 (0.052-0.9)	42	384
COAD	HNF4G	0.0214298	0.351 (0.138-0.891)	61	365
COAD	MAX	0.0463207	0.547 (0.3-0.997)	149	277
HNSC	FOS	0.0408123	1.434 (1.013-2.031)	126	352
HNSC	JUN	0.00226654	1.636 (1.189-2.252)	232	246
HNSC	MYC	0.00494556	1.575 (1.144-2.167)	250	228
HNSC	CTCF	0.00401685	1.626 (1.164-2.273)	268	210
HNSC	CTCFL	0.0168626	1.513 (1.075-2.129)	293	185
HNSC	ELF1	1.95045e-05	1.987 (1.442-2.74)	233	245
HNSC	ETS1	0.0146949	1.479 (1.078-2.031)	160	318
HNSC	FOSL1	0.0364771	1.41 (1.02-1.949)	155	323
HNSC	FOSL2	0.0261911	1.435 (1.042-1.977)	162	316
HNSC	JUN_FOS	0.0132304	1.482 (1.084-2.027)	220	258
HNSC	JUND	3.41571e-05	1.946 (1.412-2.682)	227	251
HNSC	NRF1	0.00226138	1.624 (1.186-2.223)	208	270
HNSC	RXR_RAR_DR5	0.020795	1.448 (1.056-1.986)	183	295
HNSC	SP1	0.0168308	1.464 (1.069-2.005)	203	275
HNSC	SP2	0.0281694	1.418 (1.037-1.941)	196	282
HNSC	NR2C2	0.00135607	1.741 (1.235-2.455)	117	361
HNSC	YY1	0.0109523	1.522 (1.099-2.108)	122	356
KIRC	FOS	0.00545171	0.645 (0.472-0.881)	305	212
KIRC	JUN	5.34566e-09	0.382 (0.273-0.534)	263	254
KIRC	MYC	0.00405561	0.57 (0.386-0.84)	442	75
KIRC	CTCF	0.000280997	0.563 (0.411-0.771)	357	160
KIRC	CTCFL	0.00925749	0.638 (0.453-0.897)	400	117
KIRC	E2F4	0.0253689	0.638 (0.428-0.949)	125	392

KIRC	E2F6	0.00626335	0.616 (0.434-0.875)	190	327
KIRC	EGR1	2.10067e-05	0.444 (0.302-0.652)	178	339
KIRC	ELF1	0.00159433	0.584 (0.417-0.819)	205	312
KIRC	ETS1	0.000242133	0.561 (0.41-0.767)	330	187
KIRC	FOSL1	0.000396099	0.54 (0.382-0.764)	416	101
KIRC	FOXA2	0.0215194	1.61 (1.069-2.424)	71	446
KIRC	HNF4G	0.000911417	0.594 (0.435-0.811)	332	185
KIRC	IRF4	0.000802979	0.559 (0.396-0.789)	404	113
KIRC	JUN_FOS	1.13106e-06	0.467 (0.342-0.64)	311	206
KIRC	JUNB	0.00204717	0.601 (0.433-0.834)	394	123
KIRC	JUND	0.0005622	0.58 (0.424-0.794)	296	221
KIRC	MAX	0.000286817	0.562 (0.41-0.771)	357	160
KIRC	MEF2A	2.17395e-08	0.379 (0.266-0.54)	239	278
KIRC	MEF2C	1.84387e-05	0.341 (0.203-0.571)	125	392
KIRC	MYC_MAX	0.0410002	0.724 (0.53-0.988)	290	227
KIRC	NRF1	1.92972e-06	0.4 (0.271-0.591)	174	343
KIRC	REST	0.0488188	0.668 (0.446-1.001)	442	75
KIRC	PAX5	0.00315687	0.626 (0.458-0.857)	277	240
KIRC	PBX3	0.00198271	0.59 (0.421-0.827)	390	127
KIRC	SPI1	2.09049e-06	0.46 (0.331-0.639)	403	114
KIRC	RXR_RAR_DR5	0.00711851	0.643 (0.465-0.889)	242	275
KIRC	RXRA_VDR	0.00158299	0.609 (0.446-0.831)	309	208
KIRC	SP1	0.0121442	0.531 (0.321-0.878)	81	436
KIRC	SP2	0.00902423	0.575 (0.377-0.876)	117	400
KIRC	SRF	6.57821e-08	0.289 (0.178-0.466)	153	364
KIRC	TCF12	0.0190257	0.69 (0.505-0.942)	277	240
KIRC	NR2C2	0.00215925	0.609 (0.442-0.839)	241	276
KIRC	USF1	8.99021e-06	0.44 (0.303-0.639)	199	318
KIRC	YY1	3.34266e-05	0.265 (0.135-0.52)	83	434
KIRC	ZBTB33	0.0084004	0.113 (0.016-0.805)	24	493
KIRC	ZEB1	5.97385e-05	2.142 (1.463-3.134)	68	449
KIRC	ZNF263	0.00801888	0.601 (0.411-0.879)	434	83
KIRP	RXRA	0.0372079	2.343 (1.027-5.346)	56	166
LIHC	EGR1	0.0129996	0.506 (0.293-0.875)	103	191
LUAD	JUND	0.00948775	1.635 (1.123-2.378)	232	241
LUAD	MEF2C	0.000982058	13.464 (1.777-102.048)	3	470
LUAD	TAL1_GATA1	0.0206886	1.537 (1.065-2.22)	225	248
LUAD	TCF12	0.037926	1.723 (1.025-2.897)	59	414
LUSC	FOS	0.0155074	0.61 (0.408-0.914)	130	296
LUSC	ELF1	0.0227034	3.518 (1.107-11.175)	6	420
LUSC	NRF1	0.0448748	3.057 (0.967-9.665)	6	420
LUSC	REST	0.0149819	0.651 (0.459-0.922)	278	148
THCA	EBF1	0.0420116	0.239 (0.053-1.07)	223	272
THCA	ECR_USP	0.0100013	0.216 (0.06-0.779)	458	37
THCA	HNF4A	0.0499348	0.357 (0.122-1.044)	361	134
THCA	PAX5	0.0399298	0.156 (0.02-1.196)	180	315
THCA	RXRA	0.0239565	0.247 (0.067-0.918)	447	48
THCA	RXRA_VDR	0.00828255	0.266 (0.093-0.763)	376	119
UCEC	E2F4	0.00221894	0.401 (0.218-0.734)	323	184
UCEC	E2F6	0.035514	0.487 (0.245-0.966)	211	296
UCEC	ELF1	0.0113017	0.319 (0.126-0.811)	153	354
UCEC	GATA2	0.017712	0.469 (0.248-0.89)	240	267
UCEC	HNF4G	0.041277	0.529 (0.285-0.985)	264	243

UCEC	NR1H3_RXRA	0.0386898	0.526 (0.283-0.977)	247	260
UCEC	PAX5	0.0111818	0.366 (0.163-0.822)	176	331

Supplementary Figure 1.

Change of TF activity in the different cancers studied. Cells in red indicate increased activity of the TF in the cancer with respect to the corresponding normal tissue, according to the TFTEA, cells in blue indicate a decreased activity and cells in grey indicate that no significant change in activity was detected. In addition, cells contain the discretized (Not detected / low / medium / high) protein expression value, according to the Human Proteome Atlas [57] of the TF in the healthy tissue in which the cancer originated, when available, otherwise a “0” appears. Columns correspond to cancers and rows to TFs.

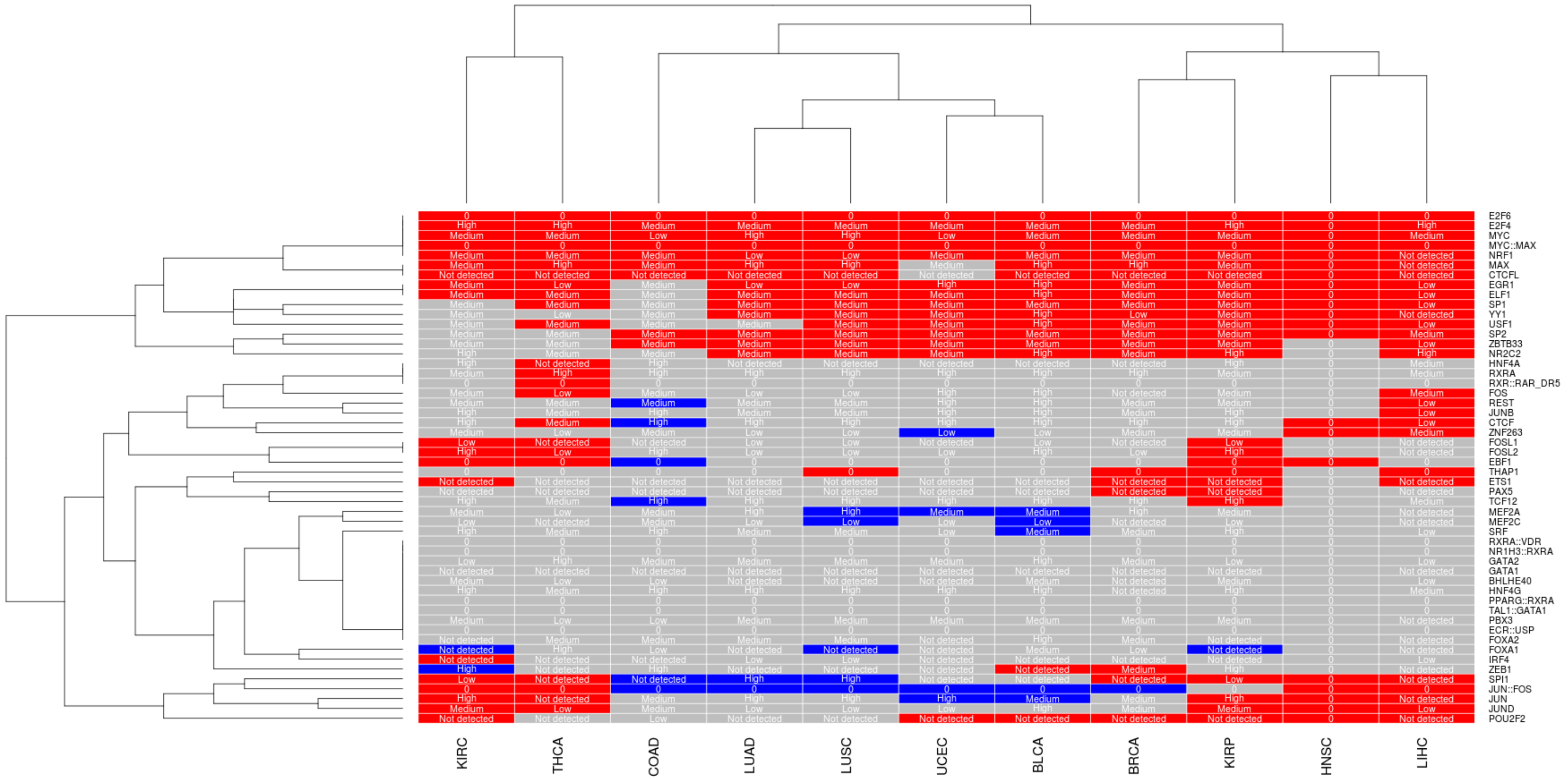
KIRC
THCA
COAD
LUAD
LUSC
UCEC
BLCA
BRCA
KIRP
HNSC
LIHC
E2F6
E2F4
MYC
MYC::MAX
NRF1
MAX
CTCFL
EGR1
ELF1
SP1
YY1
USF1
SP2
ZBTB33
NR2C2
HNF4A
RXRA
RXR::RAR_DR5
FOS
REST
JUNB
CTCF
ZNF263
FOSL1
FOSL2
EBF1
THAP1
ETS1
PAX5
TCF12
MEF2A
MEF2C
SRF
RXRA::VDR
NR1H3::RXRA
GATA2
GATA1
BHLHE40
HNF4G
PPARG::RXRA
TAL1::GATA1
PBX3
ECR::USP
FOXA2
FOXA1
IRF4
ZEB1
SPI1
JUN::FOS
JUN
JUND
POU2F2

Supplementary Figure 2.

K-M detailed plots representing TF activities significantly associated to patient survival in all the cancers analysed from Figure 3.

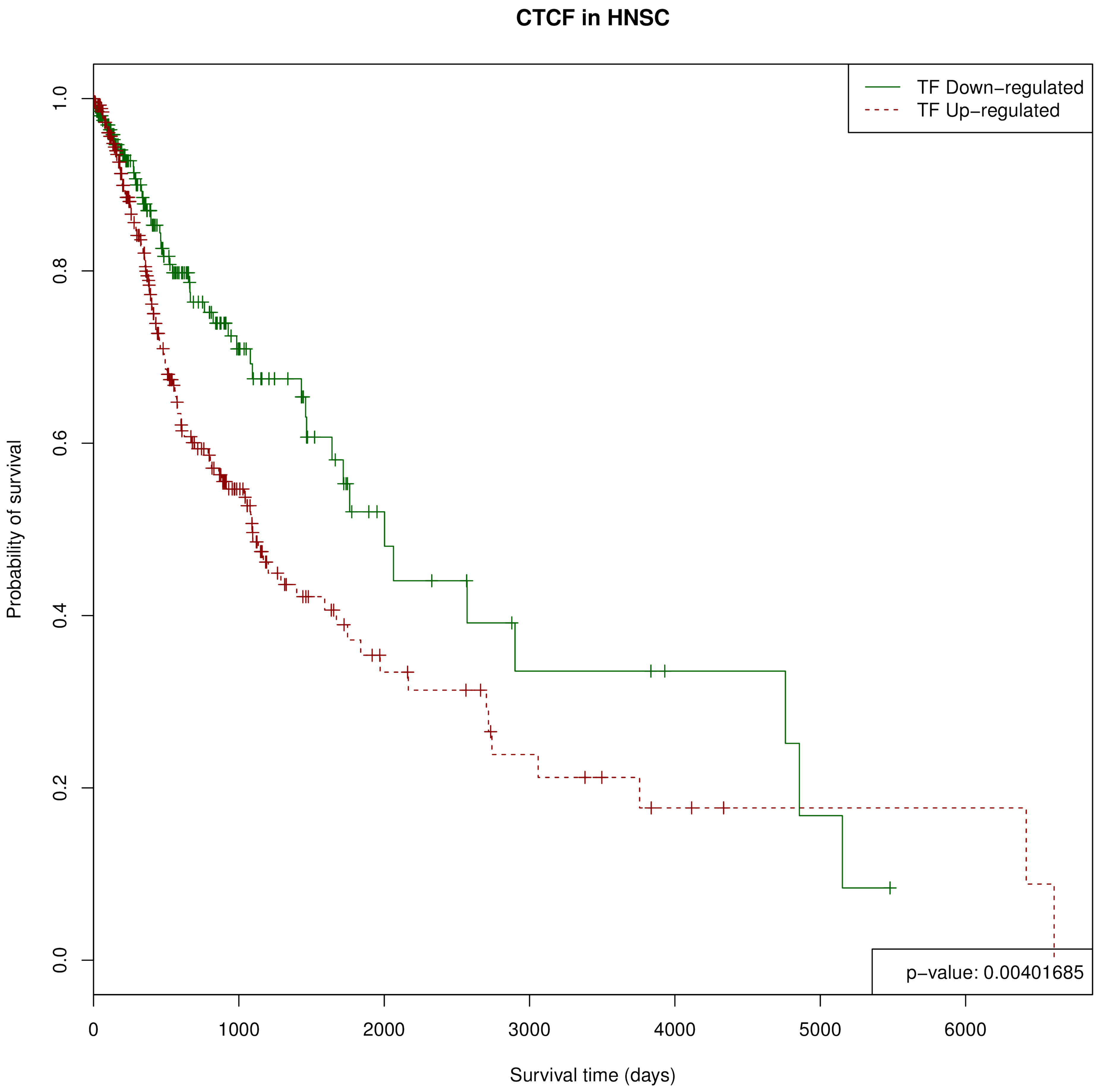

CTCF in HNSC
TF Down−regulated
TF Up−regulated
p−value: 0.00401685
Probability of survival
Survival time (days)
0.0
0.2
0.4
0.6
0.8
1.0
0
1000
2000
3000
4000
5000
6000

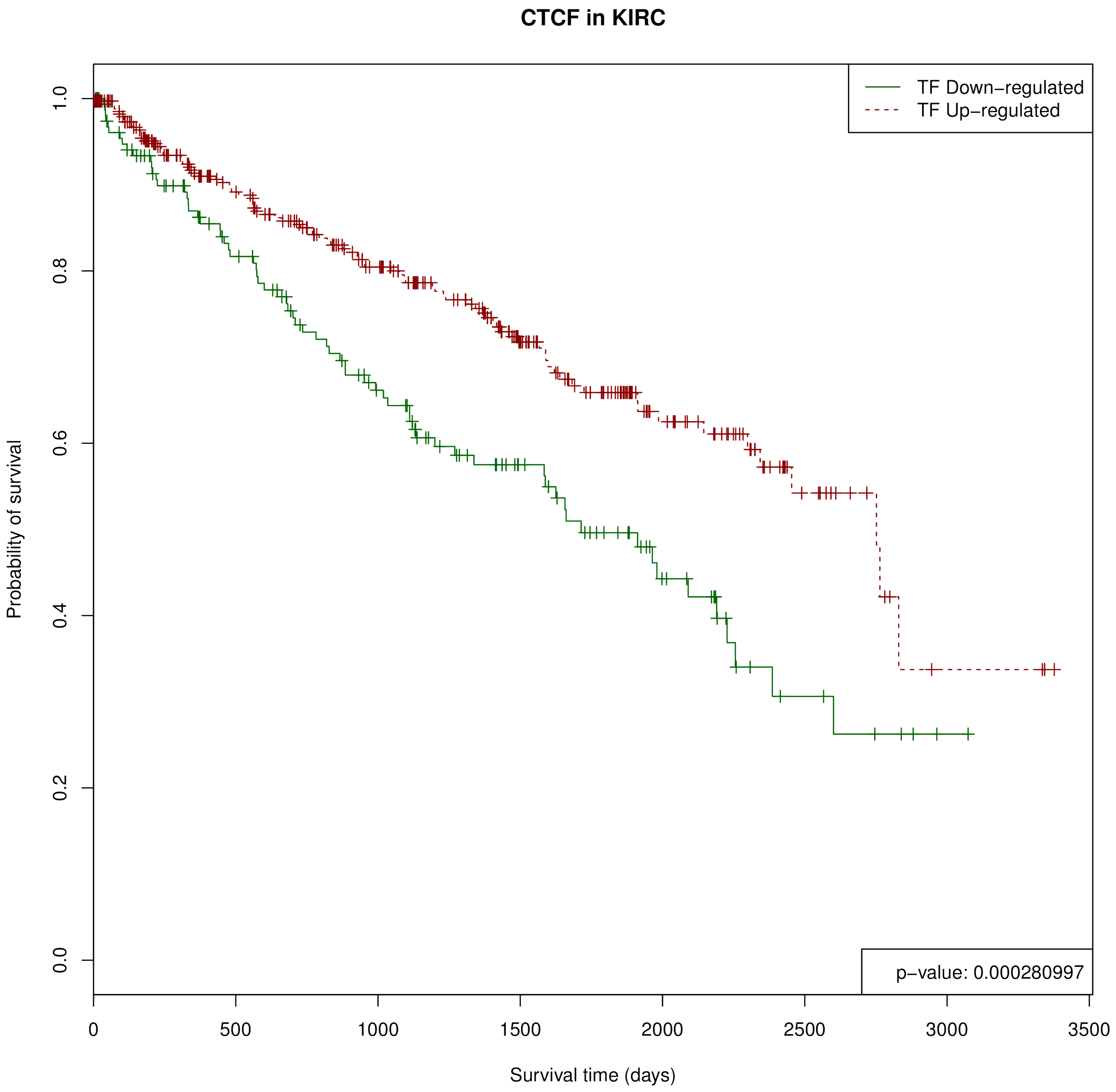
CTCF in KIRC
TF Down−regulated
TF Up−regulated
p−value: 0.000280997
Probability of survival
1.0
0.8
0.6
0.4
0.2
0.0
Survival time (days)
0
500
1000
1500
2000
2500
3000
3500

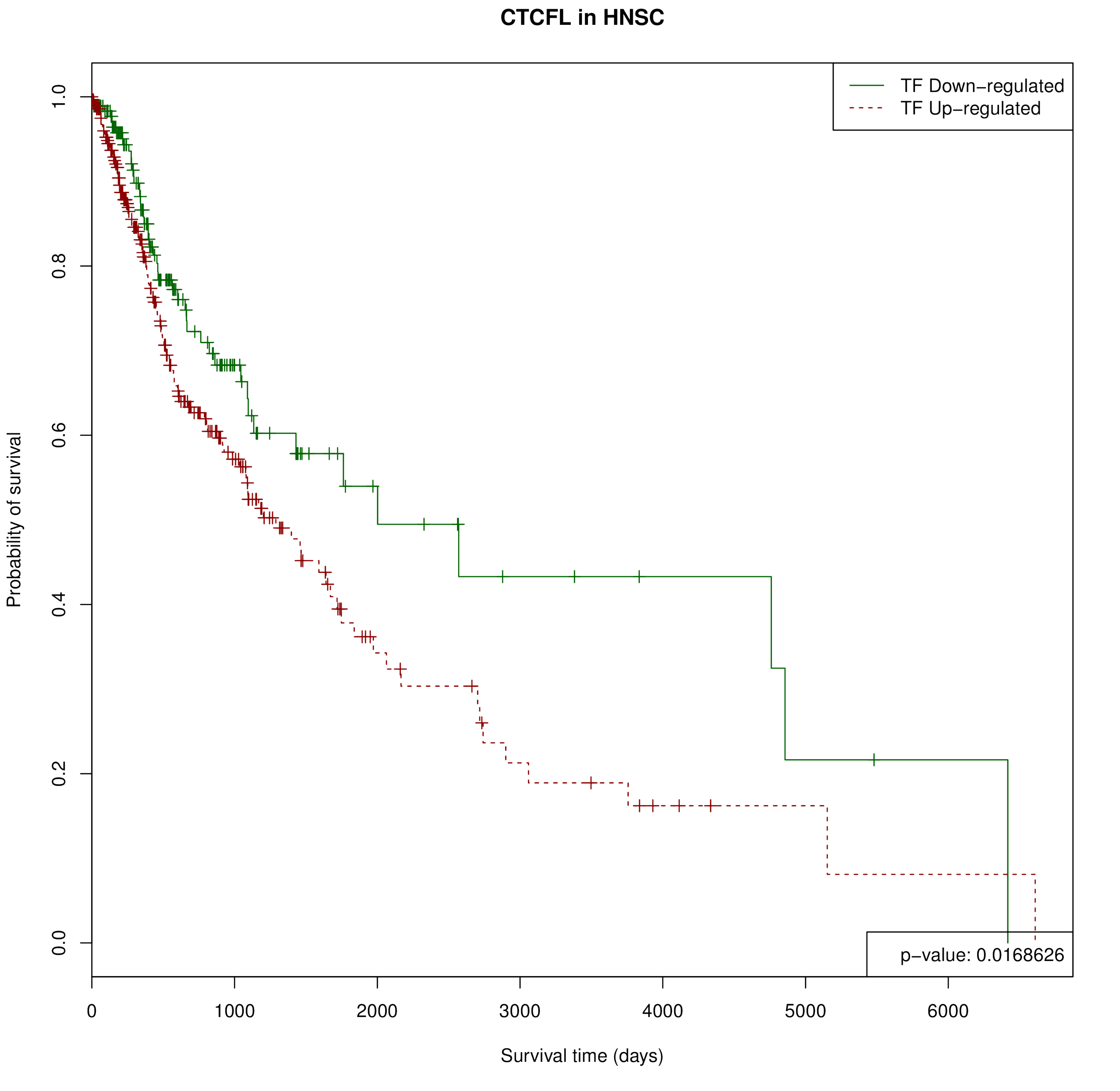
CTCFL in HNSC
TF Down−regulated
TF Up−regulated
Probability of survival
Survival time (days)
p−value: 0.0168626
0.0
0.2
0.4
0.6
0.8
1.0
0
1000
2000
3000
4000
5000
6000

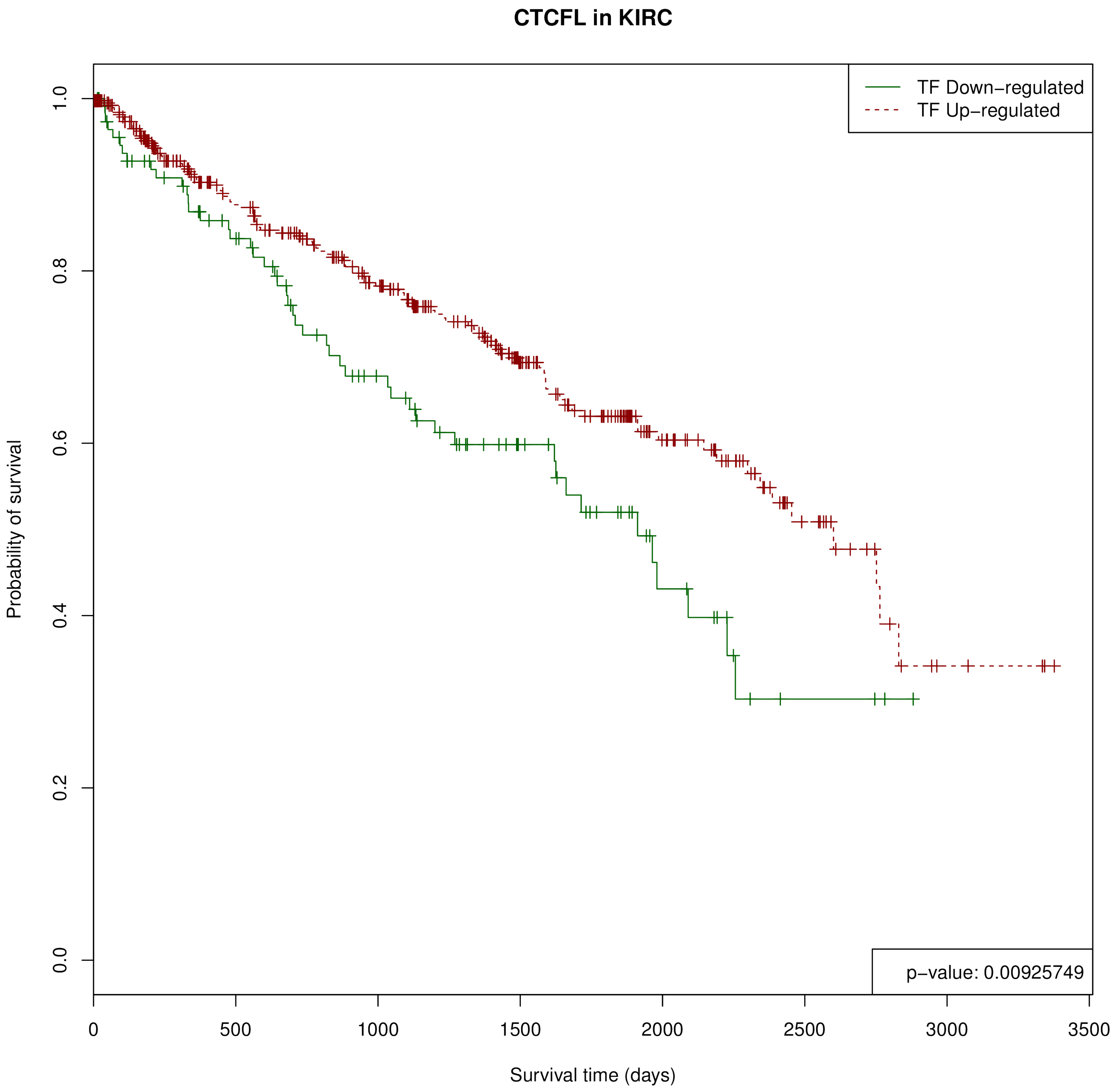

CTCFL in KIRC
TF Down−regulated
TF Up−regulated
p−value: 0.00925749
Probability of survival
Survival time (days)
0.0
0.2
0.4
0.6
0.8
1.0
0
500
1000
1500
2000
2500
3000
3500

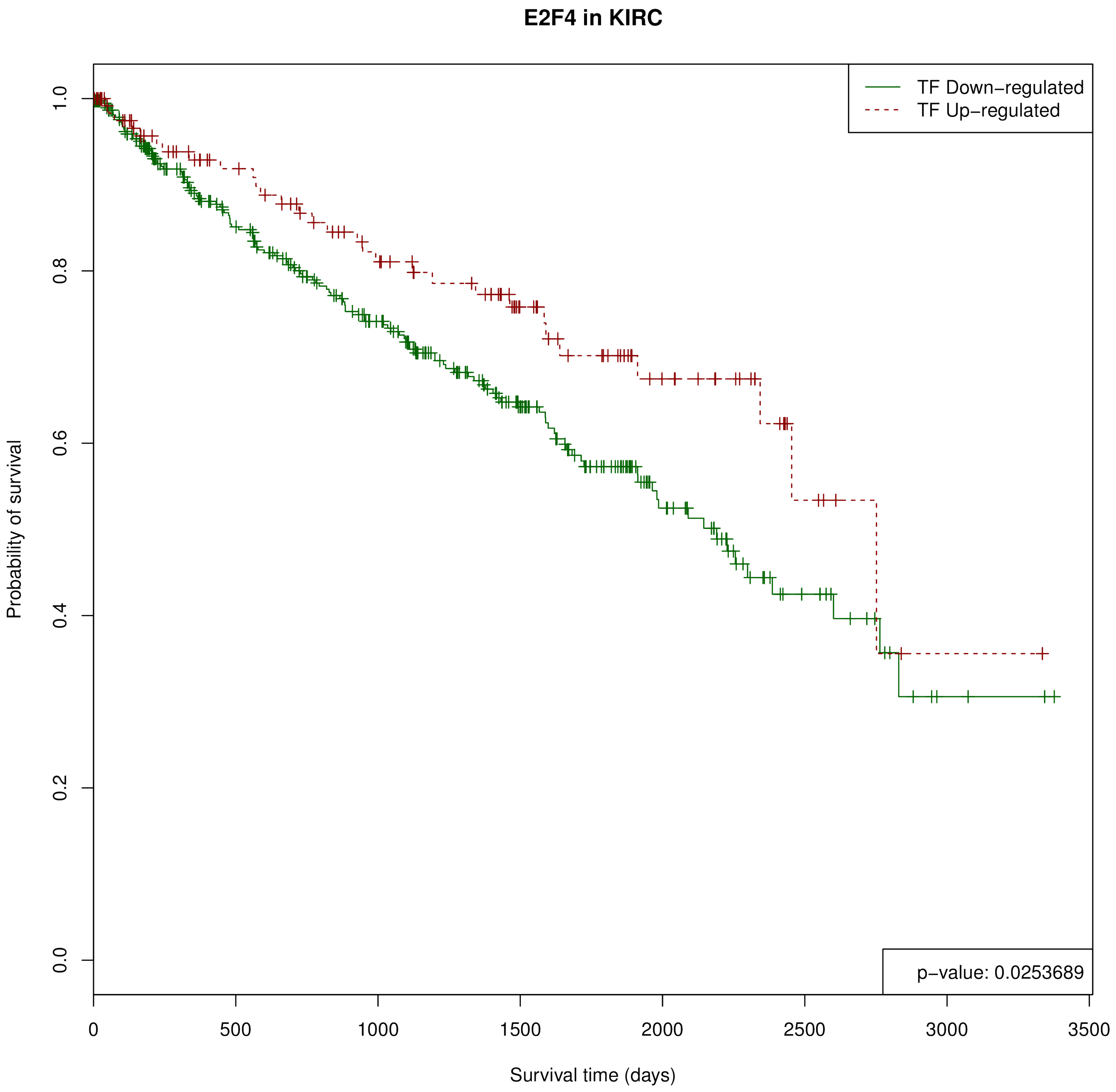
E2F4 in KIRC
TF Down−regulated
TF Up−regulated
p−value: 0.0253689
Probability of survival
Survival time (days)
0.0
0.2
0.4
0.6
0.8
1.0
0
500
1000
1500
2000
2500
3000
3500

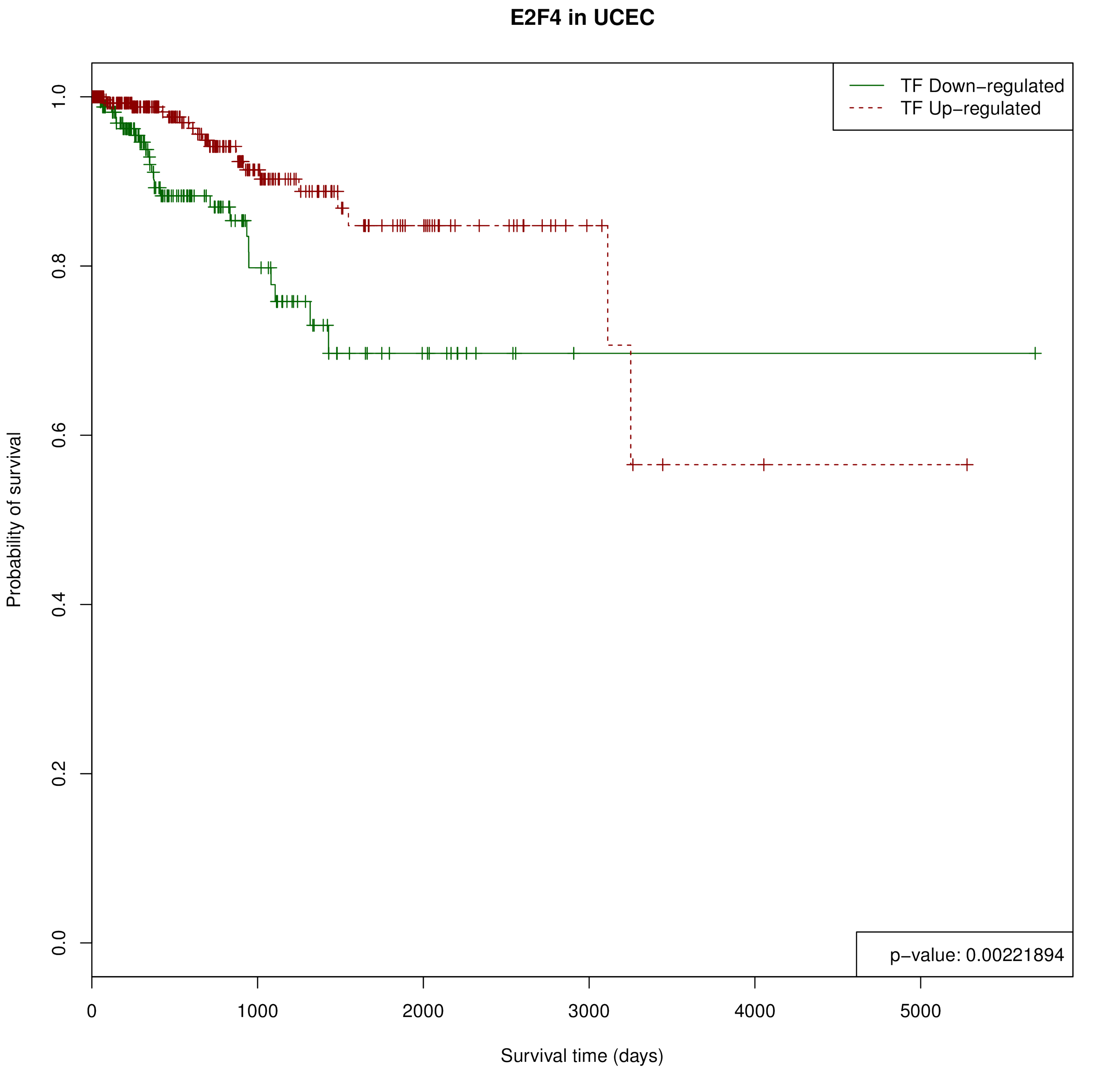
E2F4 in UCEC
TF Down−regulated
TF Up−regulated
p−value: 0.00221894
Probability of survival
Survival time (days)
0.0
0.2
0.4
0.6
0.8
1.0
0
1000
2000
3000
4000
5000

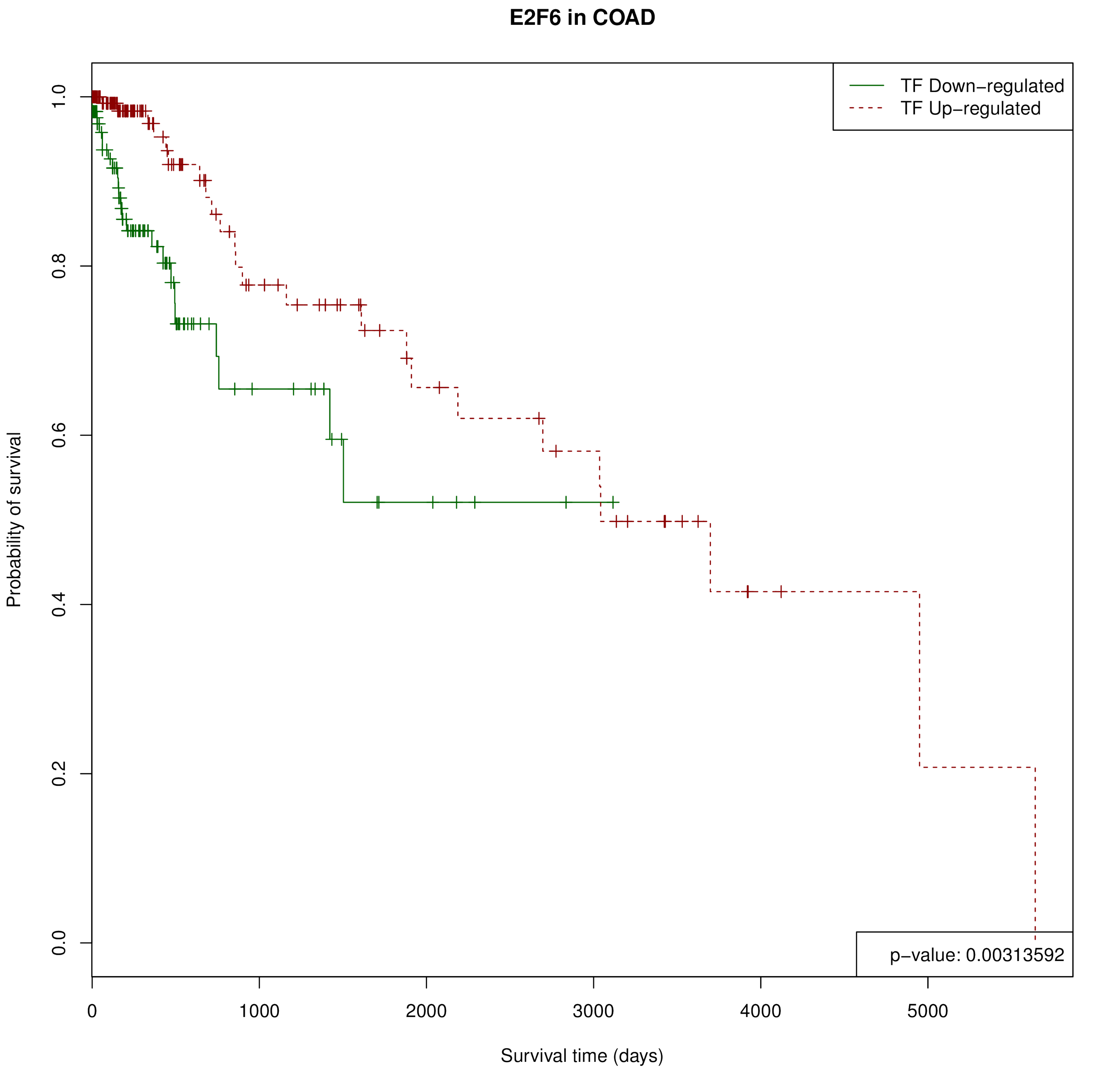
E2F6 in COAD
TF Down−regulated
TF Up−regulated
Probability of survival
0.0
0.2
0.4
0.6
0.8
1.0
0
1000
2000
3000
4000
5000
Survival time (days)
p−value: 0.00313592

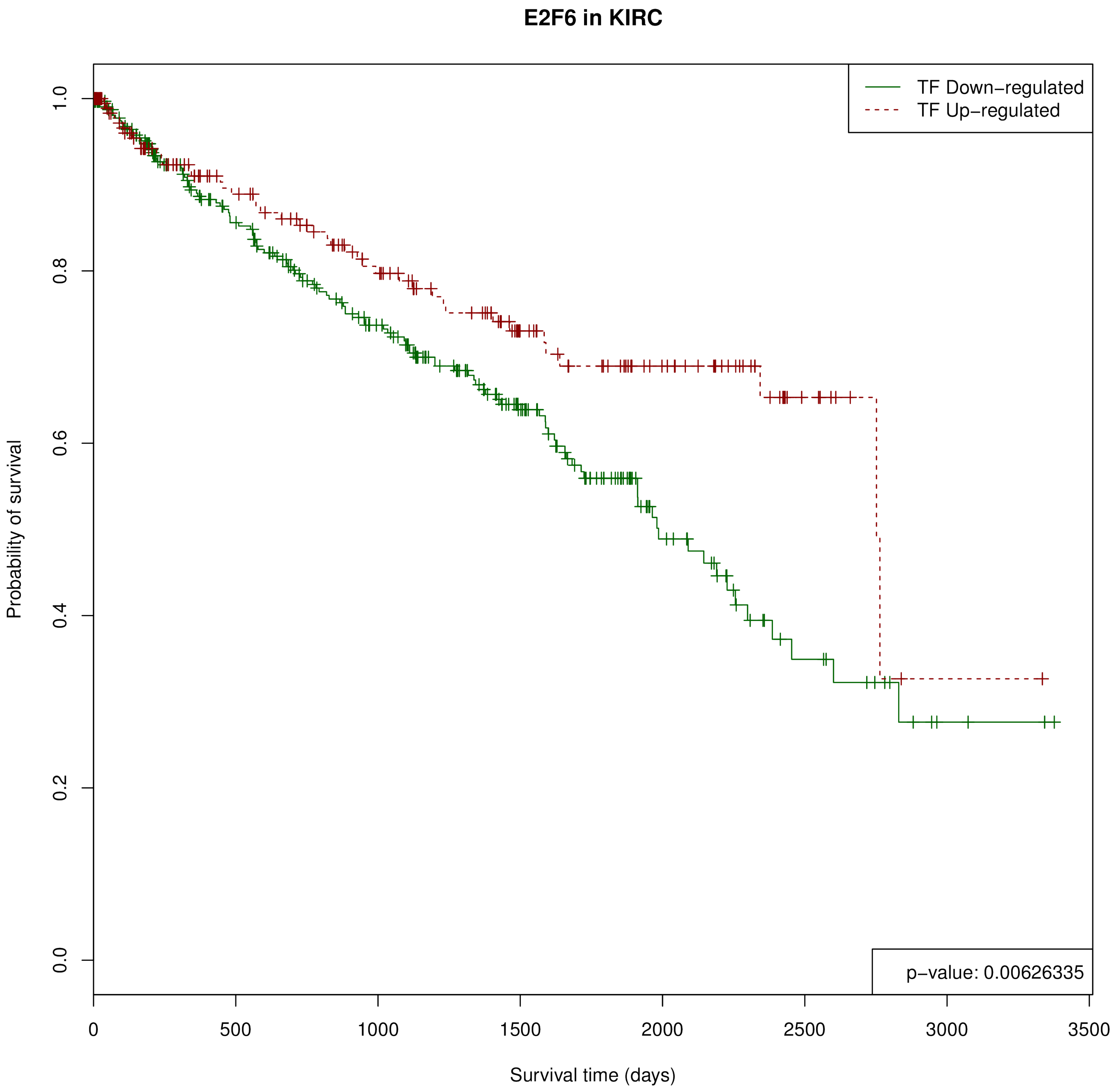

E2F6 in KIRC
TF Down−regulated
TF Up−regulated
p−value: 0.00626335
Probability of survival
Survival time (days)
0.0
0.2
0.4
0.6
0.8
1.0
0
500
1000
1500
2000
2500
3000
3500

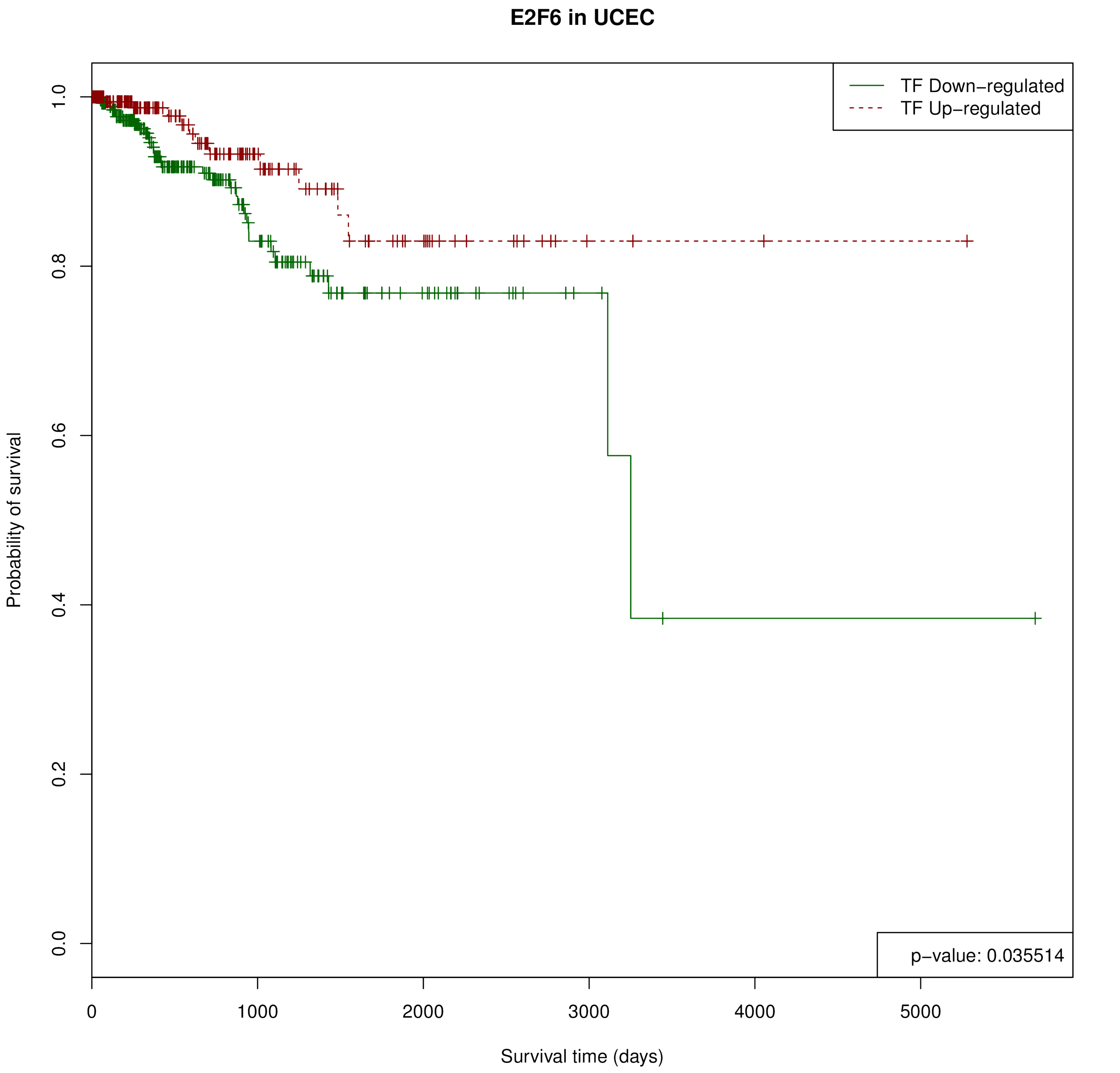

E2F6 in UCEC
TF Down−regulated
TF Up−regulated
p−value: 0.035514
Probability of survival
Survival time (days)
0.0
0.2
0.4
0.6
0.8
1.0
0
1000
2000
3000
4000
5000

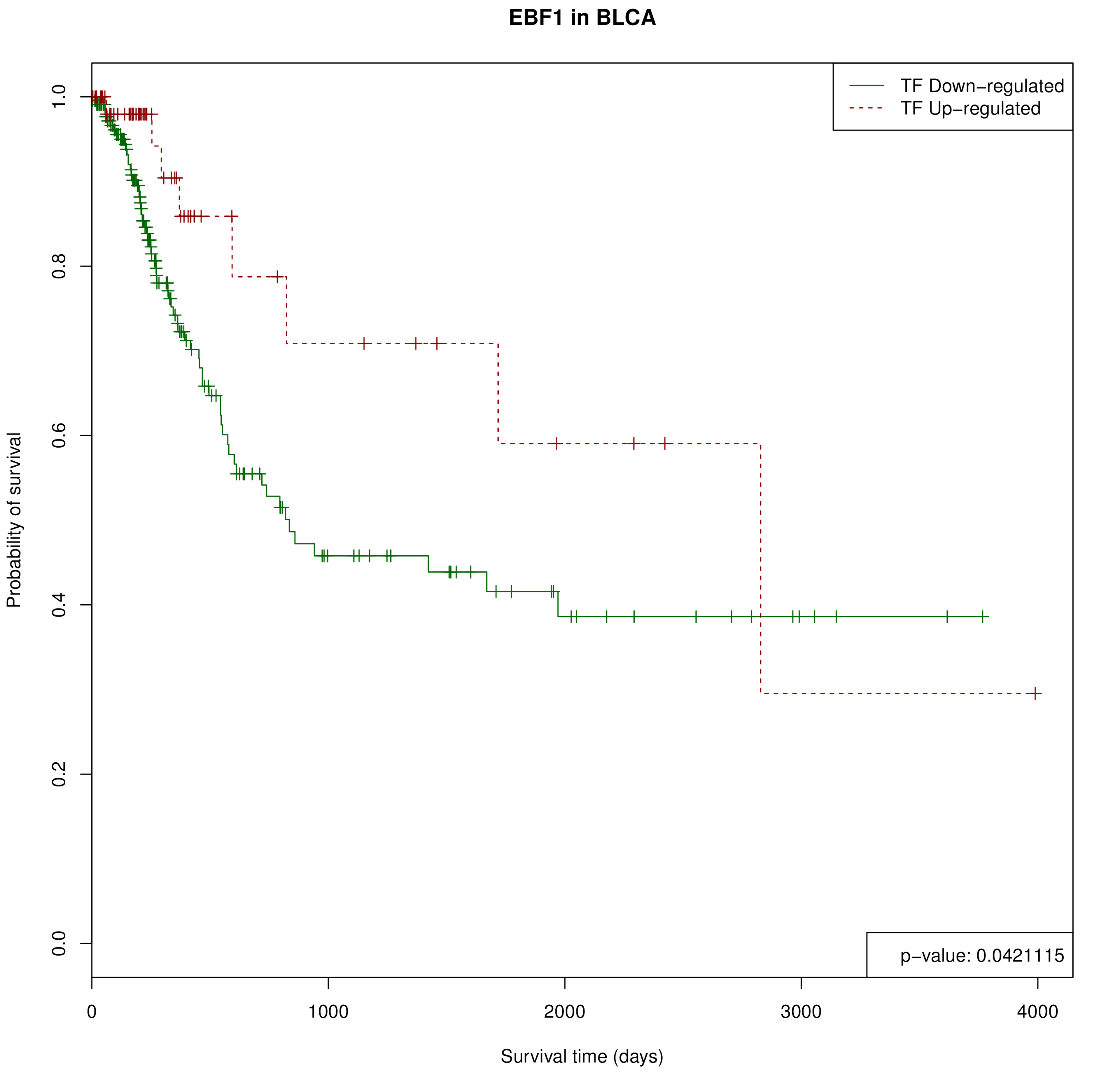
EBF1 in BLCA
TF Down−regulated
TF Up−regulated
p−value: 0.0421115
Probability of survival
Survival time (days)
0.0
0.2
0.4
0.6
0.8
1.0
0
1000
2000
3000
4000

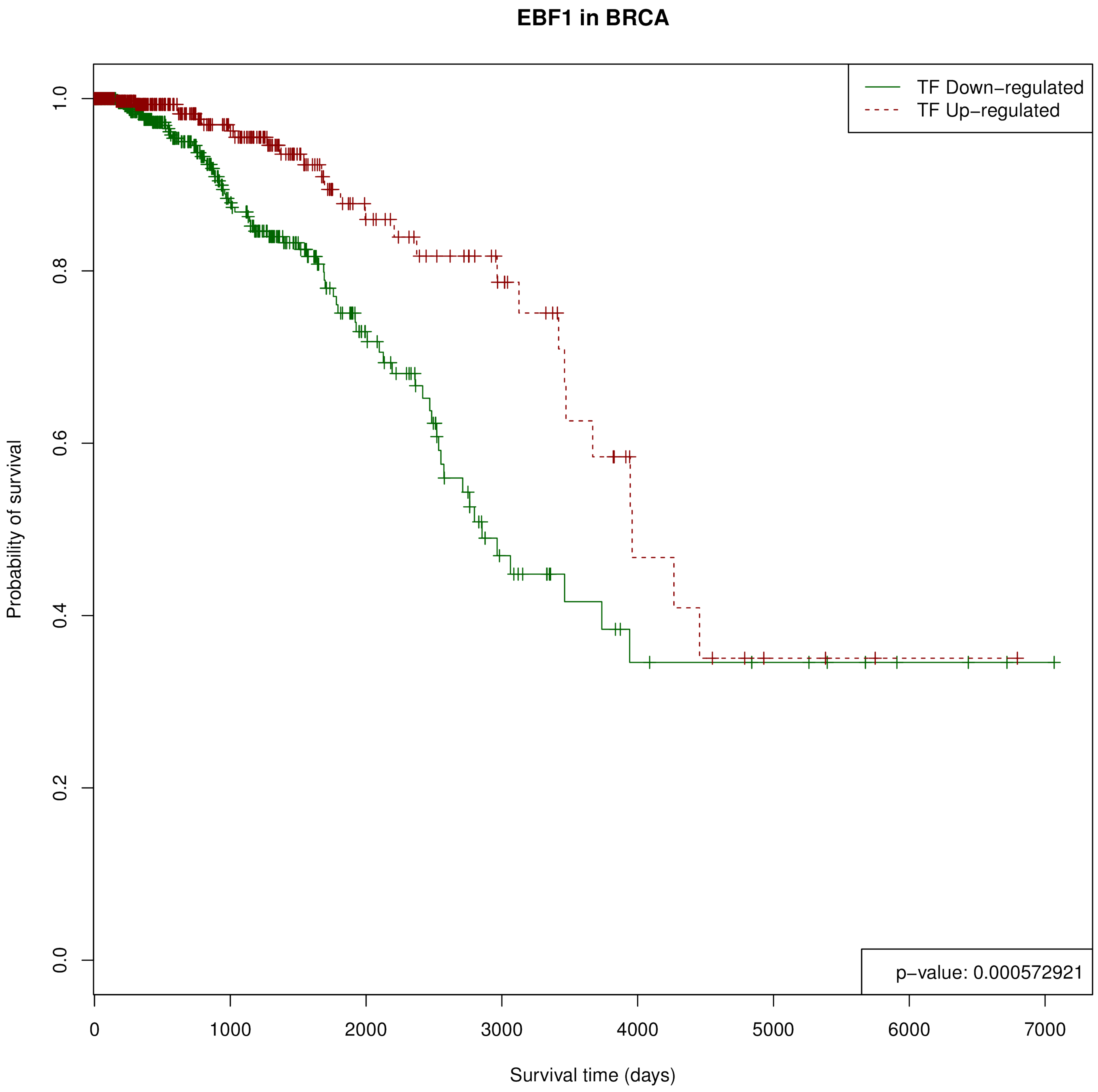

EBF1 in BRCA
TF Down−regulated
TF Up−regulated
p−value: 0.000572921
Probability of survival
Survival time (days)
0.0
0.2
0.4
0.6
0.8
1.0
0
1000
2000
3000
4000
5000
6000
7000

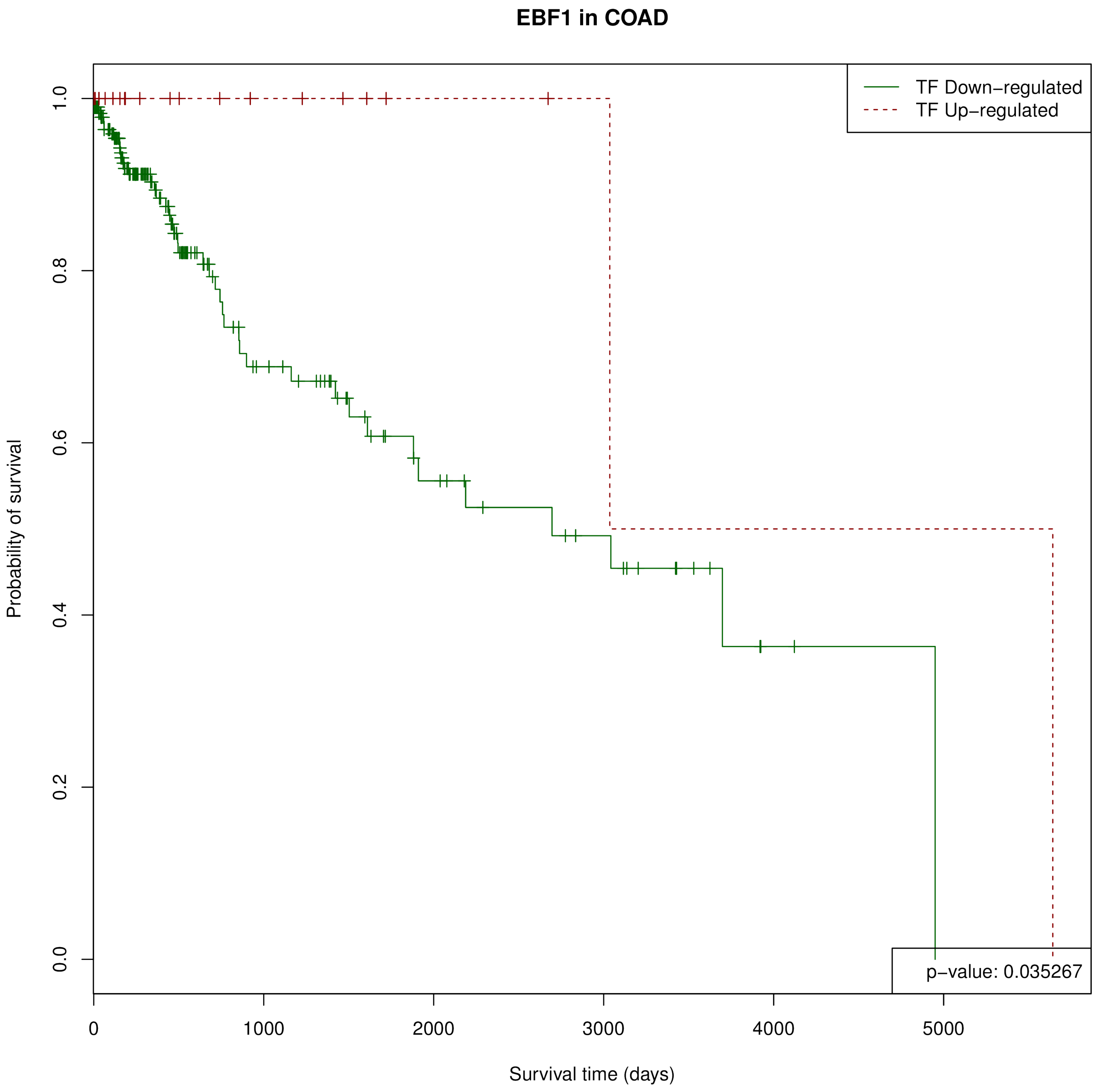

EBF1 in COAD
TF Down−regulated
TF Up−regulated
Probability of survival
0.0
0.2
0.4
0.6
0.8
1.0
0
1000
2000
3000
4000
5000
Survival time (days)
p−value: 0.035267

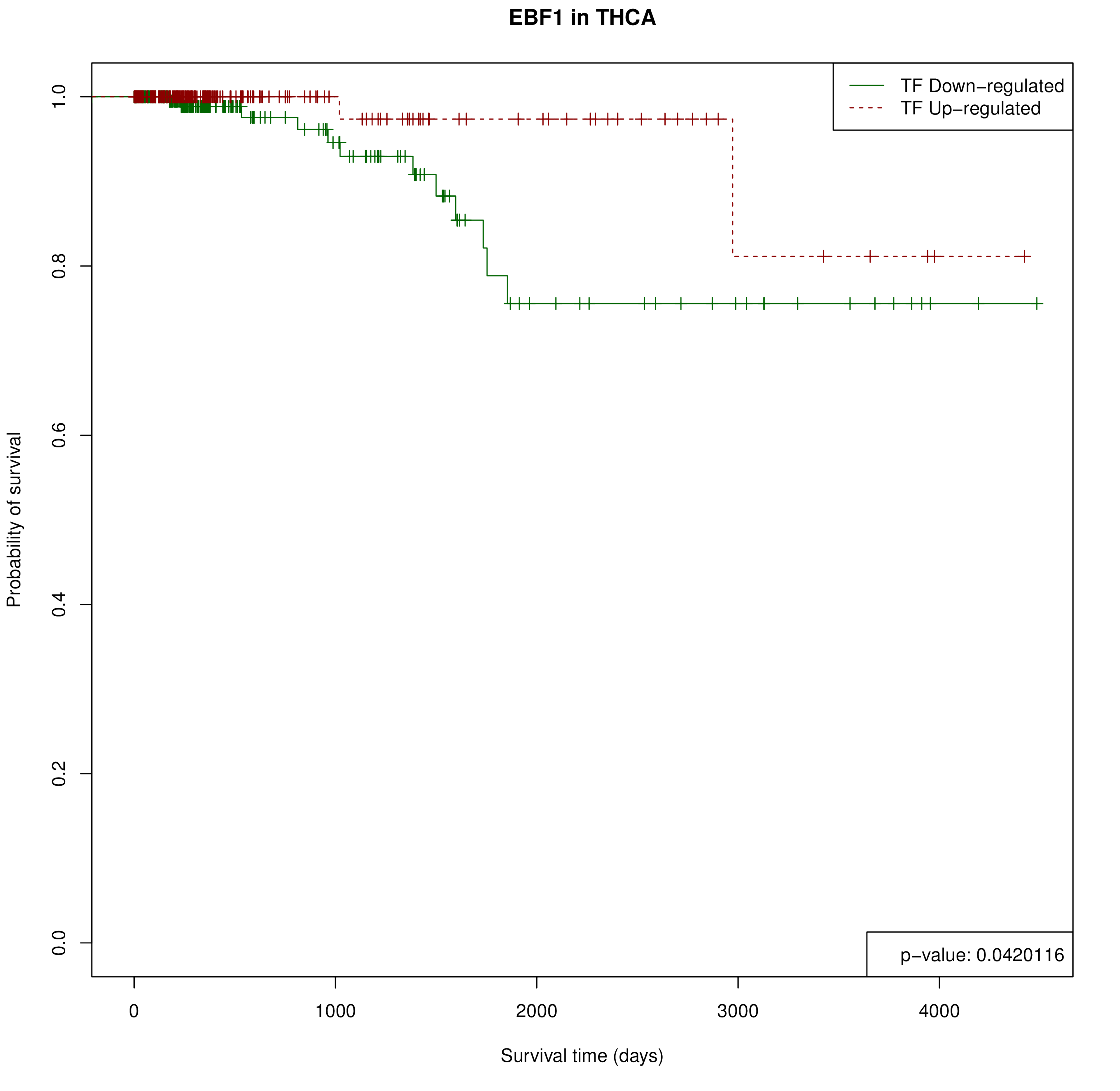

EBF1 in THCA
TF Down−regulated
TF Up−regulated
Probability of survival
0.0
0.2
0.4
0.6
0.8
1.0
Survival time (days)
0
1000
2000
3000
4000
p−value: 0.0420116

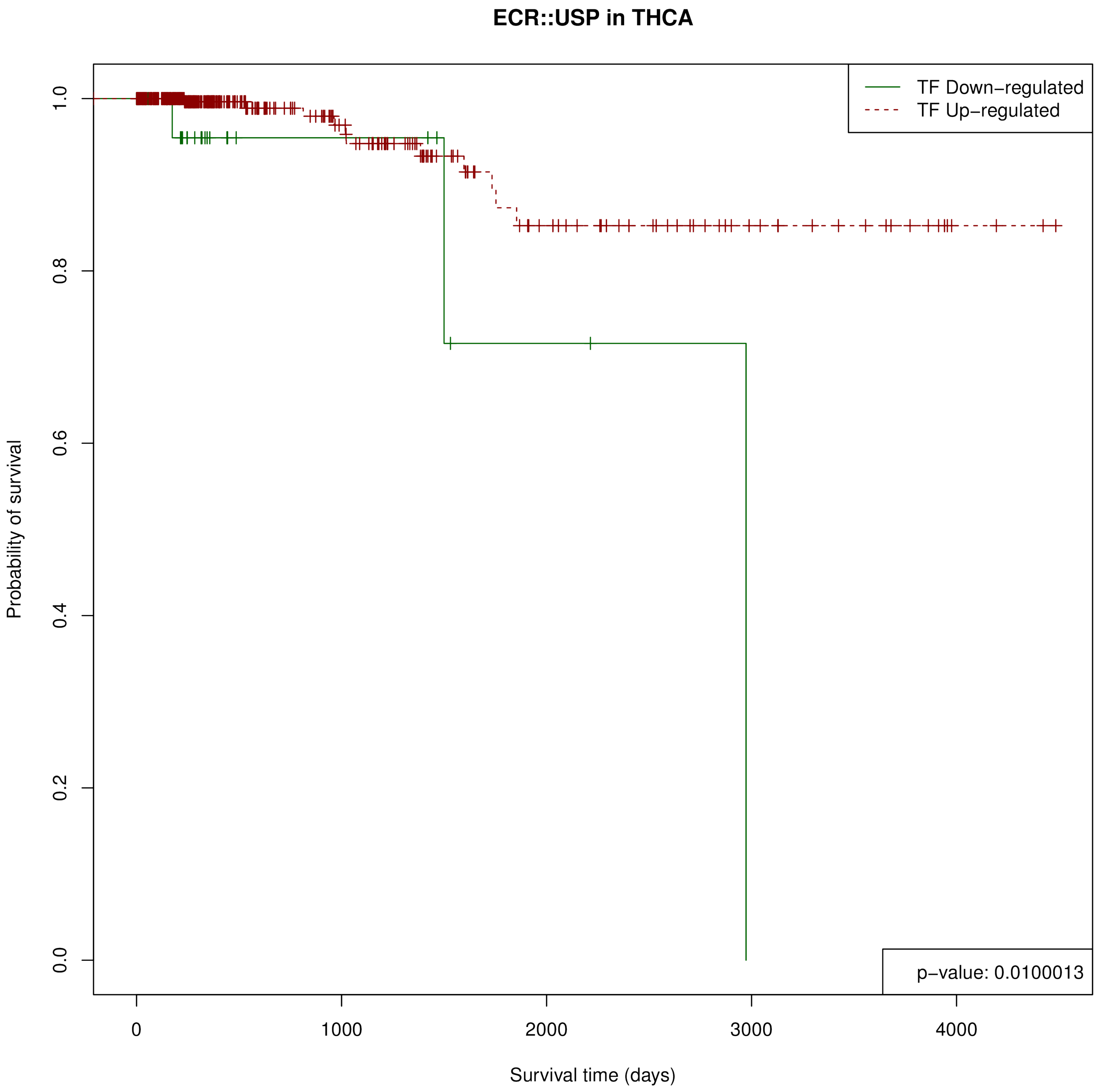
ECR::USP in THCA
TF Down−regulated
TF Up−regulated
p−value: 0.0100013
Probability of survival
Survival time (days)
0.0
0.2
0.4
0.6
0.8
1.0
0
1000
2000
3000
4000

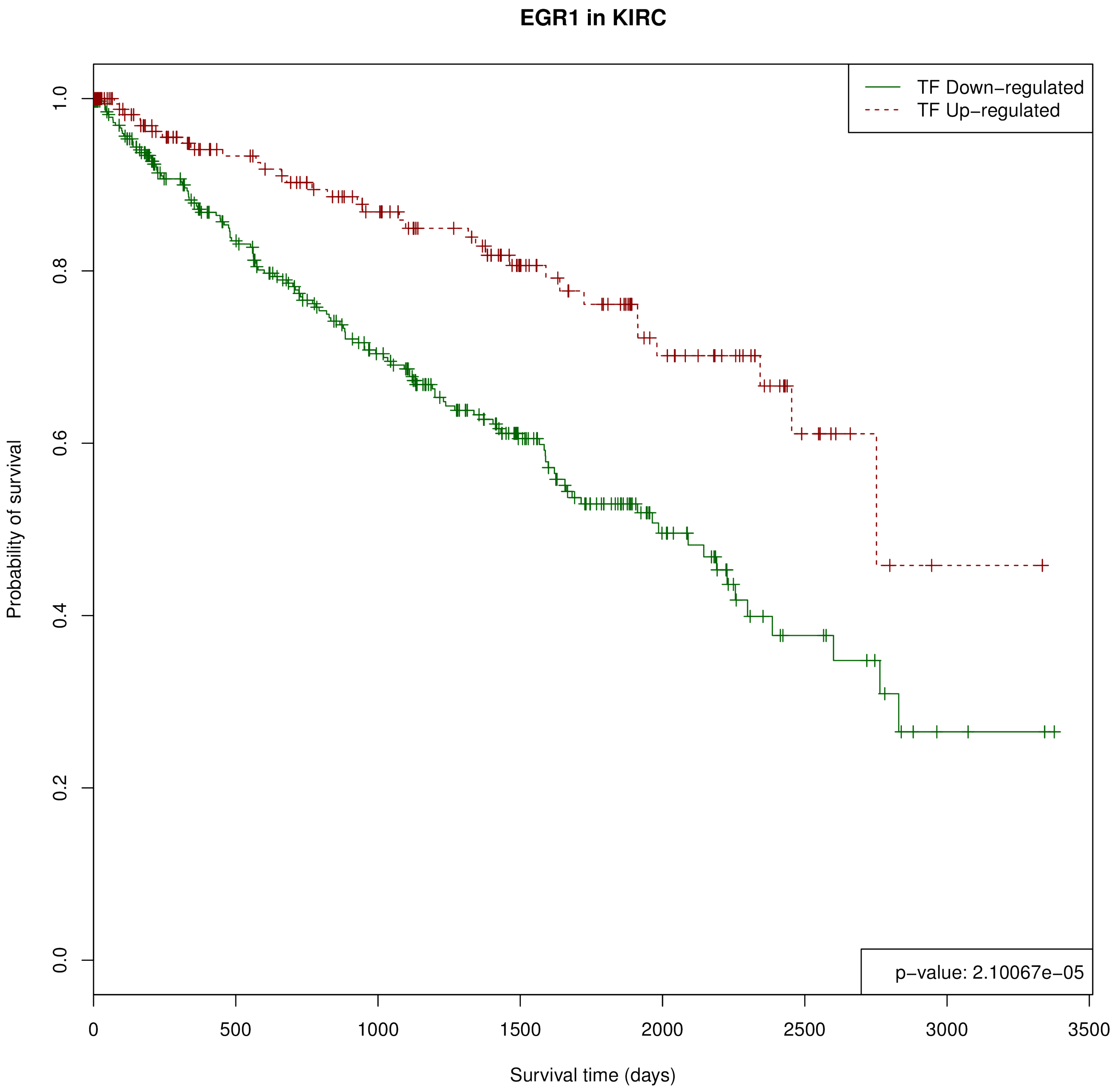
EGR1 in KIRC
TF Down−regulated
TF Up−regulated
p−value: 2.10067e−05
Probability of survival
0.0
0.2
0.4
0.6
0.8
1.0
Survival time (days)
0
500
1000
1500
2000
2500
3000
3500

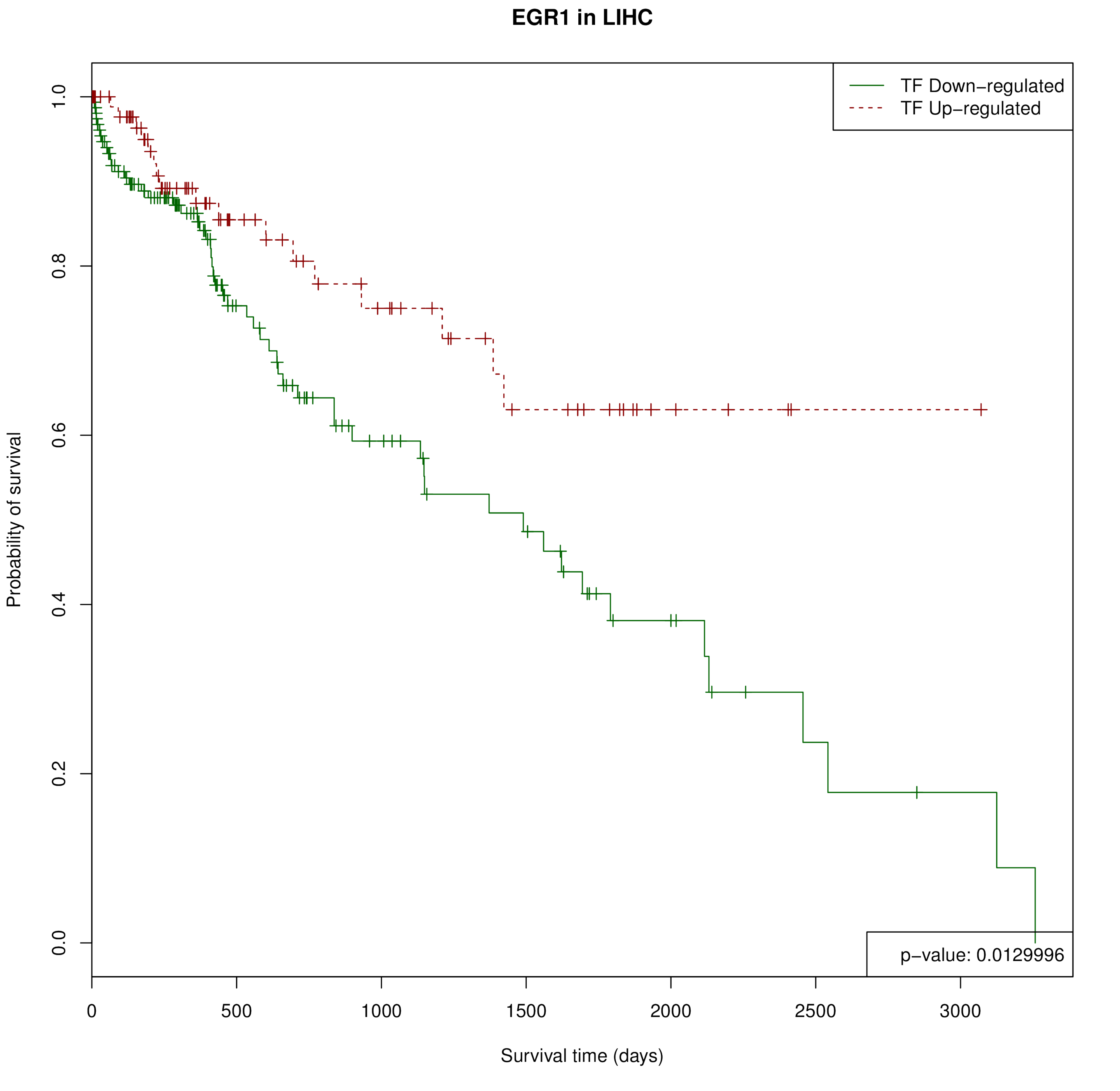
EGR1 in LIHC
TF Down−regulated
TF Up−regulated
Probability of survival
0.0
0.2
0.4
0.6
0.8
1.0
0
500
1000
1500
2000
2500
3000
Survival time (days)
p−value: 0.0129996

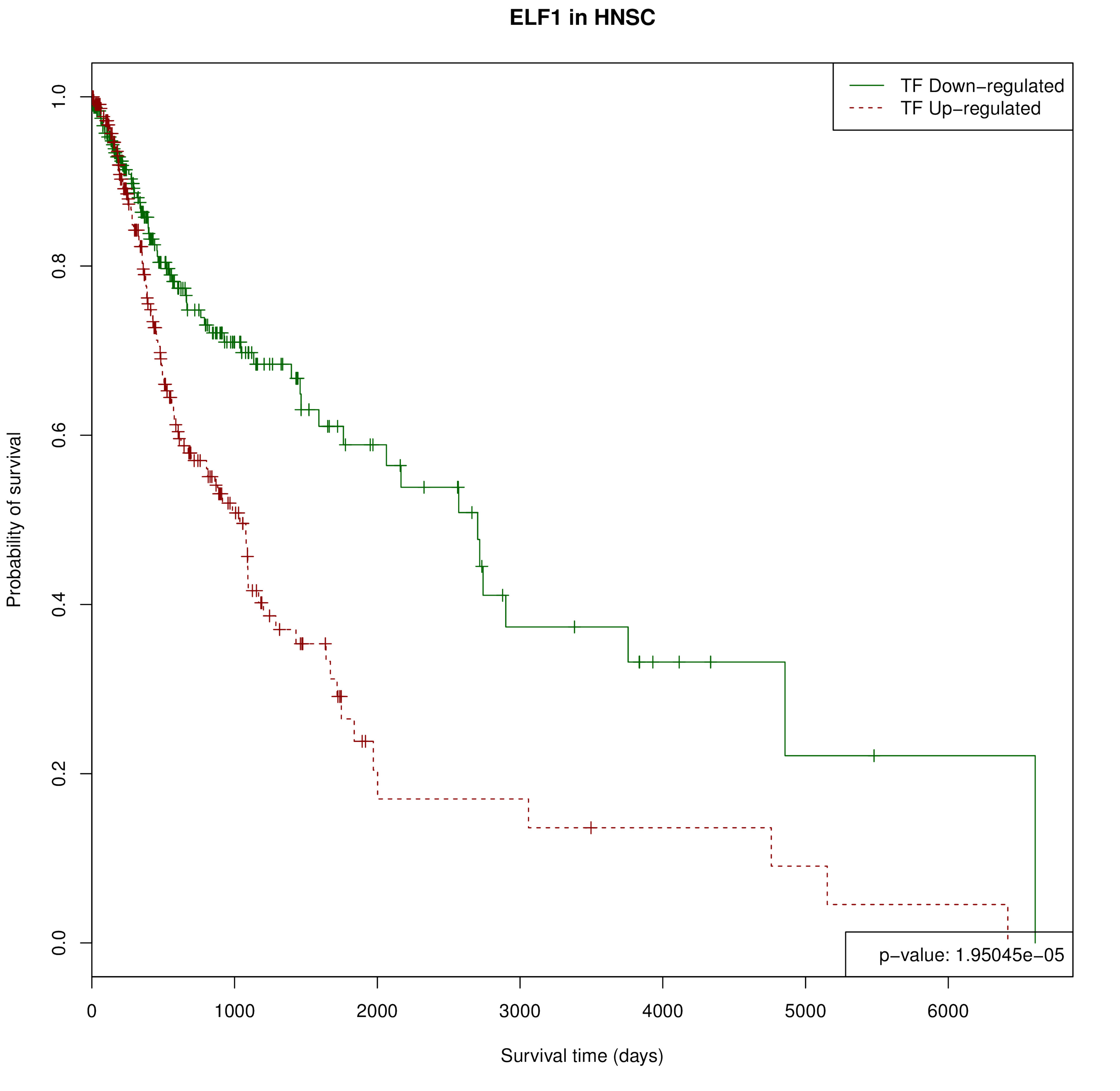

ELF1 in HNSC
TF Down−regulated
TF Up−regulated
p−value: 1.95045e−05
Probability of survival
Survival time (days)
0.0
0.2
0.4
0.6
0.8
1.0
0
1000
2000
3000
4000
5000
6000

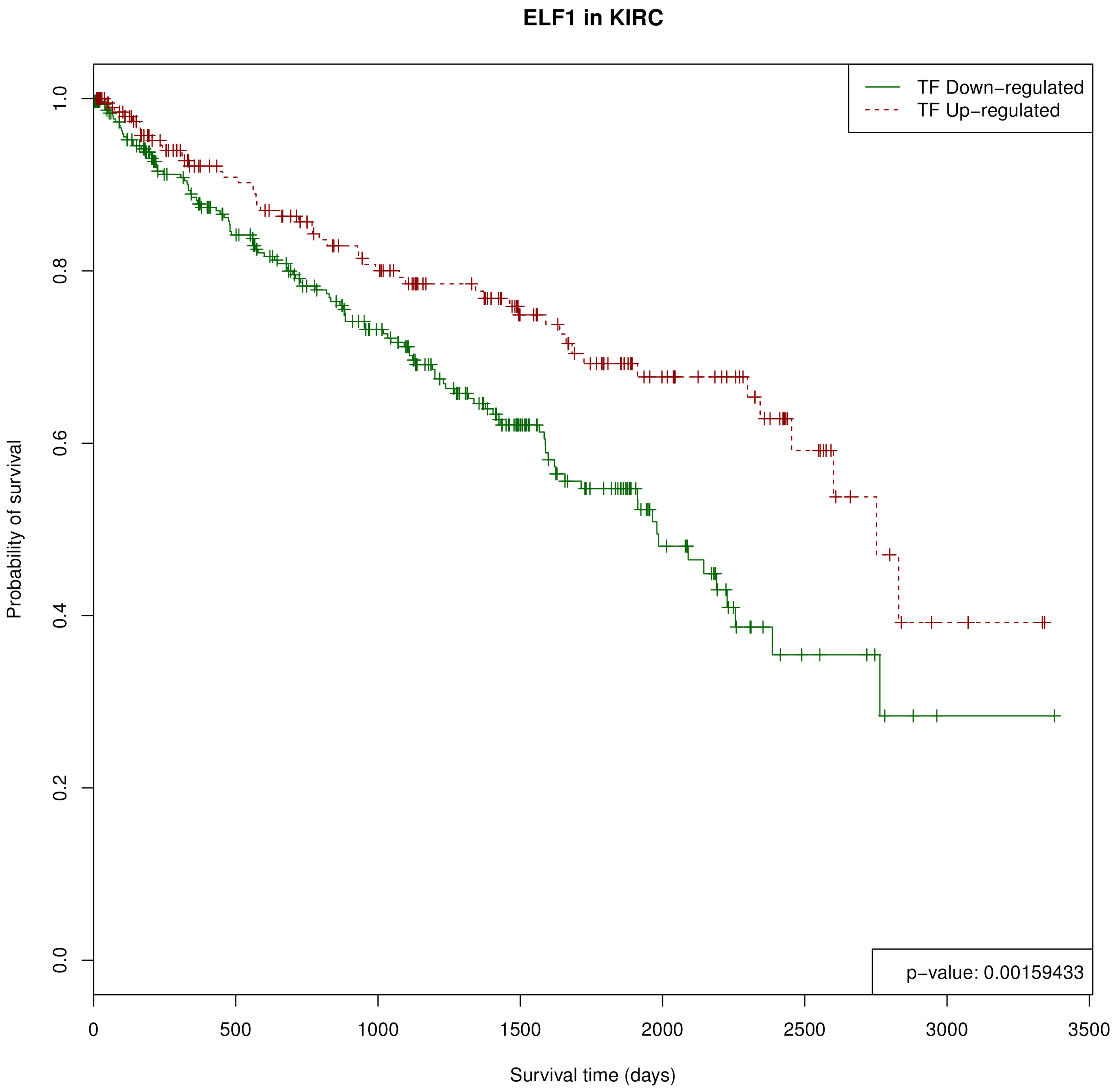

ELF1 in KIRC
TF Down−regulated
TF Up−regulated
p−value: 0.00159433
Probability of survival
Survival time (days)
0.0
0.2
0.4
0.6
0.8
1.0
0
500
1000
1500
2000
2500
3000
3500

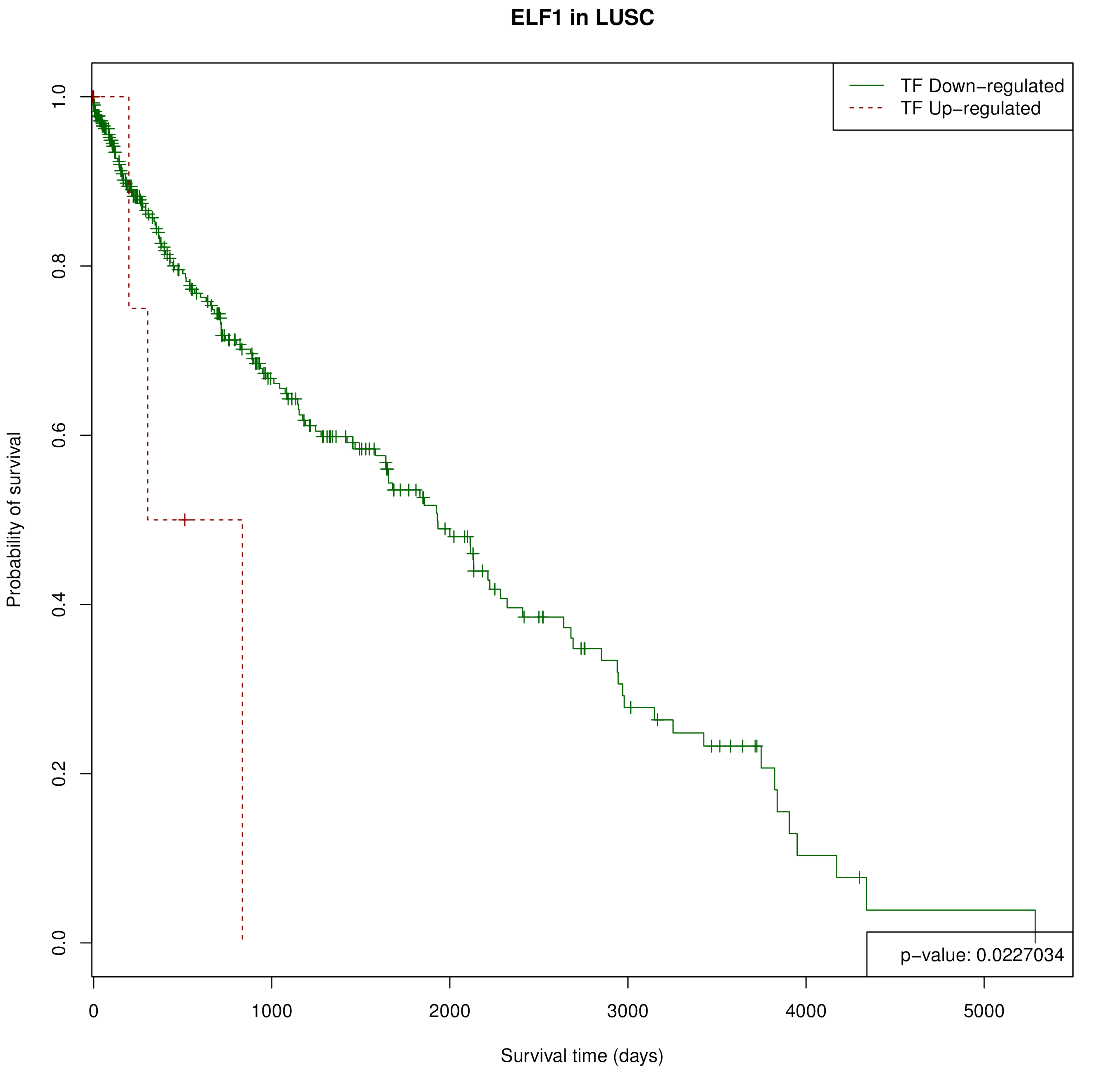
ELF1 in LUSC
TF Down−regulated
TF Up−regulated
Probability of survival
0.0
0.2
0.4
0.6
0.8
1.0
0
1000
2000
3000
4000
5000
Survival time (days)
p−value: 0.0227034

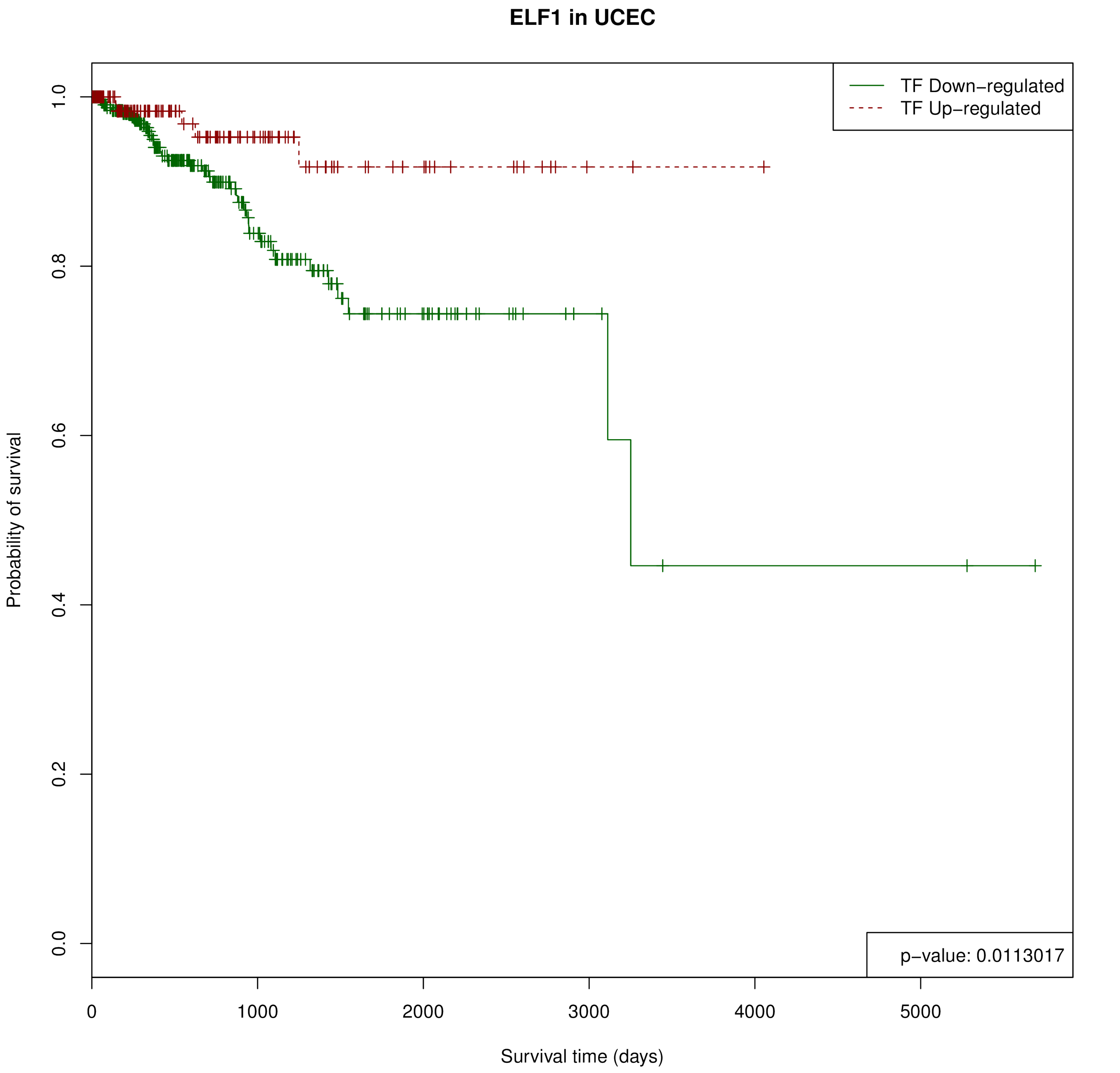
ELF1 in UCEC
TF Down−regulated
TF Up−regulated
p−value: 0.0113017
Probability of survival
Survival time (days)
0.0
0.2
0.4
0.6
0.8
1.0
0
1000
2000
3000
4000
5000

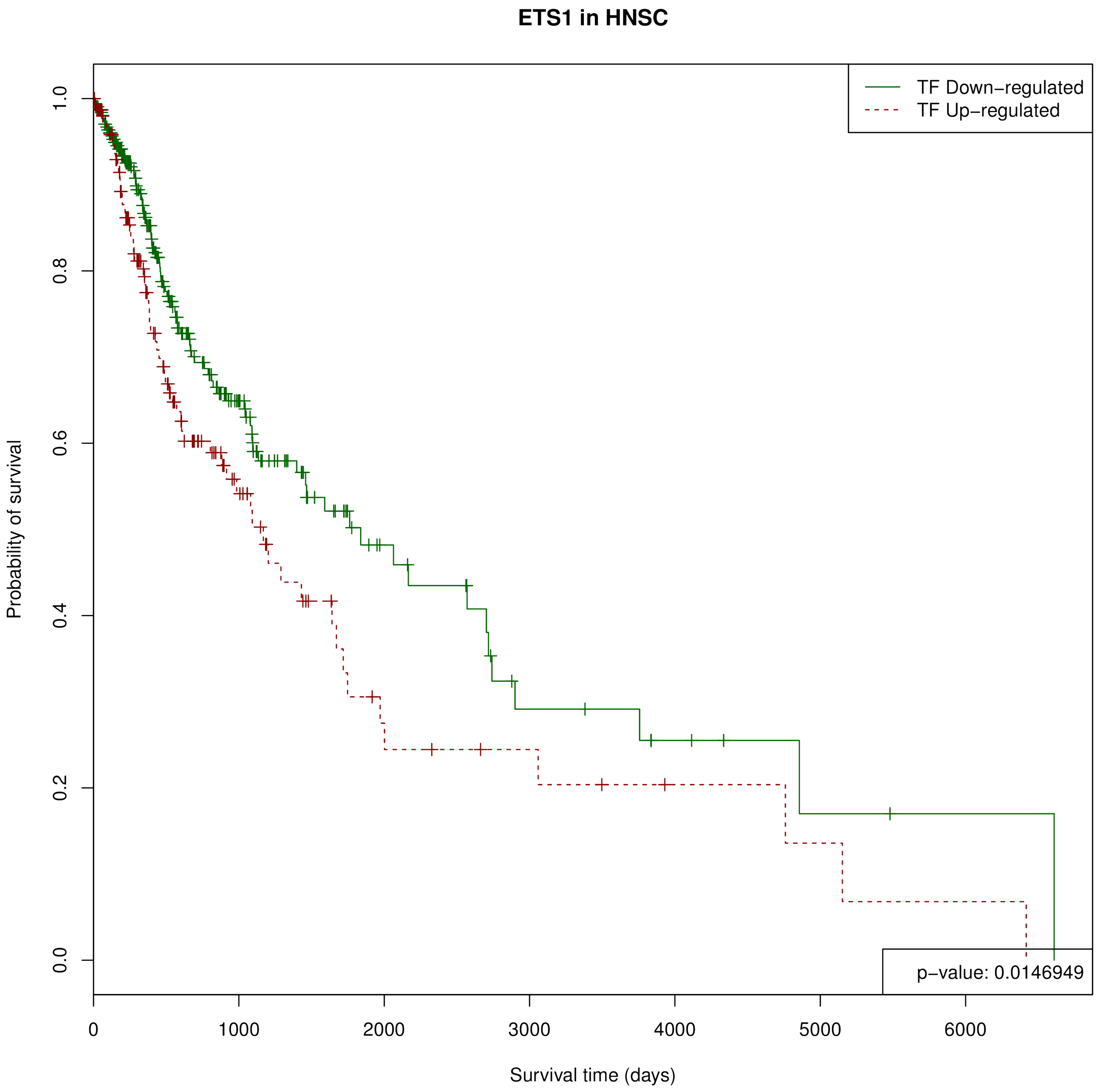

ETS1 in HNSC
TF Down−regulated
TF Up−regulated
Probability of survival
0.0
0.2
0.4
0.6
0.8
1.0
Survival time (days)
0
1000
2000
3000
4000
5000
6000
p−value: 0.0146949

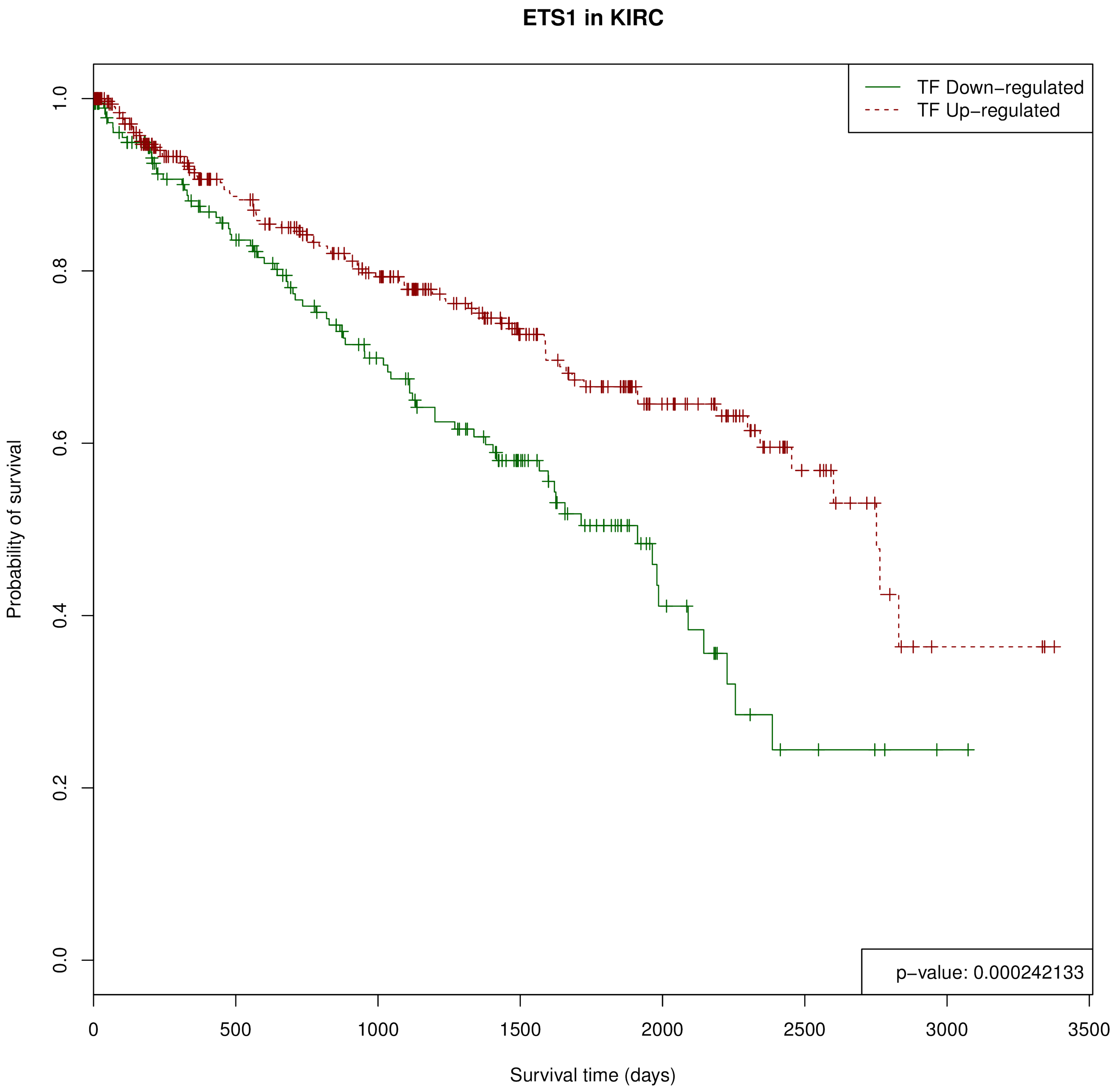
ETS1 in KIRC
TF Down−regulated
TF Up−regulated
Probability of survival
0.0
0.2
0.4
0.6
0.8
1.0
Survival time (days)
0
500
1000
1500
2000
2500
3000
3500
p−value: 0.000242133

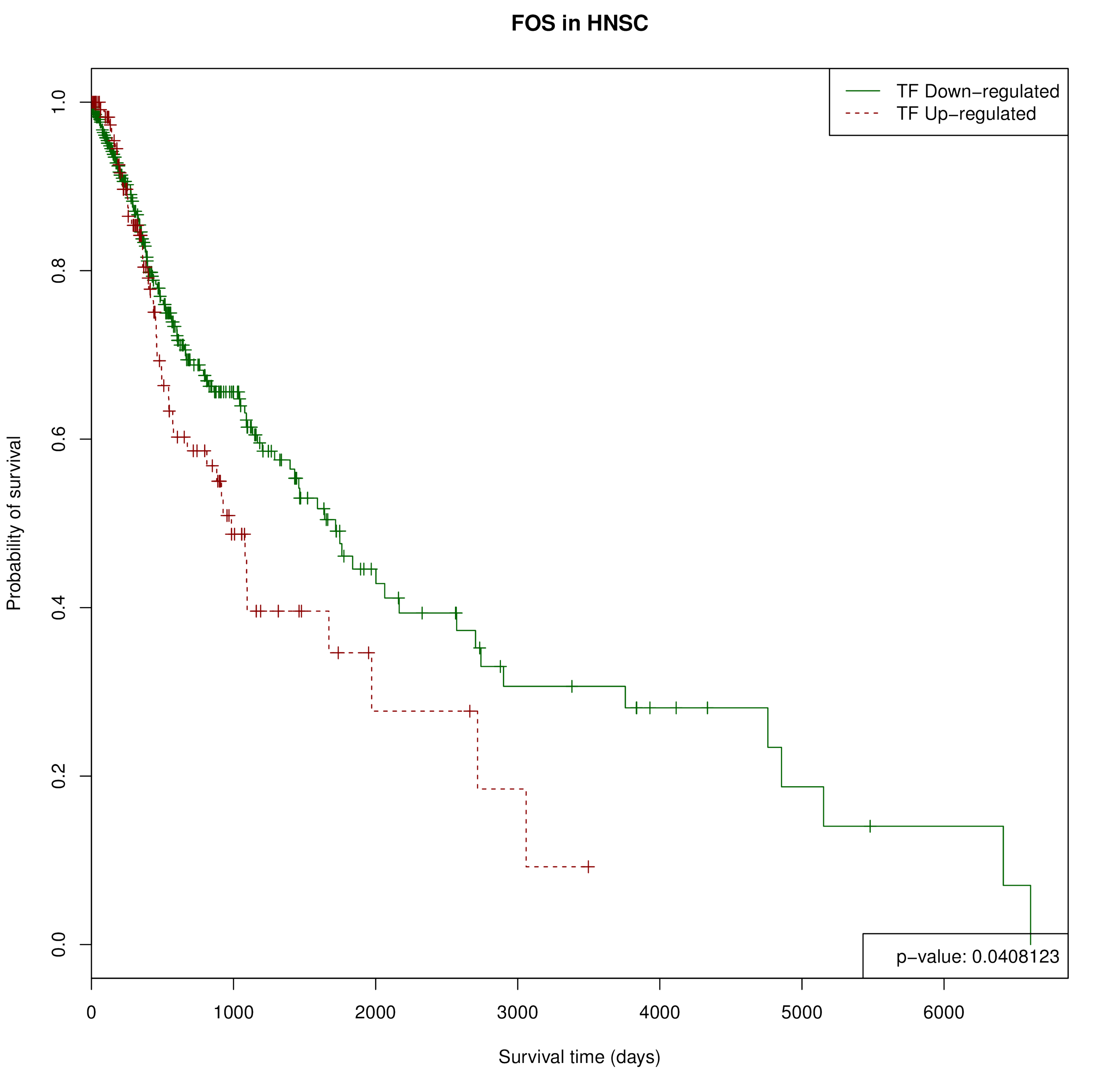

FOS in HNSC
TF Down−regulated
TF Up−regulated
Probability of survival
1.0
0.8
0.6
0.4
0.2
0.0
0
1000
2000
3000
4000
5000
6000
Survival time (days)
p−value: 0.0408123

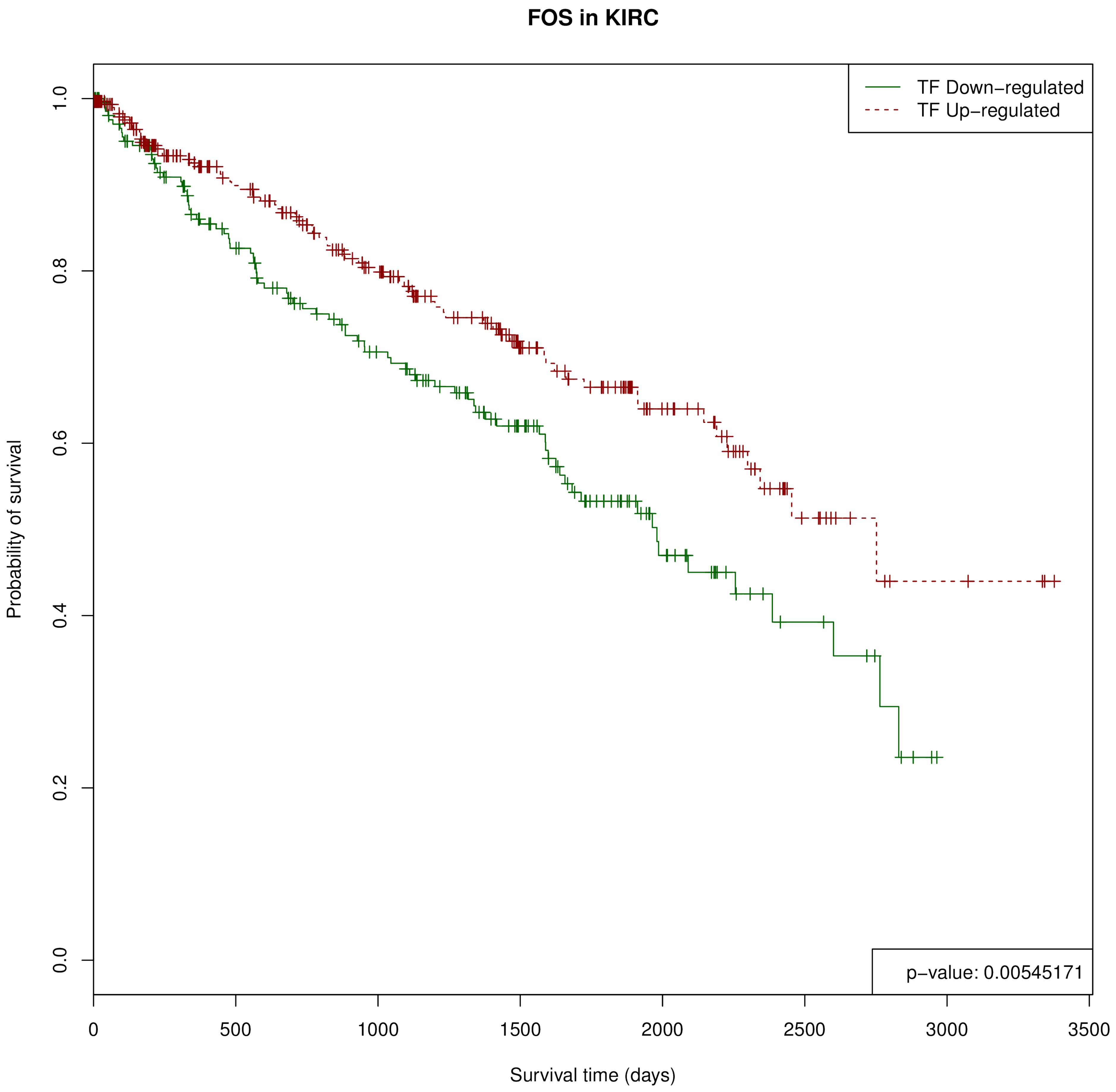
FOS in KIRC
TF Down−regulated
TF Up−regulated
p−value: 0.00545171
Probability of survival
Survival time (days)
0.0
0.2
0.4
0.6
0.8
1.0
0
500
1000
1500
2000
2500
3000
3500

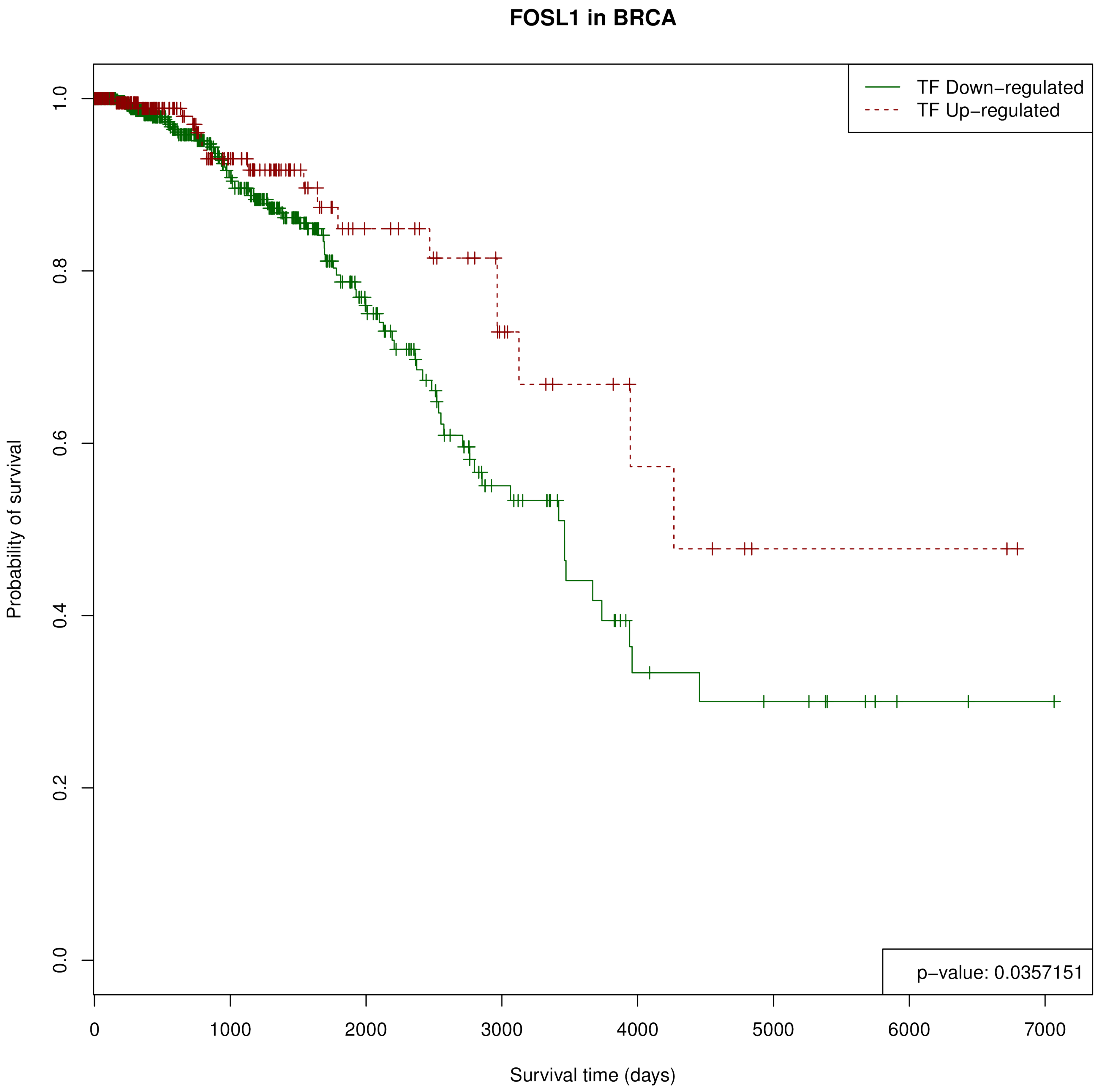
FOSL1 in BRCA
TF Down−regulated
TF Up−regulated
Probability of survival
0.0
0.2
0.4
0.6
0.8
1.0
Survival time (days)
0
1000
2000
3000
4000
5000
6000
7000
p−value: 0.0357151

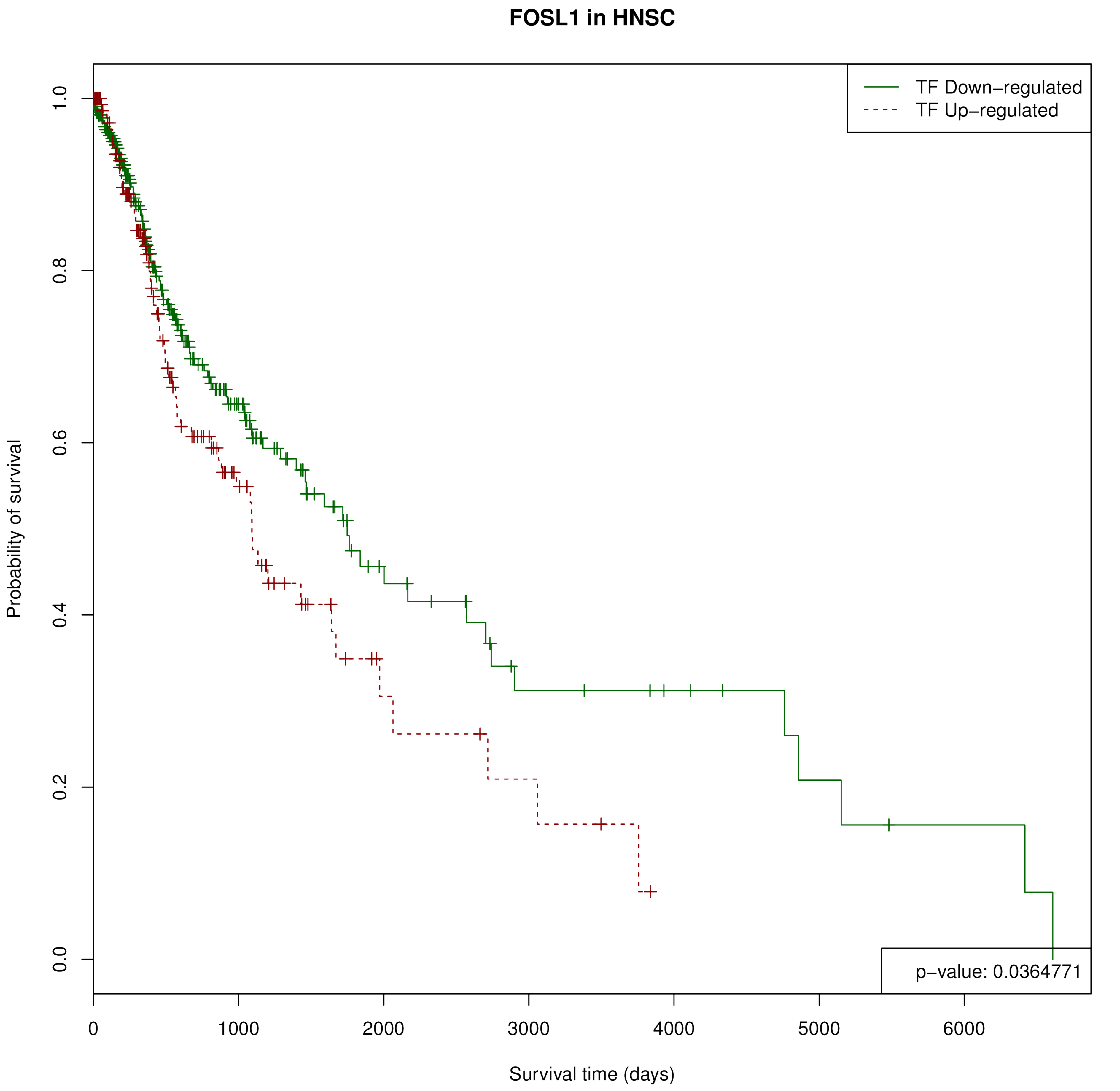
FOSL1 in HNSC
TF Down−regulated
TF Up−regulated
p−value: 0.0364771
Probability of survival
Survival time (days)
0.0
0.2
0.4
0.6
0.8
1.0
0
1000
2000
3000
4000
5000
6000

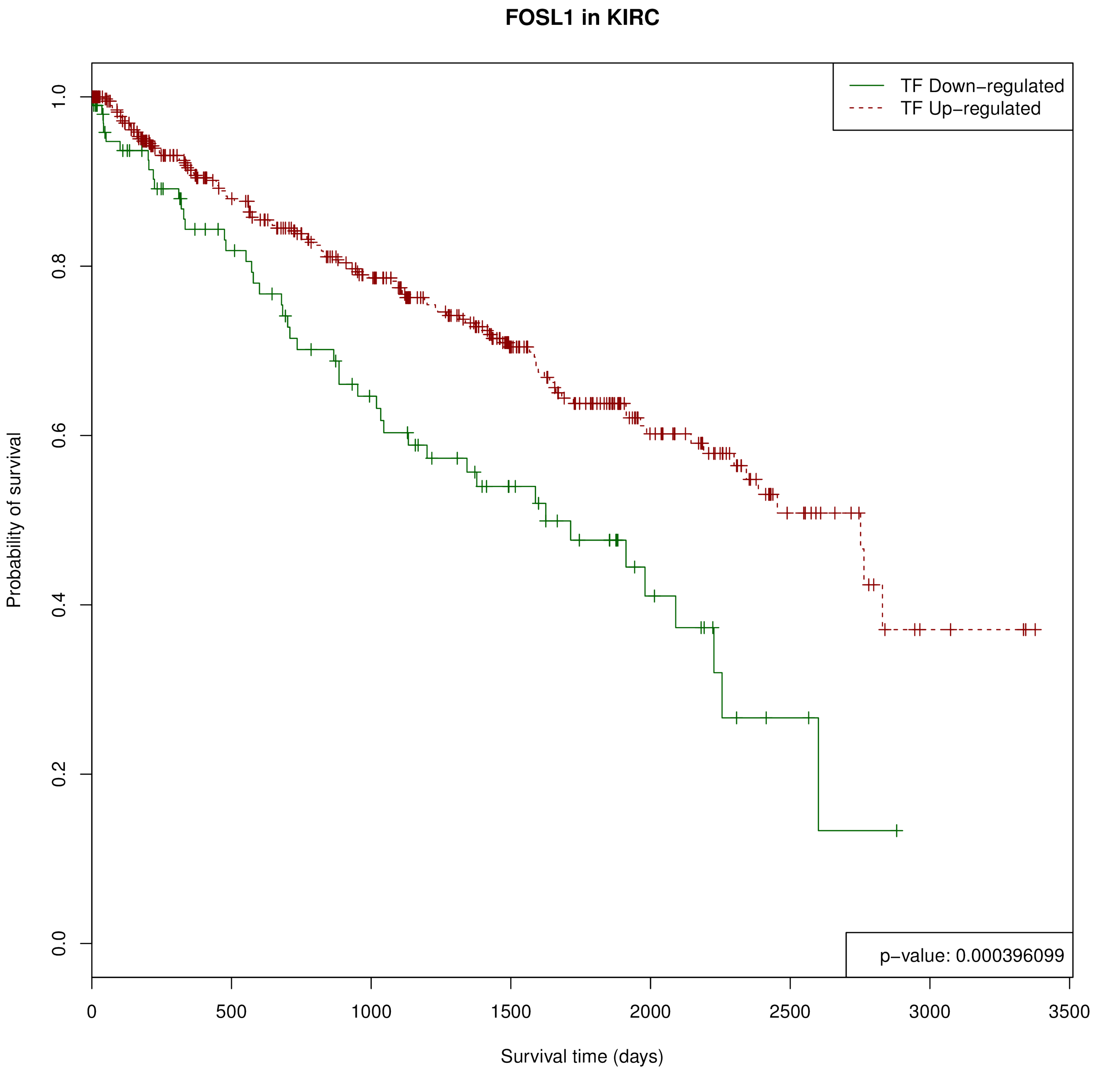
FOSL1 in KIRC
TF Down−regulated
TF Up−regulated
p−value: 0.000396099
Probability of survival
Survival time (days)
0.0
0.2
0.4
0.6
0.8
1.0
0
500
1000
1500
2000
2500
3000
3500

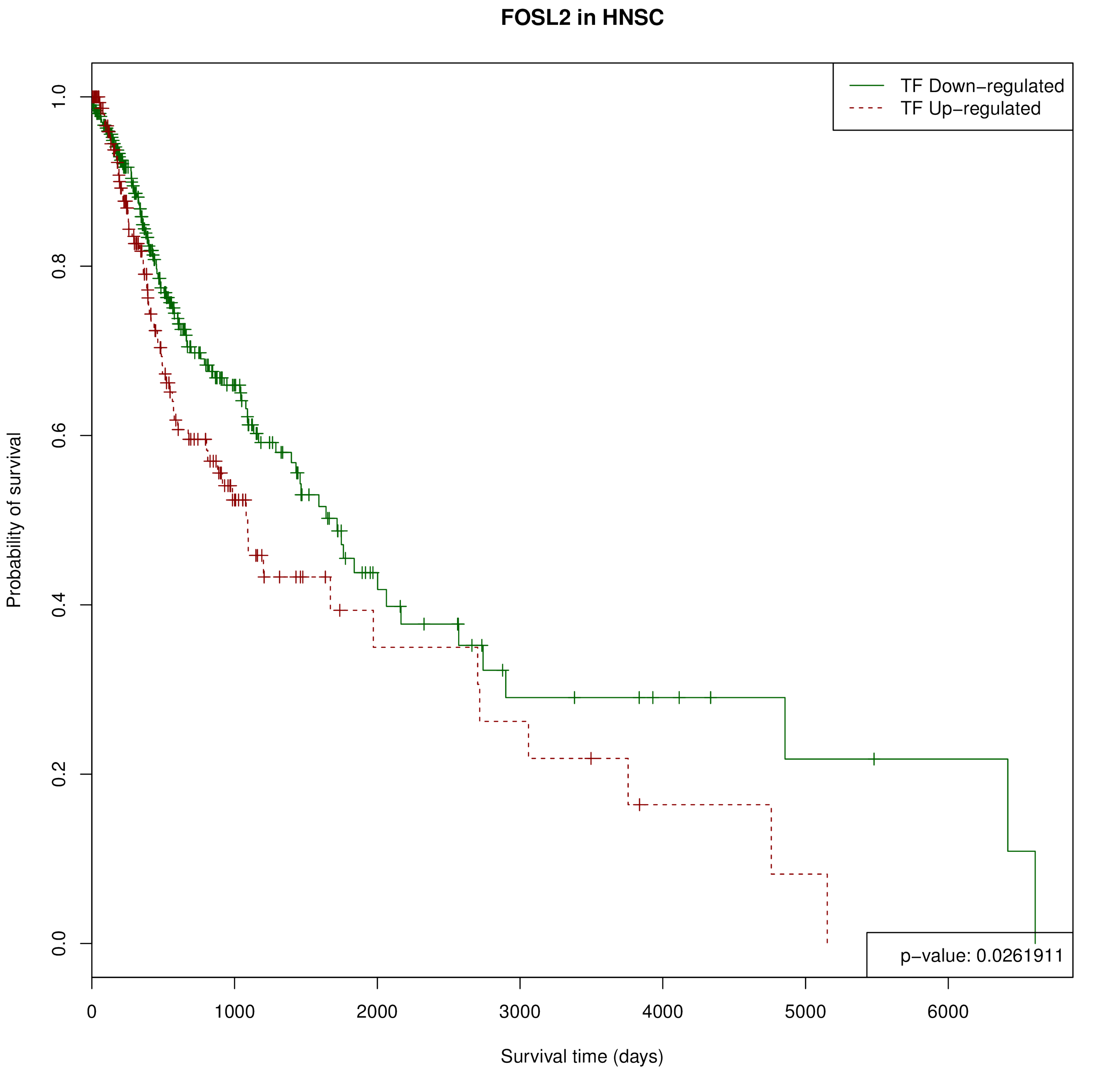

FOSL2 in HNSC
TF Down−regulated
TF Up−regulated
Probability of survival
0.0
0.2
0.4
0.6
0.8
1.0
Survival time (days)
0
1000
2000
3000
4000
5000
6000
p−value: 0.0261911

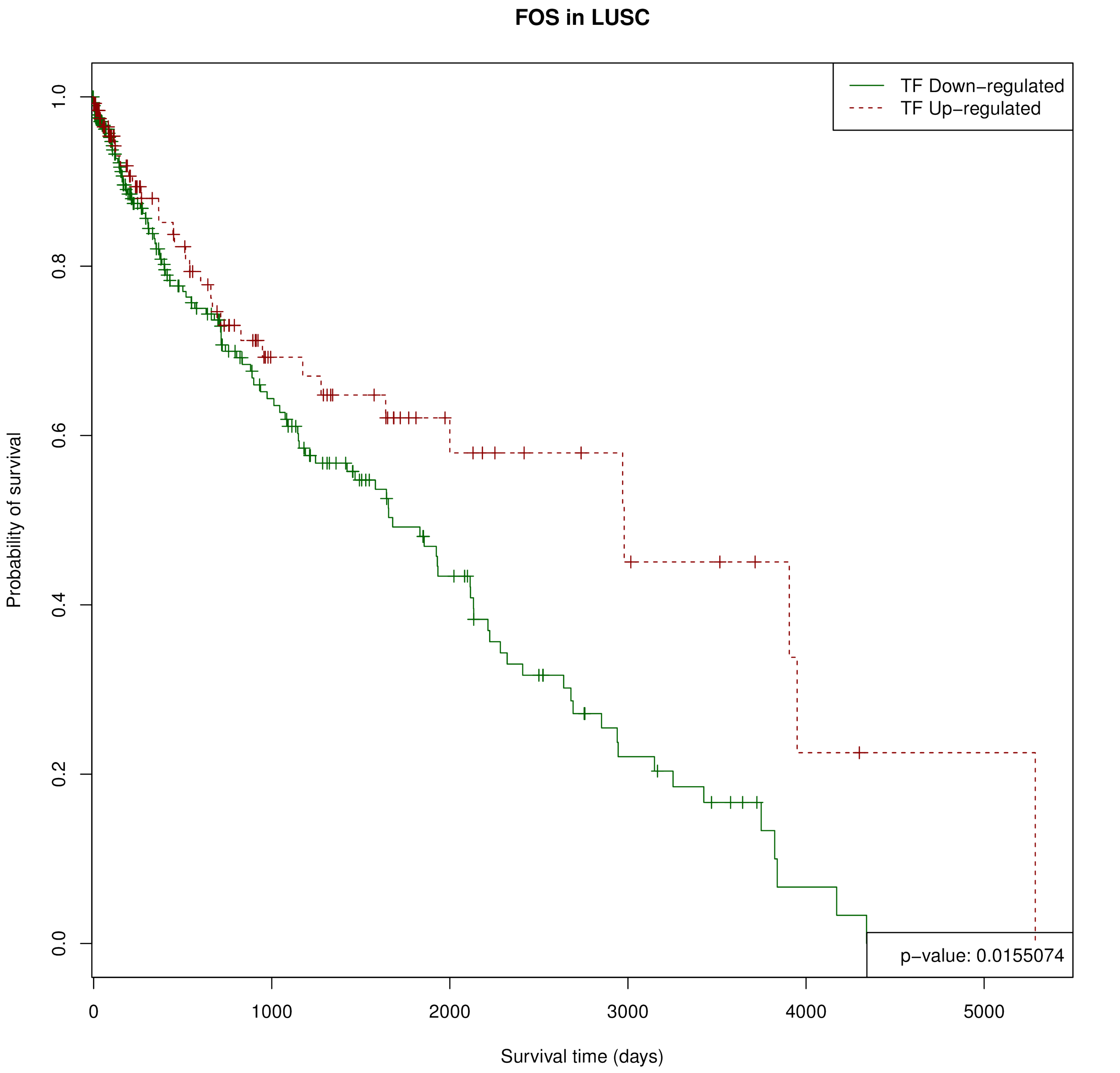

FOS in LUSC
TF Down−regulated
TF Up−regulated
Probability of survival
0.0
0.2
0.4
0.6
0.8
1.0
Survival time (days)
0
1000
2000
3000
4000
5000
p−value: 0.0155074

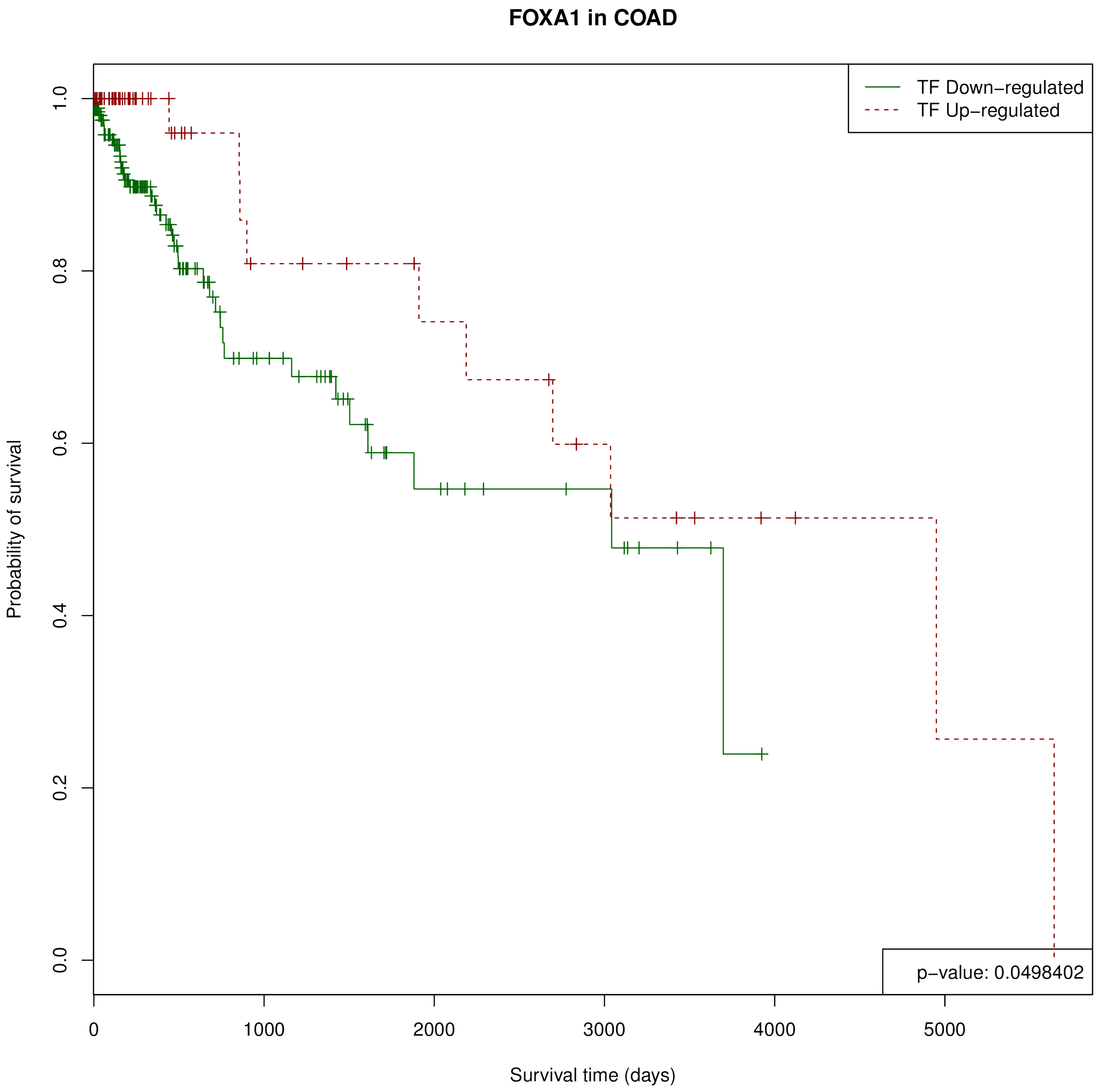

FOXA1 in COAD
TF Down−regulated
TF Up−regulated
Probability of survival
0.0
0.2
0.4
0.6
0.8
1.0
0
1000
2000
3000
4000
5000
Survival time (days)
p−value: 0.0498402

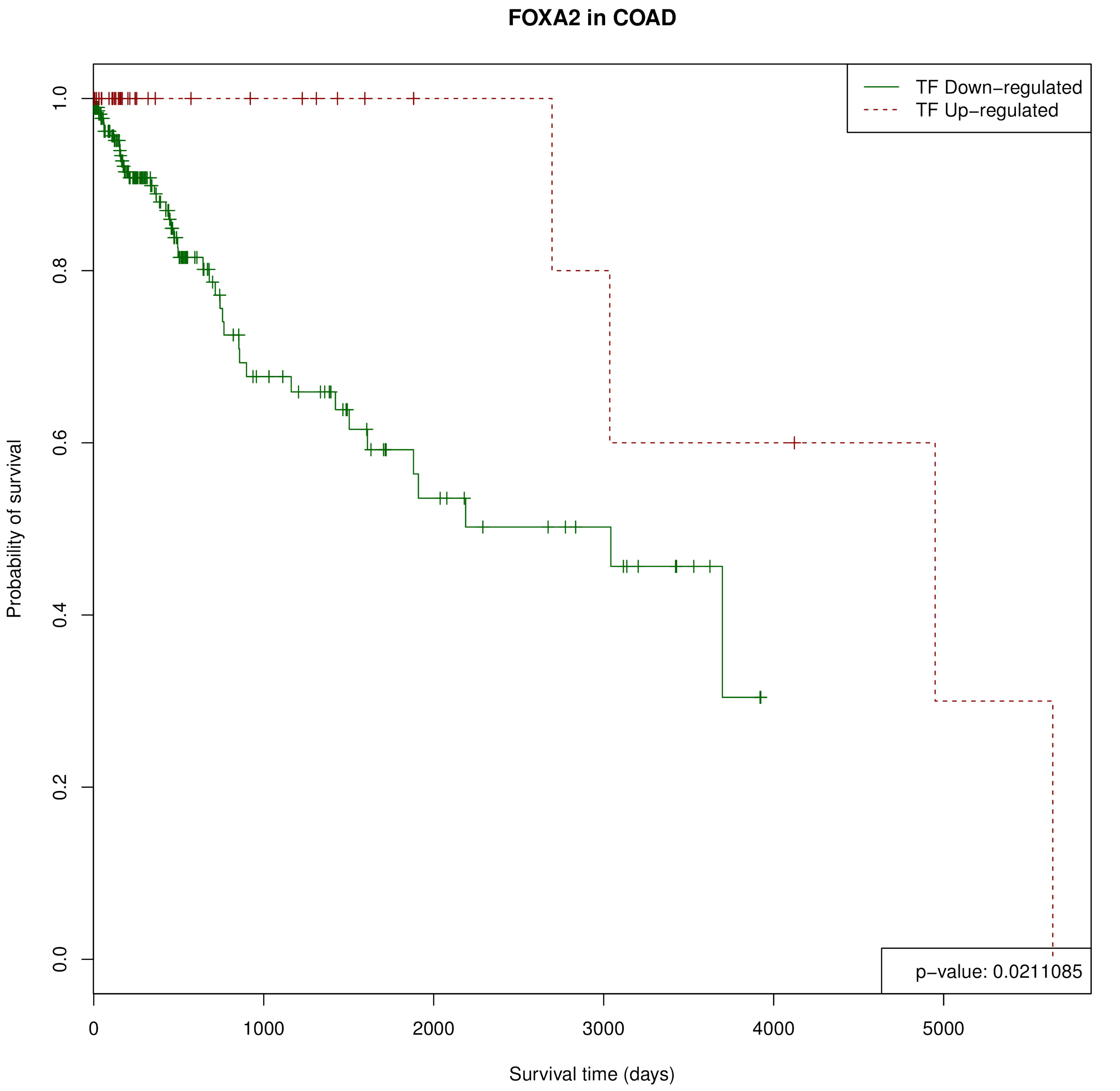
FOXA2 in COAD
TF Down−regulated
TF Up−regulated
Probability of survival
0.0
0.2
0.4
0.6
0.8
1.0
0
1000
2000
3000
4000
5000
Survival time (days)
p−value: 0.0211085

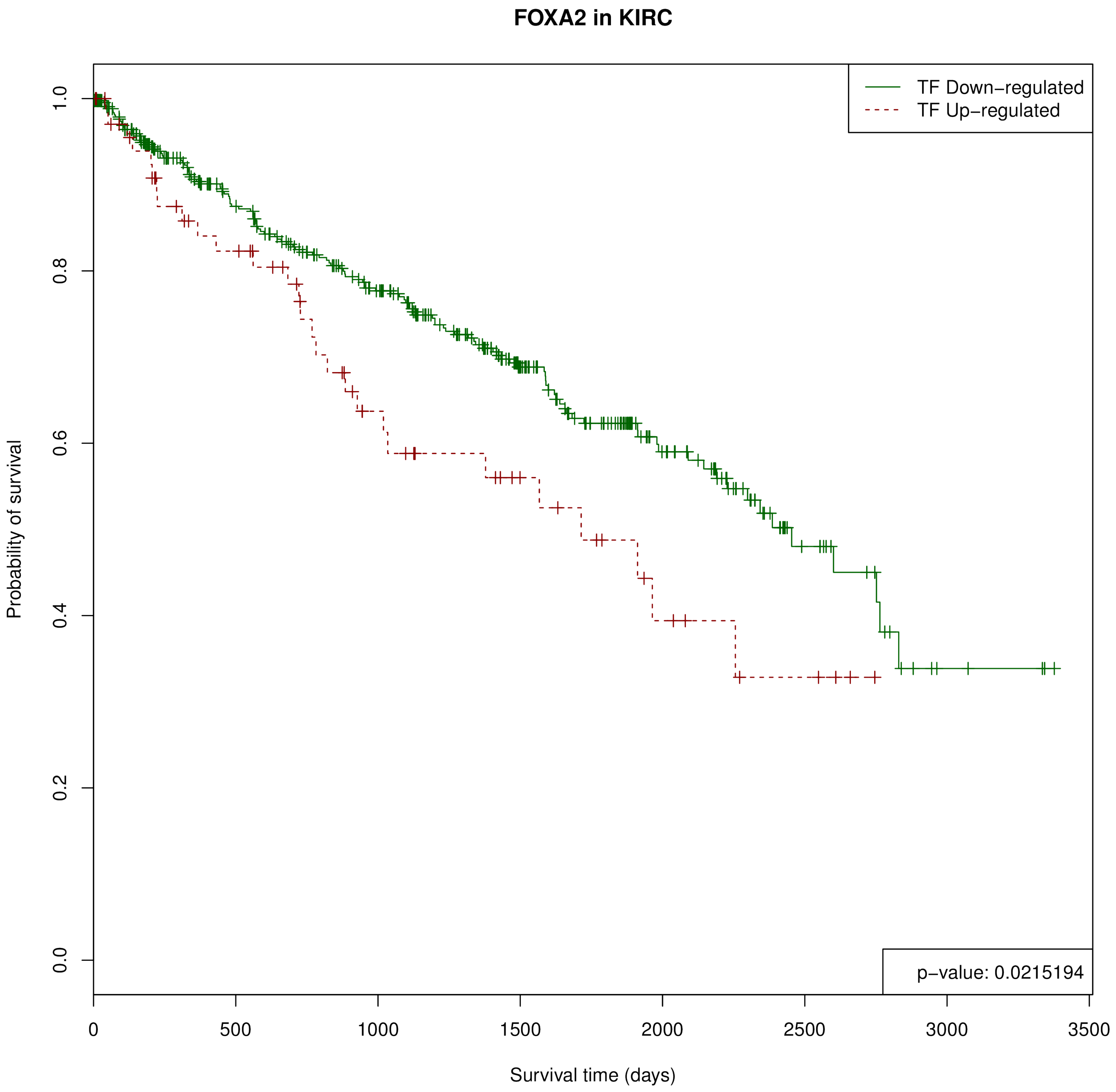

FOXA2 in KIRC
TF Down−regulated
TF Up−regulated
p−value: 0.0215194
Probability of survival
Survival time (days)
0.0
0.2
0.4
0.6
0.8
1.0
0
500
1000
1500
2000
2500
3000
3500

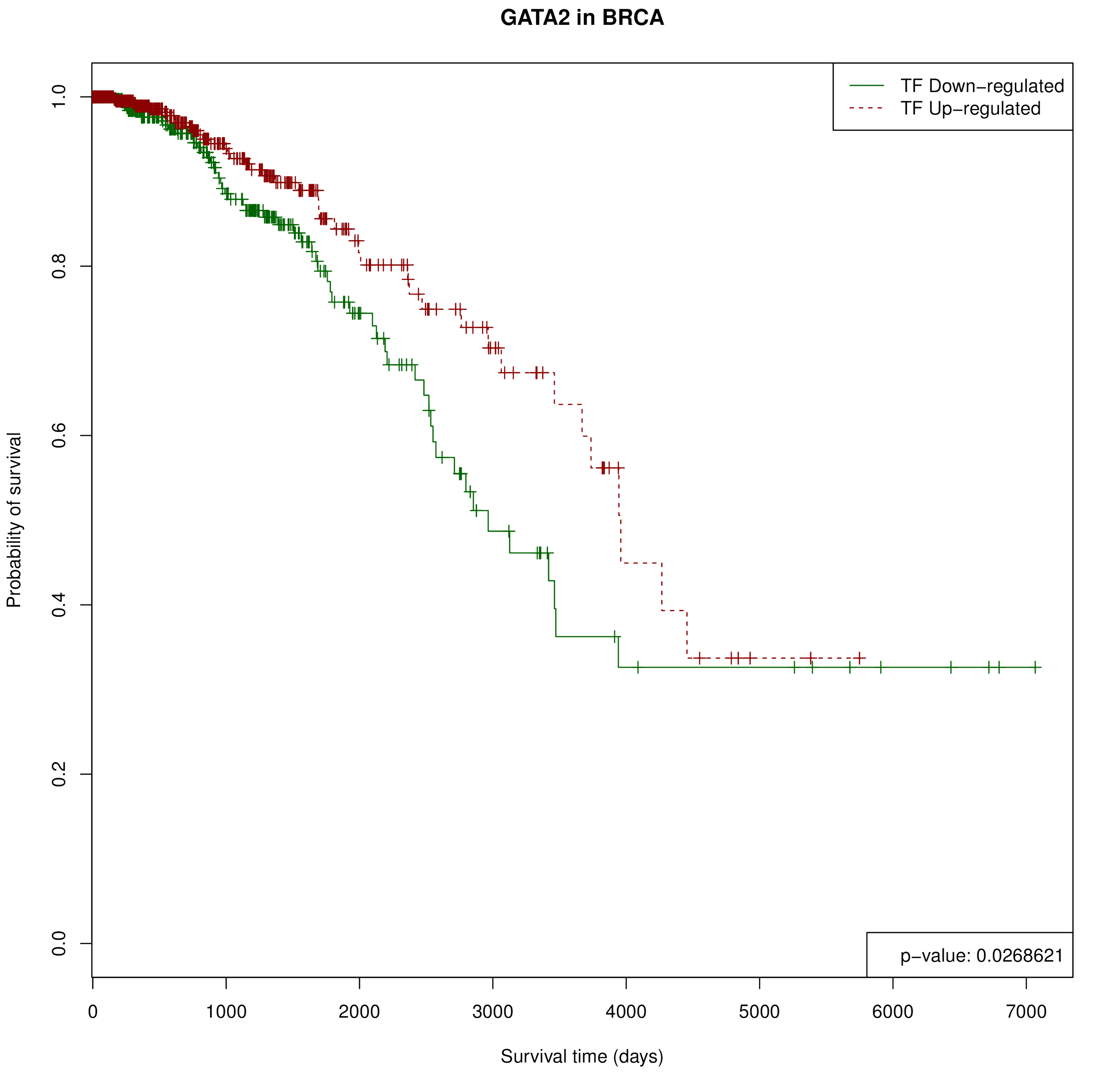
GATA2 in BRCA
TF Down−regulated
TF Up−regulated
p−value: 0.0268621
Probability of survival
Survival time (days)
0.0
0.2
0.4
0.6
0.8
1.0
0
1000
2000
3000
4000
5000
6000
7000

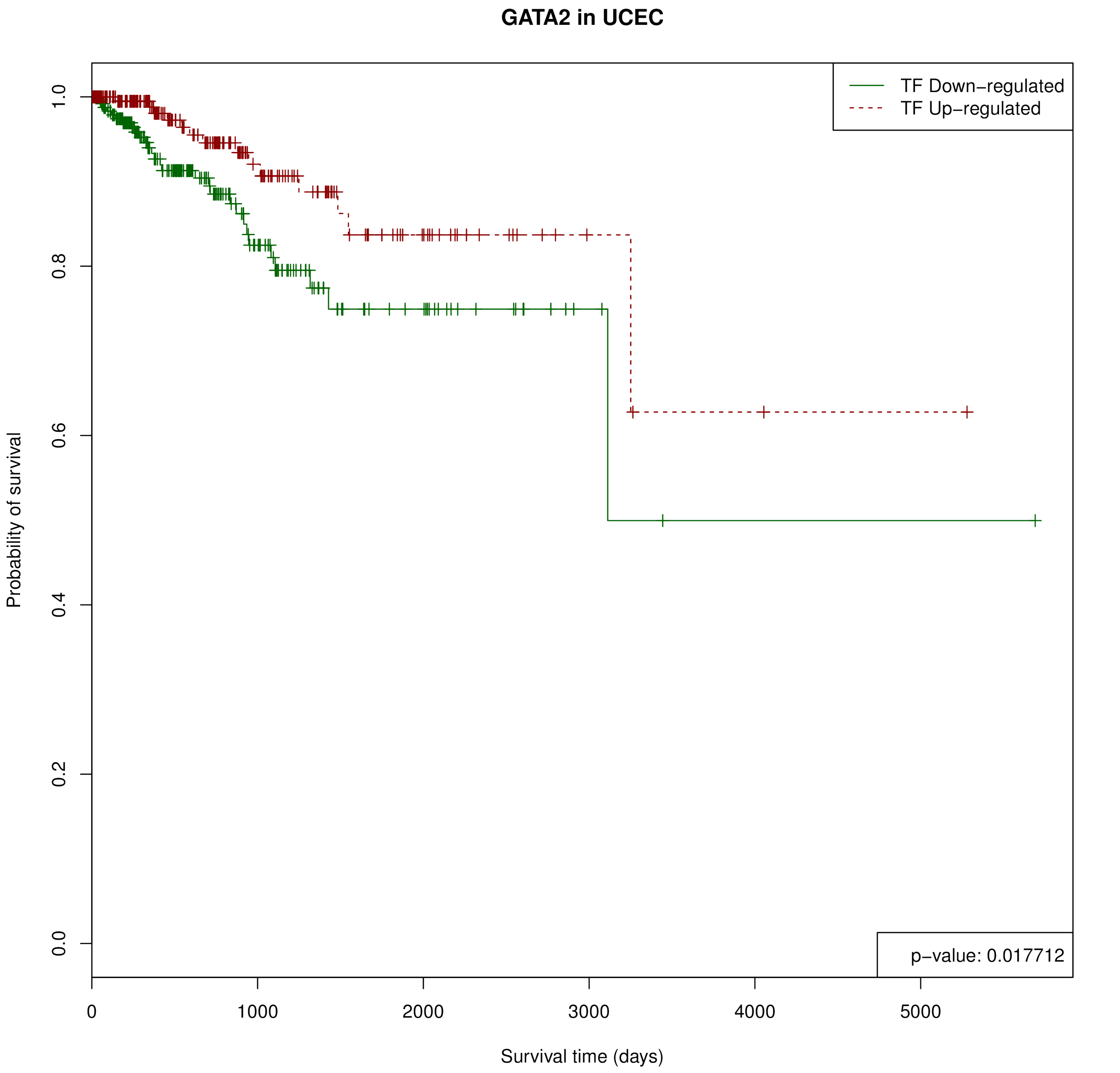

GATA2 in UCEC
TF Down−regulated
TF Up−regulated
Probability of survival
0.0
0.2
0.4
0.6
0.8
1.0
0
1000
2000
3000
4000
5000
Survival time (days)
p−value: 0.017712

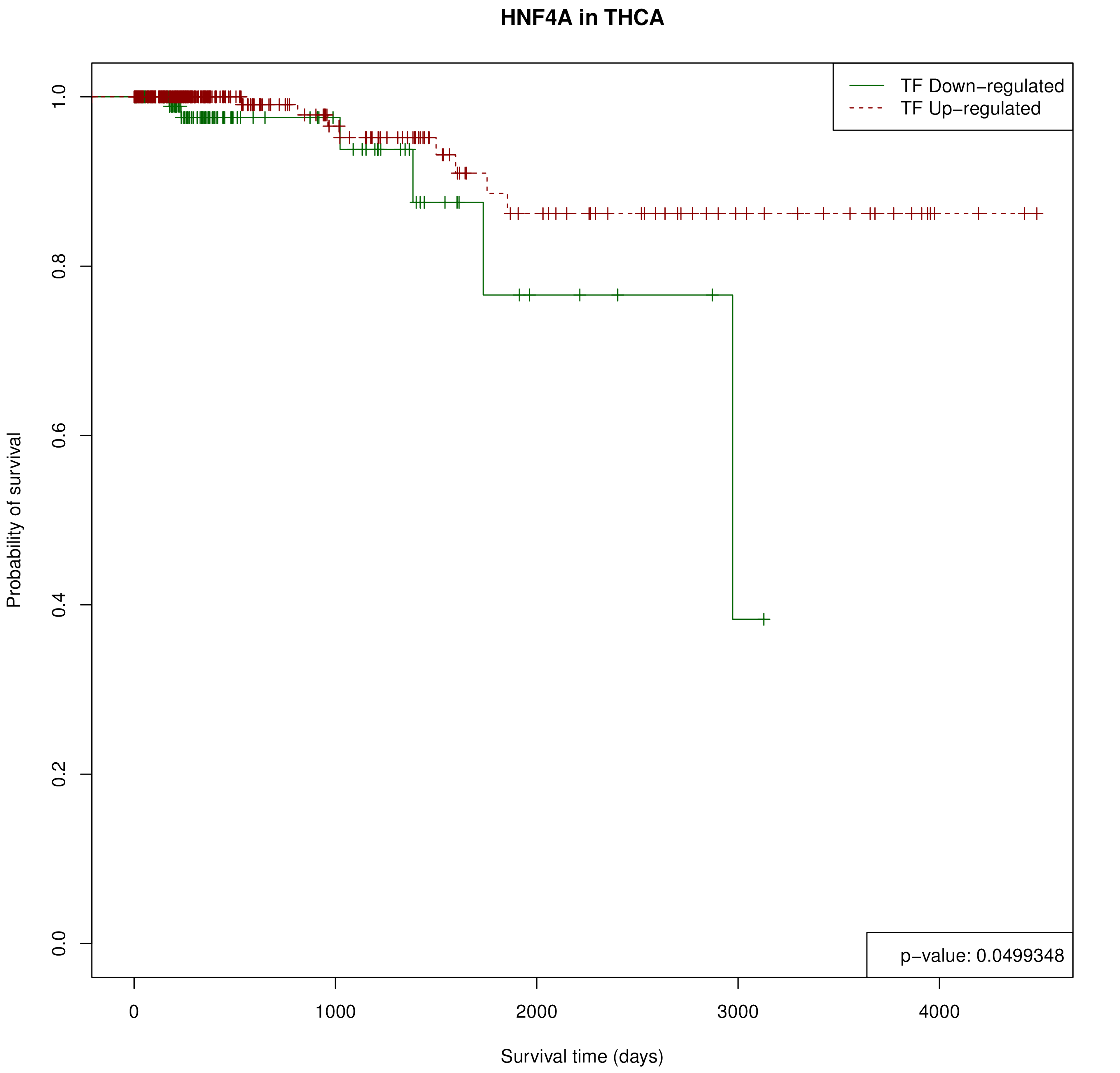
HNF4A in THCA
TF Down−regulated
TF Up−regulated
Probability of survival
1.0
0.8
0.6
0.4
0.2
0.0
0
1000
2000
3000
4000
Survival time (days)
p−value: 0.0499348

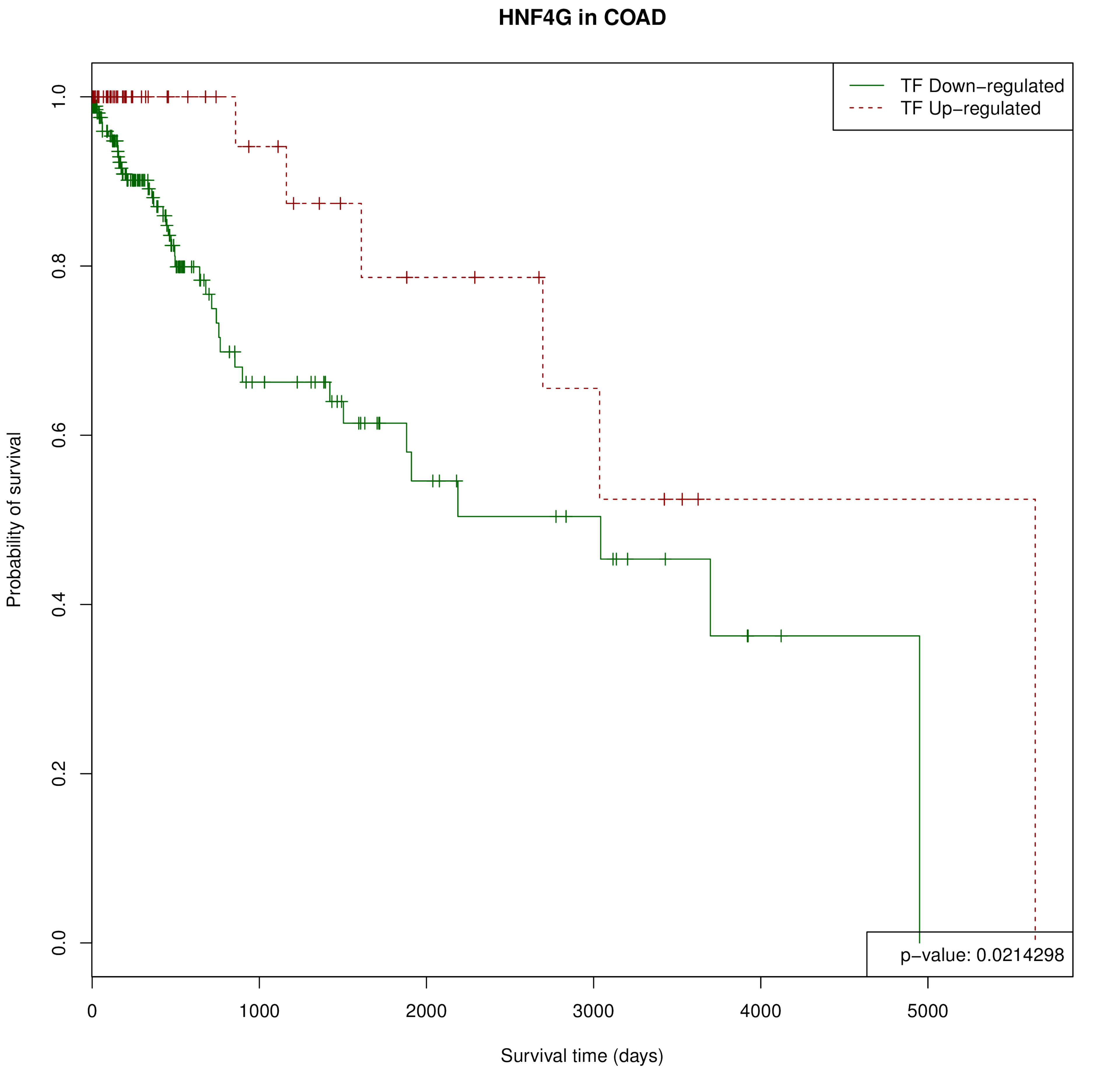
HNF4G in COAD
TF Down−regulated
TF Up−regulated
1.0
0.8
0.6
0.4
0.2
0.0
Probability of survival
0
1000
2000
3000
4000
5000
Survival time (days)
p−value: 0.0214298

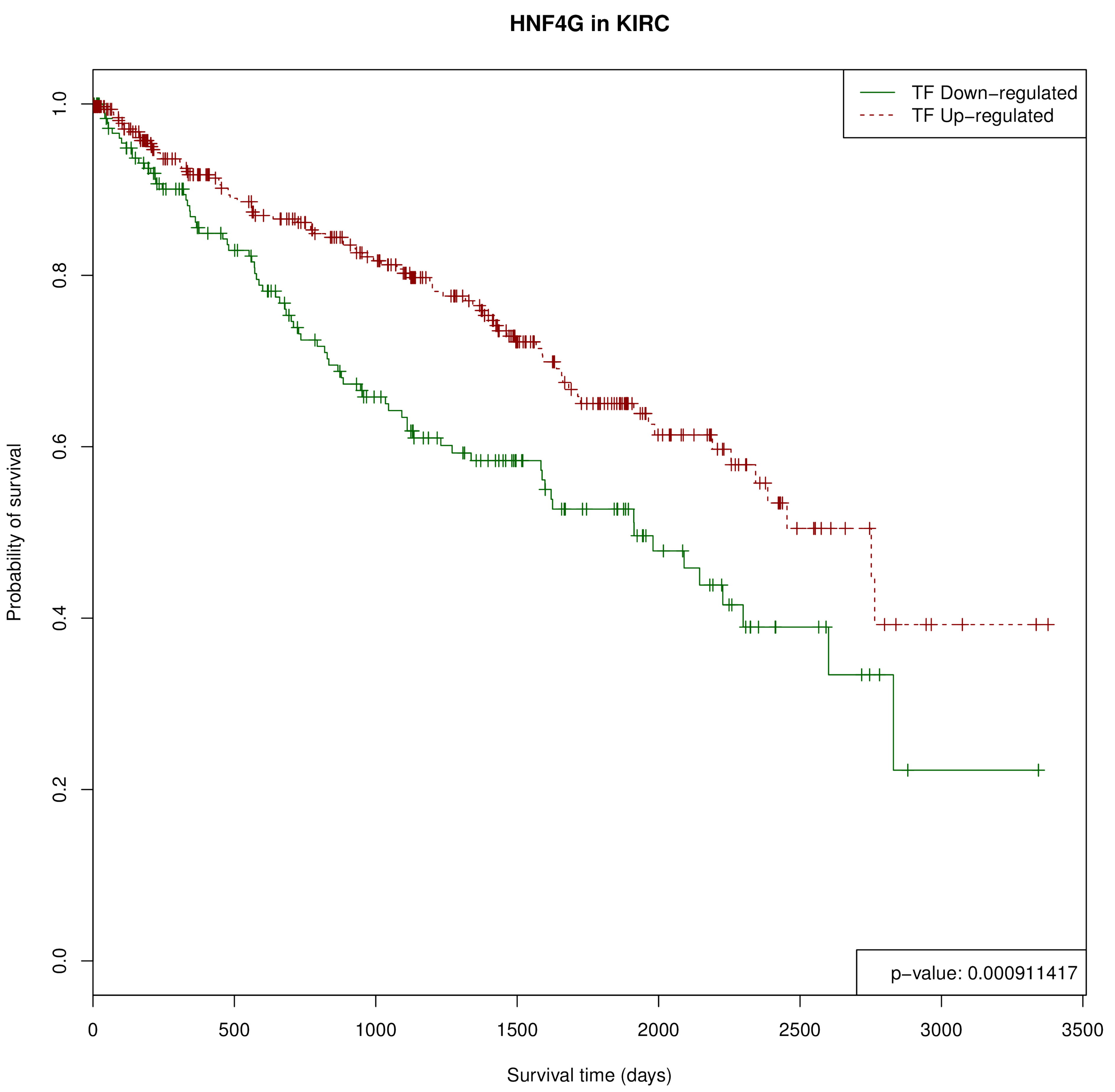

HNF4G in KIRC
TF Down−regulated
TF Up−regulated
p−value: 0.000911417
Probability of survival
Survival time (days)
0.0
0.2
0.4
0.6
0.8
1.0
0
500
1000
1500
2000
2500
3000
3500

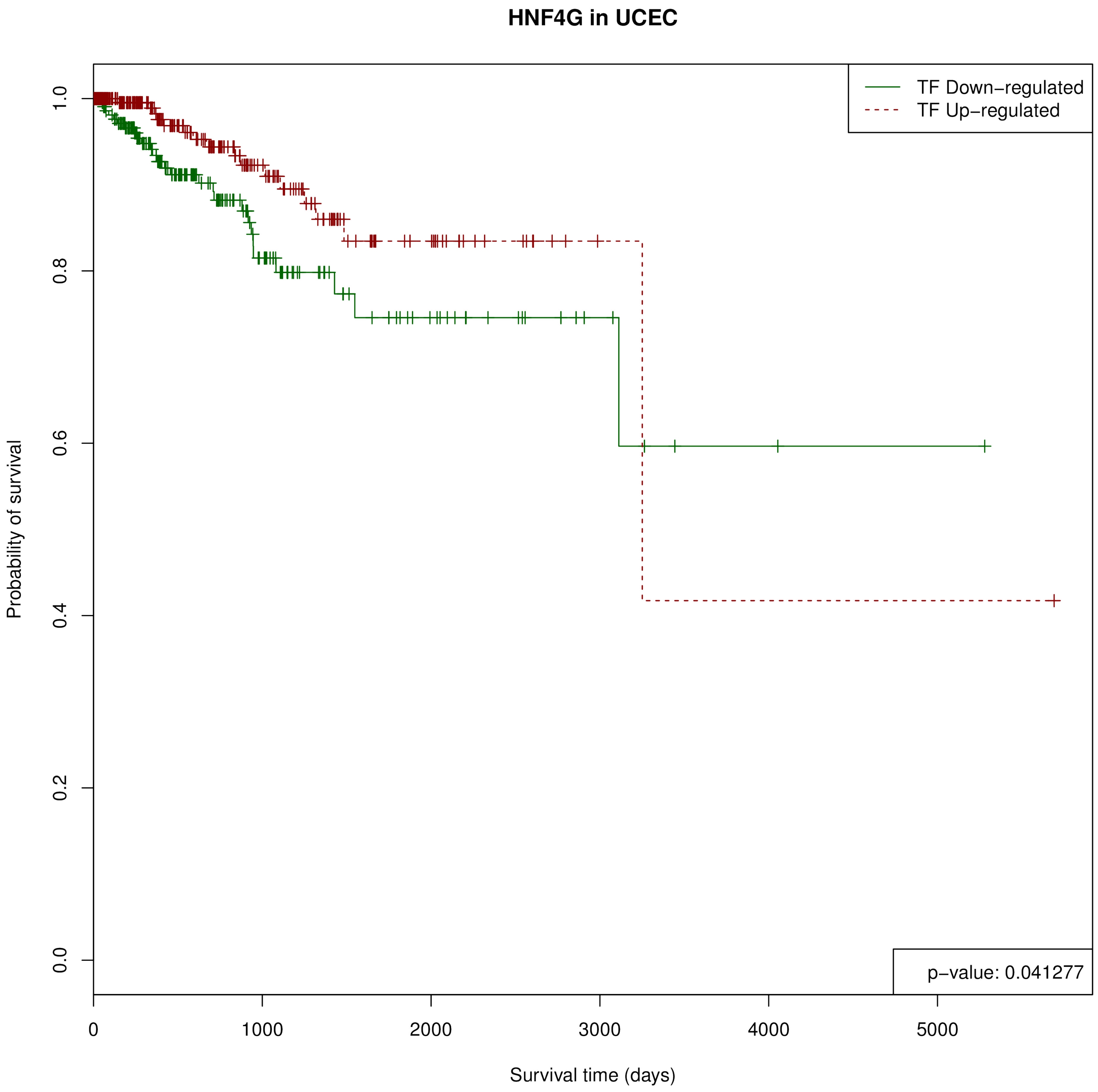
HNF4G in UCEC
TF Down−regulated
TF Up−regulated
Probability of survival
0.0
0.2
0.4
0.6
0.8
1.0
0
1000
2000
3000
4000
5000
Survival time (days)
p−value: 0.041277

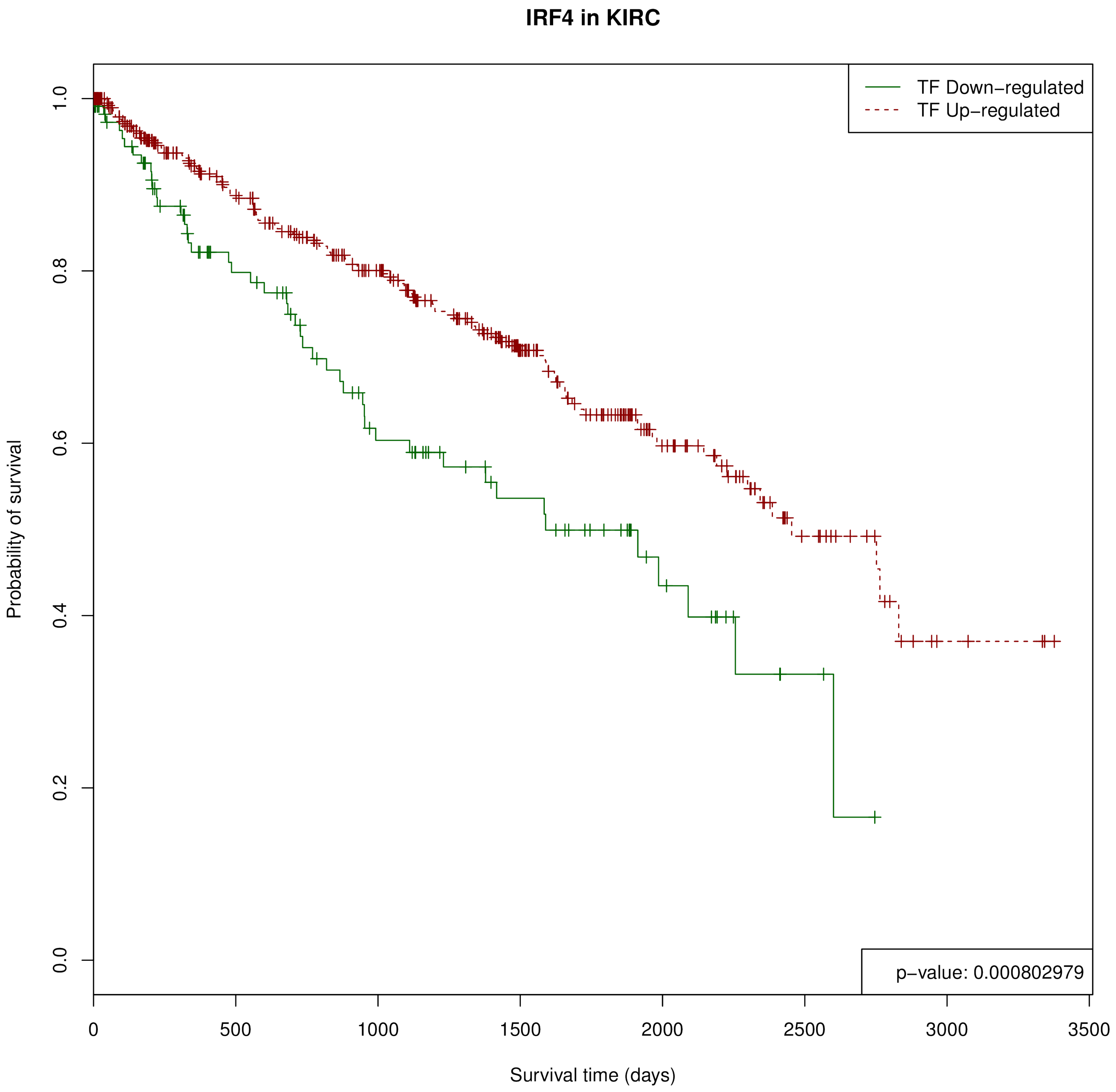
IRF4 in KIRC
TF Down−regulated
TF Up−regulated
Probability of survival
1.0
0.8
0.6
0.4
0.2
0.0
0
500
1000
1500
2000
2500
3000
3500
Survival time (days)
p−value: 0.000802979

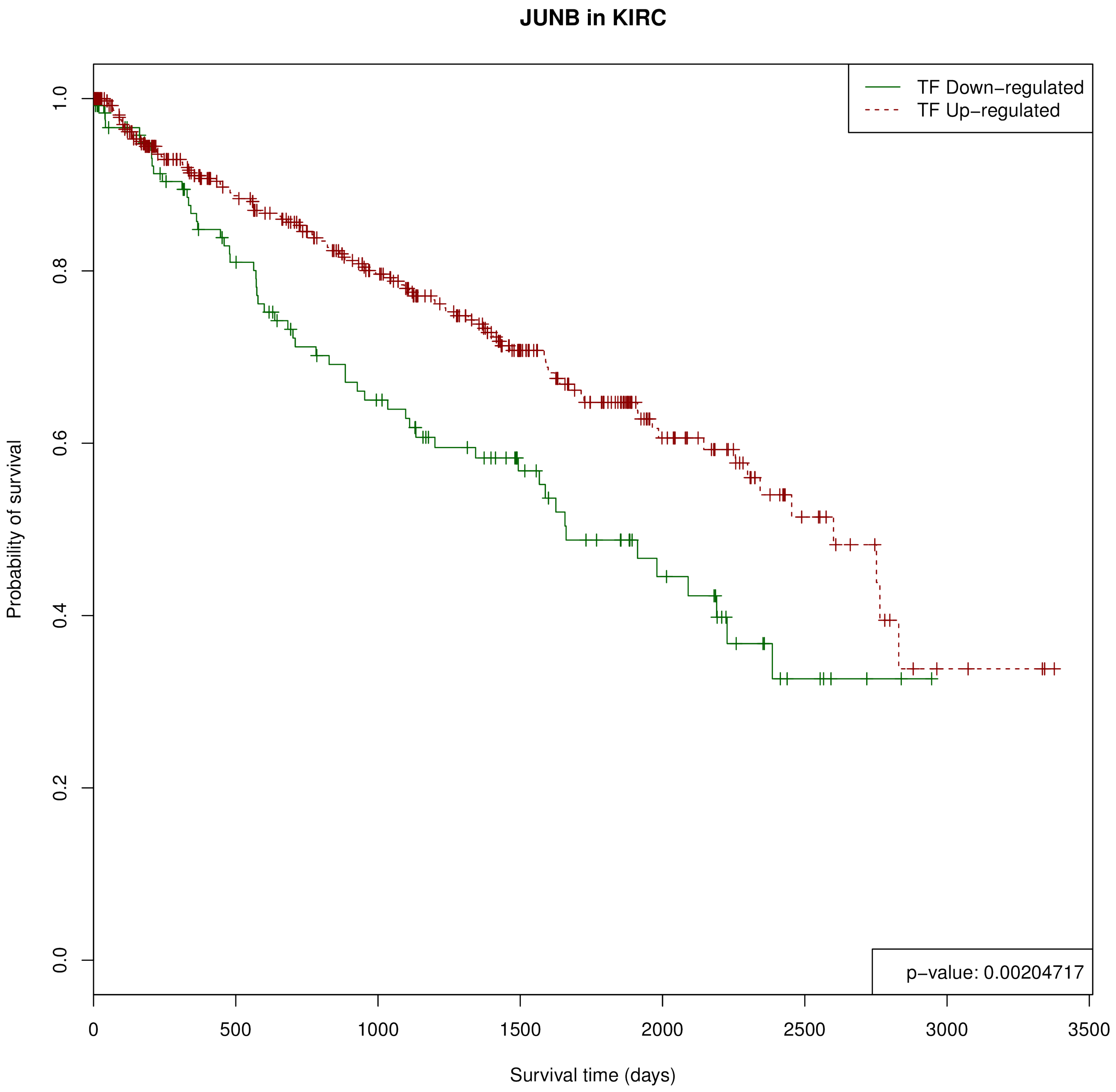

JUNB in KIRC
TF Down−regulated
TF Up−regulated
p−value: 0.00204717
Probability of survival
Survival time (days)
0.0
0.2
0.4
0.6
0.8
1.0
0
500
1000
1500
2000
2500
3000
3500

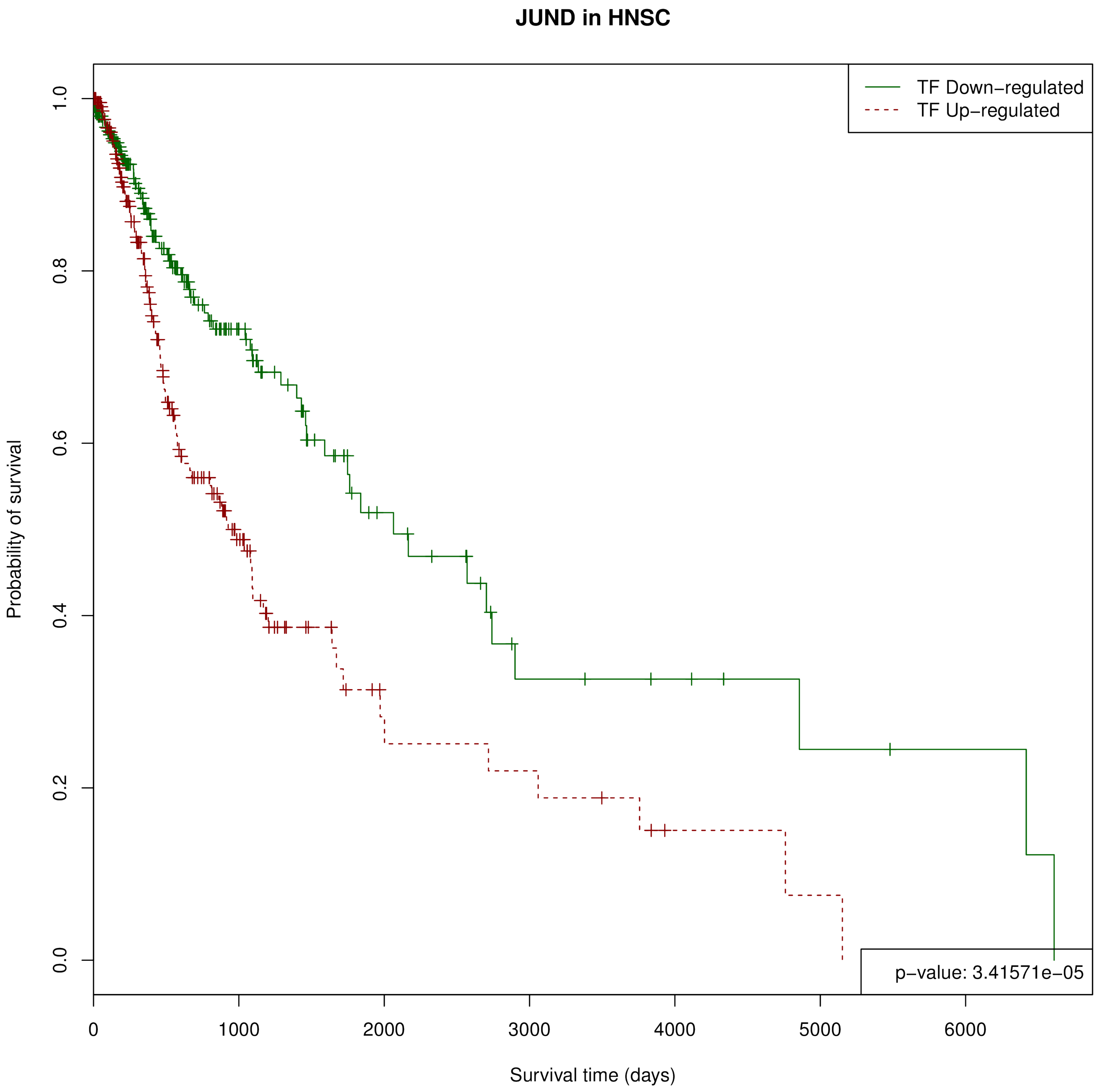
JUND in HNSC
TF Down−regulated
TF Up−regulated
Probability of survival
1.0
0.8
0.6
0.4
0.2
0.0
0
1000
2000
3000
4000
5000
6000
Survival time (days)
p−value: 3.41571e−05

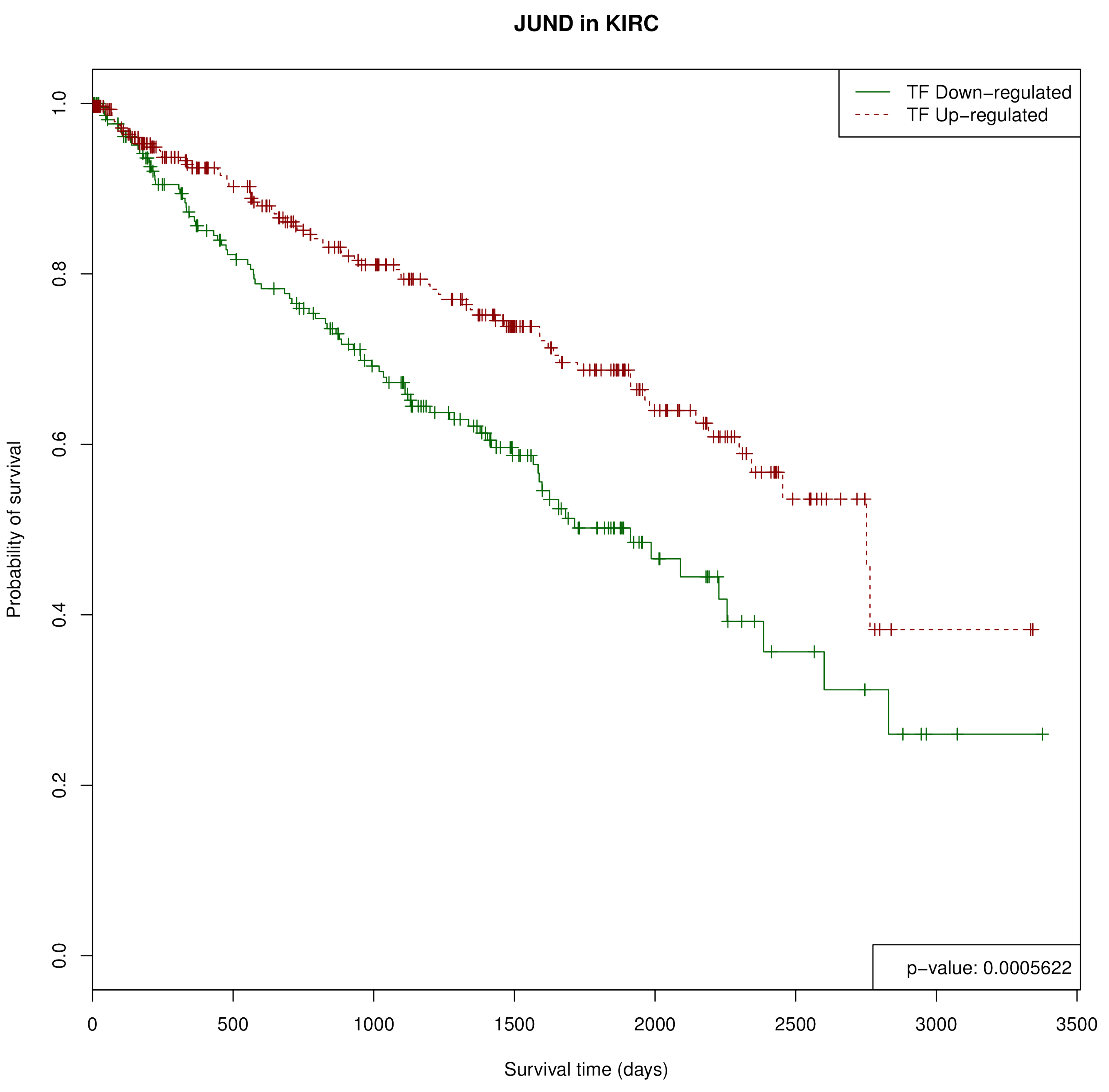

JUND in KIRC
TF Down−regulated
TF Up−regulated
p−value: 0.0005622
Probability of survival
Survival time (days)
0.0
0.2
0.4
0.6
0.8
1.0
0
500
1000
1500
2000
2500
3000
3500

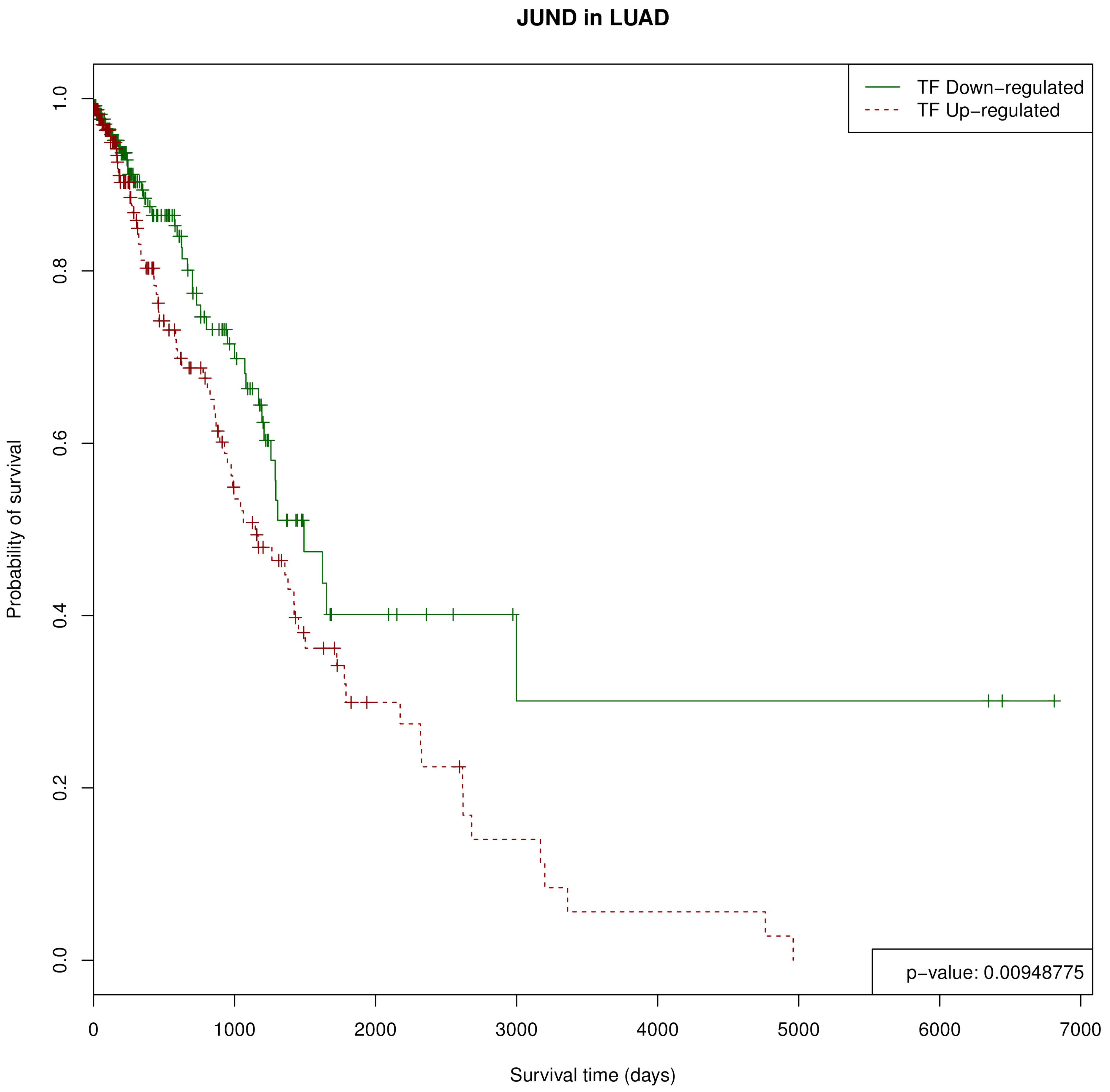
JUND in LUAD
TF Down−regulated
TF Up−regulated
p−value: 0.00948775
Probability of survival
Survival time (days)
0.0
0.2
0.4
0.6
0.8
1.0
0
1000
2000
3000
4000
5000
6000
7000

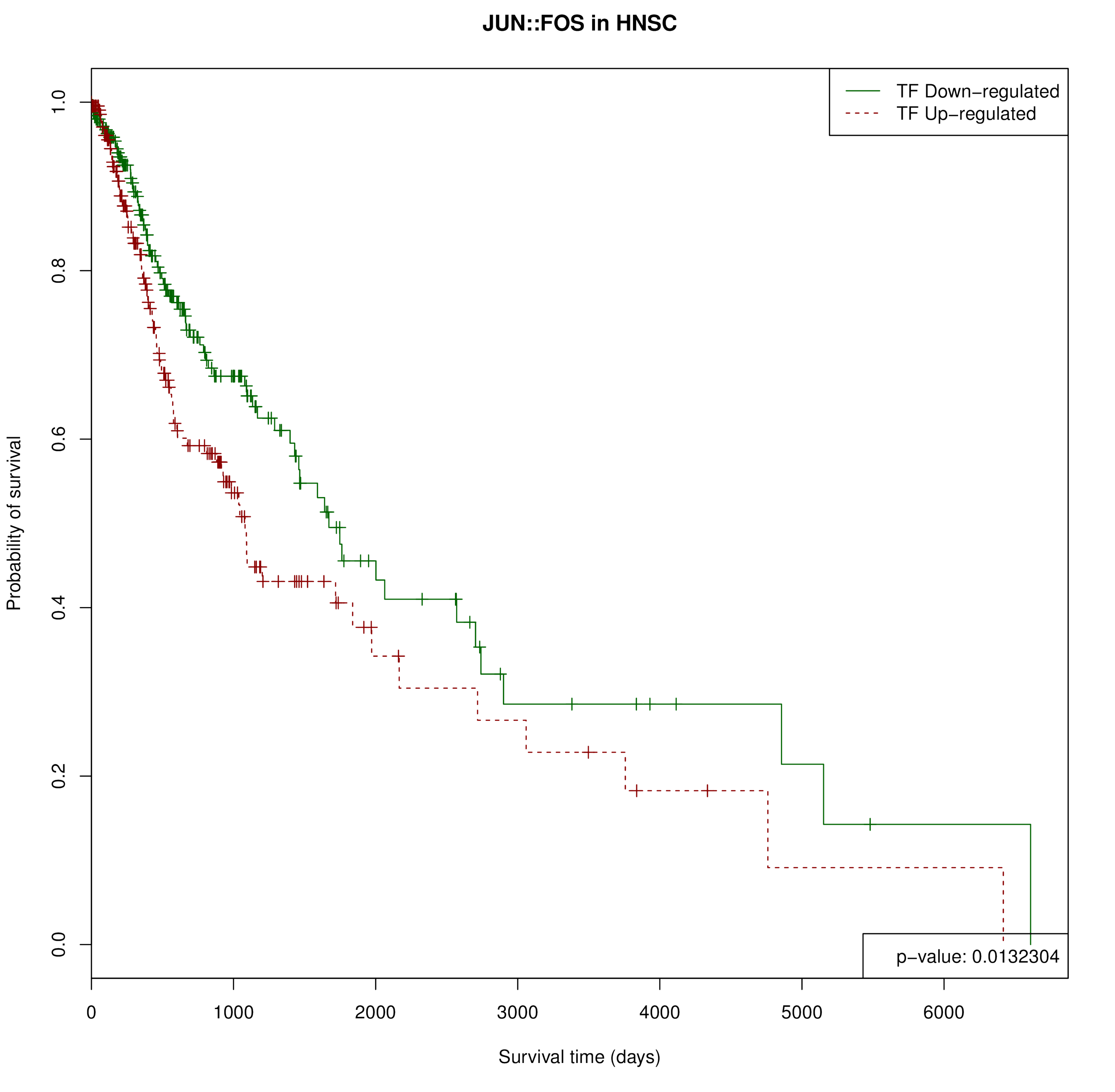

JUN::FOS in HNSC
TF Down−regulated
TF Up−regulated
p−value: 0.0132304
Probability of survival
Survival time (days)
0.0
0.2
0.4
0.6
0.8
1.0
0
1000
2000
3000
4000
5000
6000

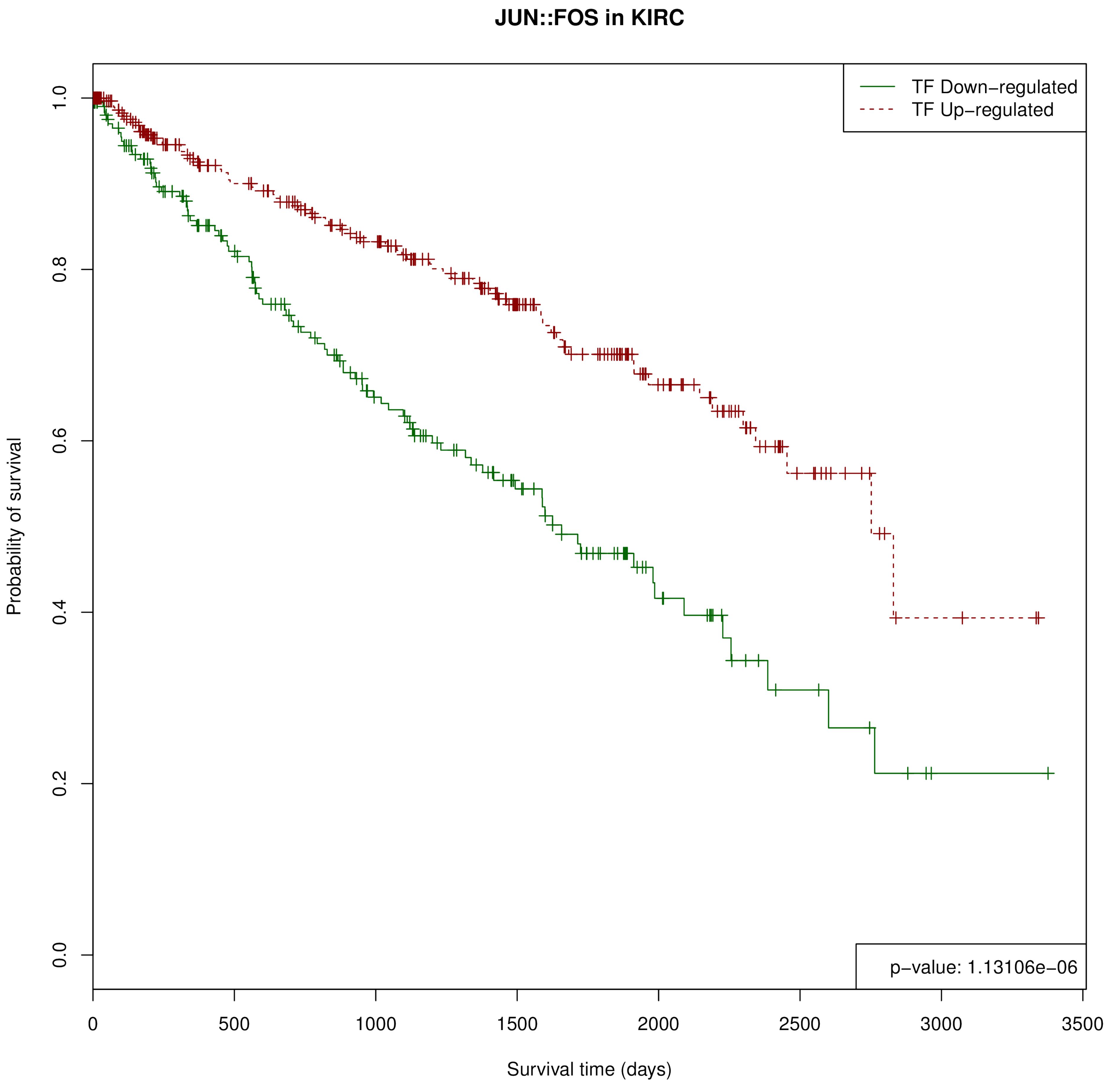

JUN::FOS in KIRC
TF Down−regulated
TF Up−regulated
p−value: 1.13106e−06
Probability of survival
Survival time (days)
0.0
0.2
0.4
0.6
0.8
1.0
0
500
1000
1500
2000
2500
3000
3500

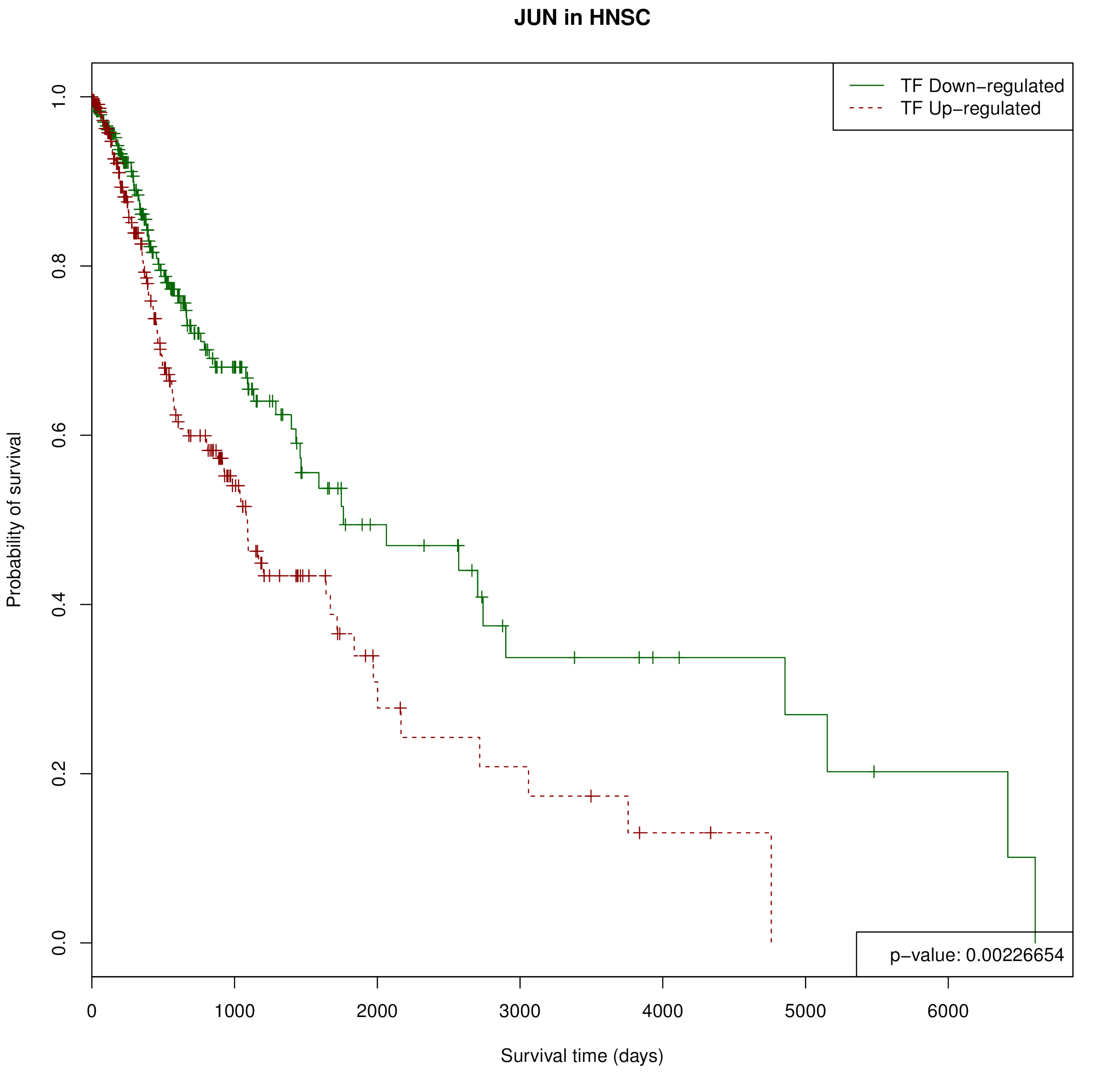

JUN in HNSC
TF Down−regulated
TF Up−regulated
p−value: 0.00226654
Probability of survival
Survival time (days)
0.0
0.2
0.4
0.6
0.8
1.0
0
1000
2000
3000
4000
5000
6000

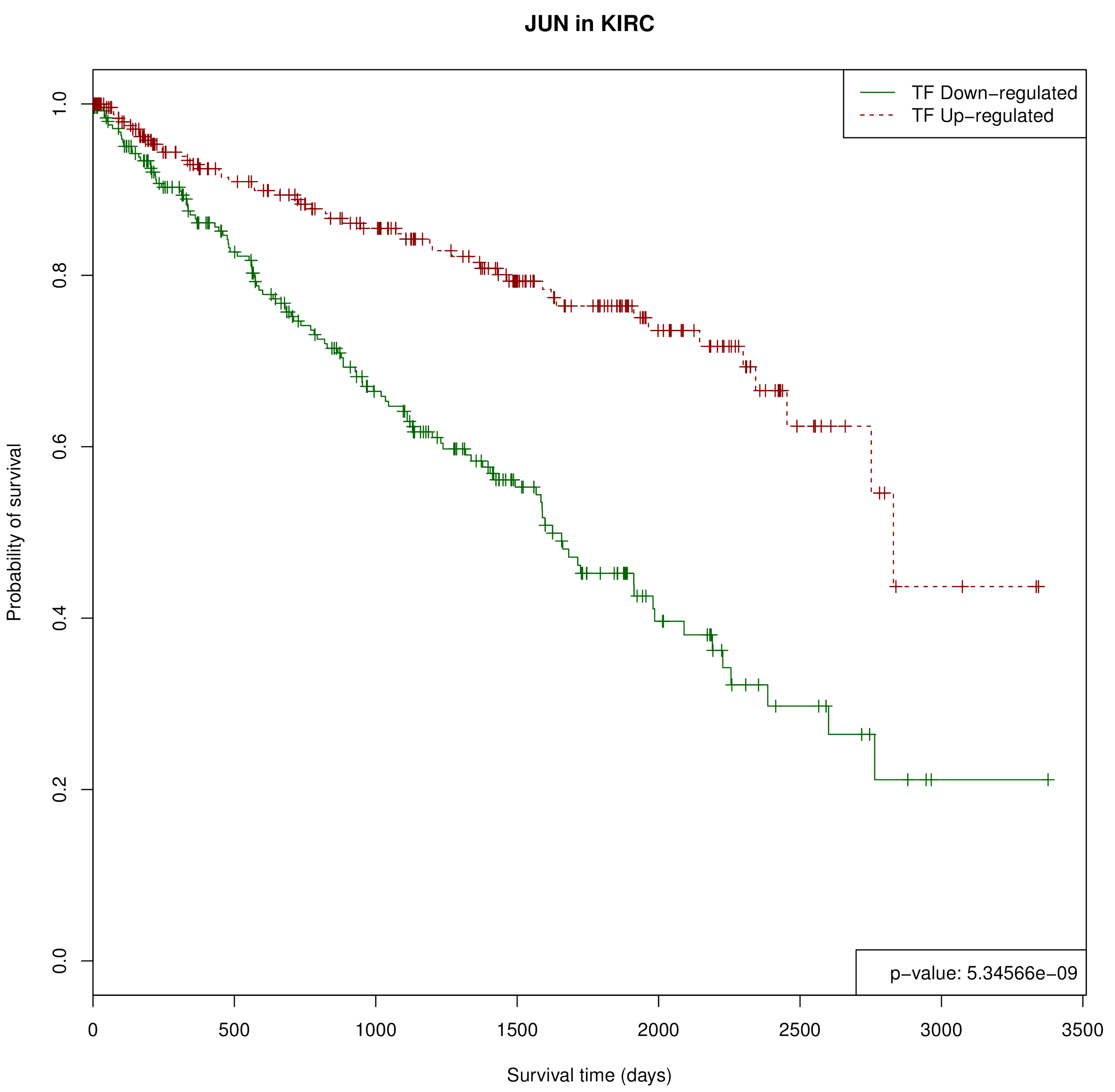
JUN in KIRC
TF Down−regulated
TF Up−regulated
Probability of survival
0.0
0.2
0.4
0.6
0.8
1.0
Survival time (days)
0
500
1000
1500
2000
2500
3000
3500
p−value: 5.34566e−09

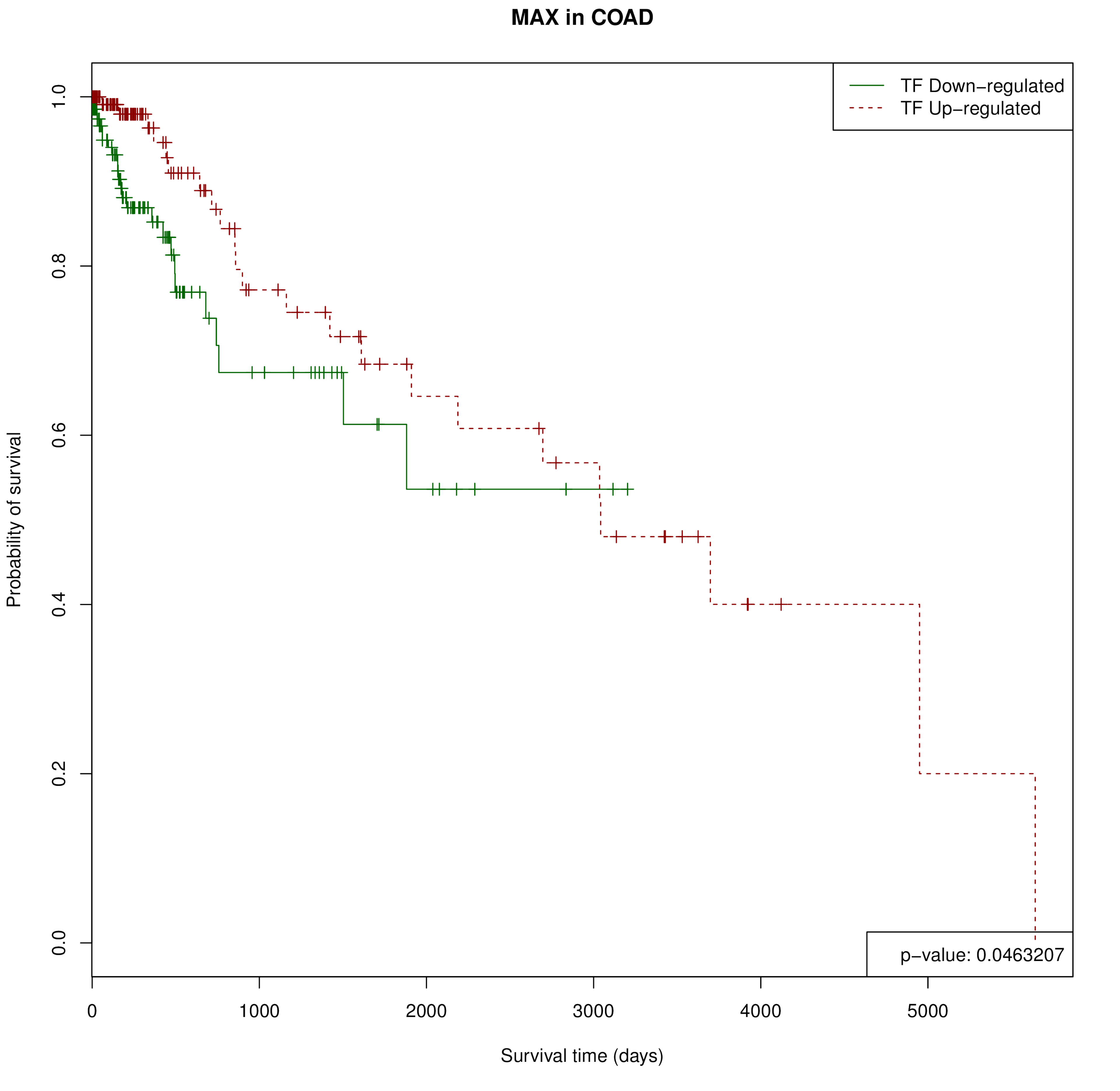

MAX in COAD
TF Down−regulated
TF Up−regulated
Probability of survival
0.0
0.2
0.4
0.6
0.8
1.0
Survival time (days)
0
1000
2000
3000
4000
5000
p−value: 0.0463207

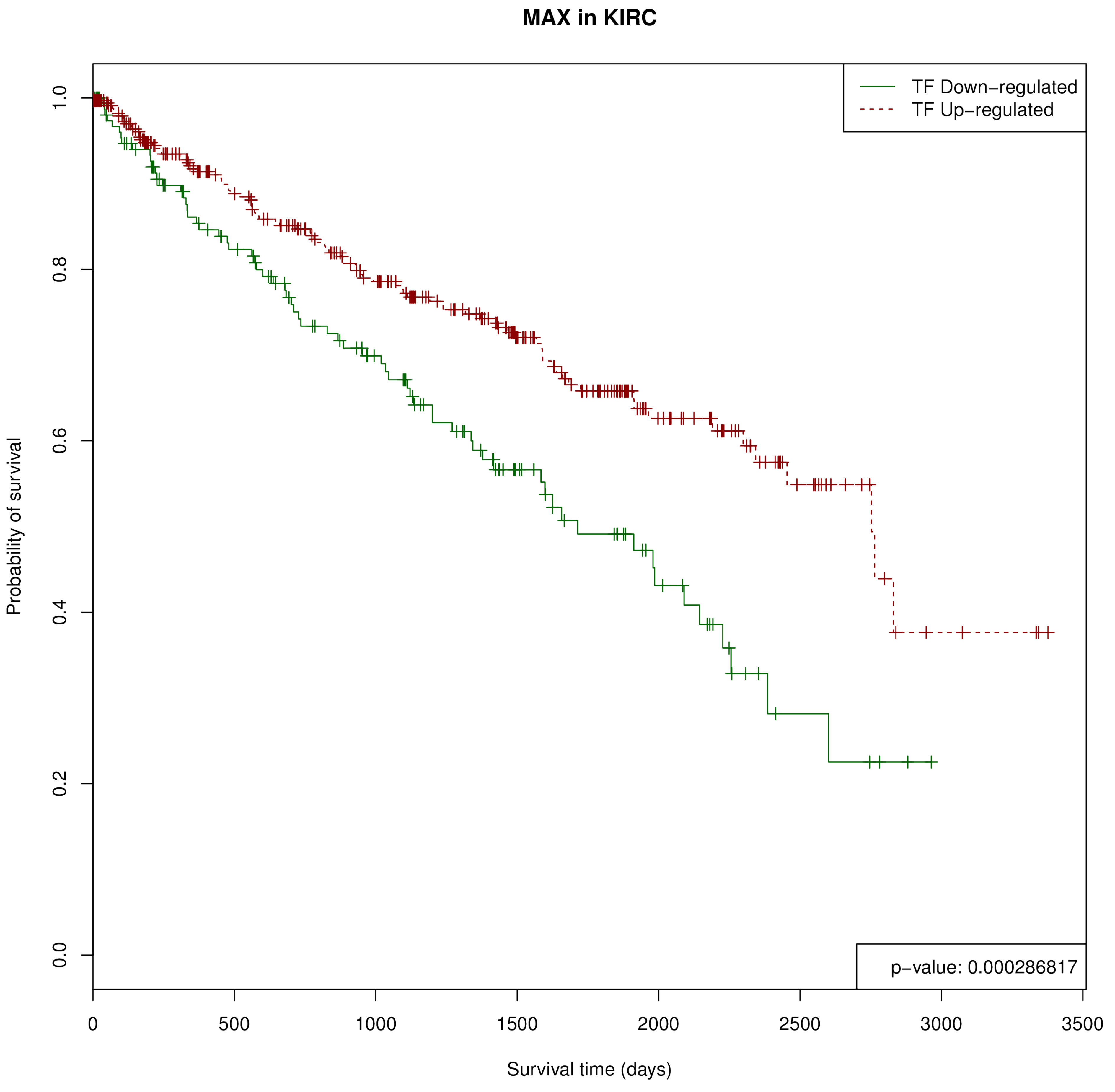

MAX in KIRC
TF Down−regulated
TF Up−regulated
p−value: 0.000286817
Probability of survival
Survival time (days)
0.0
0.2
0.4
0.6
0.8
1.0
0
500
1000
1500
2000
2500
3000
3500

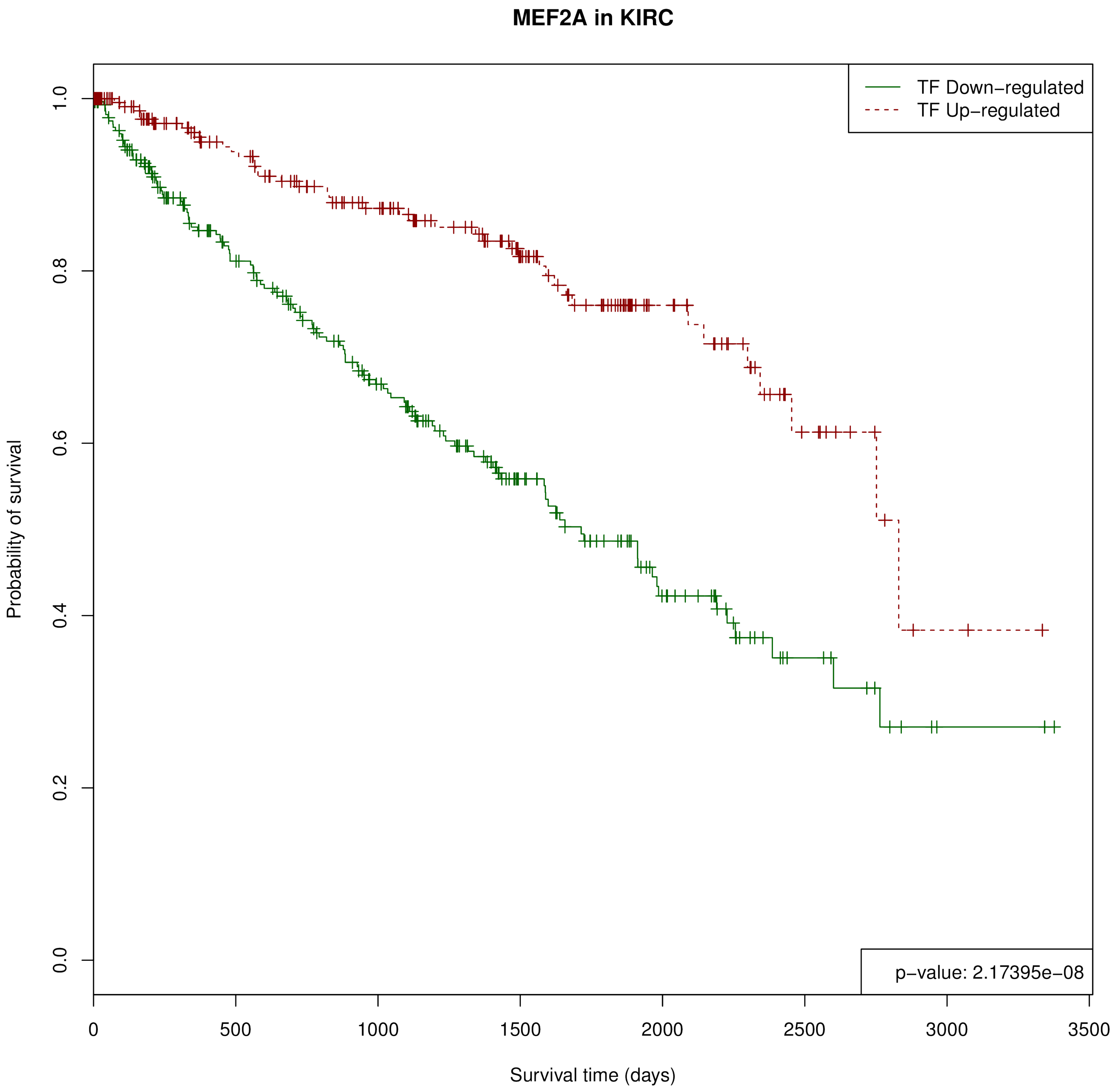
MEF2A in KIRC
TF Down−regulated
TF Up−regulated
p−value: 2.17395e−08
Probability of survival
Survival time (days)
0.0
0.2
0.4
0.6
0.8
1.0
0
500
1000
1500
2000
2500
3000
3500

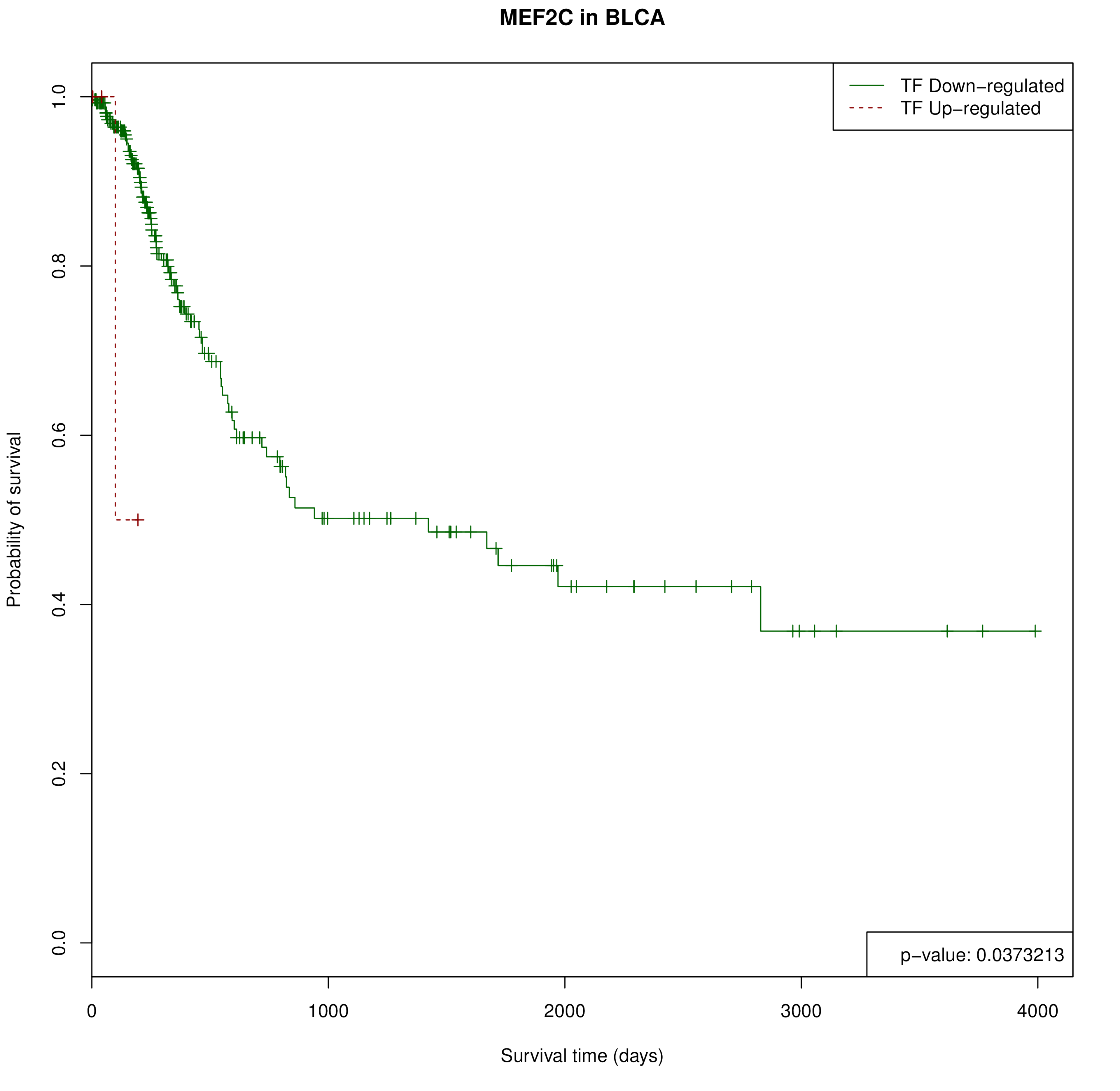
MEF2C in BLCA
TF Down−regulated
TF Up−regulated
p−value: 0.0373213
Probability of survival
Survival time (days)
0.0
0.2
0.4
0.6
0.8
1.0
0
1000
2000
3000
4000

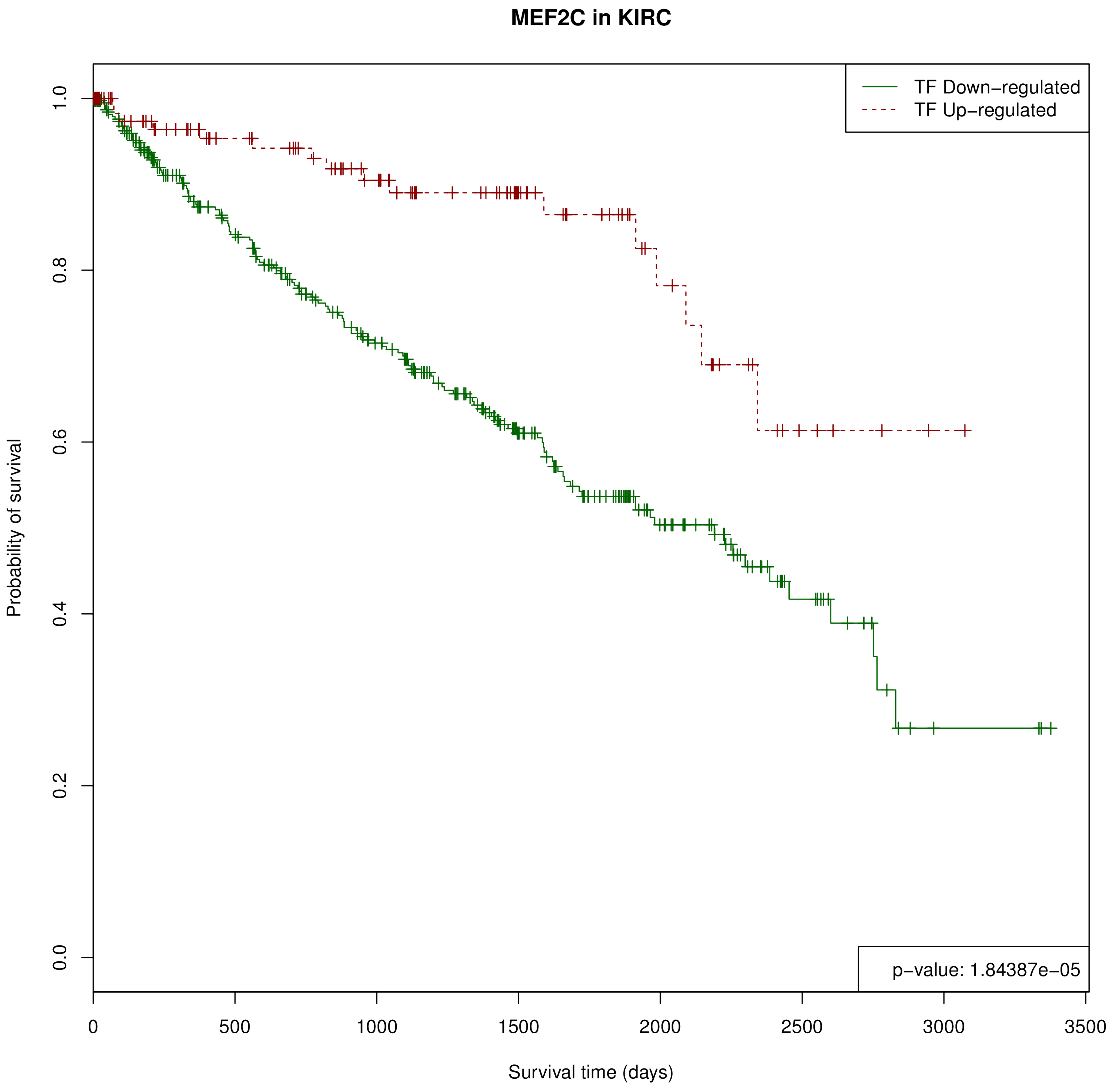

MEF2C in KIRC
TF Down−regulated
TF Up−regulated
p−value: 1.84387e−05
Probability of survival
0.0
0.2
0.4
0.6
0.8
1.0
Survival time (days)
0
500
1000
1500
2000
2500
3000
3500

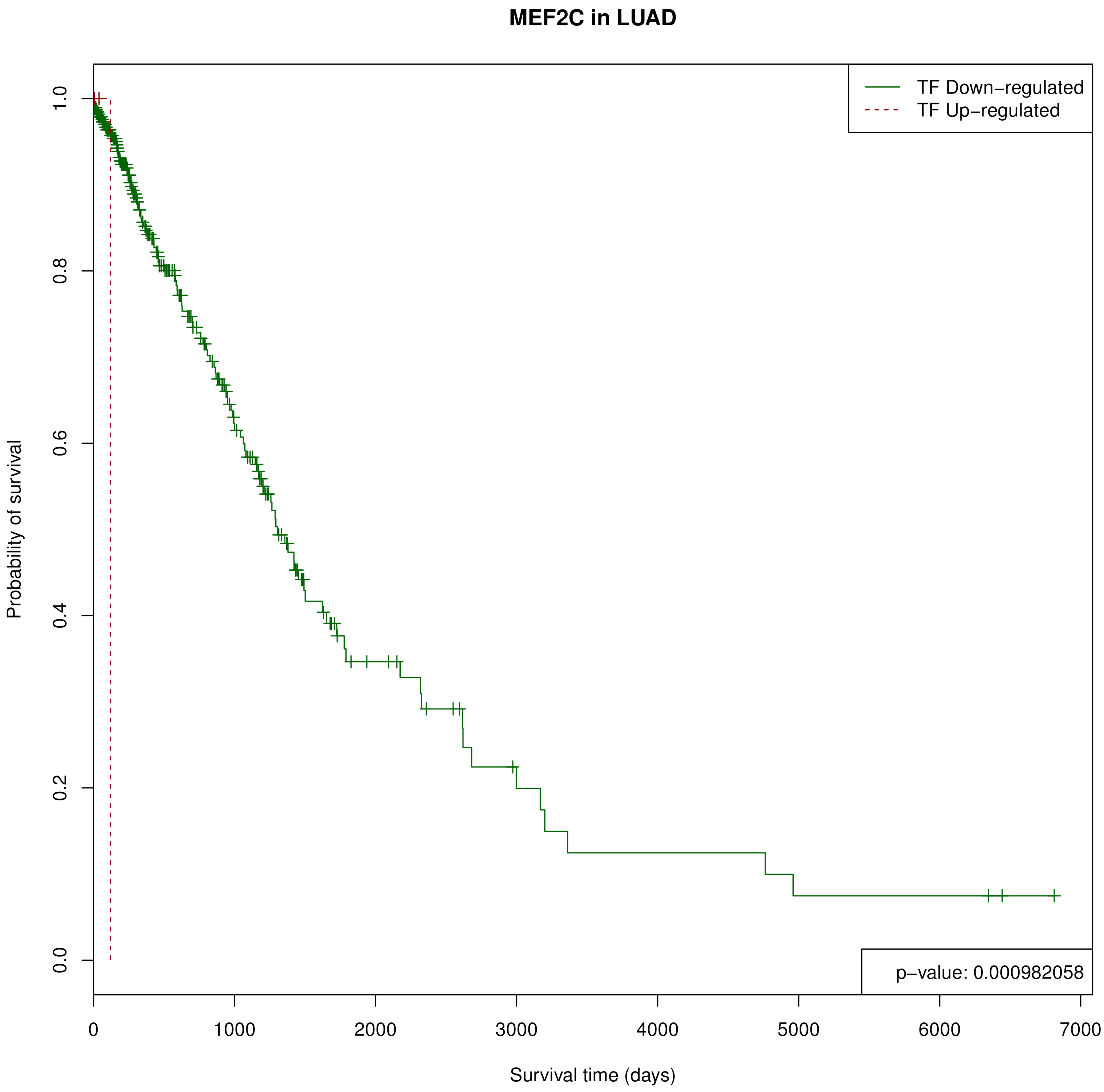

MEF2C in LUAD
TF Down−regulated
TF Up−regulated
p−value: 0.000982058
Probability of survival
Survival time (days)
0.0
0.2
0.4
0.6
0.8
1.0
0
1000
2000
3000
4000
5000
6000
7000

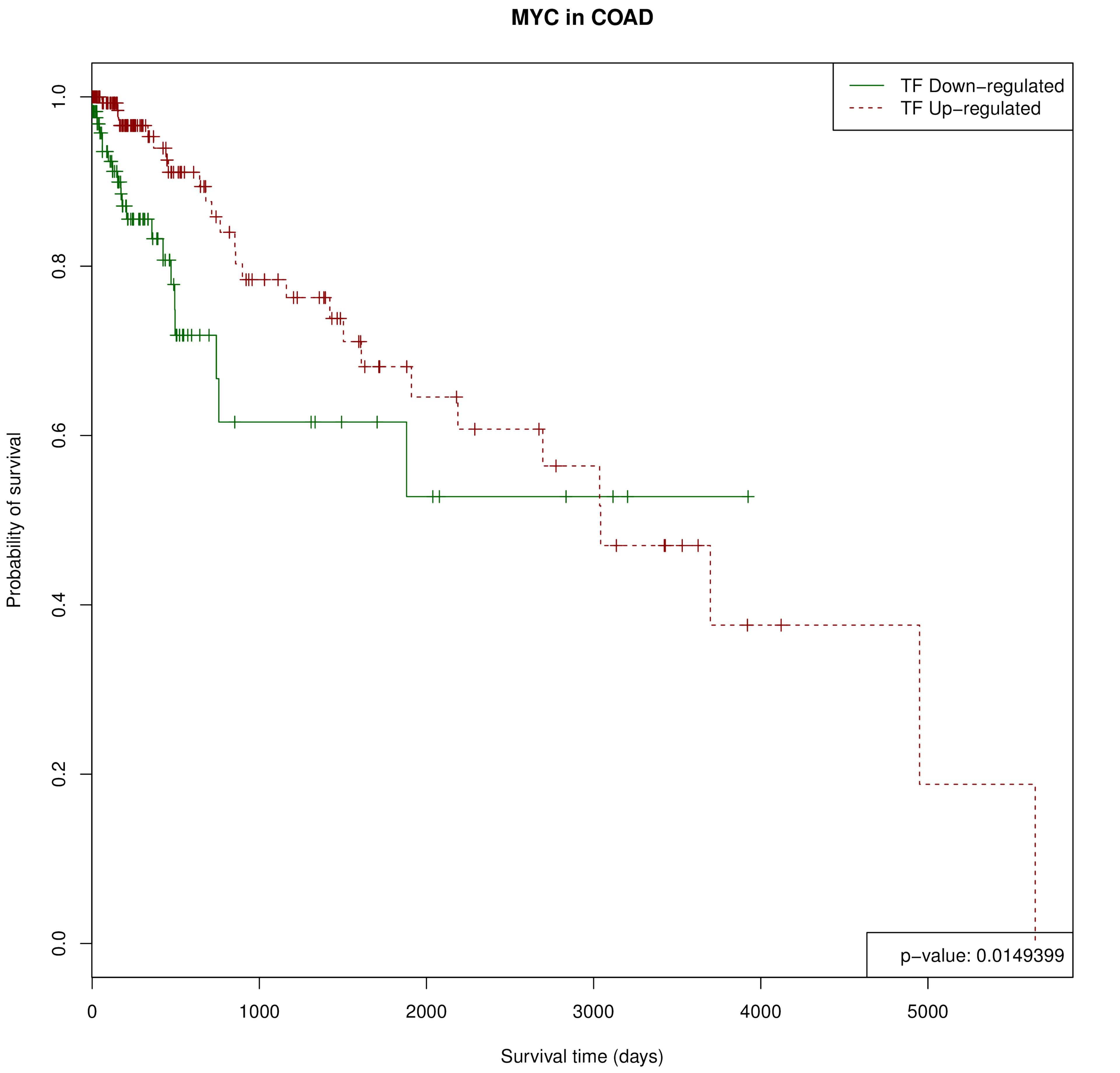
MYC in COAD
TF Down−regulated
TF Up−regulated
Probability of survival
0.0
0.2
0.4
0.6
0.8
1.0
0
1000
2000
3000
4000
5000
Survival time (days)
p−value: 0.0149399

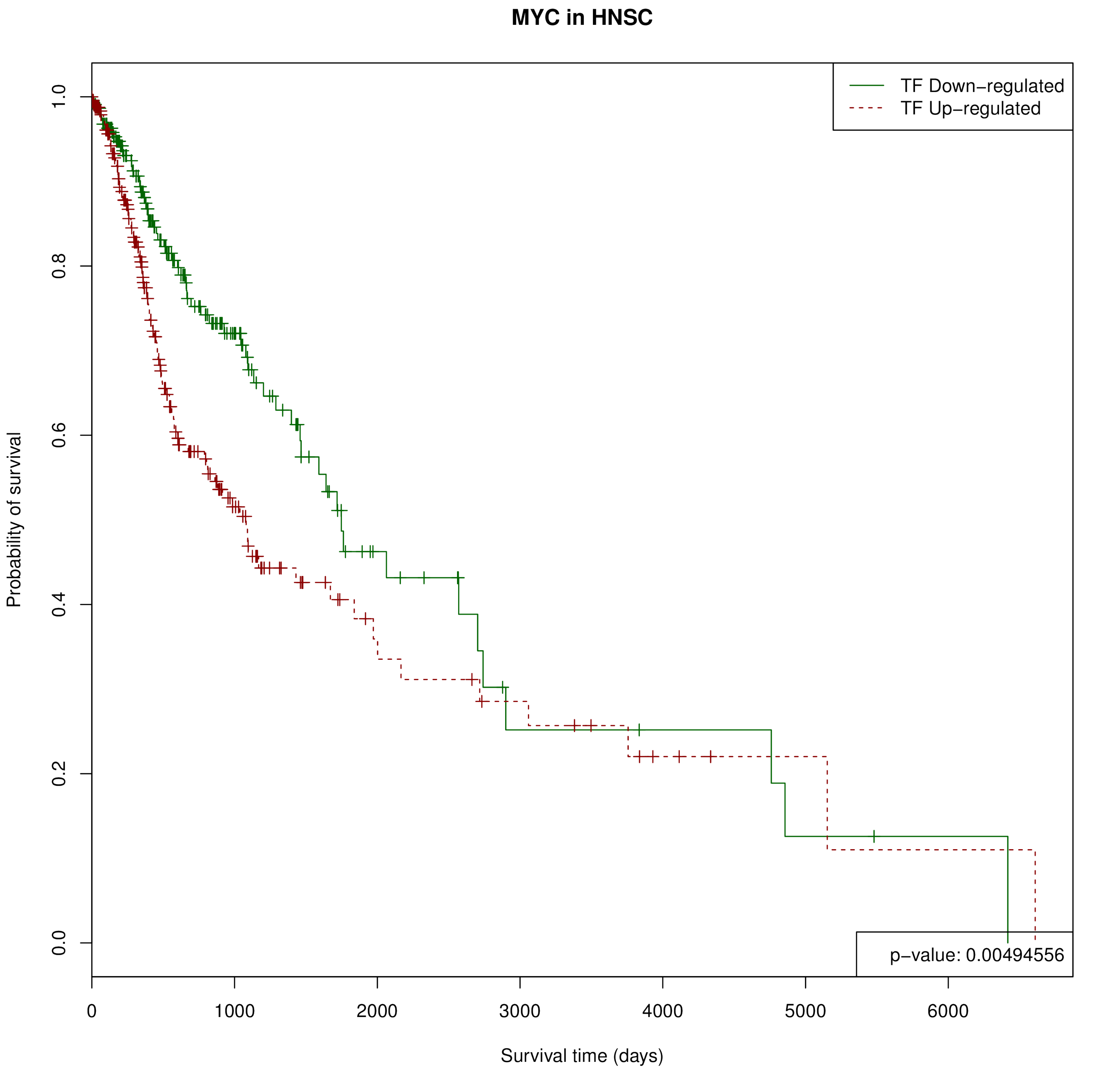

MYC in HNSC
TF Down−regulated
TF Up−regulated
p−value: 0.00494556
Probability of survival
Survival time (days)
0.0
0.2
0.4
0.6
0.8
1.0
0
1000
2000
3000
4000
5000
6000

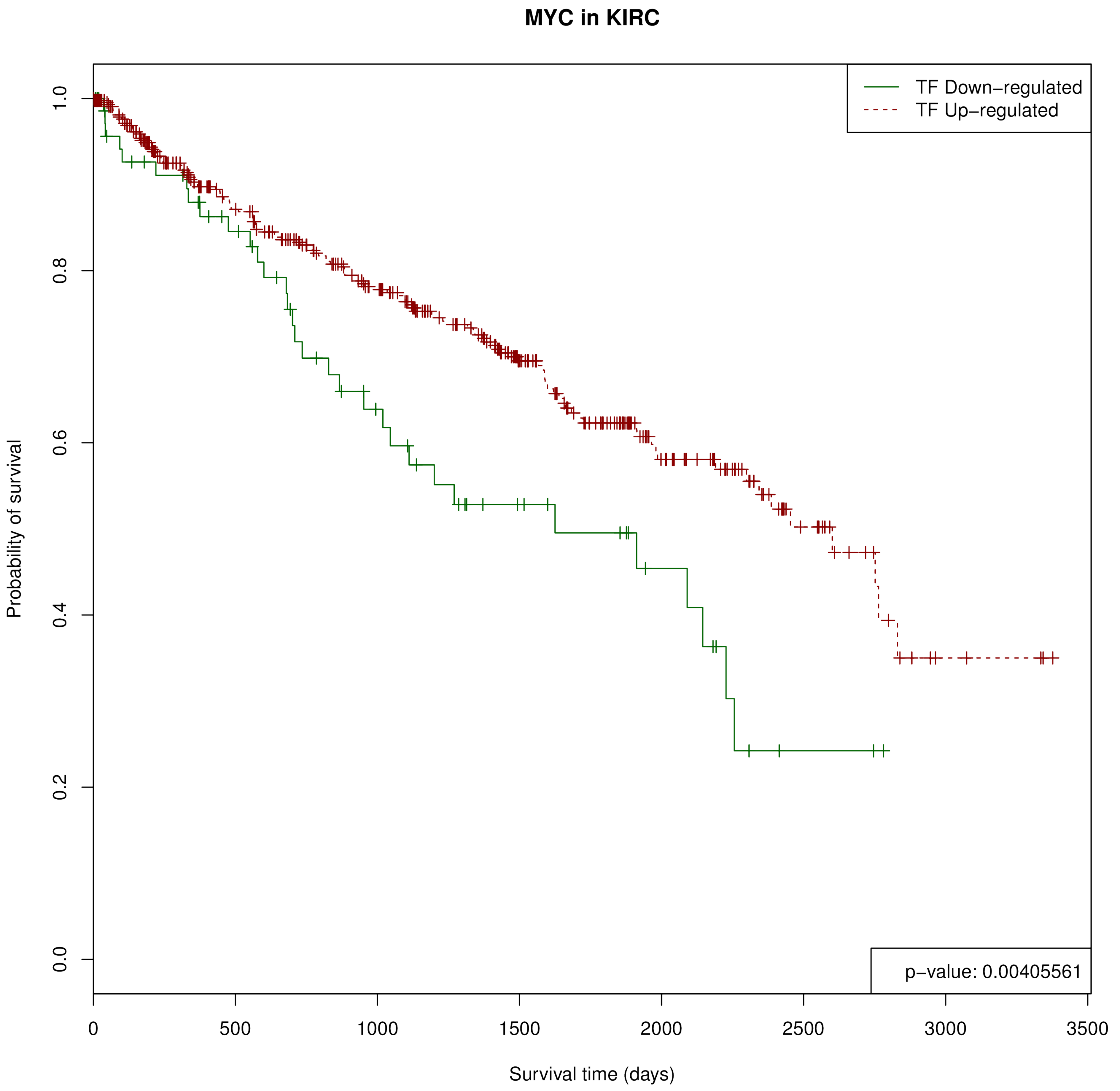
MYC in KIRC
TF Down−regulated
TF Up−regulated
Probability of survival
0.0
0.2
0.4
0.6
0.8
1.0
Survival time (days)
0
500
1000
1500
2000
2500
3000
3500
p−value: 0.00405561

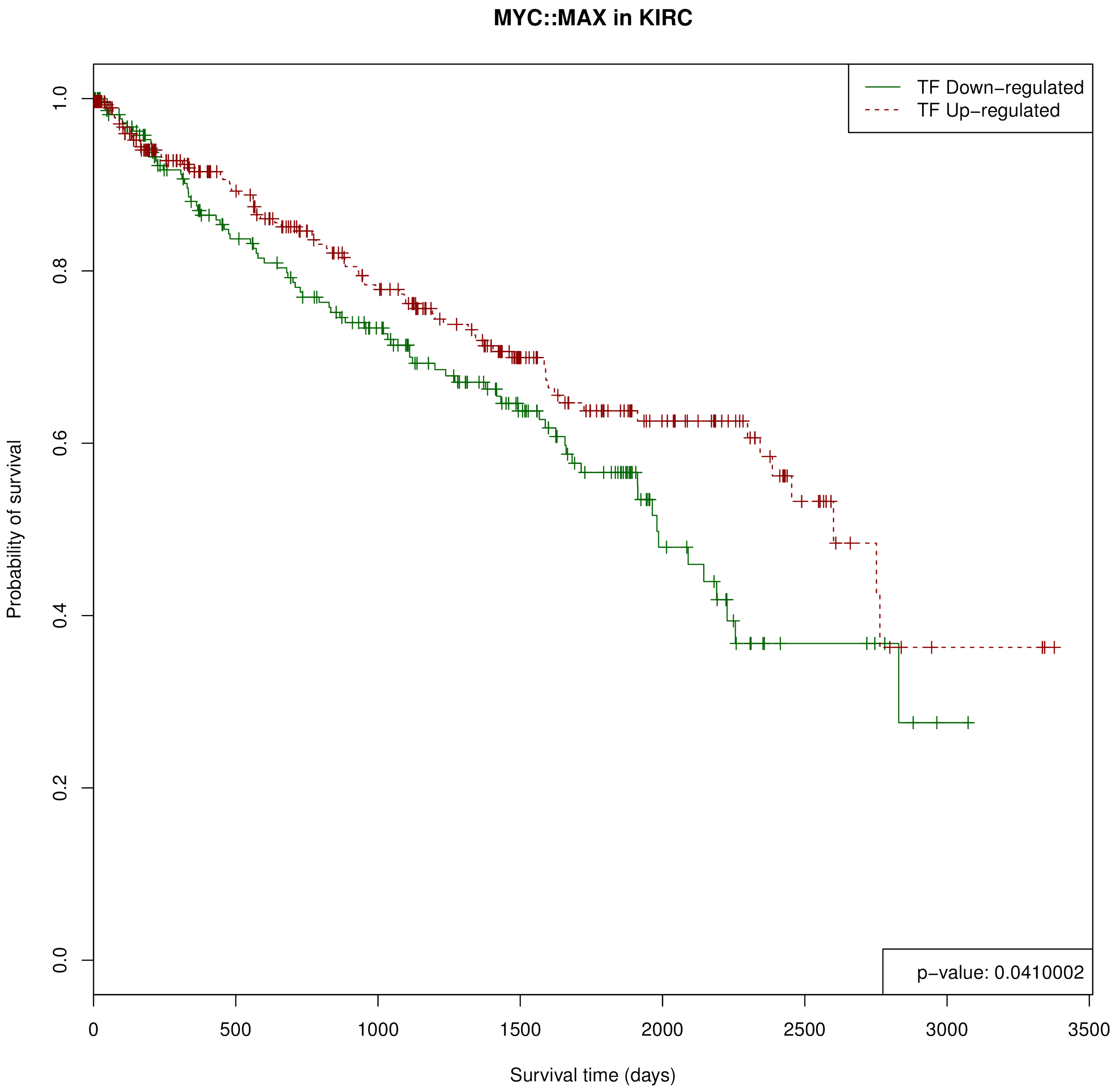
MYC::MAX in KIRC
TF Down−regulated
TF Up−regulated
p−value: 0.0410002
Probability of survival
Survival time (days)
0.0
0.2
0.4
0.6
0.8
1.0
0
500
1000
1500
2000
2500
3000
3500

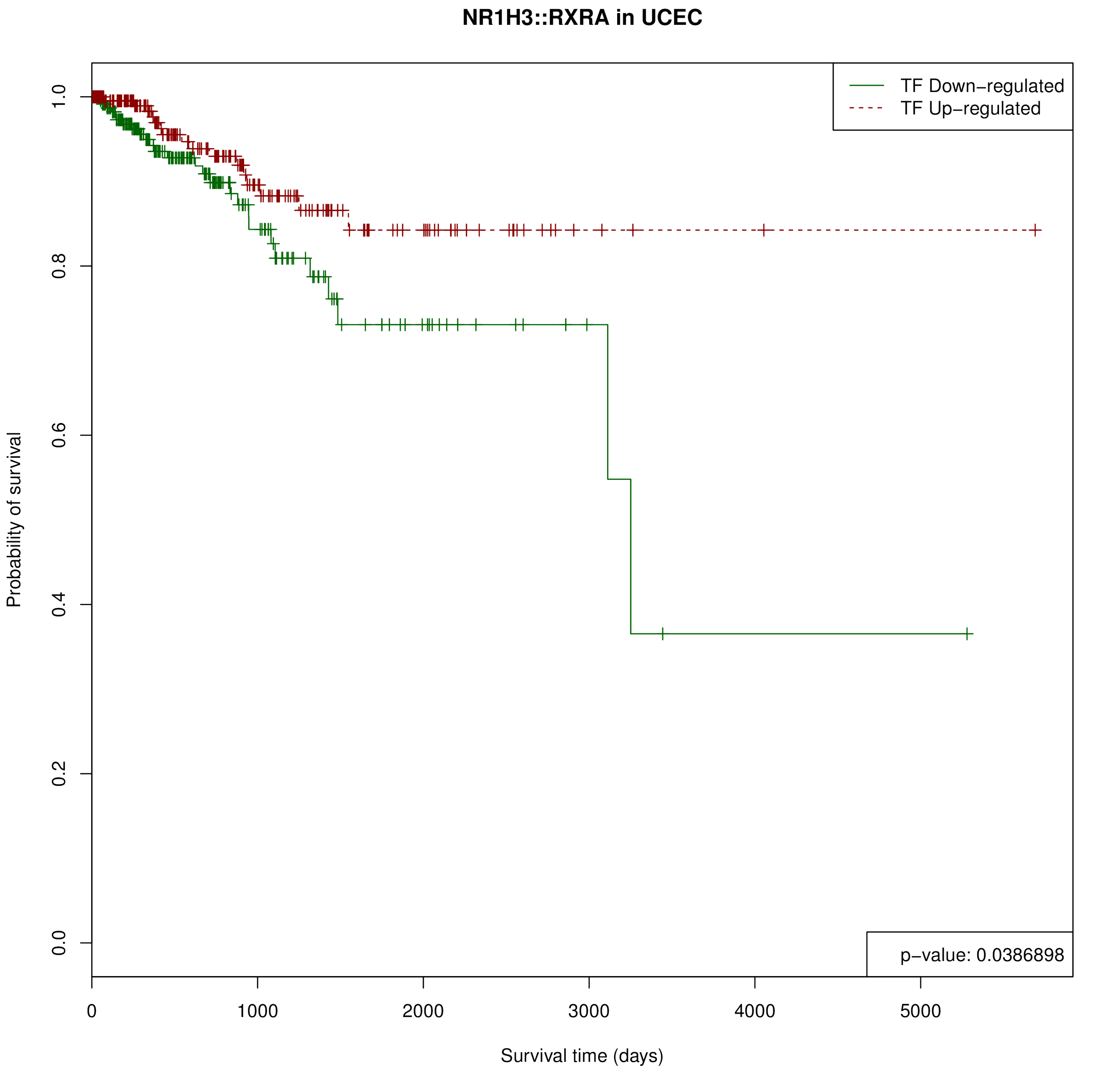

NR1H3::RXRA in UCEC
TF Down−regulated
TF Up−regulated
Probability of survival
0.0
0.2
0.4
0.6
0.8
1.0
Survival time (days)
0
1000
2000
3000
4000
5000
p−value: 0.0386898

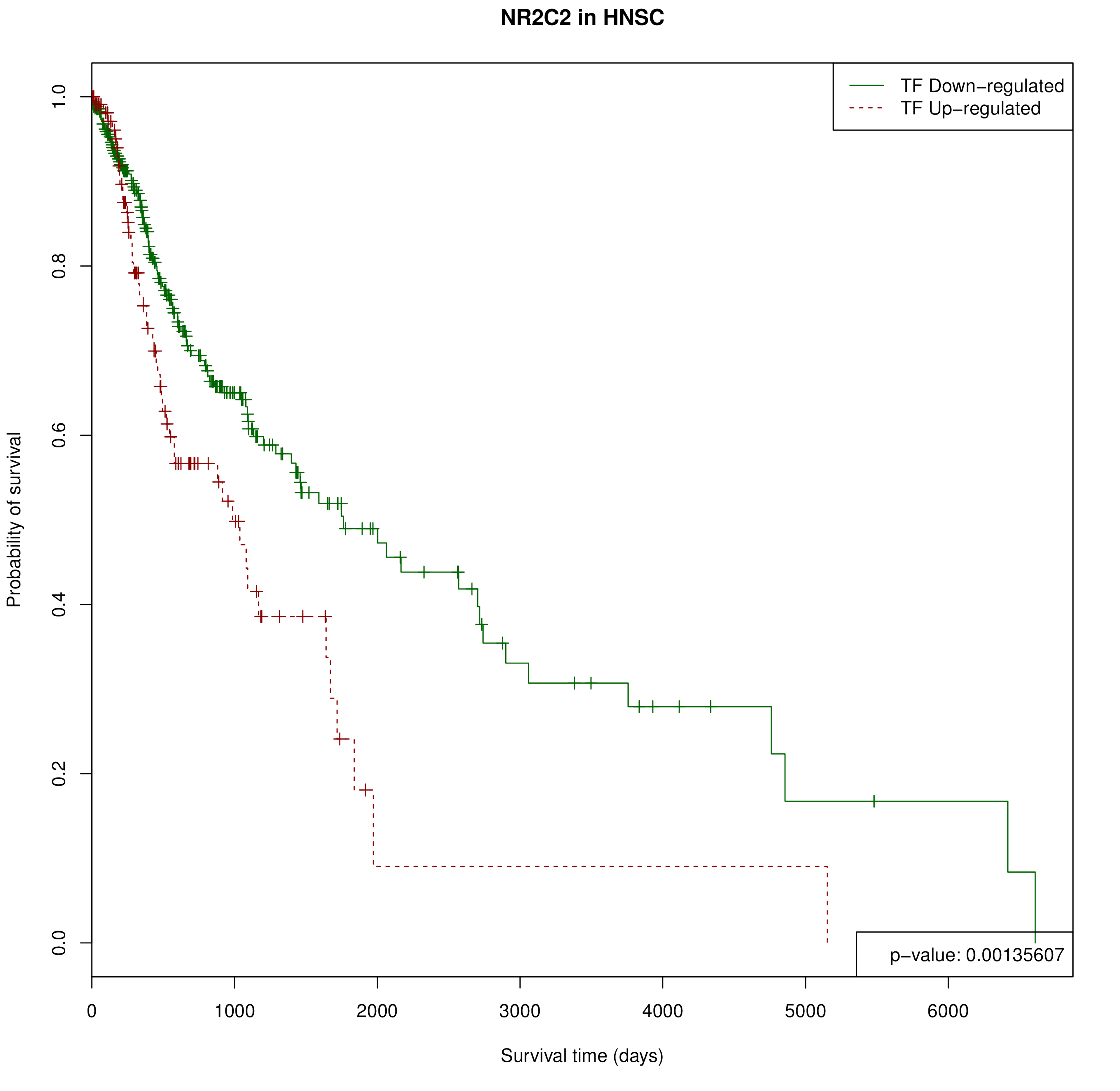

NR2C2 in HNSC
TF Down−regulated
TF Up−regulated
Probability of survival
0.0
0.2
0.4
0.6
0.8
1.0
0
1000
2000
3000
4000
5000
6000
Survival time (days)
p−value: 0.00135607

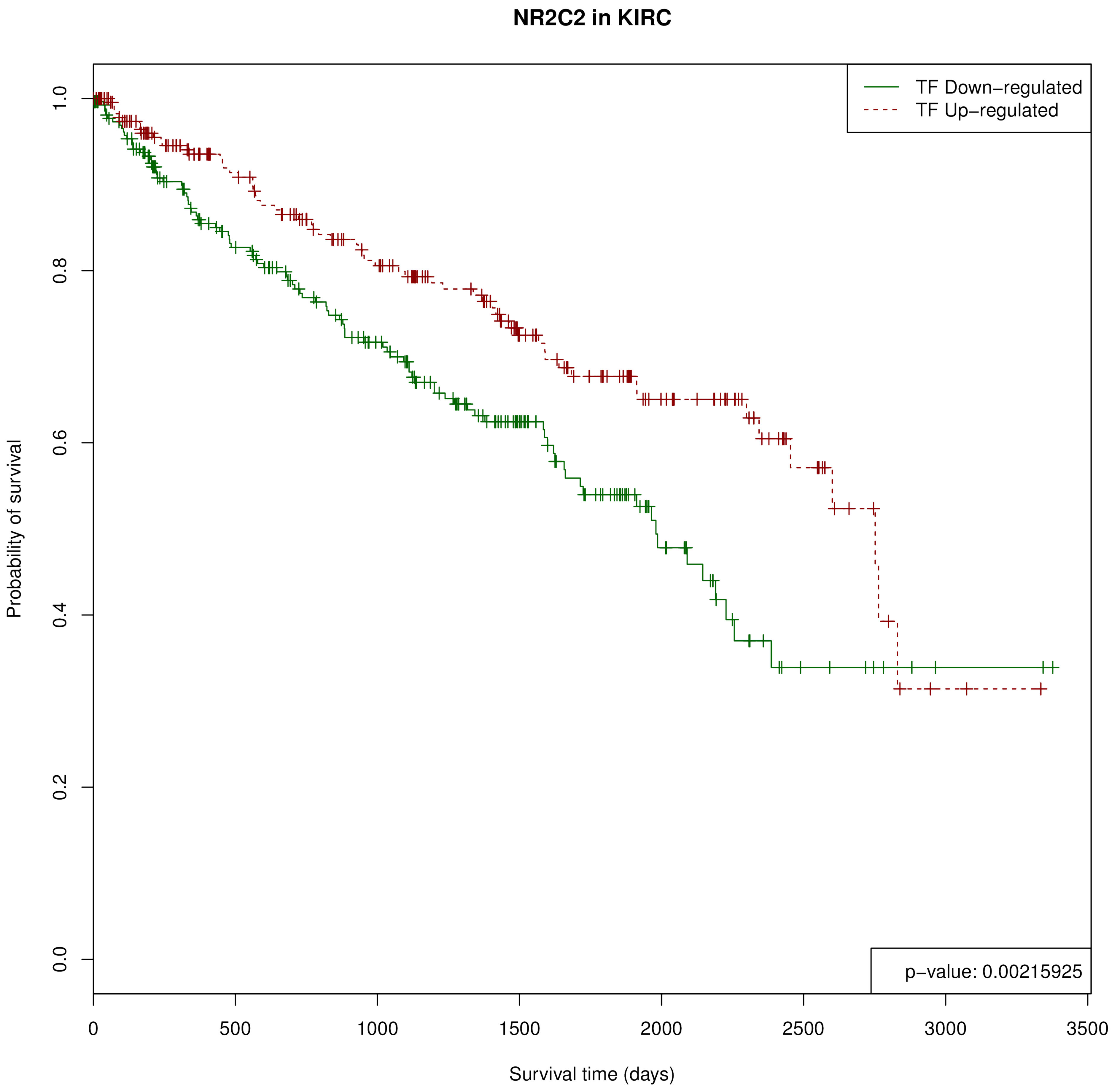

NR2C2 in KIRC
TF Down−regulated
TF Up−regulated
p−value: 0.00215925
Probability of survival
Survival time (days)
0.0
0.2
0.4
0.6
0.8
1.0
0
500
1000
1500
2000
2500
3000
3500

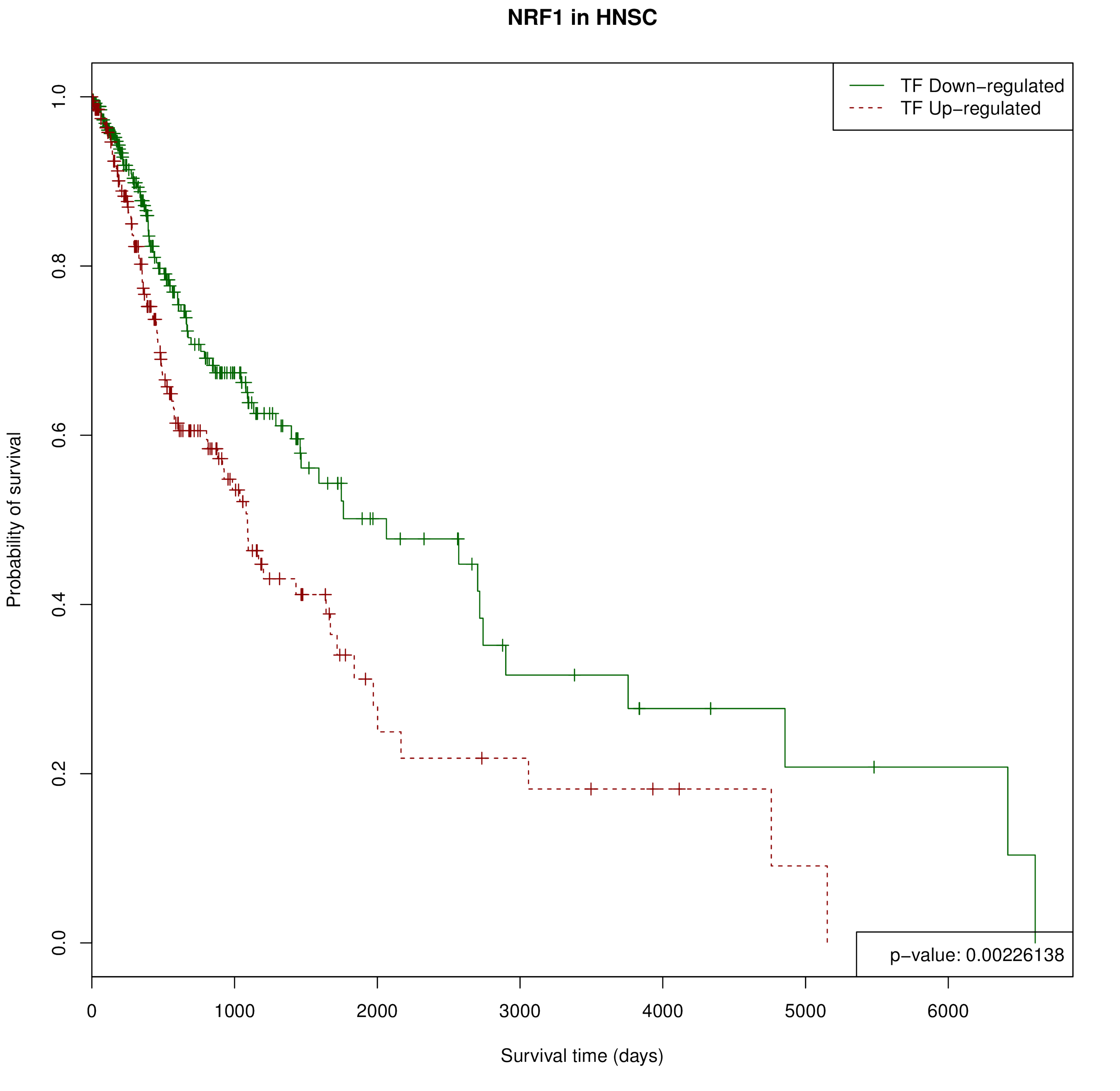
NRF1 in HNSC
TF Down−regulated
TF Up−regulated
p−value: 0.00226138
Probability of survival
Survival time (days)
0.0
0.2
0.4
0.6
0.8
1.0
0
1000
2000
3000
4000
5000
6000

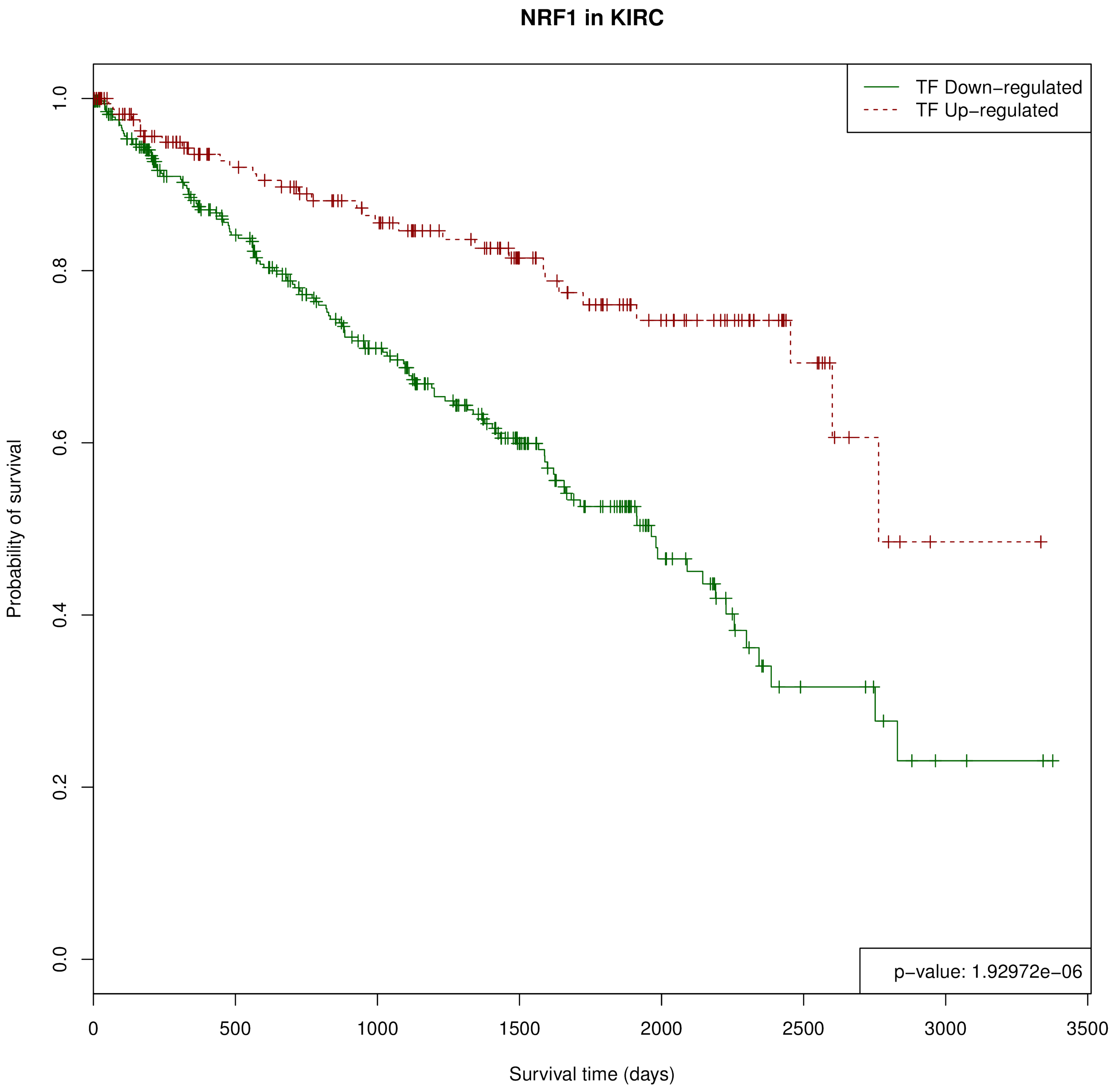
NRF1 in KIRC
TF Down−regulated
TF Up−regulated
p−value: 1.92972e−06
Probability of survival
Survival time (days)
0.0
0.2
0.4
0.6
0.8
1.0
0
500
1000
1500
2000
2500
3000
3500

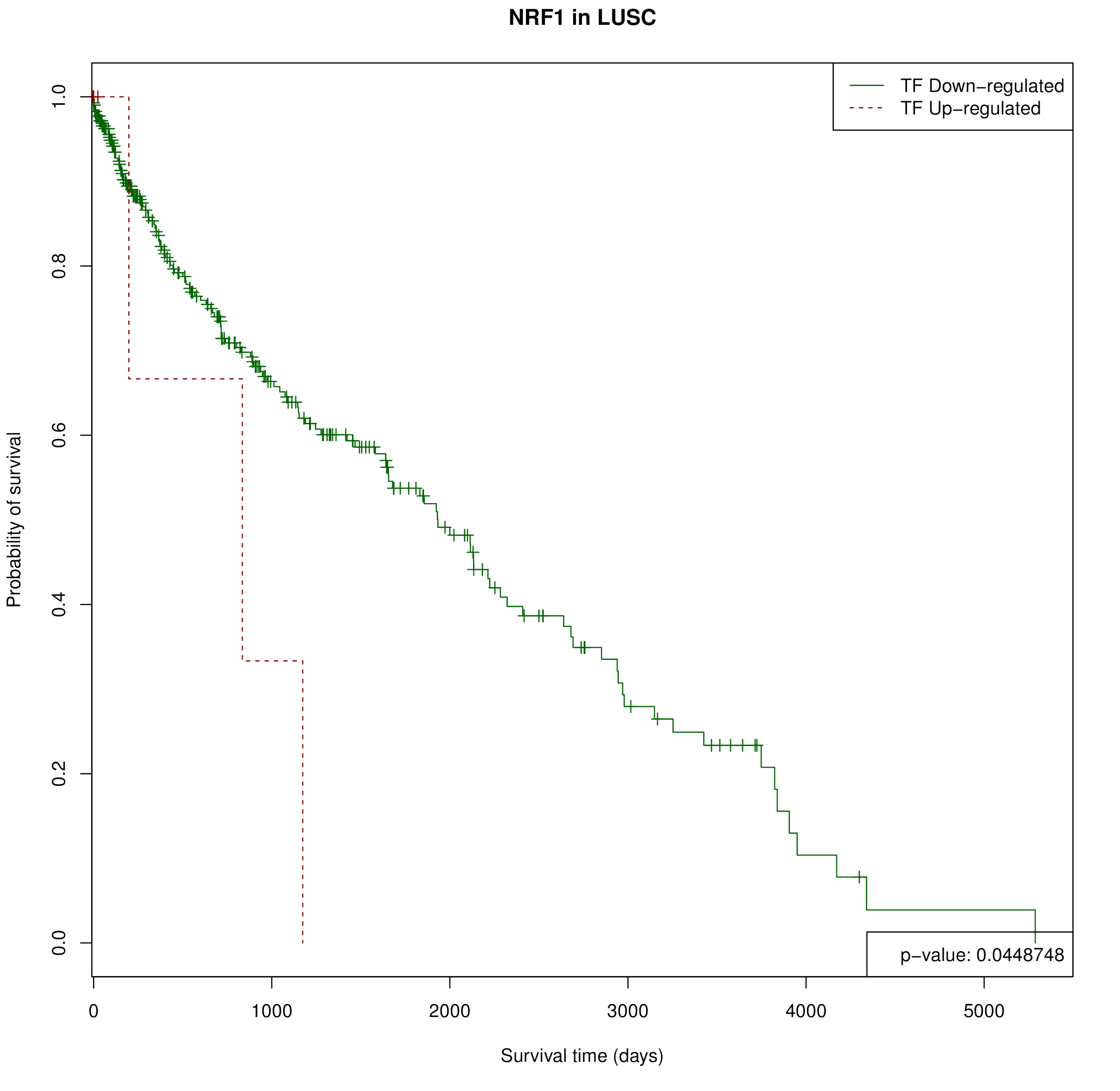
NRF1 in LUSC
TF Down−regulated
TF Up−regulated
Probability of survival
0.0
0.2
0.4
0.6
0.8
1.0
0
1000
2000
3000
4000
5000
Survival time (days)
p−value: 0.0448748

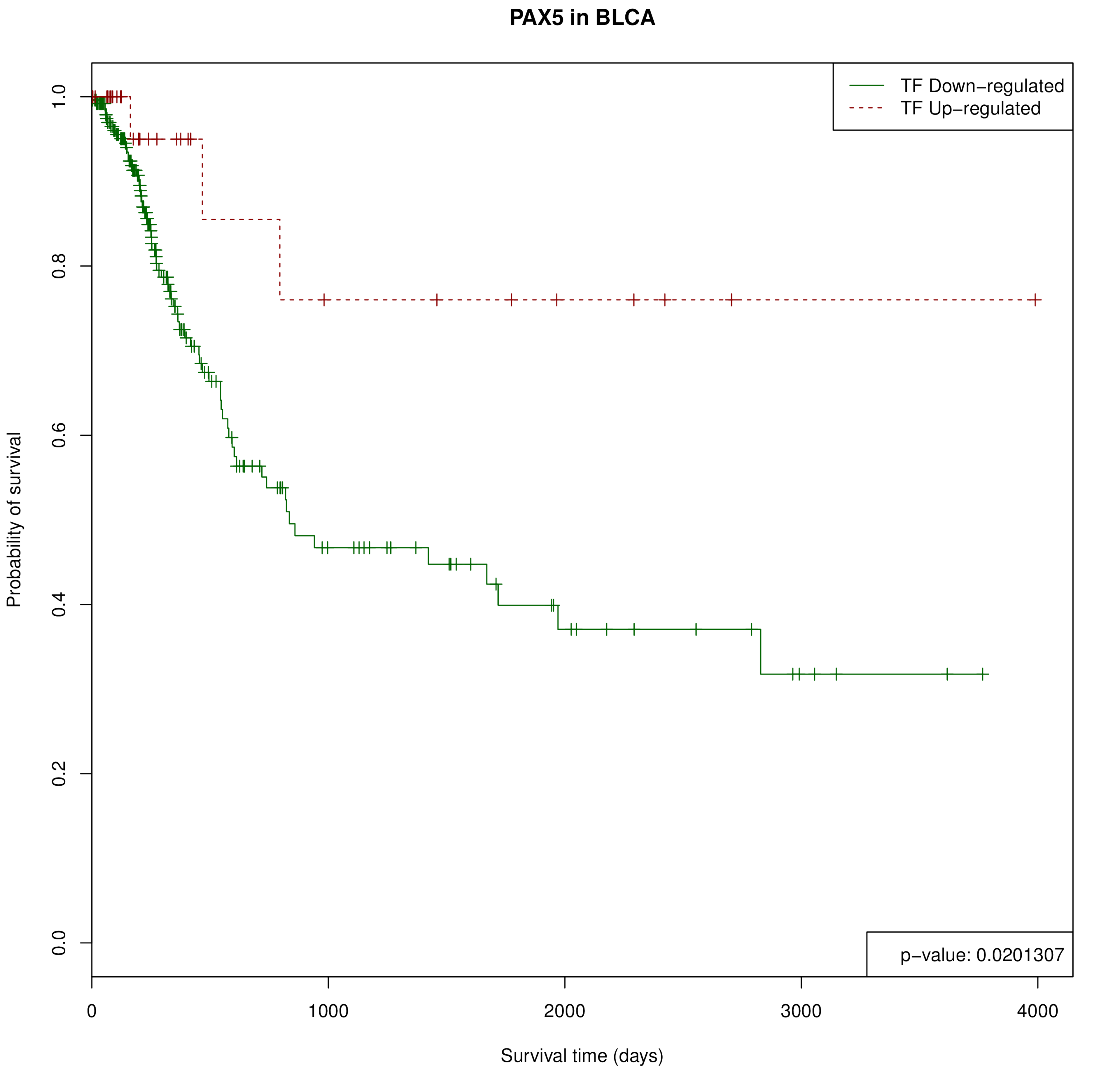
PAX5 in BLCA
TF Down−regulated
TF Up−regulated
p−value: 0.0201307
Probability of survival
Survival time (days)
0.0
0.2
0.4
0.6
0.8
1.0
0
1000
2000
3000
4000

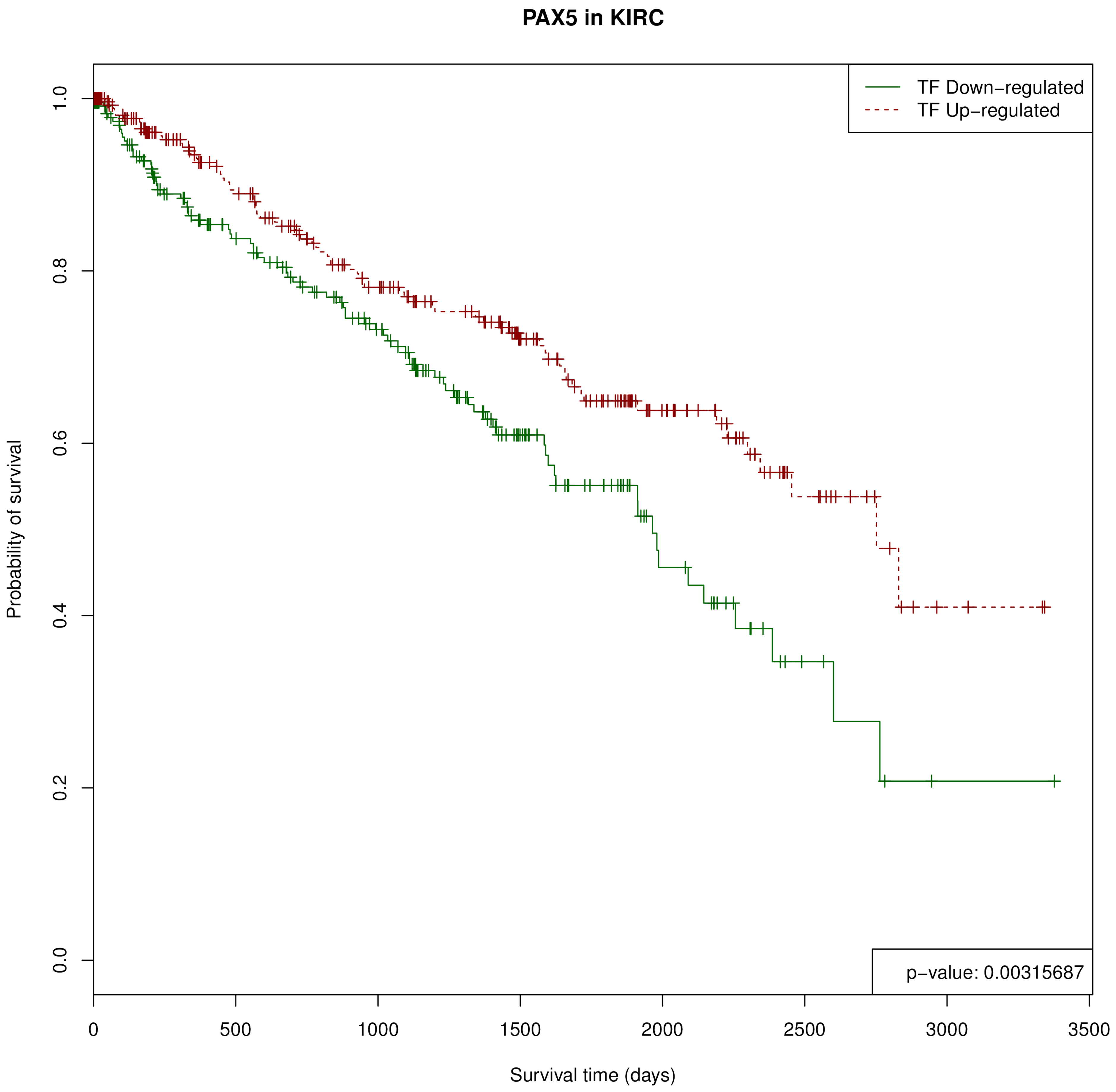

PAX5 in KIRC
TF Down−regulated
TF Up−regulated
p−value: 0.00315687
Probability of survival
Survival time (days)
0.0
0.2
0.4
0.6
0.8
1.0
0
500
1000
1500
2000
2500
3000
3500

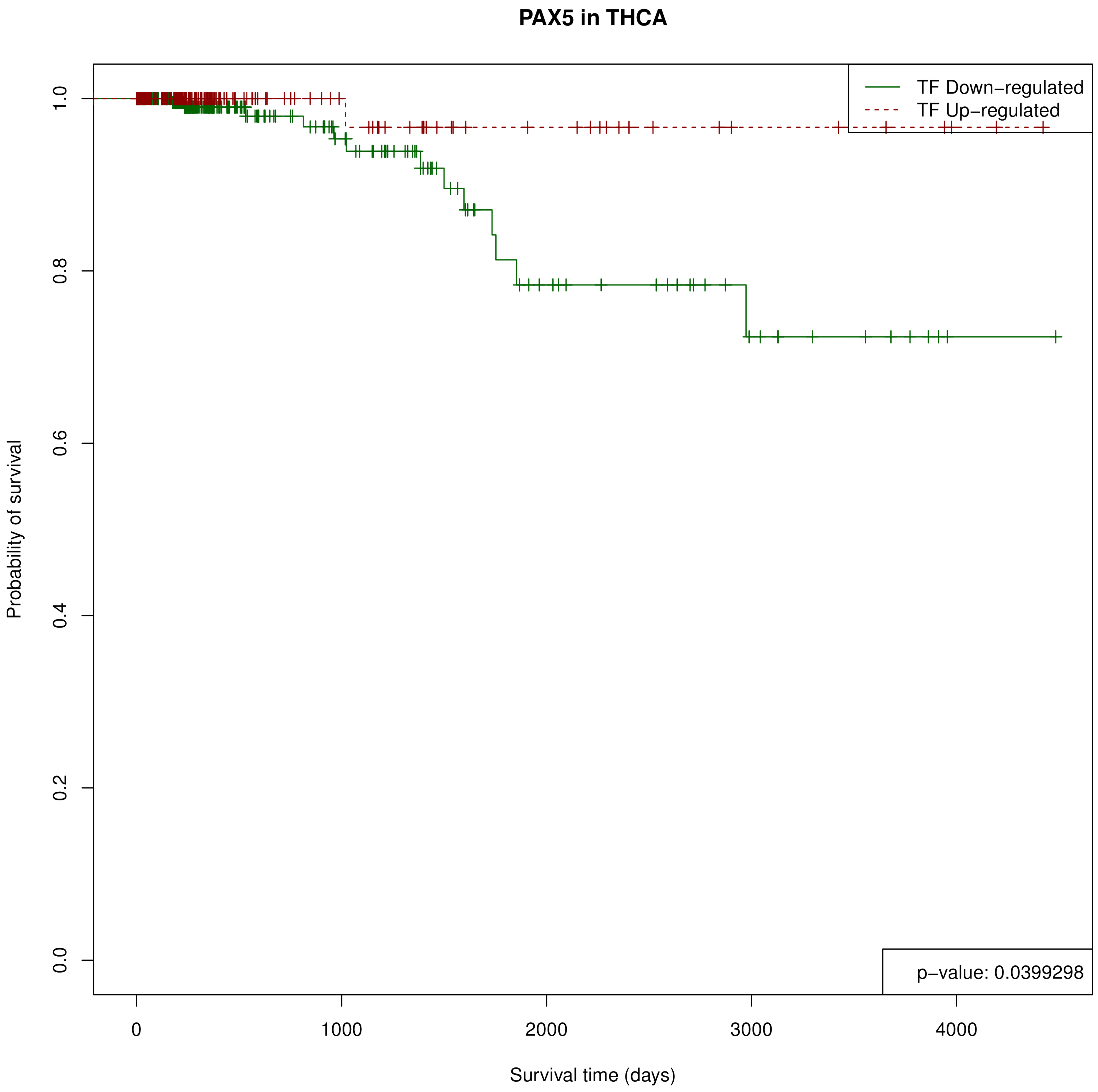
PAX5 in THCA
TF Down−regulated
TF Up−regulated
p−value: 0.0399298
Probability of survival
Survival time (days)
0.0
0.2
0.4
0.6
0.8
1.0
0
1000
2000
3000
4000

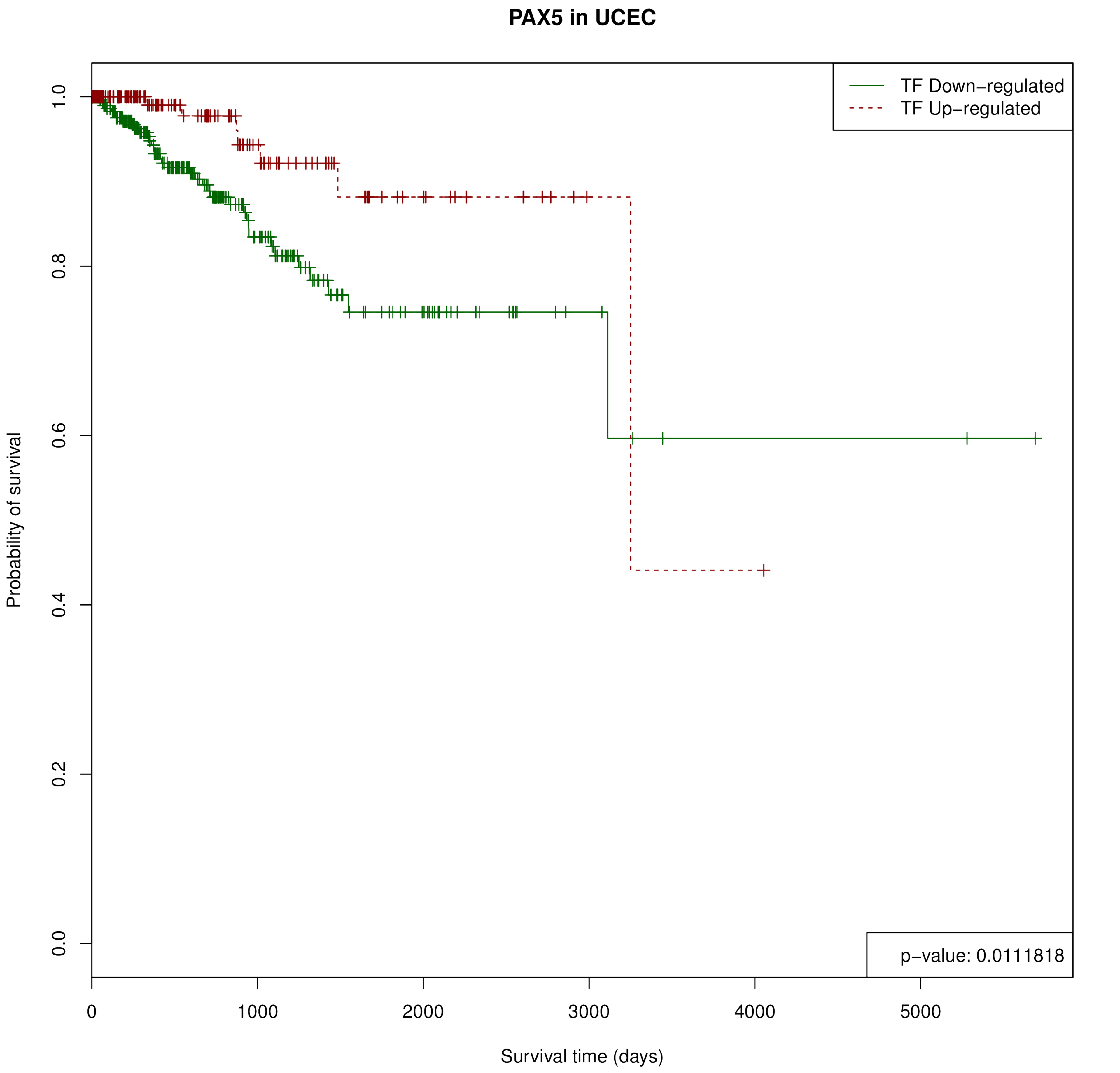

PAX5 in UCEC
TF Down−regulated
TF Up−regulated
p−value: 0.0111818
Probability of survival
Survival time (days)
0.0
0.2
0.4
0.6
0.8
1.0
0
1000
2000
3000
4000
5000

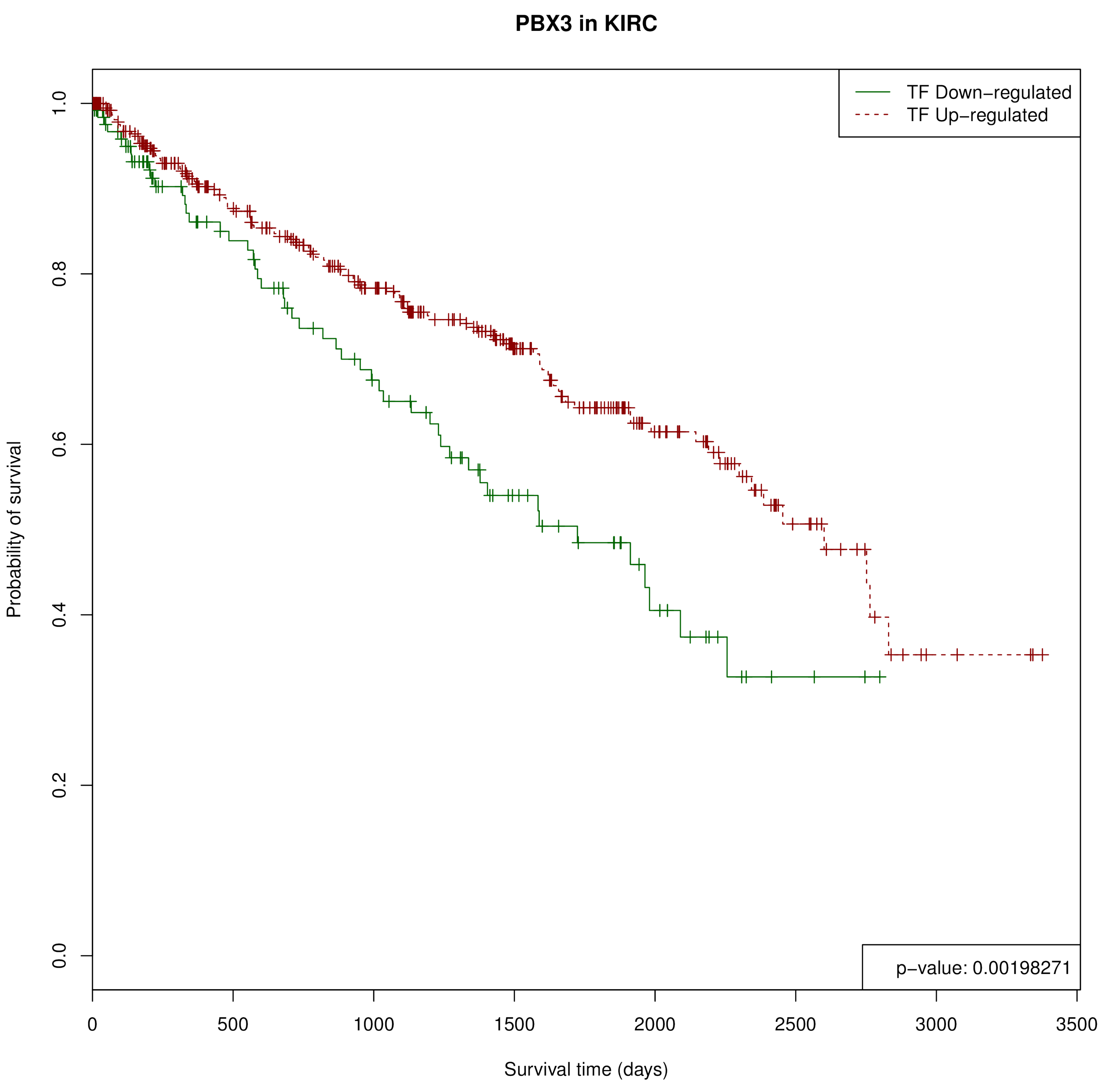
PBX3 in KIRC
TF Down−regulated
TF Up−regulated
p−value: 0.00198271
Probability of survival
Survival time (days)
0.0
0.2
0.4
0.6
0.8
1.0
0
500
1000
1500
2000
2500
3000
3500

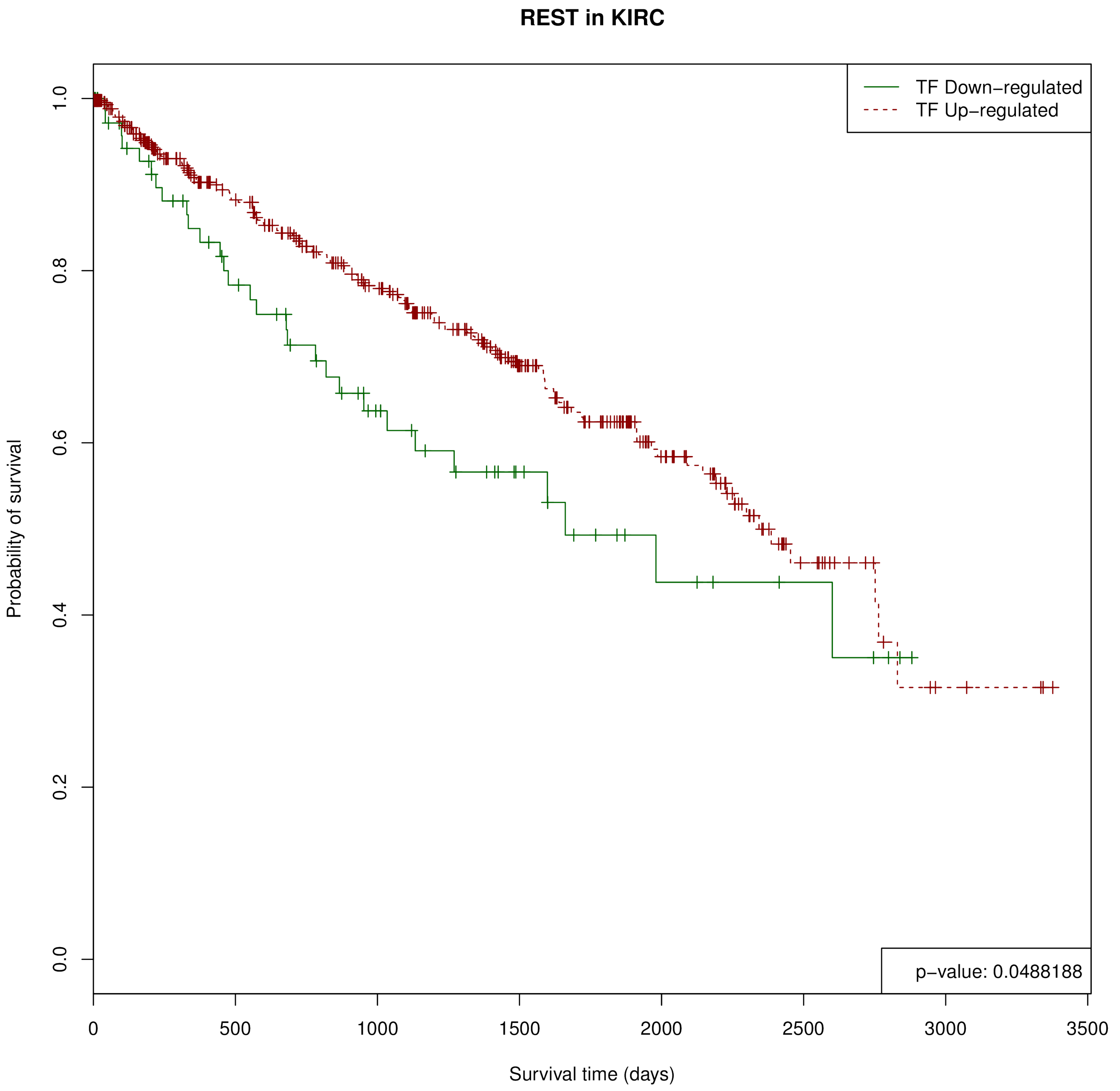
REST in KIRC
TF Down−regulated
TF Up−regulated
Probability of survival
0.0
0.2
0.4
0.6
0.8
1.0
Survival time (days)
0
500
1000
1500
2000
2500
3000
3500
p−value: 0.0488188

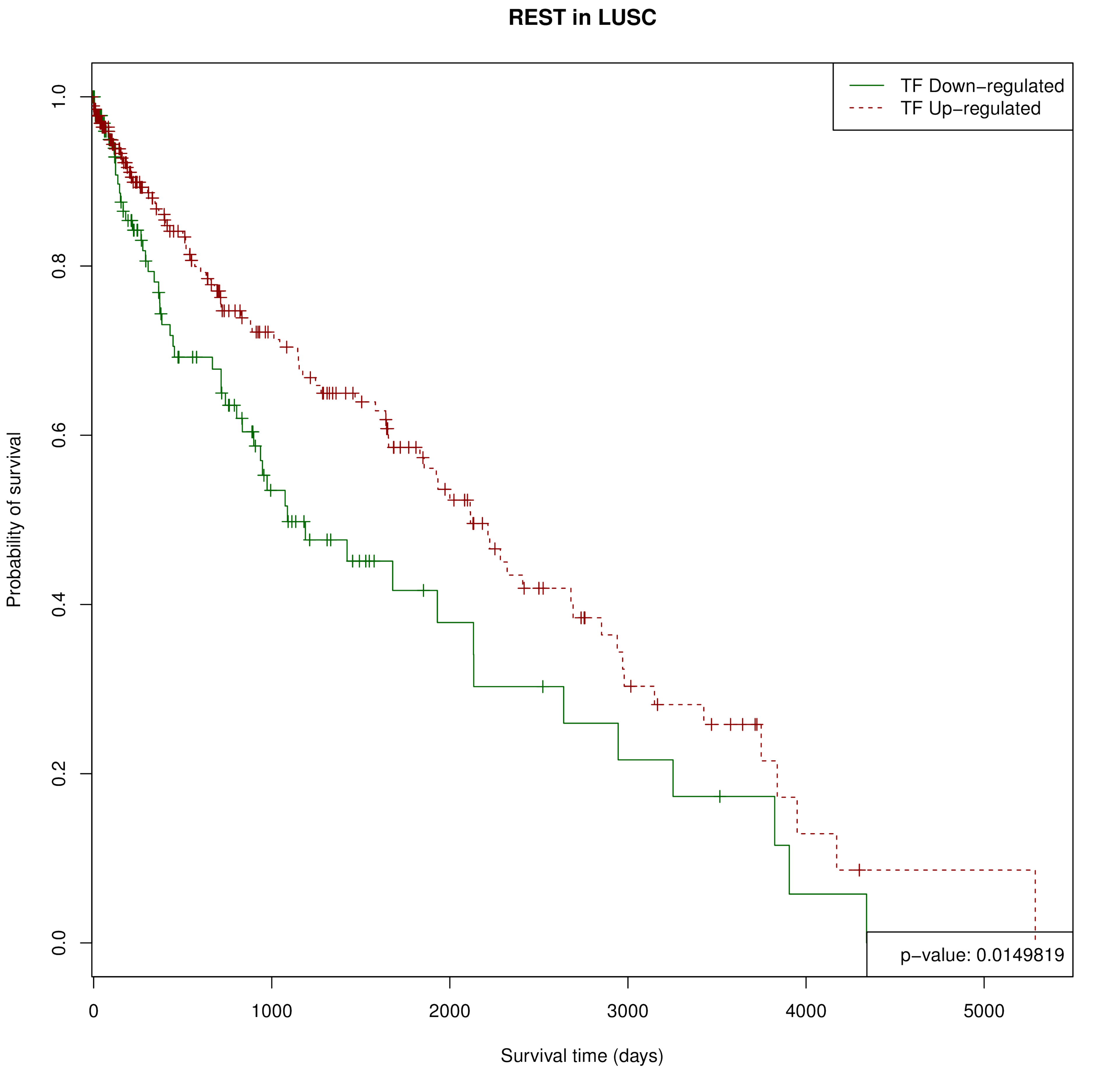

REST in LUSC
TF Down−regulated
TF Up−regulated
Probability of survival
0.0
0.2
0.4
0.6
0.8
1.0
Survival time (days)
0
1000
2000
3000
4000
5000
p−value: 0.0149819

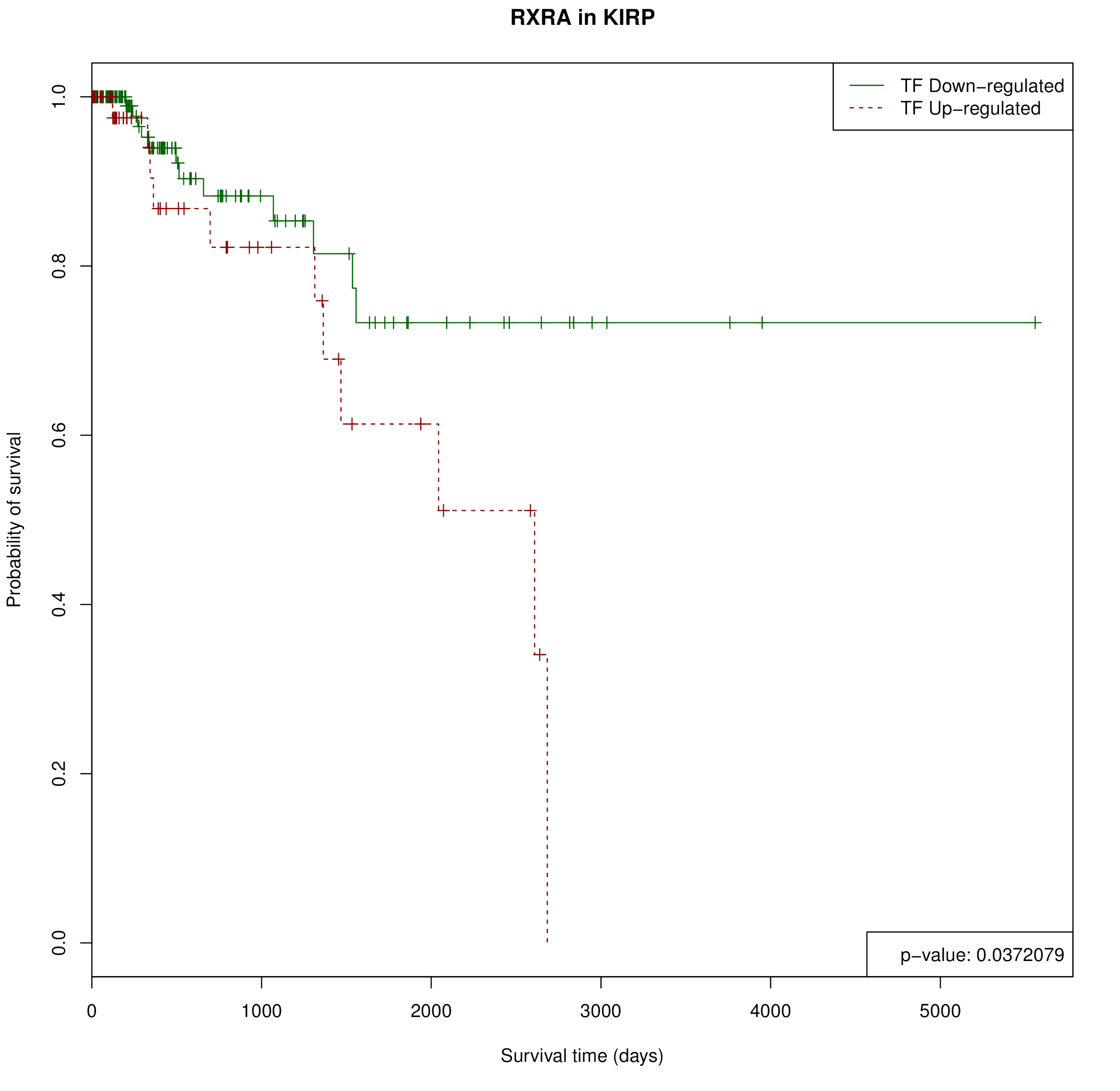
RXRA in KIRP
TF Down−regulated
TF Up−regulated
p−value: 0.0372079
Probability of survival
Survival time (days)
0.0
0.2
0.4
0.6
0.8
1.0
0
1000
2000
3000
4000
5000

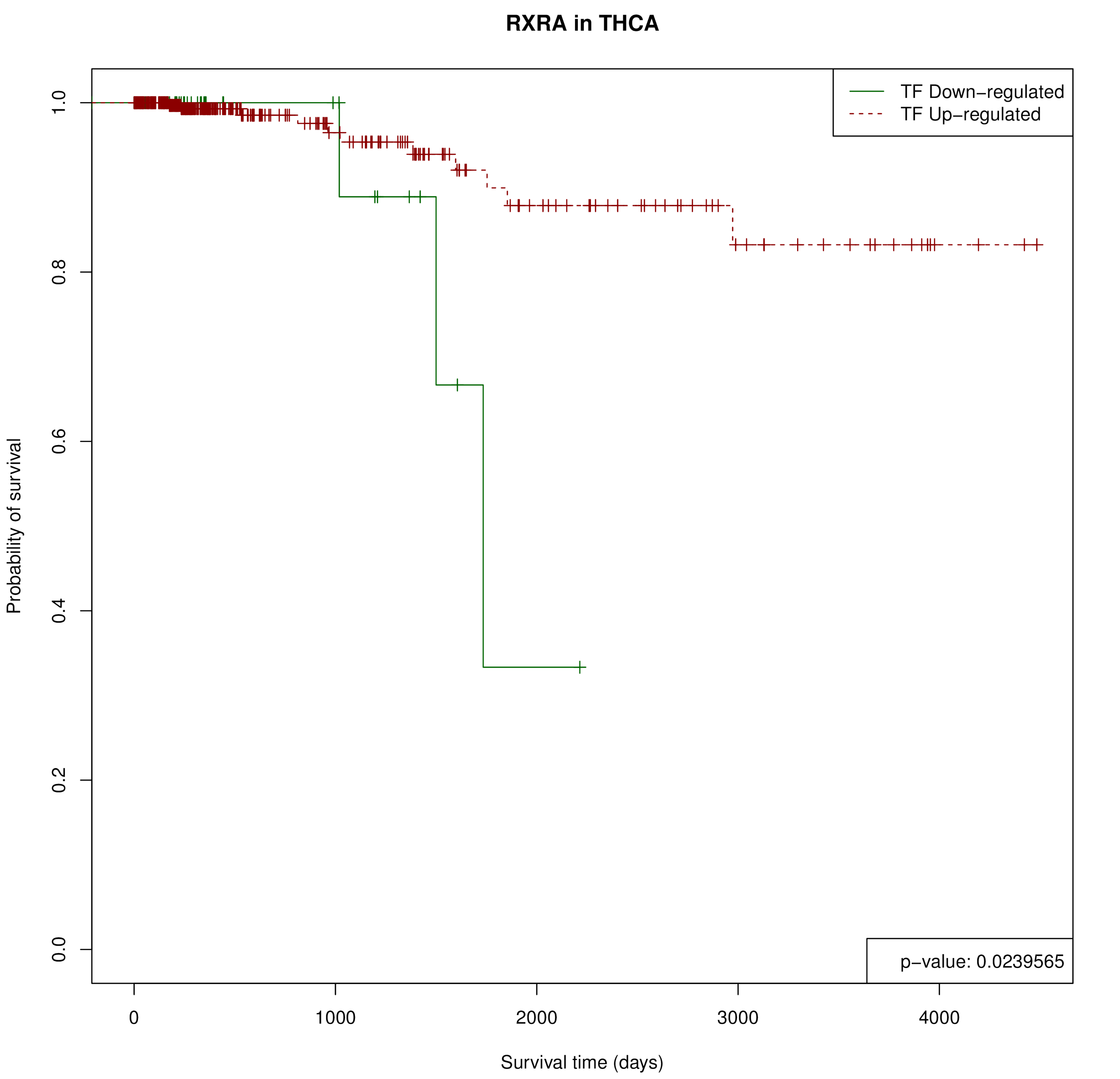
RXRA in THCA
TF Down−regulated
TF Up−regulated
1.0
0.8
0.6
0.4
0.2
0.0
Probability of survival
0
1000
2000
3000
4000
Survival time (days)
p−value: 0.0239565

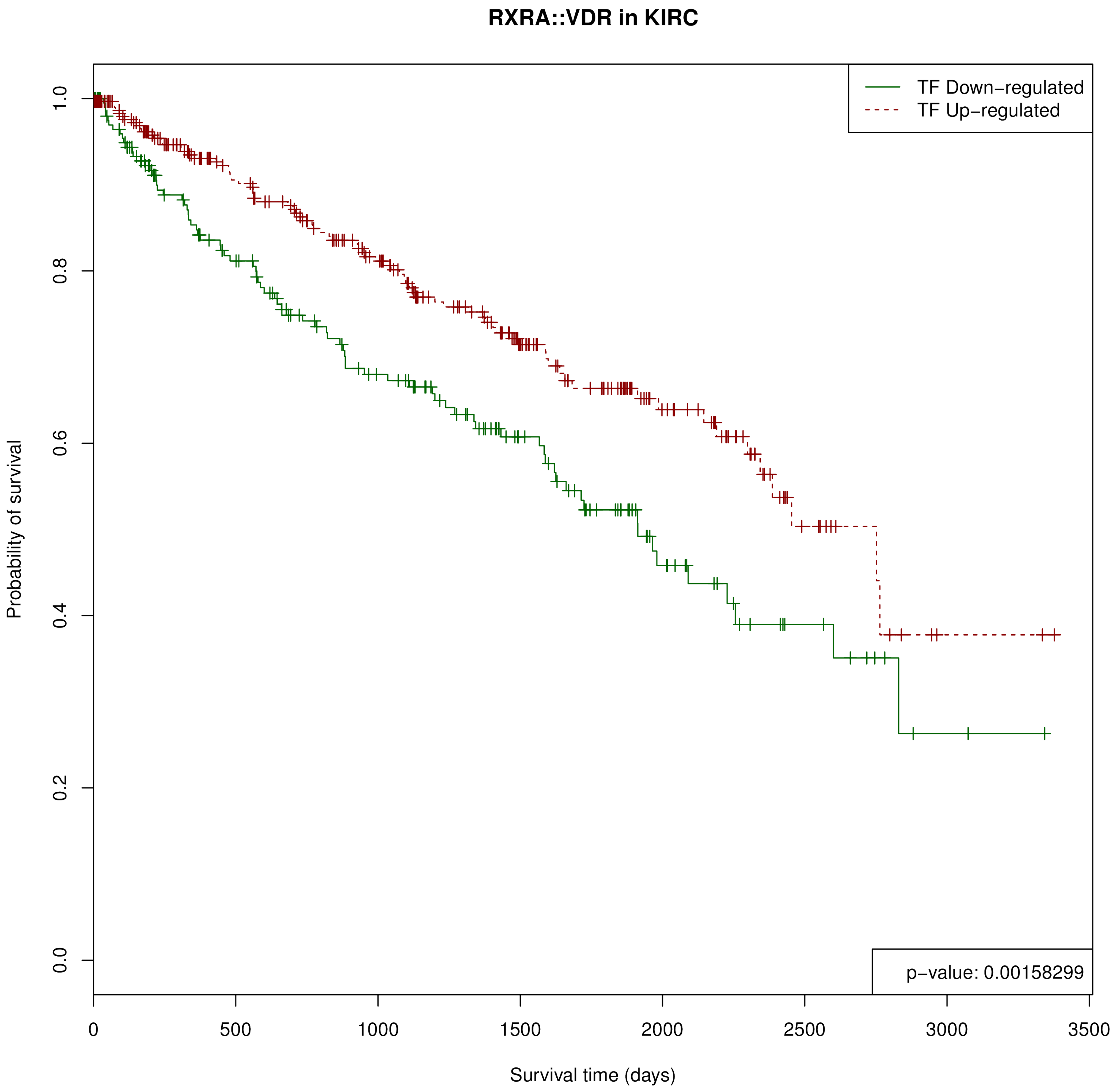

RXRA::VDR in KIRC
TF Down−regulated
TF Up−regulated
Probability of survival
0.0
0.2
0.4
0.6
0.8
1.0
p−value: 0.00158299
0
500
1000
1500
2000
2500
3000
3500
Survival time (days)

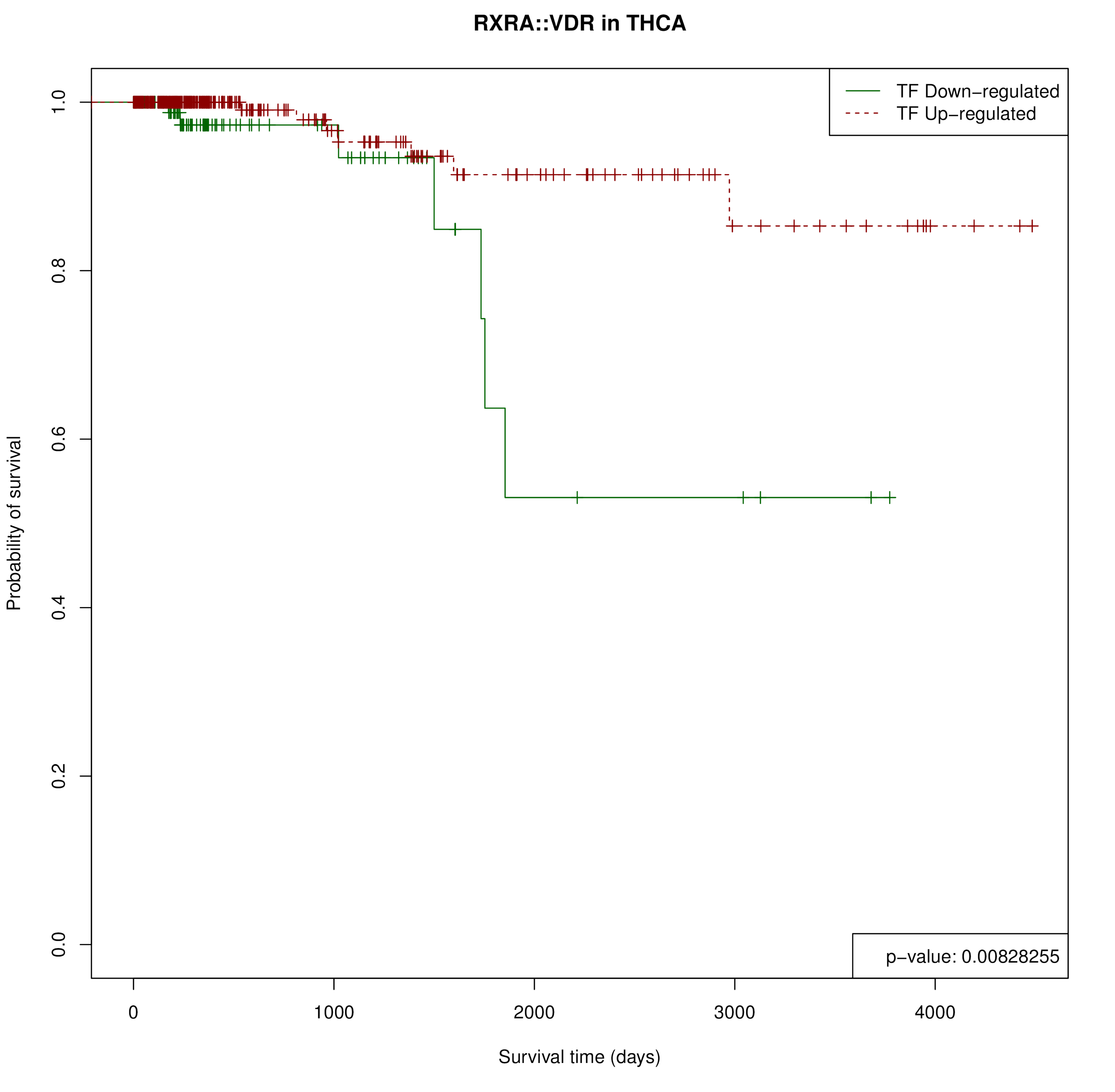
RXRA::VDR in THCA
TF Down−regulated
TF Up−regulated
p−value: 0.00828255
Probability of survival
0.0
0.2
0.4
0.6
0.8
1.0
Survival time (days)
0
1000
2000
3000
4000

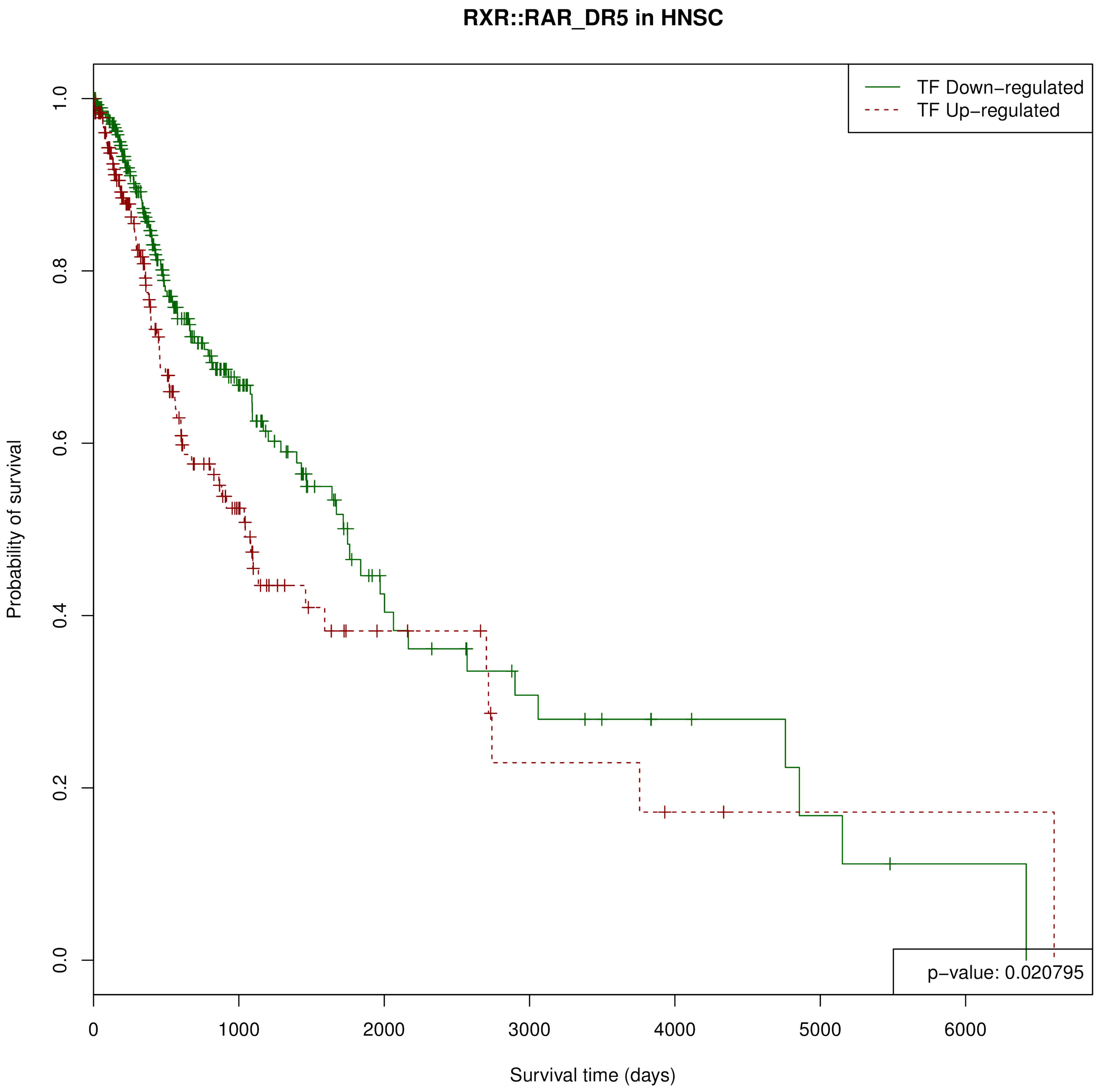
RXR::RAR_DR5 in HNSC
TF Down−regulated
TF Up−regulated
p−value: 0.020795
Probability of survival
Survival time (days)
0.0
0.2
0.4
0.6
0.8
1.0
0
1000
2000
3000
4000
5000
6000

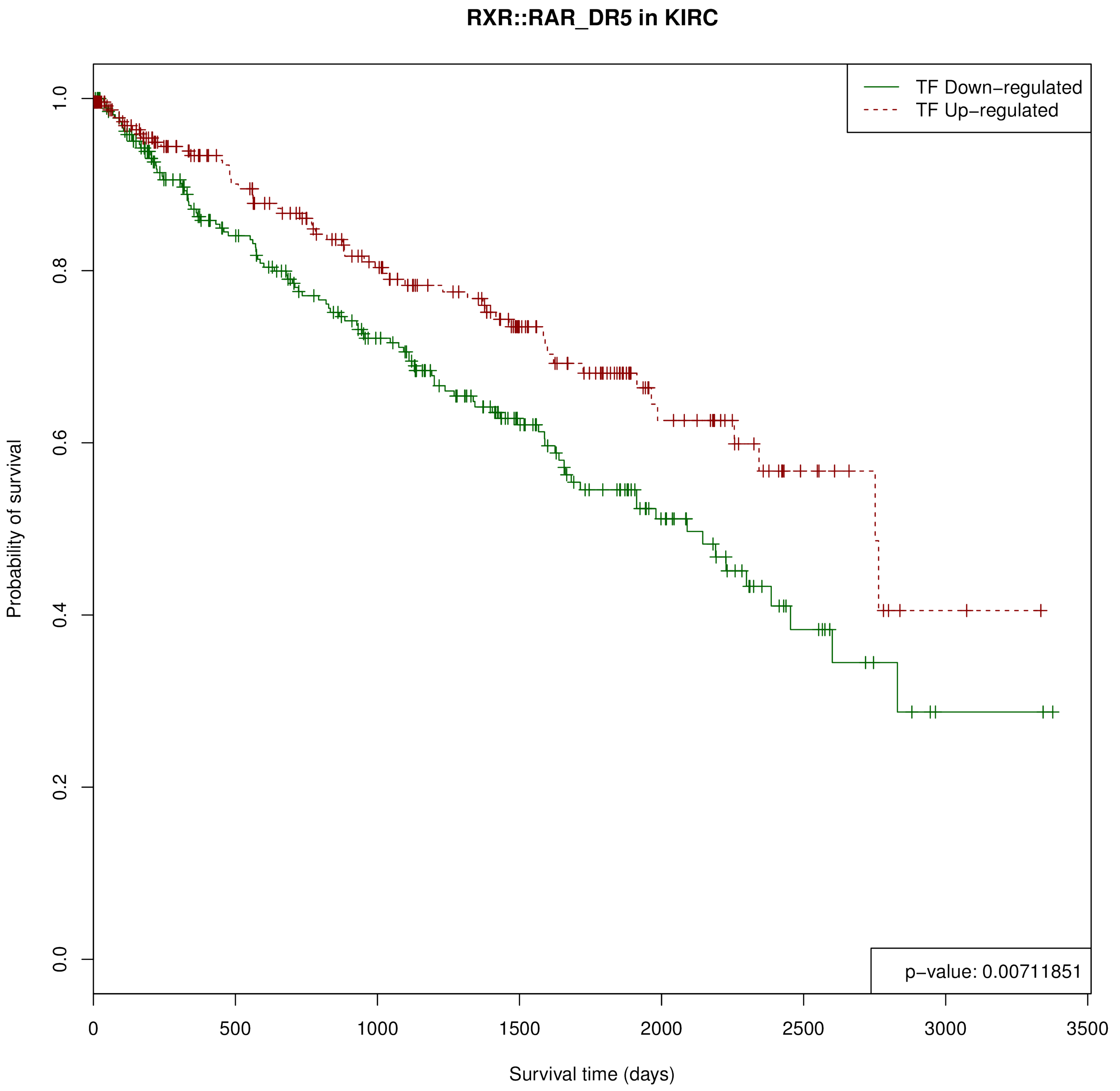
RXR::RAR_DR5 in KIRC
TF Down−regulated
TF Up−regulated
Probability of survival
0.0
0.2
0.4
0.6
0.8
1.0
Survival time (days)
0
500
1000
1500
2000
2500
3000
3500
p−value: 0.00711851

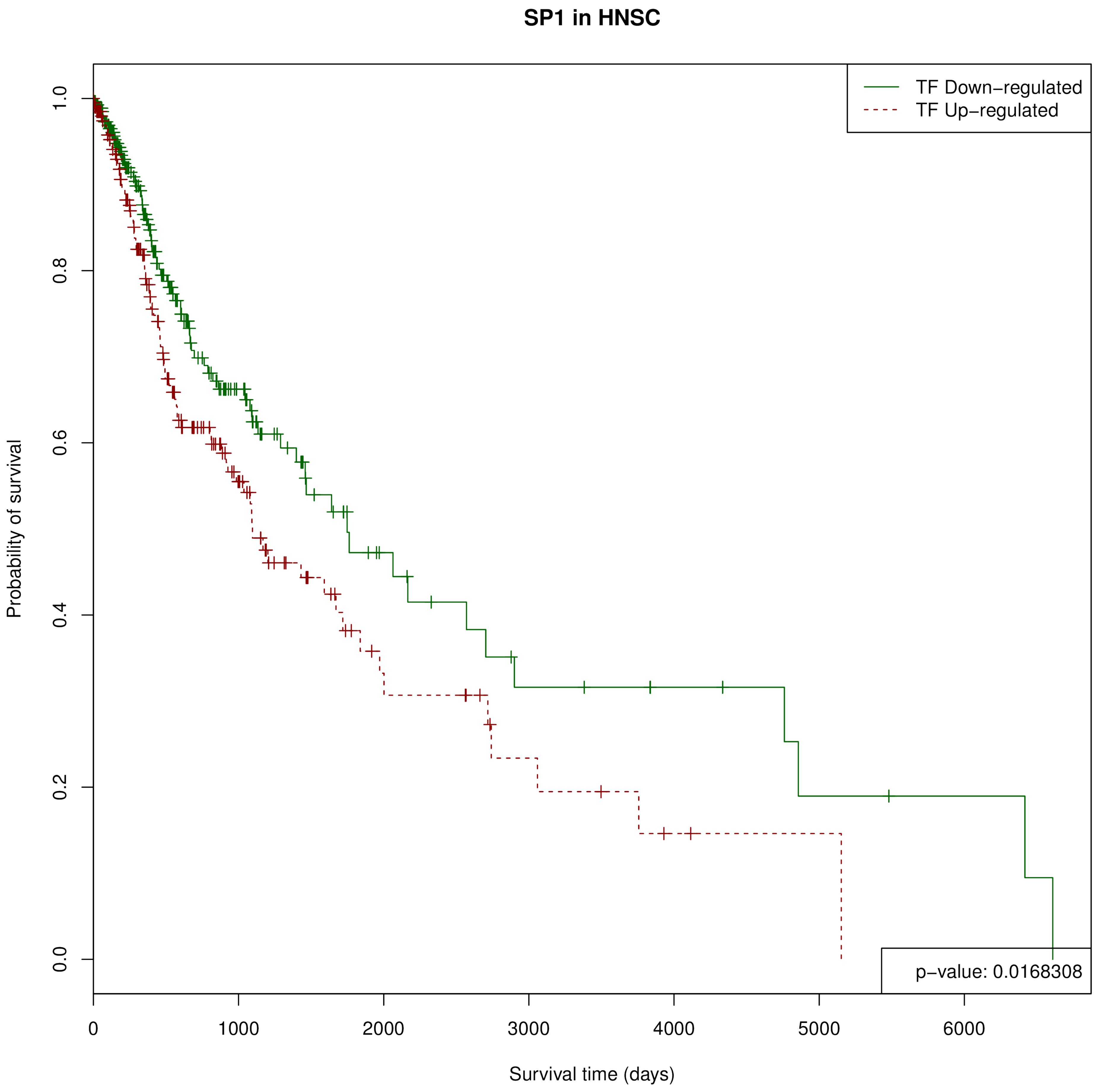
SP1 in HNSC
TF Down−regulated
TF Up−regulated
p−value: 0.0168308
Probability of survival
Survival time (days)
0.0
0.2
0.4
0.6
0.8
1.0
0
1000
2000
3000
4000
5000
6000

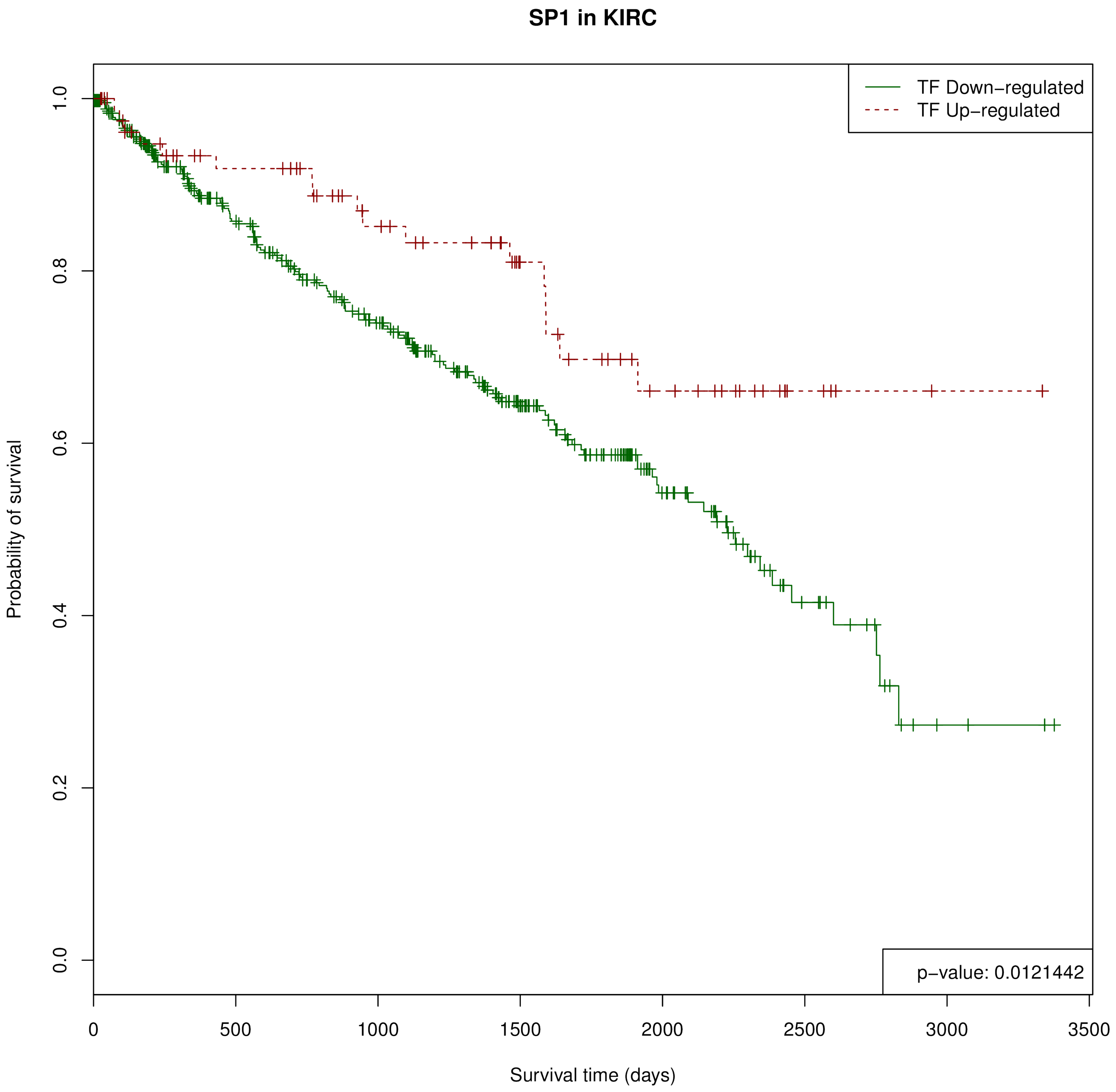

SP1 in KIRC
TF Down−regulated
TF Up−regulated
p−value: 0.0121442
Probability of survival
0.0
0.2
0.4
0.6
0.8
1.0
Survival time (days)
0
500
1000
1500
2000
2500
3000
3500

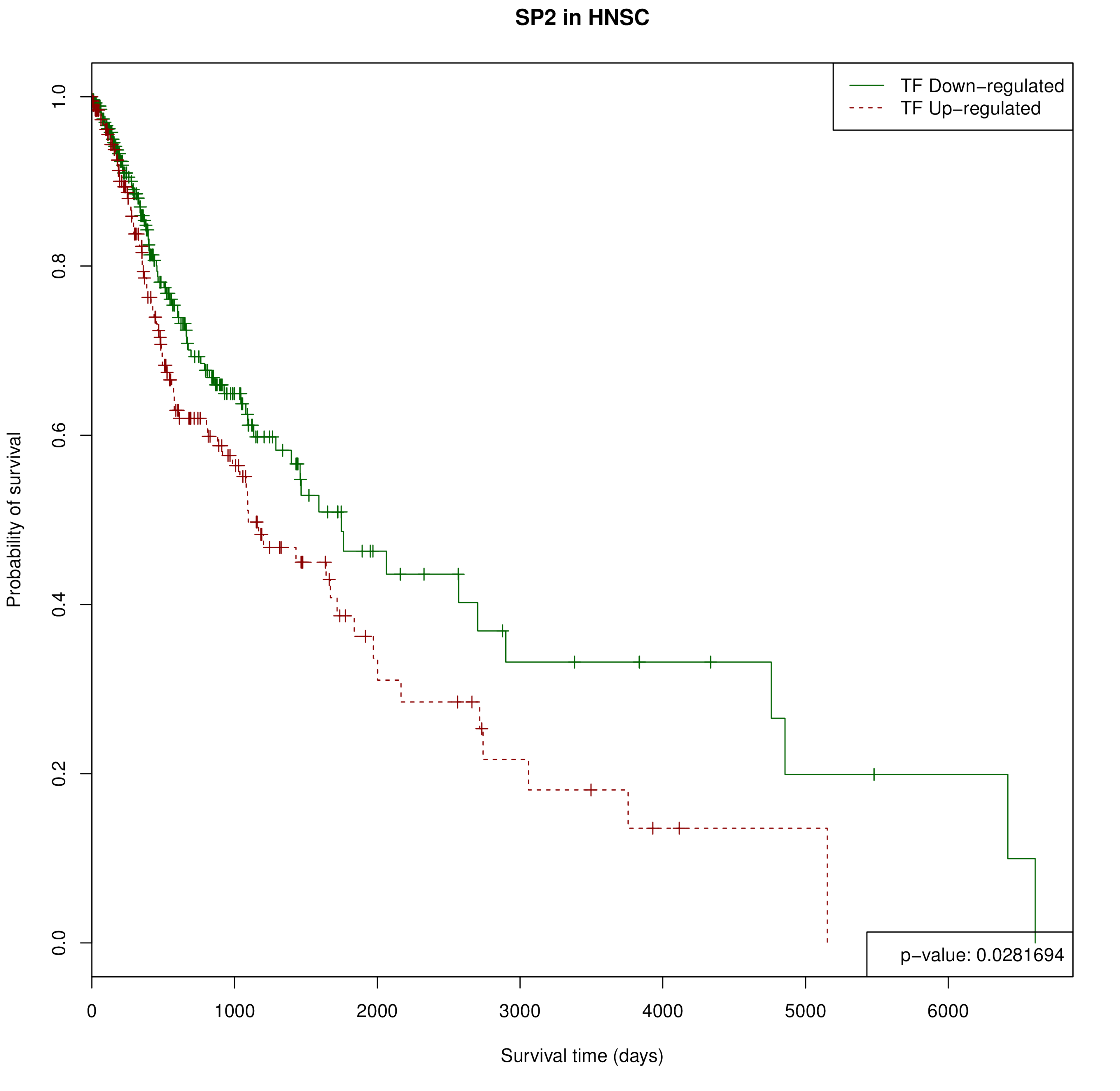

SP2 in HNSC
TF Down−regulated
TF Up−regulated
Probability of survival
0.0
0.2
0.4
0.6
0.8
1.0
0
1000
2000
3000
4000
5000
6000
Survival time (days)
p−value: 0.0281694

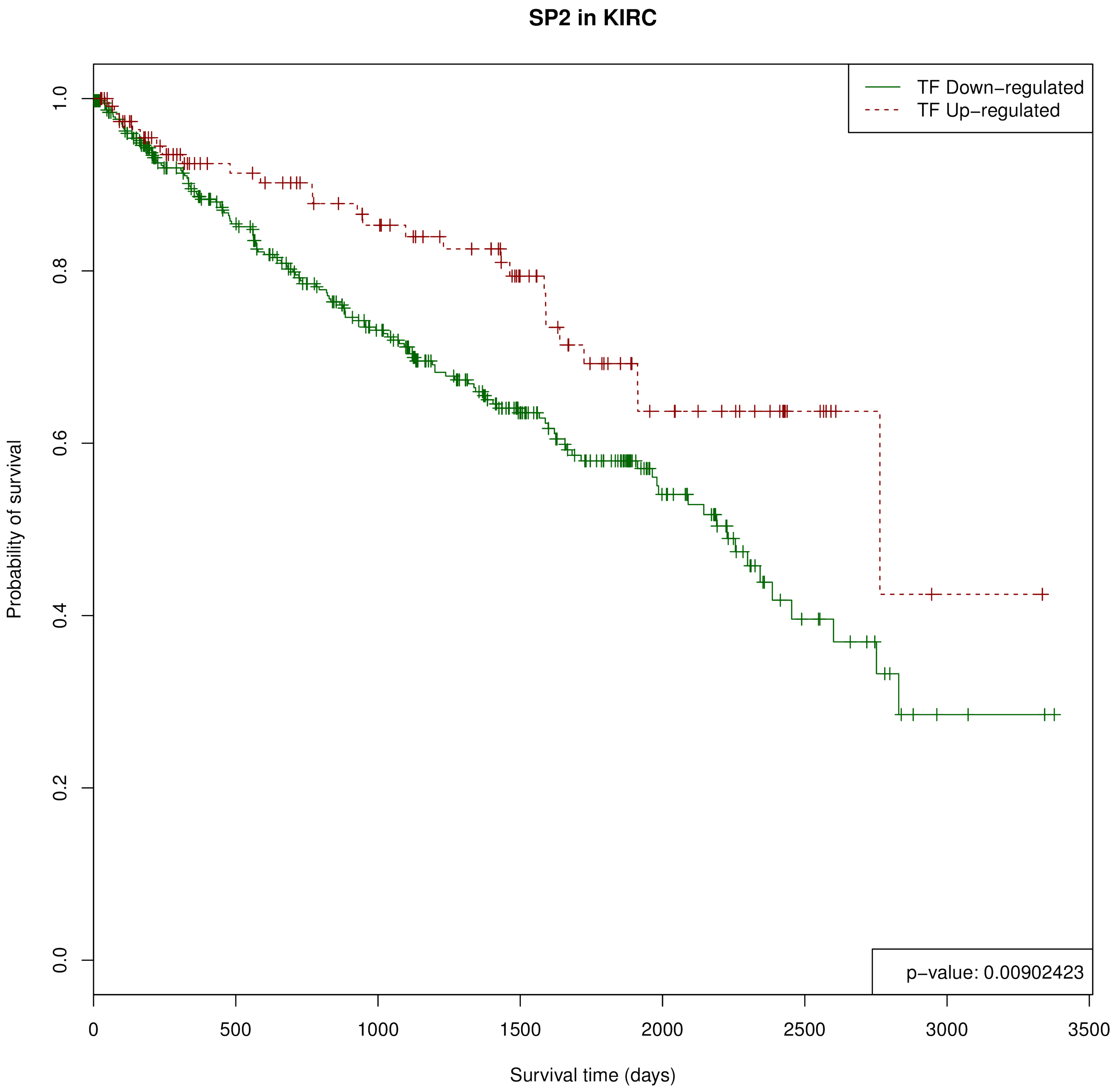
SP2 in KIRC
TF Down−regulated
TF Up−regulated
p−value: 0.00902423
Probability of survival
Survival time (days)
0.0
0.2
0.4
0.6
0.8
1.0
0
500
1000
1500
2000
2500
3000
3500

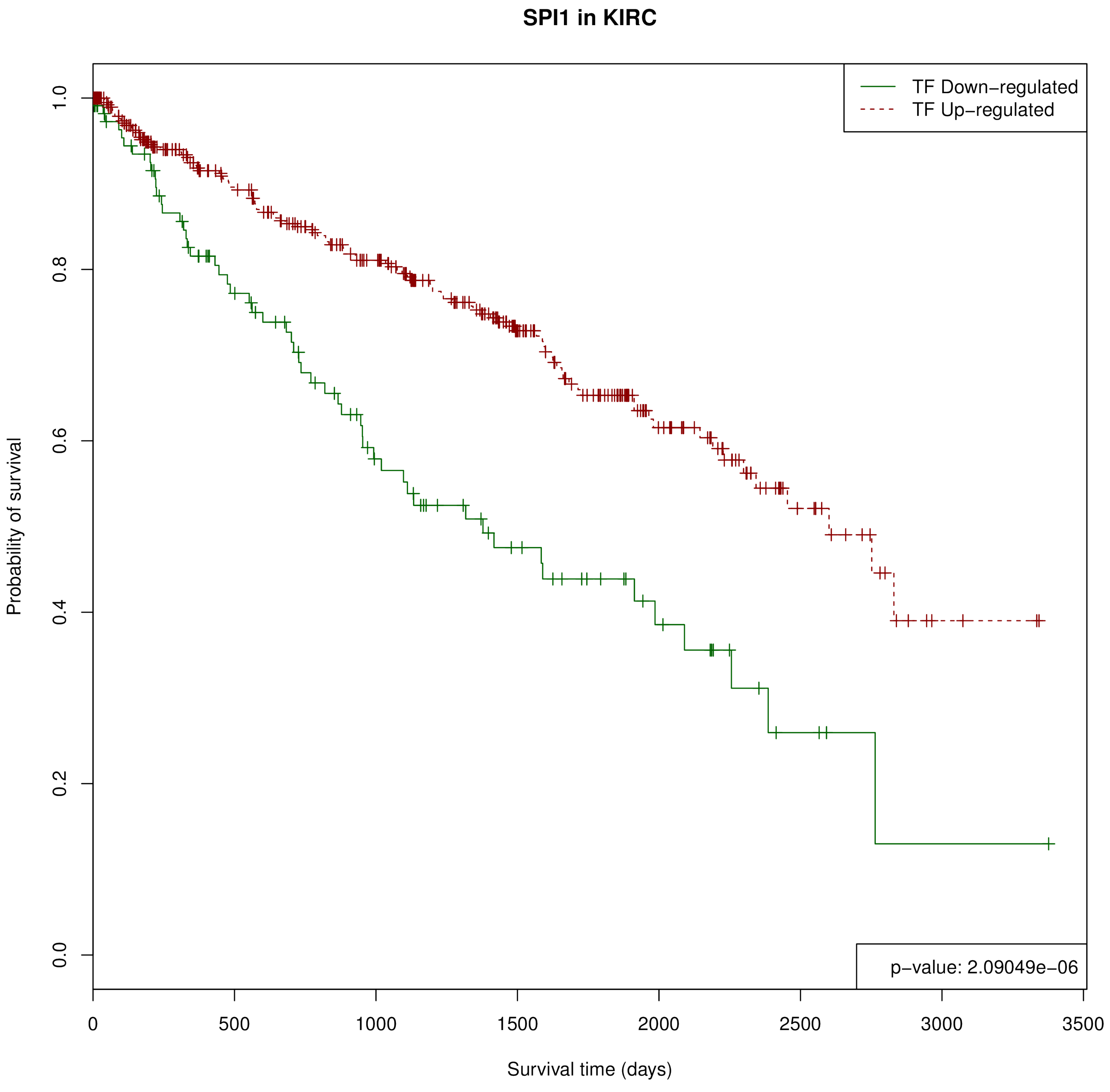
SPI1 in KIRC
TF Down−regulated
TF Up−regulated
p−value: 2.09049e−06
Probability of survival
Survival time (days)
0.0
0.2
0.4
0.6
0.8
1.0
0
500
1000
1500
2000
2500
3000
3500

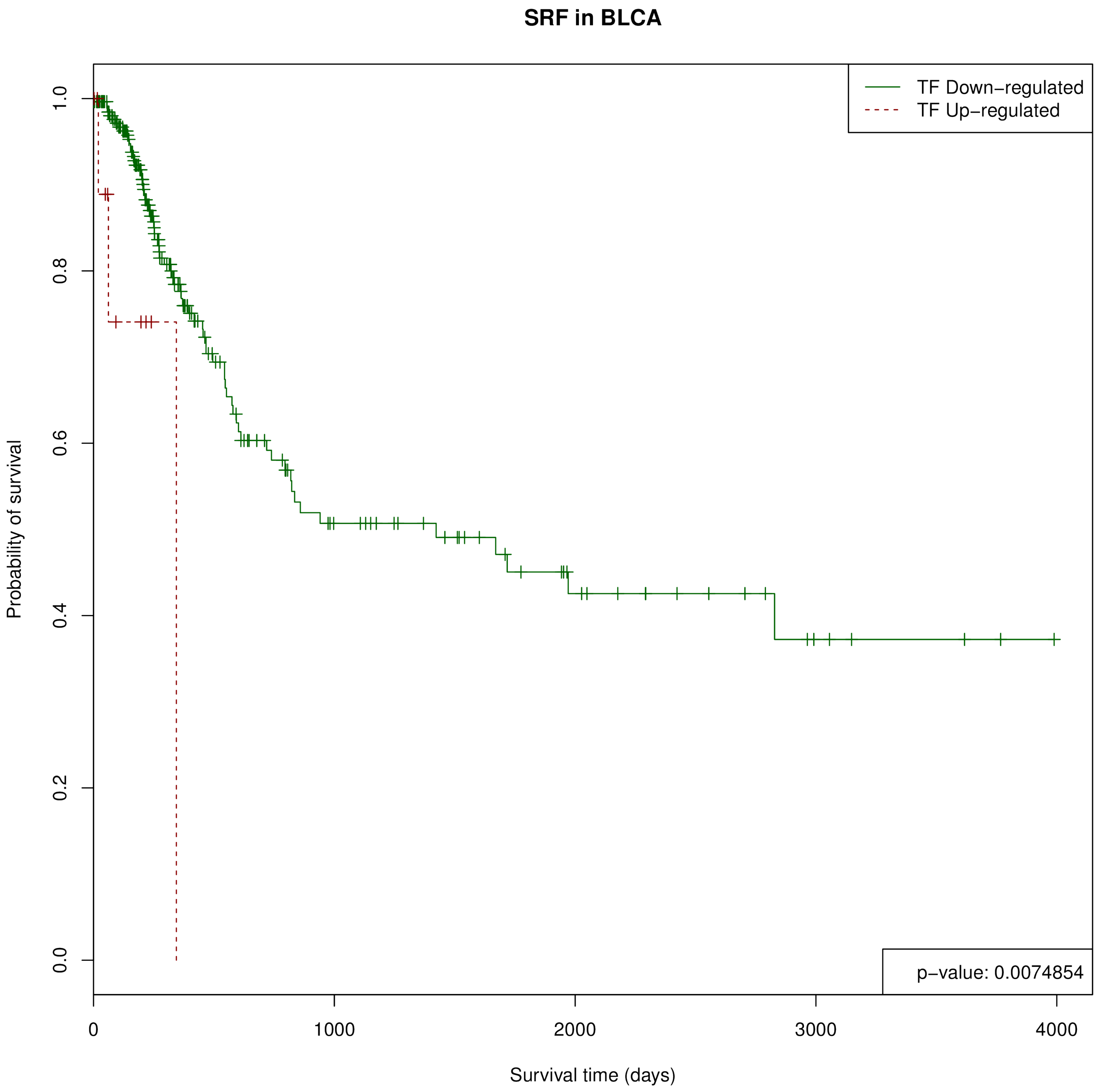

SRF in BLCA
TF Down−regulated
TF Up−regulated
Probability of survival
0.0
0.2
0.4
0.6
0.8
1.0
0
1000
2000
3000
4000
Survival time (days)
p−value: 0.0074854

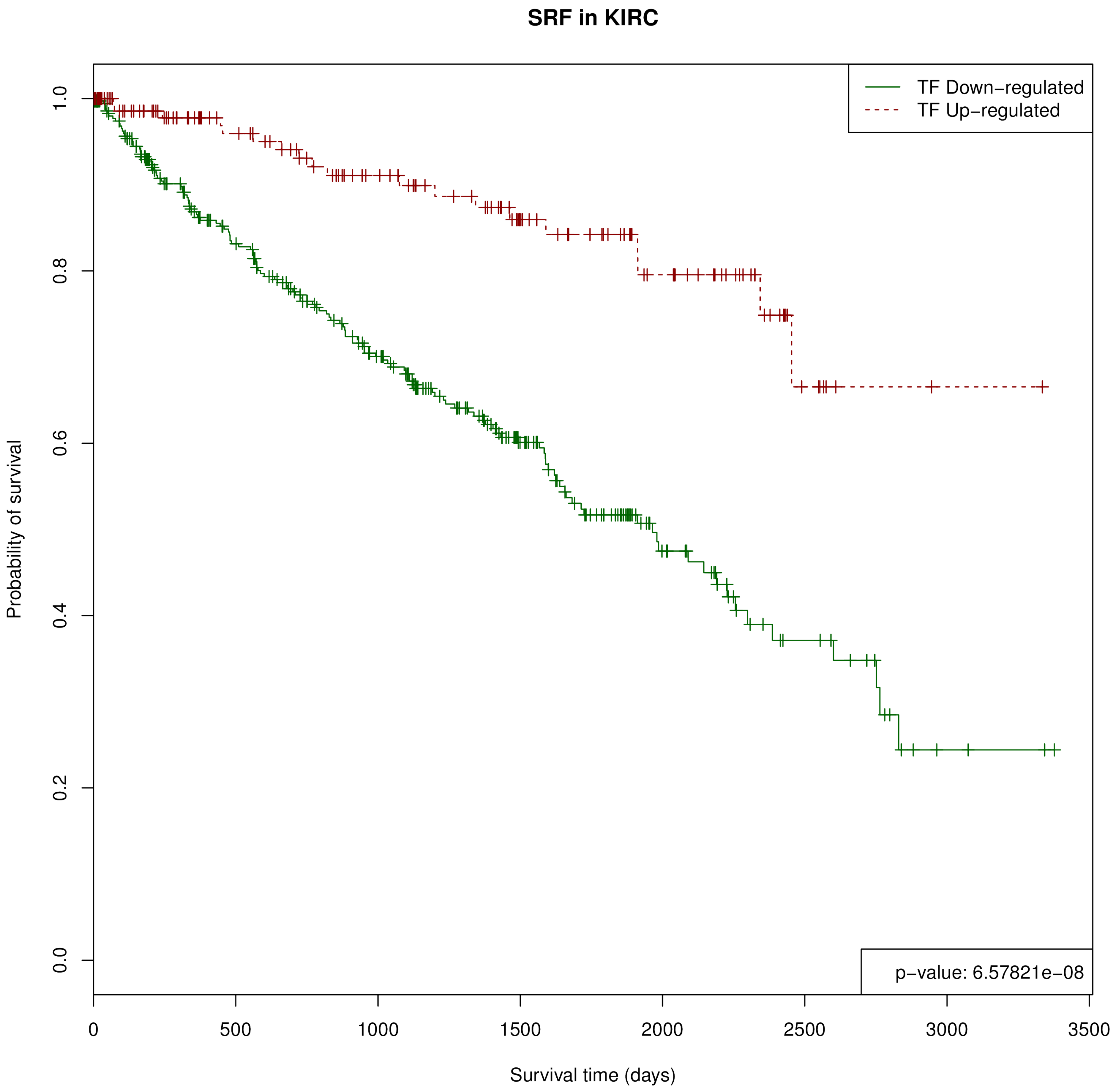
SRF in KIRC
TF Down−regulated
TF Up−regulated
p−value: 6.57821e−08
Probability of survival
0.0
0.2
0.4
0.6
0.8
1.0
0
500
1000
1500
2000
2500
3000
3500
Survival time (days)

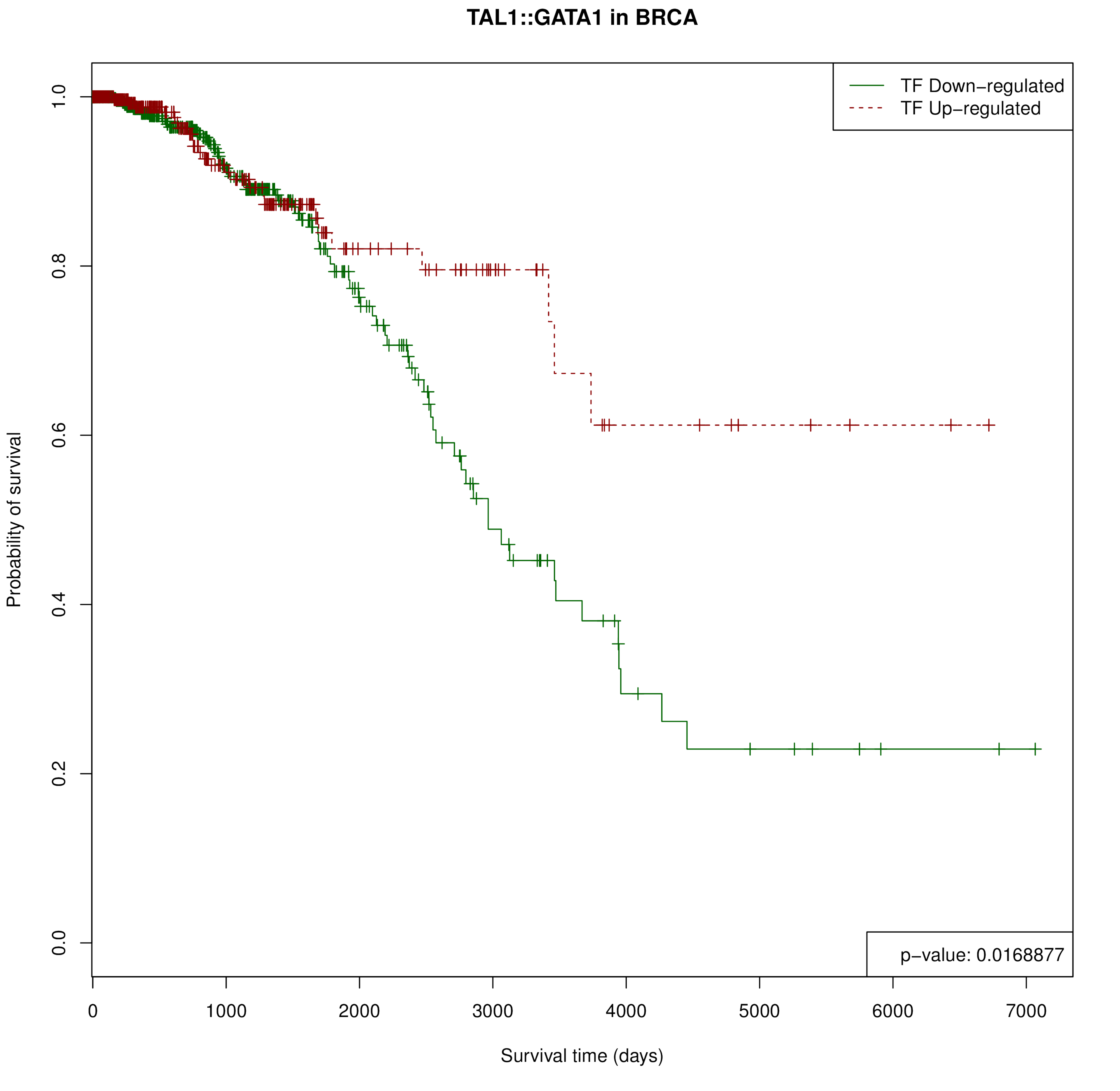
TAL1::GATA1 in BRCA
TF Down−regulated
TF Up−regulated
p−value: 0.0168877
Probability of survival
Survival time (days)
0.0
0.2
0.4
0.6
0.8
1.0
0
1000
2000
3000
4000
5000
6000
7000

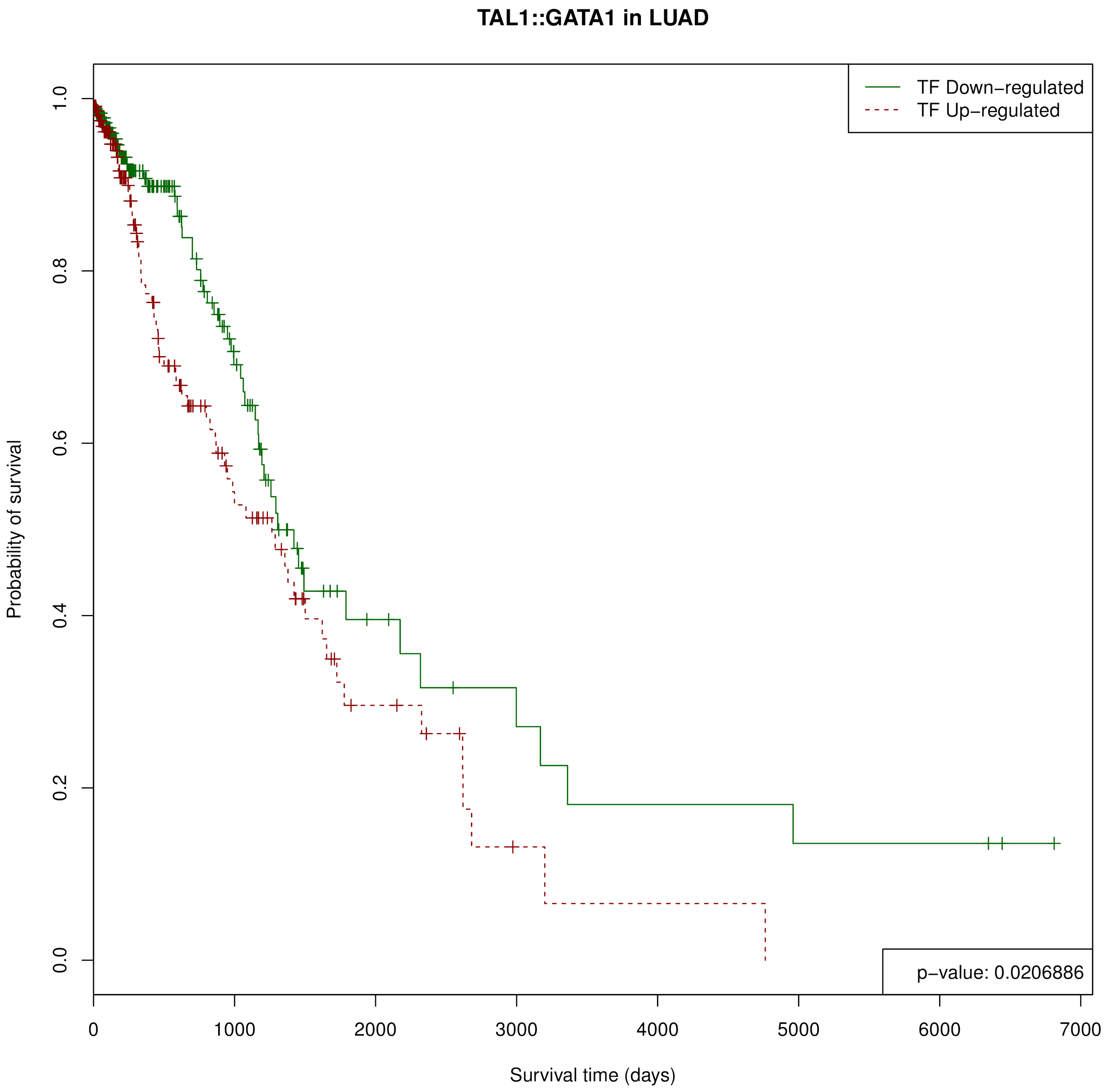

TAL1::GATA1 in LUAD
TF Down−regulated
TF Up−regulated
p−value: 0.0206886
Probability of survival
Survival time (days)
0.0
0.2
0.4
0.6
0.8
1.0
0
1000
2000
3000
4000
5000
6000
7000

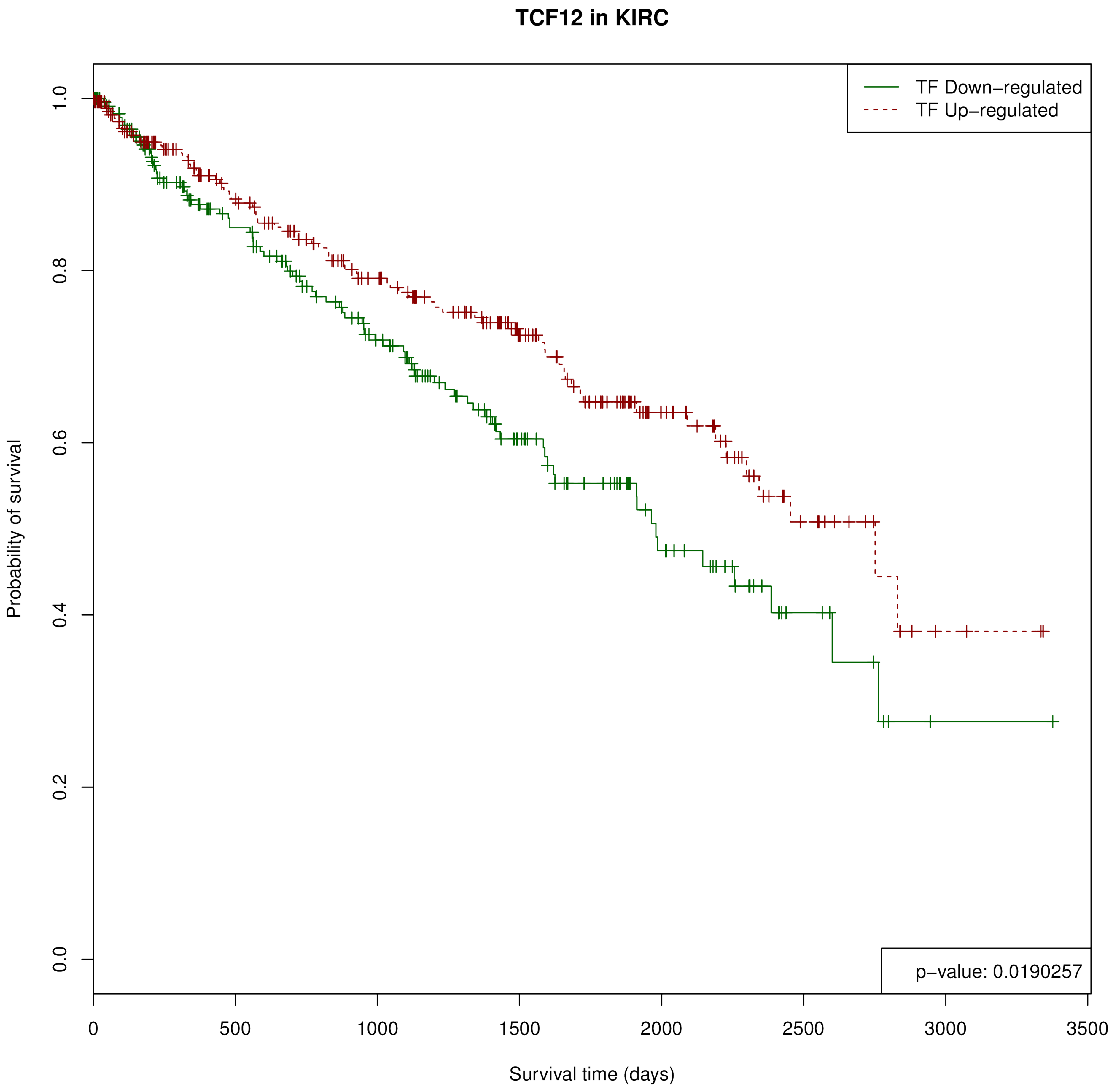

TCF12 in KIRC
TF Down−regulated
TF Up−regulated
Probability of survival
0.0
0.2
0.4
0.6
0.8
1.0
0
500
1000
1500
2000
2500
3000
3500
Survival time (days)
p−value: 0.0190257

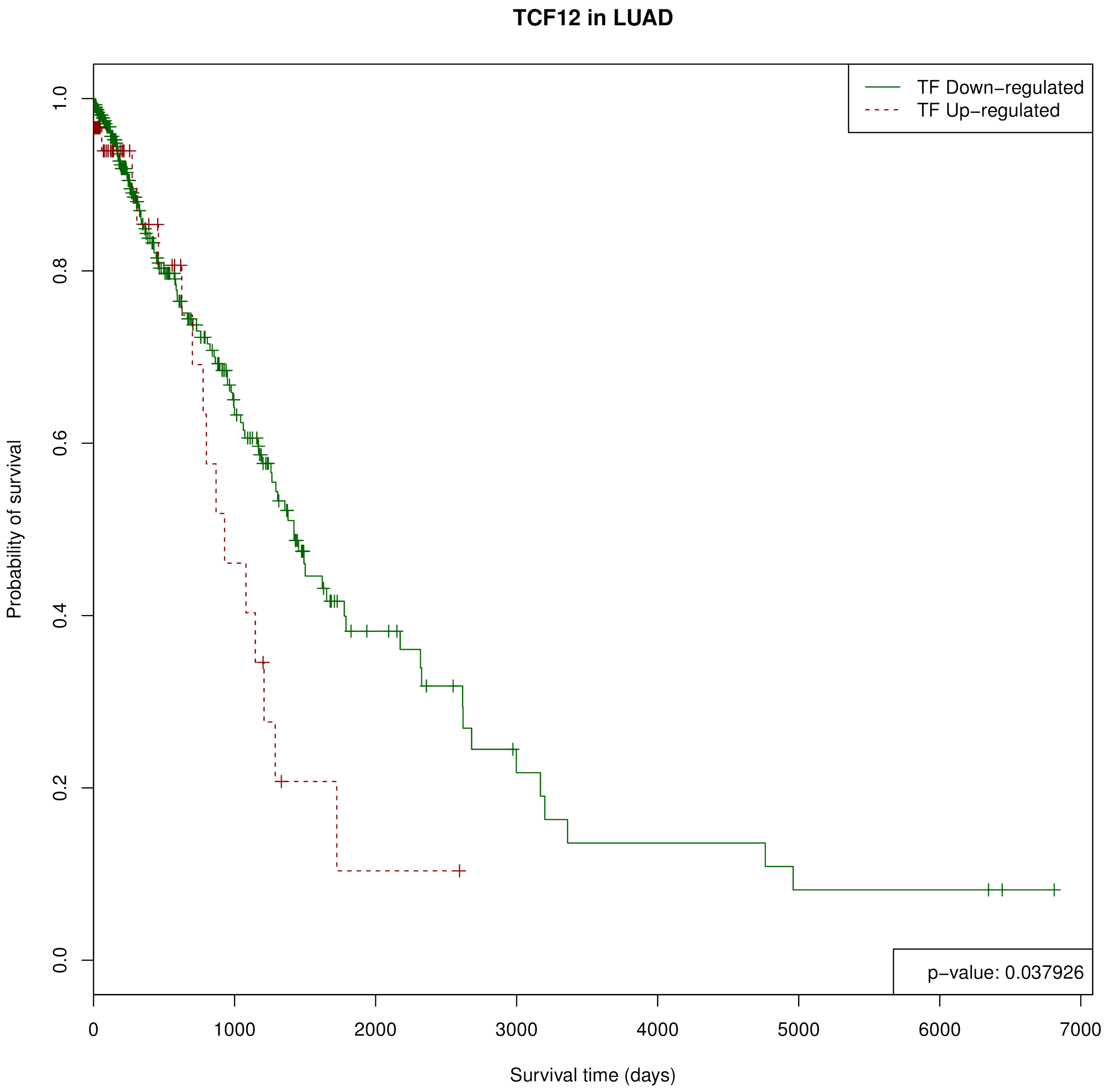

TCF12 in LUAD
TF Down−regulated
TF Up−regulated
Probability of survival
0.0
0.2
0.4
0.6
0.8
1.0
Survival time (days)
0
1000
2000
3000
4000
5000
6000
7000
p−value: 0.037926

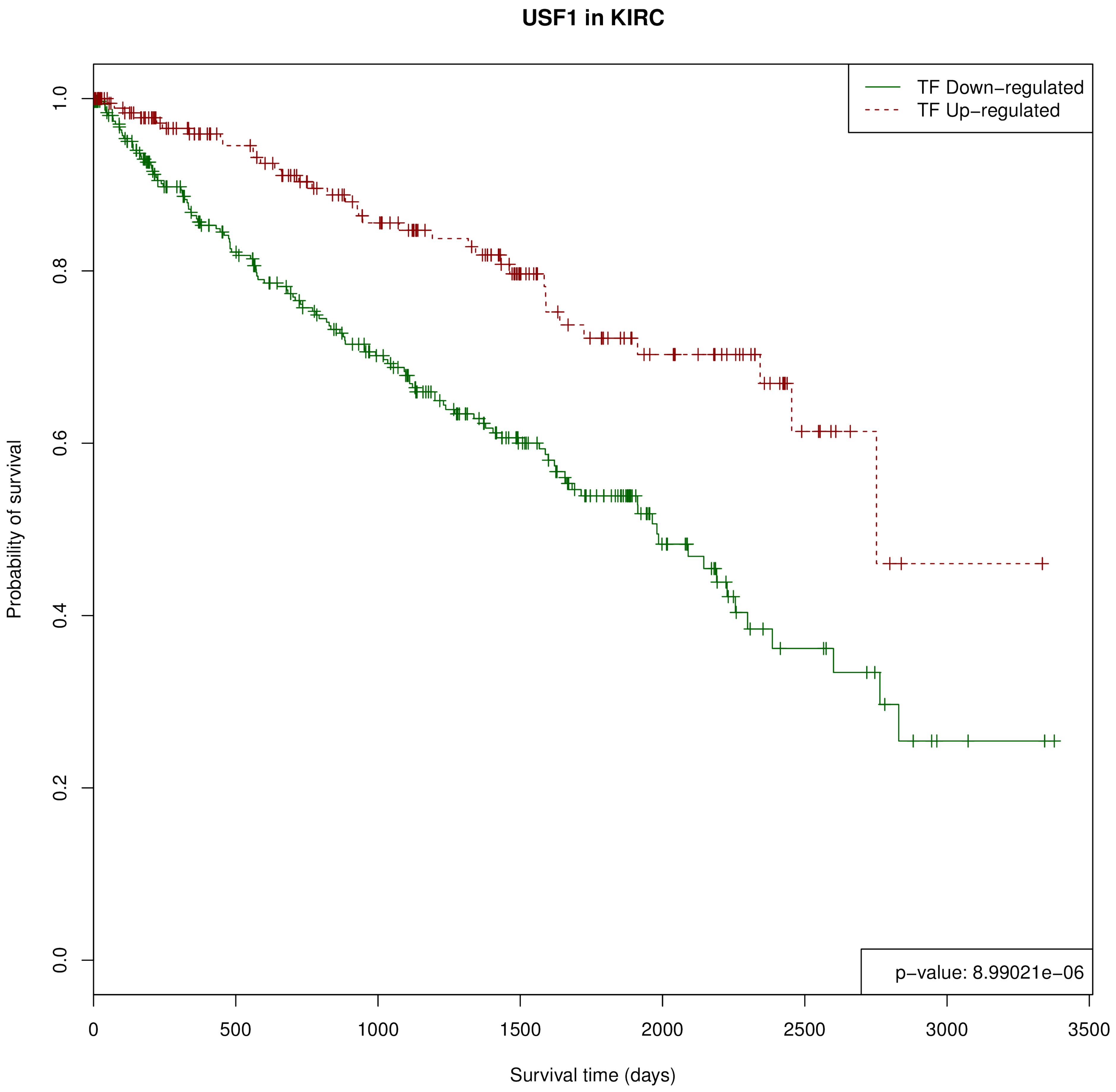
USF1 in KIRC
TF Down−regulated
TF Up−regulated
p−value: 8.99021e−06
Probability of survival
Survival time (days)
0.0
0.2
0.4
0.6
0.8
1.0
0
500
1000
1500
2000
2500
3000
3500

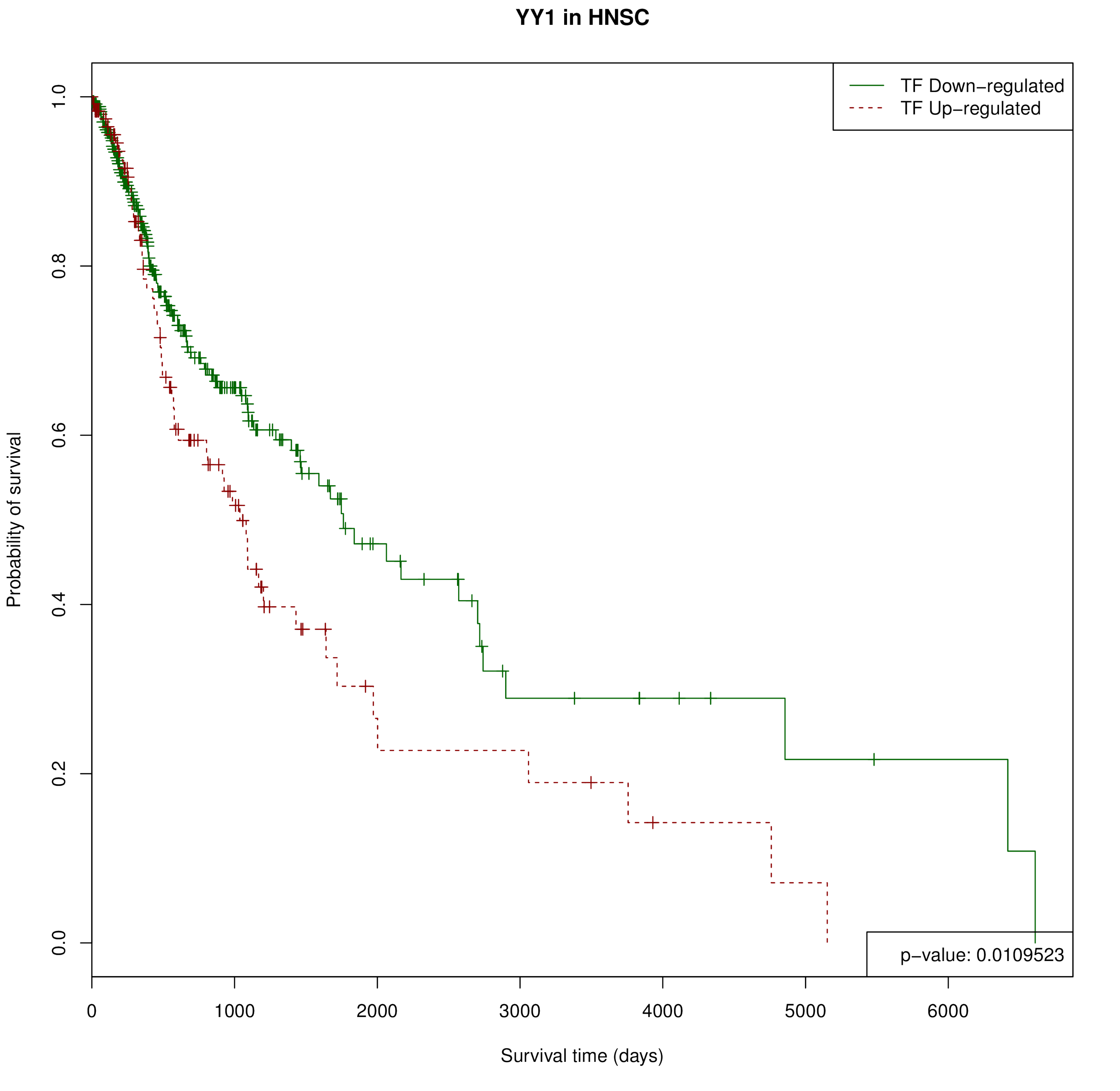
YY1 in HNSC
TF Down−regulated
TF Up−regulated
Probability of survival
0.0
0.2
0.4
0.6
0.8
1.0
0
1000
2000
3000
4000
5000
6000
Survival time (days)
p−value: 0.0109523

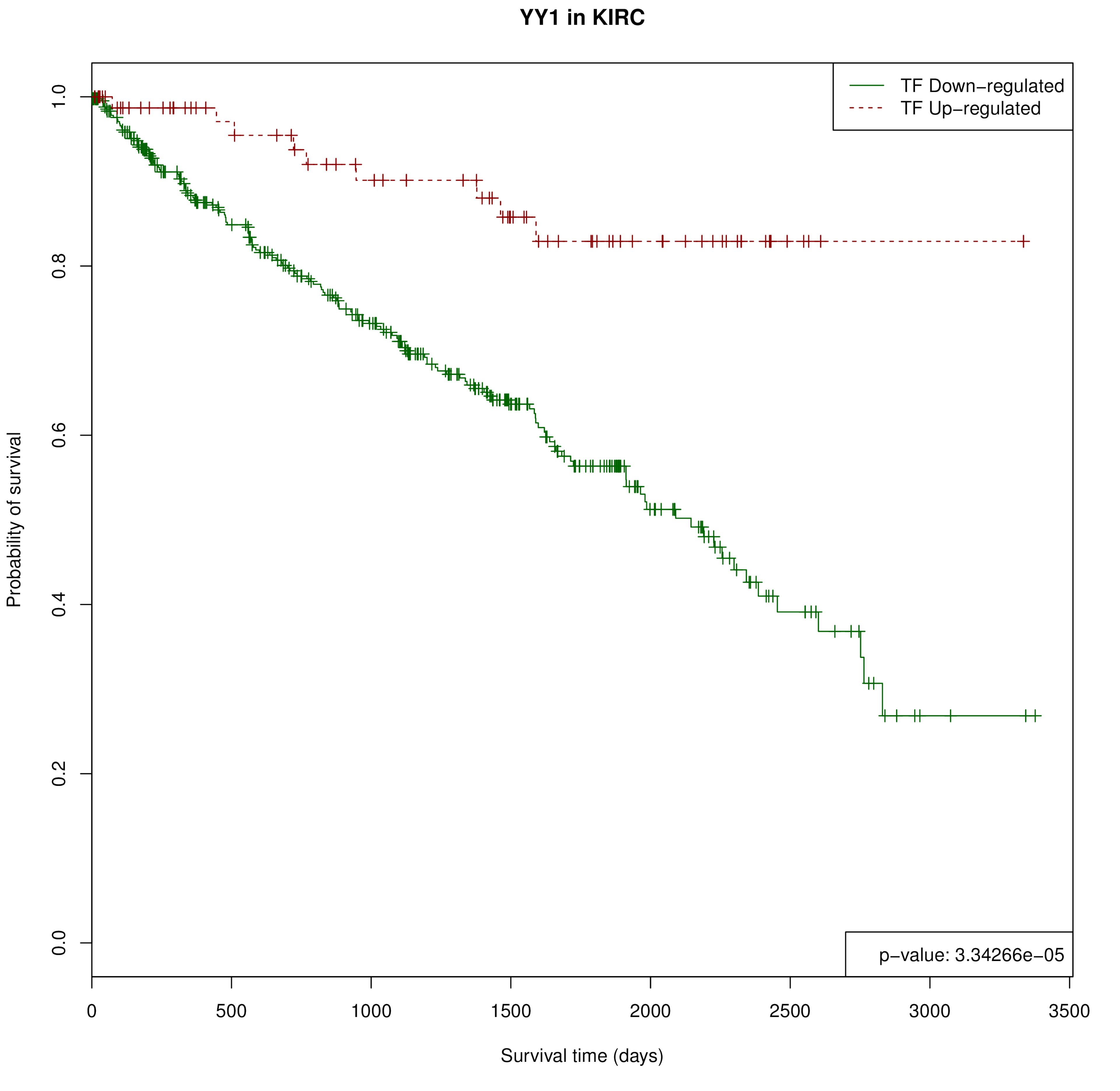

YY1 in KIRC
TF Down−regulated
TF Up−regulated
p−value: 3.34266e−05
Probability of survival
Survival time (days)
0.0
0.2
0.4
0.6
0.8
1.0
0
500
1000
1500
2000
2500
3000
3500

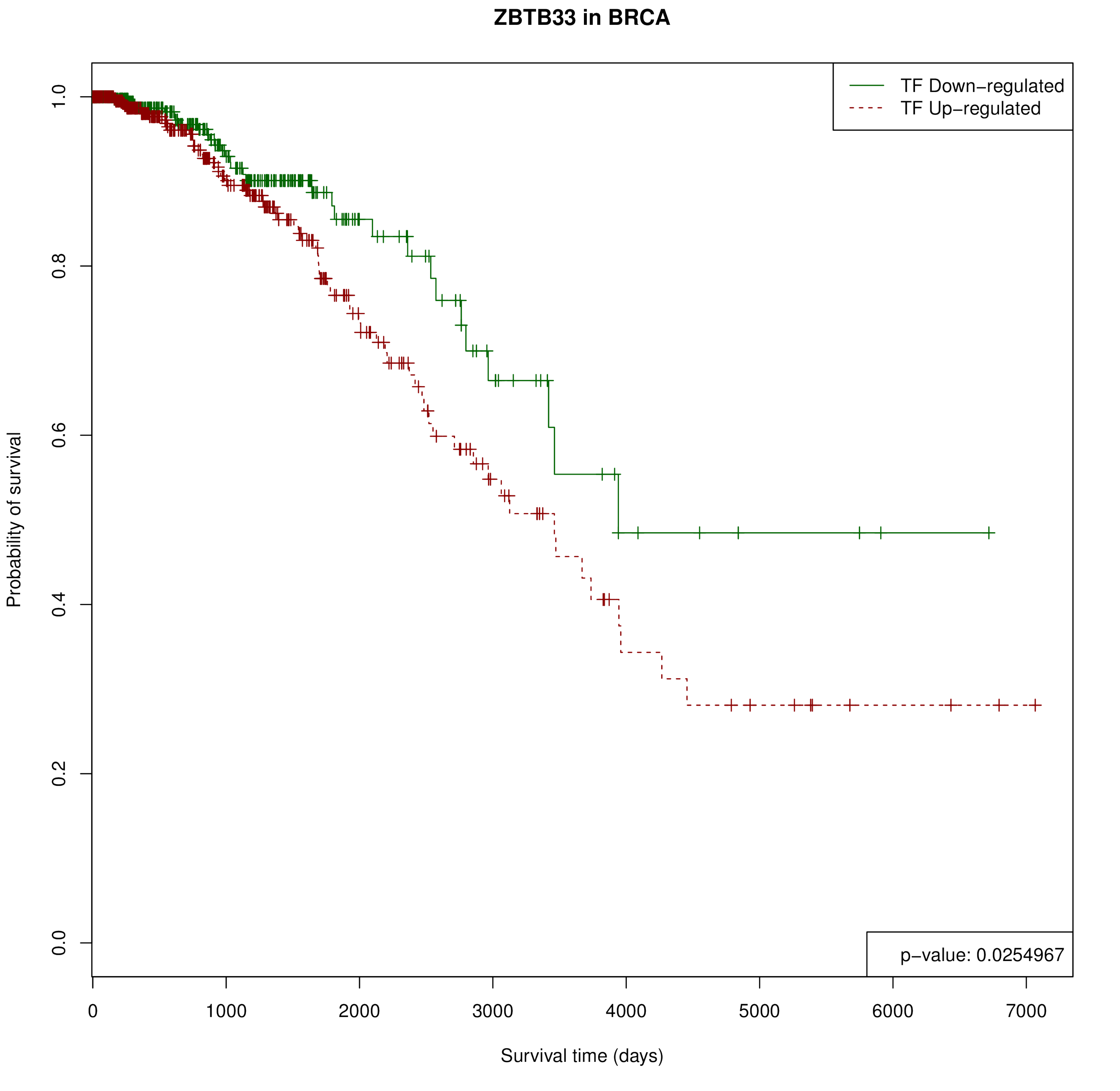
ZBTB33 in BRCA
TF Down−regulated
TF Up−regulated
p−value: 0.0254967
Probability of survival
Survival time (days)
0.0
0.2
0.4
0.6
0.8
1.0
0
1000
2000
3000
4000
5000
6000
7000

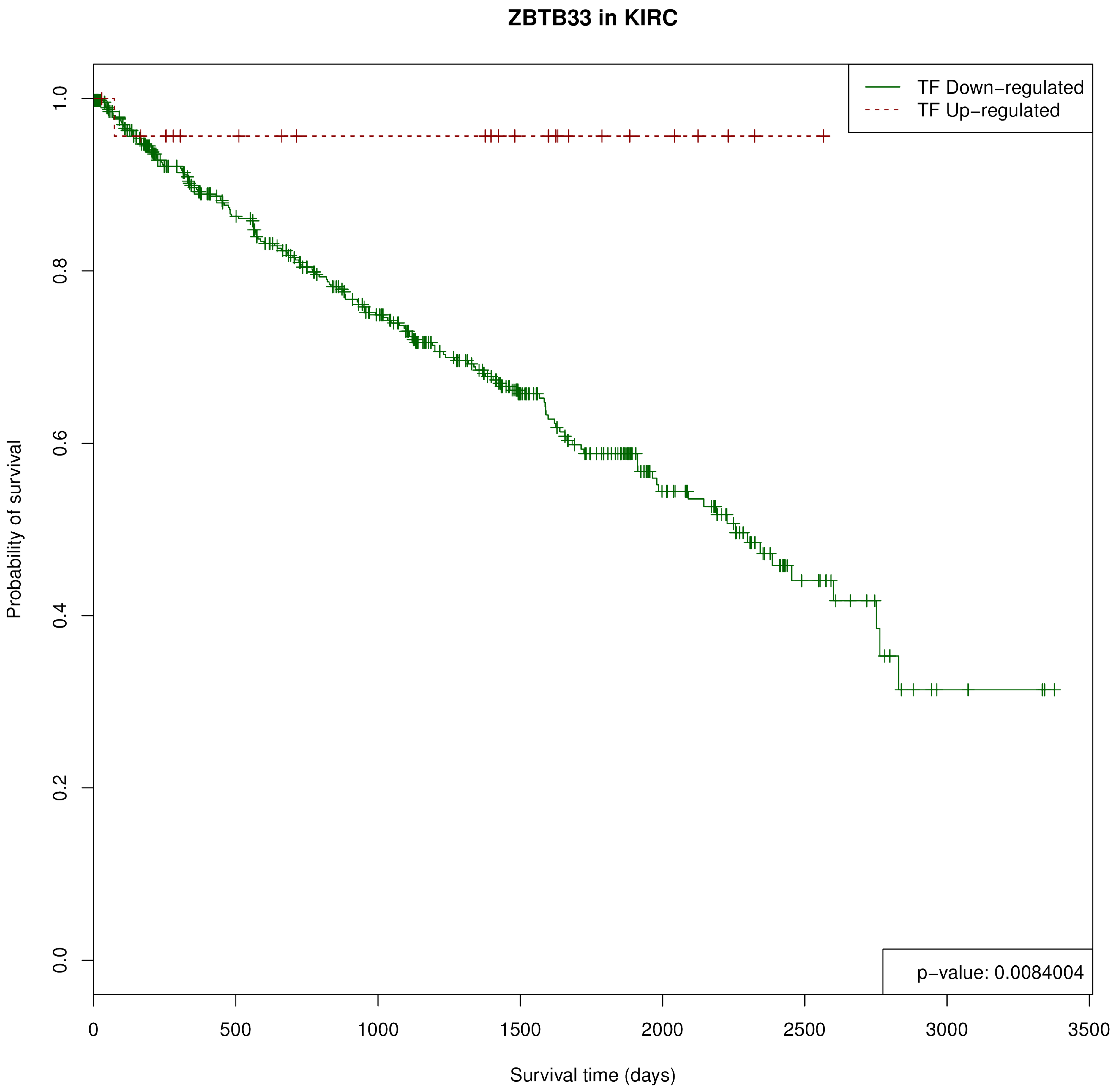
ZBTB33 in KIRC
TF Down−regulated
TF Up−regulated
p−value: 0.0084004
Probability of survival
Survival time (days)
0.0
0.2
0.4
0.6
0.8
1.0
0
500
1000
1500
2000
2500
3000
3500

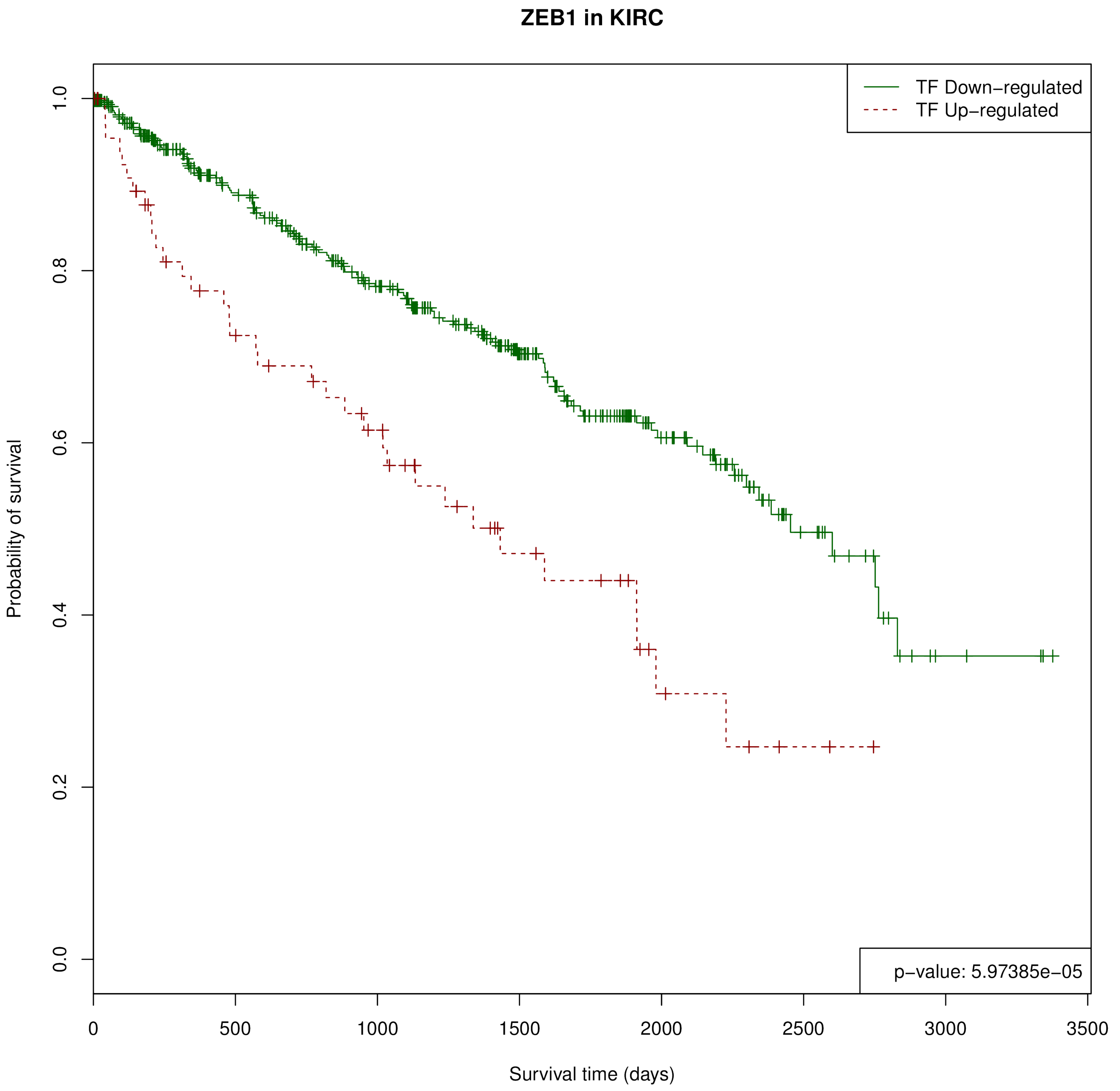

ZEB1 in KIRC
TF Down−regulated
TF Up−regulated
p−value: 5.97385e−05
Probability of survival
Survival time (days)
0.0
0.2
0.4
0.6
0.8
1.0
0
500
1000
1500
2000
2500
3000
3500

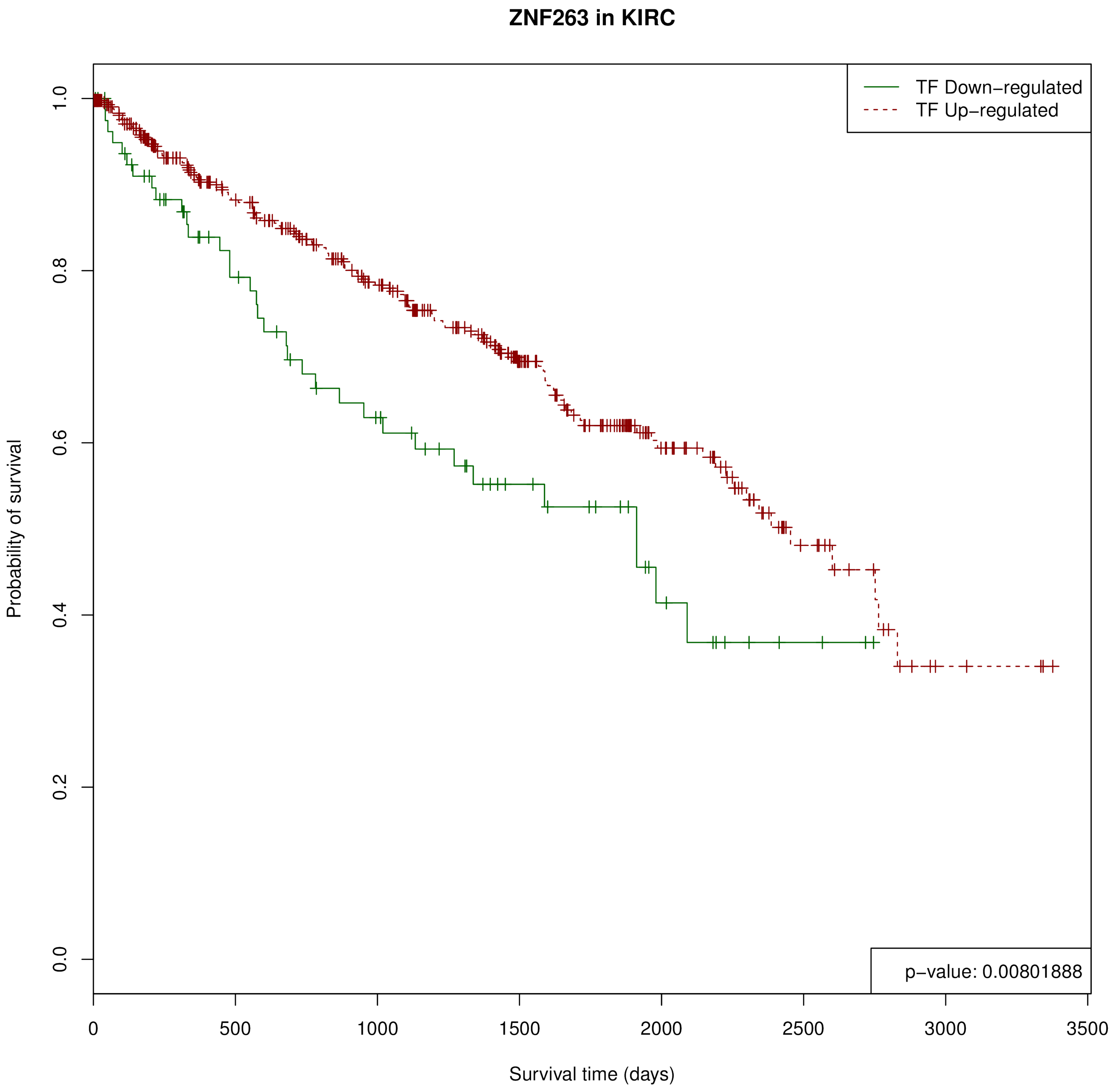

ZNF263 in KIRC
TF Down−regulated
TF Up−regulated
Probability of survival
0.0
0.2
0.4
0.6
0.8
1.0
0
500
1000
1500
2000
2500
3000
3500
Survival time (days)
p−value: 0.00801888

Supplementary Figure 3. Number of TF with a significant change of activity between cancers and the corresponding healthy tissue of origin a function of mean tumour purity.

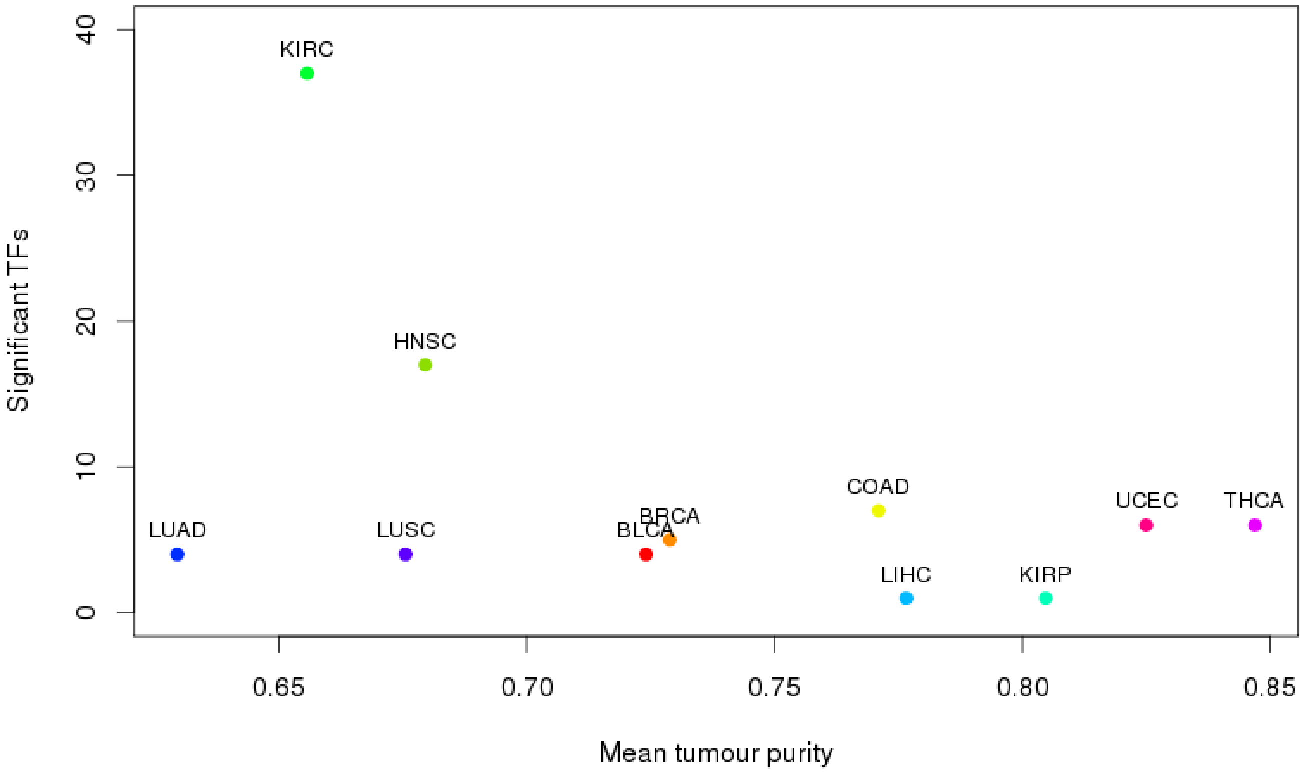

Supplementary Figure 4. Number of TF significantly associated to survival by a K-M test as a function of the number of deceased patients.

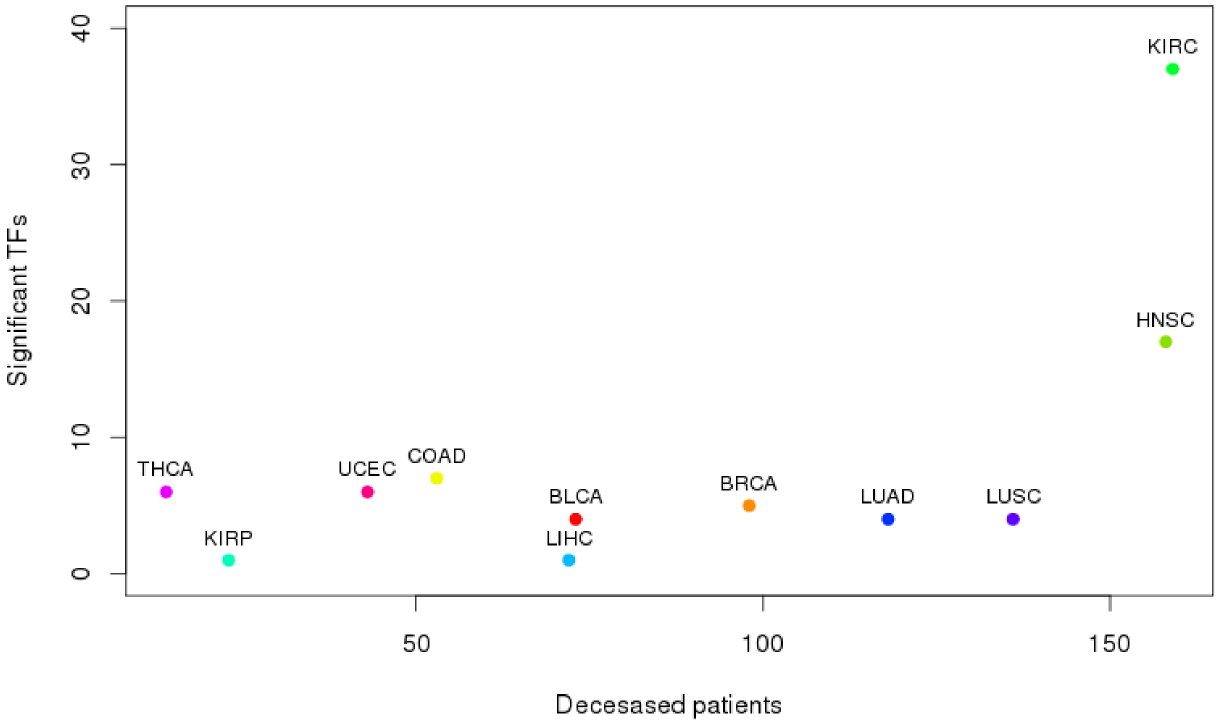

Supplement: Supplementary Figures and Table 2 [file srep39709-s4.pdf]
